# The Systems Biology Markup Language (SBML): Language Specification for Level 3 Version 2 Core Release 2

**DOI:** 10.1515/jib-2019-0021

**Published:** 2019-06-20

**Authors:** Michael Hucka, Frank T. Bergmann, Claudine Chaouiya, Andreas Dräger, Stefan Hoops, Sarah M. Keating, Matthias König, Nicolas Le Novère, Chris J. Myers, Brett G. Olivier, Sven Sahle, James C. Schaff, Rahuman Sheriff, Lucian P. Smith, Dagmar Waltemath, Darren J. Wilkinson, Fengkai Zhang

**Affiliations:** California Institute of Technology, Pasadena, CA, USA; Aix-Marseille University, CNRS, I2M, Marseille, France; Department of Computer Science, University of Tübingen, Tübingen, Germany; Computational Systems Biology of Infection and Antimicrobial-Resistant Pathogens, Institute for Biomedical Informatics (IBMI), University of Tübingen, Tübingen, Germany; German Center for Infection Research (DZIF), Tübingen, Germany; Virginia Bioinformatics Institute, Blacksburg, VA, USA; European Bioinformatics Institute, Cambridge, UK; Humboldt University Berlin, Berlin, Germany; Babraham Institute, Cambridge, UK; University of Utah, Salt Lake City, UT, USA; VU University Amsterdam, Amsterdam, Netherlands; University of Heidelberg, Heidelberg, Germany; University of Connecticut, Storrs, CT, USA; University of Washington, Seattle, WA, USA; University Medicine Greifswald, Greifswald, Germany; Newcastle University, Newcastle, Germany; NIAID/NIH, Bethesda, MD, USA

**Keywords:** Systems Biology Markup Language, Standards, Visualization, Representation

## Abstract

Computational models can help researchers to interpret data, understand biological functions, and make quantitative predictions. The **S**ystems **B**iology **M**arkup **L**anguage (SBML) is a file format for representing computational models in a declarative form that different software systems can exchange. SBML is oriented towards describing biological processes of the sort common in research on a number of topics, including metabolic pathways, cell signaling pathways, and many others. By supporting SBML as an input/output format, different tools can all operate on an identical representation of a model, removing opportunities for translation errors and assuring a common starting point for analyses and simulations. This document provides the specification for *Release 2* of *Version 2* of *SBML Level 3 Core*. The specification defines the data structures prescribed by SBML as well as their encoding in XML, the eXtensible Markup Language. *Release 2* corrects some errors and clarifies some ambiguities discovered in *Release 1*. This specification also defines validation rules that determine the validity of an SBML document, and provides many examples of models in SBML form. Other materials and software are available from the SBML project website at http://sbml.org/.

Automatically generated rough PDF by *ProofCheck* from River Valley Technologies Ltd

**Lucian P. Smith** is the corresponding author.
© 2019, Michael Hucka et al., published by Walter de Gruyter GmbH, Berlin/Boston.
This work is licensed under the Creative Commons Attribution 4.0 Public License.

## The Systems Biology Markup Language (SBML):
## Language Specification for Level 3 Version 2 Core

| | |
|---|---|
| Michael Hucka (Chair) | *California Institute of Technology, US* |
| Frank T. Bergmann | *California Institute of Technology, US* |
| Claudine Chaouiya | *Aix-Marseille University, CNRS, I2M, FR* |
| Andreas Dräger | *University of Tübingen, DE* |
| Stefan Hoops | *Virginia Bioinformatics Institute, US* |
| Sarah M. Keating | *European Bioinformatics Institute, GB* |
| Matthias König | *Humboldt University Berlin, DE* |
| Nicolas Le Novère | *Babraham Institute, GB* |
| Chris J. Myers | *University of Utah, US* |
| Brett G. Olivier | *VU University Amsterdam, NL* |
| Sven Sahle | *University of Heidelberg, DE* |
| James C. Schaff | *University of Connecticut, US* |
| Rahuman Sheriff | *European Bioinformatics Institute, GB* |
| Lucian P. Smith | *University of Washington, US* |
| Dagmar Waltemath | *University of Rostock, DE* |
| Darren J. Wilkinson | *Newcastle University, GB* |
| Fengkai Zhang | *NIAID/NIH, US* |

sbml-editors@sbml.org

**SBML Level 3 Version 2 Core**

**Release 2**

29 March 2019

Corrections and other changes to this SBML language specification may appear over time.
Notifications of new releases are broadcast on the mailing list sbml.org/forums/sbml-announce

The latest release of the SBML Level 3 Version 2 Core specification is available at
`http://sbml.org/specifications/sbml-level-3/version-2/core`

*This* release of the specification is available at
`http://sbml.org/specifications/sbml-level-3/version-2/core/release-2/`

The list of known issues in all releases of SBML Level 3 Version 2 Core is available at
`http://sbml.org/specifications/sbml-level-3/version-2/core/errata/`

Formal schemas for use with XML are available at
`http://sbml.org/specifications/sbml-level-3/version-2/schemas/`

SBML Systems Biology Markup Language

## Contents

**1 Introduction** **3**
- 1.1 Developments, discussions, and notifications of updates . . . 3
- 1.2 SBML Levels, Versions, and Releases . . . 3
- 1.3 SBML Level 3 Packages . . . 4
- 1.4 Document conventions . . . 4

**2 Overview of SBML** **9**

**3 Preliminary definitions and principles** **11**
- 3.1 Primitive data types . . . 11
- 3.2 Type ***SBase*** . . . 14
- 3.3 The `id` and `name` attributes on ***SBase*** . . . 18
- 3.4 Mathematical formulas in SBML Level 3 . . . 20

**4 SBML components** **34**
- 4.1 The SBML container . . . 34
- 4.2 Model . . . 36
- 4.3 Function definitions . . . 40
- 4.4 Unit definitions . . . 42
- 4.5 Compartments . . . 46
- 4.6 Species . . . 49
- 4.7 Parameters . . . 53
- 4.8 Initial assignments . . . 56
- 4.9 Rules . . . 59
- 4.10 Constraints . . . 66
- 4.11 Reactions . . . 68
- 4.12 Events . . . 79

**5 The Systems Biology Ontology and the `sboTerm` attribute** **91**
- 5.1 Principles . . . 91
- 5.2 Using SBO and `sboTerm` . . . 92
- 5.3 Relationships to the SBML `annotation` element . . . 97
- 5.4 Discussion . . . 98

**6 A standard format for the `annotation` element** **100**
- 6.1 Motivation . . . 100
- 6.2 XML namespaces in the standard annotation . . . 100
- 6.3 General syntax for the standard annotation . . . 101
- 6.4 Use of URIs . . . 102
- 6.5 Relation elements . . . 103
- 6.6 History . . . 105
- 6.7 Examples . . . 106

**7 Example models expressed in XML using SBML** **113**
- 7.1 A simple example application of SBML . . . 113
- 7.2 A simple example using the `conversionFactor` attribute . . . 115
- 7.3 An alternative formulation of the `conversionFactor` example . . . 118
- 7.4 Example of a discrete version of a simple dimerization reaction . . . 120
- 7.5 Example involving assignment rules . . . 124
- 7.6 Example involving algebraic rules . . . 126
- 7.7 Example with combinations of `boundaryCondition` and `constant` values on **Species** with **RateRule** objects . . . 128
- 7.8 Example of translation from a multi-compartmental model to ODEs . . . 130
- 7.9 Example involving function definitions . . . 133
- 7.10 Example involving *delay* functions . . . 134
- 7.11 Example involving events . . . 135
- 7.12 Example involving two-dimensional compartments . . . 137
- 7.13 Example of a reaction located at a membrane . . . 141
- 7.14 Example using an event with a non-persistent trigger and a delay . . . 143

**8 Recommended practices** **146**
- 8.1 Recommended practices concerning common SBML attributes and objects . . . 146
- 8.2 Recommended practices concerning specific SBML components . . . 148

**A Validation and consistency rules for SBML** **155**

**B A method for assessing whether an SBML model is overdetermined** **175**

**C A mathematical technique for maintaining unit consistency in a kinetic law with variable stoichiometry** **178**

**Acknowledgments** **179**

**References** **180**

## 1 Introduction

This document defines Version 2 of the **S**ystems **B**iology **M**arkup **L**anguage (SBML) Level 3 Core, an electronic model representation format for systems biology. SBML is oriented towards describing biological processes of the sort common in research on a number of topics, including metabolic pathways, cell signaling pathways, and many others. SBML is defined neutrally with respect to programming languages and software encoding; however, it is oriented primarily towards allowing models to be encoded using XML, the eXtensible Markup Language (Bray et al., 2004). This document contains many examples of SBML models written in XML. Formal schemas describing the syntax of SBML, as well as other materials and software, are available from the SBML project web site, `http://sbml.org/`.

The SBML project is not an attempt to define a universal language for representing quantitative models. The rapidly evolving views of biological function, coupled with the vigorous rates at which new computational techniques and individual tools are being developed today, are incompatible with a one-size-fits-all idea of a universal language. A more realistic alternative is to acknowledge the diversity of approaches and methods being explored by different software tool developers, and seek a common intermediate format—a *lingua franca*—enabling communication of the most essential aspects of the models.

The definition of the model description language presented here does not specify *how* programs should communicate or read/write SBML. We assume that for a simulation program to communicate a model encoded in SBML, the program will have to translate its internal data structures to and from SBML, use a suitable transmission medium and protocol, etc., but these issues are outside the scope of this document.

### 1.1 Developments, discussions, and notifications of updates

SBML has been, and continues to be, developed in collaboration with an international community of researchers and software developers. As in many projects, the primary medium for interactions between members is electronic messaging. Discussions about SBML take place on the combination web forum and mailing list `https://groups.google.com/d/forum/sbml-discuss`. The mailing list archives and a web-browser-based interface to the list are available at the same location.

A low-volume, broadcast-only web forum/mailing list is available where notifications of revisions to the SBML specification, notices of votes on SBML technical issues, and other critical matters are announced. This list is `https://groups.google.com/d/forum/sbml-announce` and anyone may subscribe to it freely. This list will never be used for advertising and its membership will never be disclosed. *It is vitally important that all users of SBML stay informed about new releases and other developments by subscribing to* `sbml-announce`, even if they do not wish to participate in discussions on `sbml-discuss`. Please visit `sbml.org/Forums/` for information about how to subscribe to the list as well as for access to the list archives.

### 1.2 SBML Levels, Versions, and Releases

Major editions of SBML are termed *levels* and represent substantial changes to the composition and structure of the language. The edition of SBML defined in this document, SBML Level 3, represents an evolution of the language resulting from the practical experiences of users and developers working with SBML since its introduction in the year 2001 (Hucka et al., 2001, 2003). All of the constructs of Level 1 can be mapped to Level 2; likewise, all of the constructs from Level 2 can be mapped to Level 3 (when Level 3 is considered in terms of the Core and Level 3 packages; see next section). In addition, a subset of Level 3 constructs can be mapped to Level 2, and a subset of Level 2 constructs can be mapped to Level 1. However, the levels remain distinct; a valid SBML Level 1 document is not a valid SBML Level 2 document, and so on.

In practice, once a new level of SBML is defined, no further development is undertaken on lower levels. An exception is made for the correction of problems and other issues that may be identified in the specifications of lower levels; such corrections are handled as described below.

Minor revisions of SBML are termed *versions* and constitute changes within a level to correct, adjust, and refine language features. The present document defines Level 3 *Version 2 Core*. A separate document provides information about the changes between SBML Level 3 and SBML Level 2.

Specification documents inevitably require minor editorial changes as their users discover errors and ambiguities. As a practical reality, these discoveries occur over time. In the context of SBML, such problems are formally announced publicly as *errata* in a given specification document. Borrowing concepts from the World Wide Web Consortium (Jacobs, 2004), we define SBML errata as changes of the following types: (a) formatting changes that do not result in changes to textual content; (b) corrections that do not affect conformance of software implementing support for a given combination of SBML level and version; and (c) corrections that *may* affect such software conformance, but add no new language features. A change that affects conformance is one that either turns conforming data, processors, or other conforming software into non-conforming software, or turns non-conforming software into conforming software, or clears up an ambiguity or insufficiently-documented part of the specification in such a way that software whose conformance was once unclear now becomes clearly conforming or non-conforming (Jacobs, 2004). In short, errata do not change the fundamental semantics or syntax of SBML; they clarify and disambiguate the specification and correct errors. (New syntax and semantics are only introduced in SBML versions and levels.) A public tracking system for reporting and monitoring such issues is available at http://sbml.org/issue-tracker, and we urge readers to use that system to report any issues found in this document.

SBML errata eventually result in new *Releases* of the specification. Each such release is numbered, with the first release of the specification being number 1. Subsequent releases of an SBML specification document contain a section describing the accumulated issues corrected since the first release. If errata are acknowledged for SBML Level 3 Version 2 Core since the publication of Release 1, they are listed publicly at http://sbml.org/specifications/sbml-level-3/version-2/core/errata/. Announcements of errata, updates to the SBML specification and other major changes are made on the https://groups.google.com/d/forum/sbml-announce web forum and mailing list.

### 1.3 SBML Level 3 Packages

SBML Level 3 is being developed as a modular language, with a central core comprising a self-sufficient model definition language, and extension packages layered on top of this core to provide additional, optional sets of features. This document defines the core of Level 3. The definition is based largely on SBML Level 2, with some modifications to address sources of problems found by experience with Level 2, and some simplifications to remove Level 2 constructs that are expected to be supported more thoroughly through SBML Level 3 packages. Section 4.1.3 on p. 35 describes the mechanism by which models defined in SBML Level 3 can declare which packages they use.

The specifications for packages available for SBML Level 3 is maintained separately on the SBML website at http://sbml.org/Documents/Specifications. A list of packages is not provided in this specification document (i.e., for Level 3 Core) because the development of packages for Level 3 proceeds independently, and new ones may be introduced over time after Level 3 Core is published. The SBML website provides information about ongoing activities in this area, as well as about the process whereby individuals and groups may propose new packages.

### 1.4 Document conventions

In this section, we describe the conventions used in this specification document to communicate information more effectively.

#### 1.4.1 Color conventions

Throughout this document, we use coloring to carry additional information for the benefit of those viewing the document on media that can display color:

- We use red color in text and figures to indicate changes between this version of the specification, namely SBML Level 3 Version 2 Core Release 2, and the *most recent previous release* of the specification (which, for the present case, is SBML Level 3 Version 2 Core *Release 1*). The changes may be either additions or deletions of text; in the case of deletions, entire sentences, paragraphs or sections are colored to indicate a change has occurred inside them. In UML diagrams, a red *box* is used to indicate a deletion, and red *text* to indicate a semantic change or addition.

- We use blue color in text to indicate a hyperlink from one point in this document to another. Clicking your computer's pointing device on blue-colored text will cause a jump to the section, figure, table or page to which the link refers. (Of course, this capability is only available when using electronic formats that support hyperlinking, such as PDF and HTML.)

#### 1.4.2 Typographical conventions for names

We use the following typographical conventions to distinguish objects and data types from other entities:

***AbstractClass***: Abstract classes are classes that are never instantiated directly, but rather serve as parents of other object classes. Their names begin with a capital letter and they are printed in a slanted, bold, sans-serif typeface. In electronic document formats, the class names are also hyperlinked to their definitions in the specification. For example, in the PDF and HTML versions of this document, clicking on the word ***SBase*** will send the reader to the section containing the definition of this class.

**Class**: Names of ordinary (concrete) classes begin with a capital letter and are printed in an upright, bold, sans-serif typeface. In electronic document formats, the class names are also hyperlinked to their definitions in the specification. For example, in the PDF and HTML versions of this document, clicking on the word **Species** will send the reader to the section containing the definition of this class.

`SomeThing`, `otherThing`: Attributes of classes, data type names, literal XML, and generally all tokens *other* than SBML UML class names, are printed in an upright typewriter typeface. Primitive types defined by SBML begin with a capital letter; SBML also makes use of primitive types defined by XML Schema 1.0 (Biron and Malhotra, 2000; Fallside, 2000; Thompson et al., 2000), but unfortunately, XML Schema does not follow any capitalization convention and primitive types drawn from the XML Schema language may or may not start with a capital letter.

#### 1.4.3 UML notation

Previous specifications of SBML used a notation that was at one time (in the days of SBML Level 1) fairly close to UML, the Unified Modeling Language (Eriksson and Penker, 1998; Oestereich, 1999), though many details were omitted from the UML diagrams themselves. Over the years, the notation used in successive specifications of SBML grew increasingly less UML-like. Beginning with SBML Level 2 Version 3, we have completely overhauled the specification's use of UML and once again define the XML syntax of SBML using, as much as possible, proper and complete UML 1.0. We then systematically map this UML notation to XML. In the rest of this section, we summarize the UML notation used in this document and explain the few embellishments needed to support transformation to XML form.

We see three main advantages to using UML as a basis for defining SBML data objects. First, compared to using other notations or a programming language, the UML visual representations are generally easier to grasp by readers who are not computer scientists. Second, the notation is implementation-neutral: the objects can be encoded in any concrete implementation language—not just XML, but C, Java and other languages as well. Third, UML is a de facto industry standard that is documented in many resources. Readers are therefore more likely to be familiar with it than other notations.

##### *Object class definitions*

Object classes in UML diagrams are drawn as simple tripartite boxes, as shown in Figure 1 on the next page (left). UML allows for operators as well as data attributes to be defined, but SBML only uses data attributes, so all SBML class diagrams use only the top two portions of a UML class box (Figure 1 on the following page, right).

As mentioned above, the names of ordinary (concrete) classes begin with a capital letter and are printed in an upright, bold, sans-serif typeface. The names of attributes begin with a lower-case letter and generally use a mixed case (sometimes called "camel case") style when the name consists of multiple words. Attributes and their data types appear in the part below the class name, with one attribute defined per line. The colon character on each line separates the name of the attribute (on the left) from the type of data that it stores (on the right). The subset of data types permitted for SBML attributes is given in Section 3.1 on p. 11.

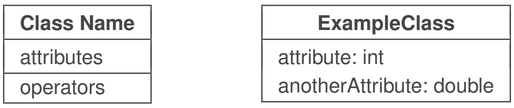

***Figure 1:*** *(Left) The general form of a UML class diagram. (Right) Example of a class diagram of the sort seen in SBML. SBML classes never use operators, so SBML class diagrams only show the top two parts.*

In the right-hand diagram of Figure 1, the symbols `attribute` and `anotherAttribute` represent attributes of the object class **ExampleClass**. The data type of `attribute` is `int`, and the data type of `anotherAttribute` is `double`. In the scheme used by SBML for translating UML to XML, object attributes map directly to XML attributes. Thus, in XML, **ExampleClass** would yield an element of the form `<`***`element`***` attribute="42" anotherAttribute="10.0">`.

Notice that the element name is not `<ExampleClass ...>`. Somewhat paradoxically, the name of the element is *not* the name of the UML class defining its structure. The reason for this may be subtle at first, but quickly becomes obvious: object classes define the form of an object's *content*, but a class definition by itself does not define the *label* or symbol referring to an instance of that content. It is this label that becomes the name of the XML element. In XML, this symbol is most naturally equated with an element name. This point will hopefully become clearer with additional examples below.

#### *Subelements*

We use UML *composition* to indicate a class object can have other class objects as parts. Such containment hierarchies map directly to element-subelement relationships in XML. Figure 2 gives an example.

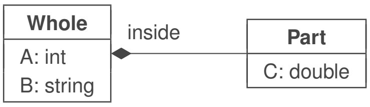

***Figure 2:*** *Example illustrating composition: the definition of one class of objects employing another class of objects in a part-whole relationship. In this particular example, an instance of a* ***Whole*** *class object must contain exactly one instance of a* ***Part*** *class object, and the label referring to the* ***Part*** *class object is* `inside`*. In XML, this symbol becomes the name of a subelement and the content of the subelement follows the definition of* ***Part****.*

The line with the black diamond indicates composition, with the diamond located on the "container" side and the other end located at the object class being contained. The label on the line is used to refer to instances of the contained object, which in XML, maps directly to the name of an XML element. The class pointed to by the composition relationship (**Part** in Figure 2) defines the *contents* of that element. Thus, if we are told that some element named `barney` is of class `Whole`, the following is an example XML fragment consistent with the class definition of Figure 2:

```
<barney A="110" B="some string">
    <inside C="444.4">
</barney>
```

Sometimes numbers are placed above the line near the "contained" side of a composition to indicate how many instances can be contained. The common cases in SBML are the following: `[0..*]` to signify a list containing zero or more; `[1..*]` to signify a list containing at least one; and `[0..1]` to signify exactly zero or one. The absence of a numerical label means "exactly 1". This notation appears throughout this specification document.

#### *Inheritance*

Classes can inherit properties from other classes. Since SBML only uses data attributes and not operators, inheritance in SBML simply involves data attributes from a parent class being inherited by child classes. Inheritance is indicated by a line between two classes, with an open triangle next to the parent class; Figure 3 illustrates this. In this example, the instances of object class **Child** would have not only attributes `C` and `D`, but also attributes `A` and `B`. All of these attributes would be required (not optional) on instances of class **Child** because they are mandatory on both the **Parent** and **Child** classes.

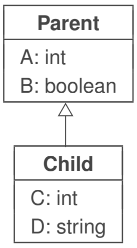

***Figure 3:*** *Inheritance.*

#### *Additional notations for XML purposes*

Not everything is easily expressed in plain UML. For example, it is often necessary to indicate some constraints placed on the values of an attribute. In computer programming uses of UML, such constraints are often expressed using Object Constraint Language (OCL), but since we are most interested in the XML rendition of SBML, in this specification we use XML Schema 1.0 (when possible) as the language for expressing value constraints. Constraints on the values of attributes are written as expressions surrounded by braces ({ }) after the data type declaration, as in the example of Figure 4.

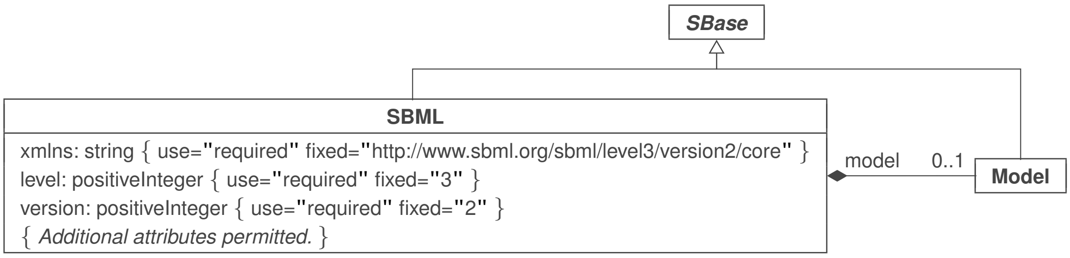

***Figure 4:*** *A more complex example definition drawing on the concepts introduced so far in this section. Both* ***SBML*** *and* ***Model*** *are derived from* **SBase***; further,* ***SBML*** *contains a single* ***Model*** *object named* `model`*. Note the constraints on the values of the attributes in* ***SBML****; they are enclosed in braces and often written in XML Schema language. The particular constraints here state that the* `xmlns`, `level` *and* `version` *attributes must be present, and that the values are fixed as indicated. In addition, other attributes are permitted (for example, such as those added by Level 3 packages).*

In other situations, when something cannot be concisely expressed using a few words of XML Schema, we write constraints using English language descriptions surrounded by braces ({ }). To help distinguish these from literal XML Schema, we set the English text in a slanted typeface. The text accompanying all SBML component definitions provides explanations of the constraints and any other conditions applicable to the use of the components.

#### *XML Namespace*

XML defines how to declare namespaces on XML elements. In most SBML documents, the SBML namespace will be the default namespace for the document, and thus individual elements in the document will not need to be prefixed with the SBML namespace. However, it is legal to declare an SBML namespace and use it to place SBML elements explicitly in that namespace. On the other hand, it is possible to create documents with no default namespace, in which case, elements in the document *must* be prefixed.

In contrast to element names, XML *attribute* names are completely defined by the XML element in which they appear, and never have a default namespace defined for them. The XML element itself declares whether any attributes appearing on it should be written with a namespace prefix.

For SBML Core, any attribute shown in any UML diagram in this specification *must* be written *without* a namespace prefix in an SBML document, even if a namespace prefix is defined in the SBML document.

The convention for SBML packages is to allow attributes to be defined *either* with no namespace prefix, *or* to be defined with that package's namespace as a prefix, for any new element defined by that package. When a package extends an existing SBML element to have a new attribute, the convention is to *require* that this attribute be prefixed with that package's namespace. Previously-released SBML packages did not make this explicit, but are assumed to follow this convention. As these packages undergo updates in the future, these rules will be made explicit.

## 2 Overview of SBML

The following is an example of a simple network of biochemical reactions that can be represented in SBML:

$$S_1 \xrightarrow{k_1[S_1]/([S_1]+k_2)} S_2$$
$$S_2 \xrightarrow{k_3[S_2]} S_3 + S_4$$

In this particular set of chemical equations above, the symbols in square brackets (e.g., "$[S_1]$") represent concentrations of molecular species, the arrows represent reactions, and the formulas above the arrows represent the rates at which the reactions take place. (And while this example uses concentrations, it could equally have used other measures such as molecular counts.) Broken down into its constituents, this model contains a number of components: reactant species, product species, reactions, reaction rates, and parameters in the rate expressions. To analyze or simulate this network, additional components must be made explicit, including compartments for the species, and units on the various quantities.

SBML allows models of arbitrary complexity to be represented. Each type of component in a model is described using a specific type of data object that organizes the relevant information. The top level of an SBML model definition consists of lists of these components, with every list being optional:

| | |
|---|---|
| *beginning of model definition* | |
| *list of function definitions (optional)* | (Section 4.3) |
| *list of unit definitions (optional)* | (Section 4.4) |
| *list of compartments (optional)* | (Section 4.5) |
| *list of species (optional)* | (Section 4.6) |
| *list of parameters (optional)* | (Section 4.7) |
| *list of initial assignments (optional)* | (Section 4.8) |
| *list of rules (optional)* | (Section 4.9) |
| *list of constraints (optional)* | (Section 4.10) |
| *list of reactions (optional)* | (Section 4.11) |
| *list of events (optional)* | (Section 4.12) |
| *end of model definition* | |

The meaning of each component is as follows:

*Function definition*: A named mathematical function that may be used throughout the rest of a model.

*Unit definition*: A named definition of a new unit of measurement. Named units can be used in the expression of quantities in a model.

*Compartment*: A well-stirred container of finite size where species may be located. Compartments may or may not represent actual physical structures.

*Species*: A pool of entities of the same kind located in a compartment and participating in reactions (processes). In biochemical network models, common examples of species include ions, proteins and other molecules; however, in practice, an SBML species can be any kind of entity that makes sense in the context of a given model.

*Parameter*: A quantity with a symbolic name. In SBML, the term *parameter* is used in a generic sense to refer to named quantities regardless of whether they are constants or variables in a model. SBML Level 3 provides the ability to define parameters that are global to a model as well as parameters that are local to a single reaction.

*Initial Assignment*: A mathematical expression used to determine the initial conditions of a model. This type of object can only be used to define how the value of a symbol can be calculated from other values and symbols at the start of simulated time.

*Rule*: A mathematical expression added to the set of equations constructed based on the reactions defined in a model. Rules can be used to define how a symbol's value can be calculated from other symbols, or used to define the rate of change of a symbol. The set of rules in a model can be used with the reaction rate equations to determine the behavior of the model with respect to time. Rules constrain the model for the entire duration of simulated time.

*Constraint*: A means of detecting out-of-bounds conditions during a dynamical simulation and optionally issuing diagnostic messages. Constraints are defined by an arbitrary mathematical expression computing a true/false value from model symbols. An SBML constraint applies at all instants of simulated time; however, the set of constraints in model should not be used to *determine* the behavior of the model with respect to time.

*Reaction*: A statement describing some transformation, transport or binding process that can change the amount of one or more species. For example, a reaction may describe how certain entities (reactants) are transformed into certain other entities (products). Reactions have associated kinetic rate expressions describing how quickly they take place.

*Event*: A statement describing an instantaneous, discontinuous change in one or more symbols of any type (species, compartment, parameter, etc.) when a triggering condition is satisfied.

A software package can read an SBML model description and translate it into its own internal format for model analysis. For example, a package might provide the ability to simulate the model by constructing differential equations representing the network and then perform numerical time integration on the equations to explore the model's dynamic behavior. By supporting SBML as an input and output format, different software tools can all operate on an identical external representation of a model, removing opportunities for errors in translation and assuring a common starting point for analyses and simulations.

## 3 Preliminary definitions and principles

This section covers certain concepts and constructs that are used repeatedly in the rest of SBML Level 3.

### 3.1 Primitive data types

Most primitive types in SBML are taken from the data types defined in XML Schema 1.0 (Biron and Malhotra, 2000; Fallside, 2000; Thompson et al., 2000). A few other primitive types are defined by SBML itself. What follows is a summary of the XML Schema types and the definitions of the SBML-specific types. Note that, while we have tried to provide accurate and complete summaries of the XML Schema types, the following descriptions should not be taken to be normative definitions of these types. Readers should consult the XML Schema 1.0 specification for the normative definitions of the XML data types used by SBML.

#### *3.1.1 Type* `string`

The XML Schema 1.0 type `string` is used to represent finite-length strings of characters. The characters permitted to appear in XML Schema `string` include all Unicode characters (Unicode Consortium, 1996) except for two delimiter characters, 0xFFFE and 0xFFFF (Biron and Malhotra, 2000). In addition, the following quoting rules specified by XML for character data (Bray et al., 2004) must be obeyed:

- The ampersand (`&`) character must be escaped using the entity `&`.
- The apostrophe (`'`) and quotation mark (`"`) characters must be escaped using the entities `'` and `"`, respectively, when those characters are used to delimit a string attribute value.

Other XML built-in character or entity references, e.g., `<` and `&x1A;`, are permitted in strings.

#### *3.1.2 Type* `boolean`

The XML Schema 1.0 type `boolean` is used for SBML object attributes that represent binary true/false values. XML Schema 1.0 defines the possible literal values of `boolean` as the following: "`true`", "`false`", "`1`", and "`0`". The value "`1`" maps to "`true`" and the value "`0`" maps to "`false`" in attribute values.

Note that there is a discrepancy between the value spaces of type `boolean` as defined by XML Schema 1.0 and MathML: the latter uses only "`true`" and "`false`" to represent Boolean values, with "`0`" and "`1`" reserved for numbers. Software tools should take care not to attempt using "`0`" and "`1`" as Boolean values in MathML expressions. See further discussion in Section 3.4.4 on p. 24.

#### *3.1.3 Type* `int`

The XML Schema 1.0 type `int` is used to represent decimal integer numbers in SBML. The literal representation of an `int` is a finite-length sequence of decimal digit characters with an optional leading sign ("`+`" or "`-`"). If the sign is omitted, "`+`" is assumed. The value space of `int` is the same as a standard 32-bit signed integer in programming languages such as C, i.e., 2147483647 to $-2147483648$.

#### *3.1.4 Type* `positiveInteger`

The XML Schema 1.0 type `positiveInteger` is used to represent nonzero, nonnegative, decimal integers: i.e., 1, 2, 3, . . . . The literal representation of an integer is a finite-length sequence of decimal digit characters, optionally preceded by a positive sign ("`+`"). There is no restriction on the absolute size of `positiveInteger` values in XML Schema; however, the only situations where this type is used in SBML involve very low-numbered integers. Consequently, applications may safely treat `positiveInteger` as unsigned 32-bit integers.

#### *3.1.5 Type* `double`

The XML Schema 1.0 type `double` is the data type of floating-point numerical quantities in SBML. It is restricted to IEEE double-precision 64-bit floating-point type IEEE 754-1985. The value space of `double`

consists of (a) the numerical values $m \cdot 2^x$, where $m$ is an integer whose absolute value is less than $2^{53}$, and $x$ is an integer between -1075 and 970, inclusive, (b) the special value positive infinity (**INF**), (c) the special value negative infinity (**-INF**), and (d) the special value not-a-number (**NaN**). The order relation on the values is the following: $x < y$ if and only if $y - x$ is positive for values of $x$ and $y$ in the value space of **double**. Positive infinity is greater than all other values other than **NaN**. **NaN** is equal to itself but is neither greater nor less than any other value in the value space. (Software implementors should consult the XML Schema 1.0 definition of **double** for additional details about equality and relationships to IEEE 754-1985.)

The general form of **double** numbers is "$x$**e**$y$", where $x$ is a decimal number (the mantissa), "**e**" is a separator character, and $y$ is an exponent; the meaning of this is "$x$ multiplied by 10 raised to the power of $y$", i.e., $x \cdot 10^y$. More precisely, a **double** value consists of a mantissa with an optional leading sign ("+" or "-"), optionally followed by the character **E** or **e** followed by an integer (the exponent). The mantissa must be a decimal number: an integer optionally followed by a period (**.**) optionally followed by another integer. If the leading sign is omitted, "+" is assumed. An omitted **E** or **e** (and associated exponent) means that a value of 0 is assumed for the exponent. If the **E** or **e** is present, it must be followed by an integer, or else an error results. The integer exponent must consist of a decimal number optionally preceded by a leading sign ("+" or "-"). If the sign is omitted, "+" is assumed. The following are examples of legal literal **double** values:

```
-1E4,   +4,   234.234e3,   6.02E-23,   0.3e+11,   2,   0,   -0,   INF,   -INF,   NaN
```

As described in Section 3.4, SBML uses a subset of the MathML 2.0 standard (W3C, 2000b) for expressing mathematical formulas in XML. This is done by stipulating that the MathML language be used whenever a mathematical formula must be written into an SBML model. Doing this, however, requires facing two problems: first, the syntax of numbers in scientific notation ("e-notation") is different in MathML from that just described for **double**, and second, the value space of integers and floating-point numbers in MathML is not defined in the same way as in XML Schema 1.0. We elaborate on these issues in Section 3.4.2; here we summarize the solution taken in SBML. First, within MathML, the mantissa and exponent of numbers in "e-notation" format must be separated by one <sep/> element. This leads to numbers of the form <cn type="e-notation"> 2 <sep/> -5 </cn>. Second, SBML stipulates that the representation of numbers in MathML expressions obey the same restrictions on values as defined for types **double** and **int** (Section 3.1.3).

#### 3.1.6 Type ID

The XML Schema 1.0 type **ID** is identical to the XML 1.0 type **ID**. The literal representation of this type consists of strings of characters restricted as summarized in Figure 5. Note that this definition is different from that in the XML 1.1 specification, where the definition of the **ID** type was expanded and changed. SBML uses the original 1.0 version.

```
NameChar   ::=   letter | digit | '.' | '-' | '_' | ':' | CombiningChar | Extender
ID         ::=   ( letter | '_' | ':' ) NameChar*
```

***Figure 5:*** *Type* **ID** *expressed in the variant of BNF used by the XML 1.0 specification (Bray et al., 2004). The characters* **(** *and* **)** *are used for grouping, the character* ***** *indicates "zero or more times", and the character* **|** *indicates "or". The production* **letter** *consists of the basic upper and lower case alphabetic characters of the Latin alphabet along with a large number of related characters defined by Unicode 2.0; similarly, the production* **digit** *consists of the numerals* **0..9** *along with related Unicode 2.0 characters. The* **CombiningChar** *production is a list of characters that add such things as accents to the preceding character. (For example, the Unicode character* **#x030A** *when combined with 'a' produces 'å'.) The* **Extender** *production is a list of characters that extend the shape of the preceding character. Please consult the XML 1.0 specification (Bray et al., 2004) for the complete definitions of* **letter**, **digit**, **CombiningChar**, *and* **Extender**.

In SBML, type **ID** is the data type of the **metaid** attribute on ***SBase***, described in Section 3.2 on p. 14. An important aspect of **ID** is the XML requirement that a given value of **ID** must be unique throughout an XML document. All data values of type **ID** are considered to reside in a single common global namespace spanning the entire XML document, regardless of the attribute where type **ID** is used and regardless of the level of nesting of the objects (or XML elements).

#### 3.1.7 *Type* SId

The type SId is the type of the id attribute found on the abstract class ***SBase***, and thus available on every component class derived from ***SBase*** in SBML Level 3 Version 2 Core. SId is a data type derived from the basic XML type string, but with restrictions about the characters permitted and the sequences in which those characters may appear. The definition is shown in Figure 6.

```
letter  ::=  'a'..'z','A'..'Z'
digit   ::=  '0'..'9'
idChar  ::=  letter | digit | '_'
SId     ::=  ( letter | '_' ) idChar*
```

***Figure 6:*** *The definition of the type* SId*. (Please see the caption of Figure 5 for an explanation of the notation.)*

The equality of SId values is determined by an exact character sequence match; i.e., comparisons of these identifiers must be performed in a case-sensitive manner. This applies to all uses of SId.

Type SId is purposefully not derived from the XML ID type (Section 3.1.6). Using ID would force all SBML identifiers to exist in a single global namespace, affecting not only **Reaction** local parameter definitions but also SBML packages for (e.g.) hierarchical model composition. Further, the use of ID for SBML identifiers would have limited utility because MathML 2.0 ci elements are not of the type IDREF (see Section 3.4). Since the IDREF/ID linkage cannot be exploited in MathML constructs, the utility of XML's ID type is greatly reduced. Finally, unlike ID, SId does not include Unicode character codes; the identifiers are plain text.

#### 3.1.8 *Type* SIdRef

Type SIdRef is used for all attributes that refer to identifiers of type SId in a **Model** object. This type is derived from SId, but with the restriction that the value of an attribute having type SIdRef must equal the value of *some* SId attribute in the model where it appears. In other words, a SIdRef value must be an existing identifier in a model.

As with SId, the equality of SIdRef values is determined by exact character sequence match; i.e., comparisons of these identifiers must be performed in a case-sensitive manner.

#### 3.1.9 *Type* UnitSId

The type UnitSId is derived from SId (Section 3.1.7) and has identical syntax. The UnitSId type is used as the data type for the identifiers of units (Section 4.4.1 on p. 42) in SBML objects. The purpose of having a separate type for such identifiers is to enable the space of possible unit identifier values to be separated from the space of all other identifier values in SBML. The equality of UnitSId values is determined by an exact character sequence match; i.e., comparisons of these identifiers must be performed in a case-sensitive manner.

A number of reserved symbols are defined in the space of values of UnitSId. These reserved symbols are the list of base unit names defined in Table 2 on p. 44.

#### 3.1.10 *Type* UnitSIdRef

Type UnitSIdRef is used for all attributes that refer to identifiers of type UnitSId, which are the identifiers of units (Section 4.4.1 on p. 42) in SBML objects. This type is derived from UnitSId, but with the restriction that the value of an attribute having type UnitSIdRef must match either the value of a UnitSId attribute in the model, or one of the base units in Table 2 on p. 44. In other words, the value of a UnitSIdRef attribute must be an existing unit identifier in the model or in SBML.

As with UnitSId, the equality of UnitSIdRef values is determined by exact character sequence match; i.e., comparisons of these identifiers must be performed in a case-sensitive manner.

#### 3.1.11 Type LocalSId

The `LocalSId` type is used as the data type for the `id` attribute of **LocalParameter**. The type `LocalSId` is derived from `SId` (Section 3.1.7) and has identical syntax; it is defined as its own type to make it easier to specify a modified scope for identifiers referenced by mathematical formulas inside **Reaction** objects.

As explained in Section 3.3.1 on p. 18, each **Reaction** object introduces a local namespace for local parameter identifiers (i.e., the `id` attribute values of **LocalParameter** objects within that **Reaction**). The consequence is that any `math` `<ci>` child of the **KineticLaw** whose text matches the `id` value of a **LocalParameter** in that same **KineticLaw** is taken to refer to that **LocalParameter** object, and not to any other object that may have the same `id` value in the rest of the **Model**. The `LocalSId` value of a **LocalParameter** object's `id` attribute is not part of the `SId` namespace of the model, nor of the `LocalSId` namespace of any other **Reaction** object.

#### 3.1.12 Type SBOTerm

The type `SBOTerm` is used as the data type of the attribute `sboTerm` on ***SBase***. The type consists of strings of characters matching the restricted pattern described in Figure 7.

```
digit     ::=  '0'..'9'
SBOTerm   ::=  'SBO:' digit digit digit digit digit digit digit
```

***Figure 7:*** *The definition of* `SBOTerm`*. (Please see the caption of Figure 5 for an explanation of the notation.)*

Examples of valid string values of type `SBOTerm` are "`SBO:0000014`" and "`SBO:0003204`". These values are meant to be the identifiers of terms from an ontology whose vocabulary describes entities and processes in computational models. Section 5 on p. 91 provides more information about the ontology and principles for the use of these terms in SBML models.

### 3.2 Type *SBase*

Nearly every object composing an SBML Level 3 model definition has a specific data type that is derived directly or indirectly from a single abstract type called ***SBase***. In addition to serving as the parent class for most other classes of objects in SBML, this base type is designed to allow a modeler or a software package to attach arbitrary information to each major element or list in an SBML model. The definition of ***SBase*** is presented in Figure 8.

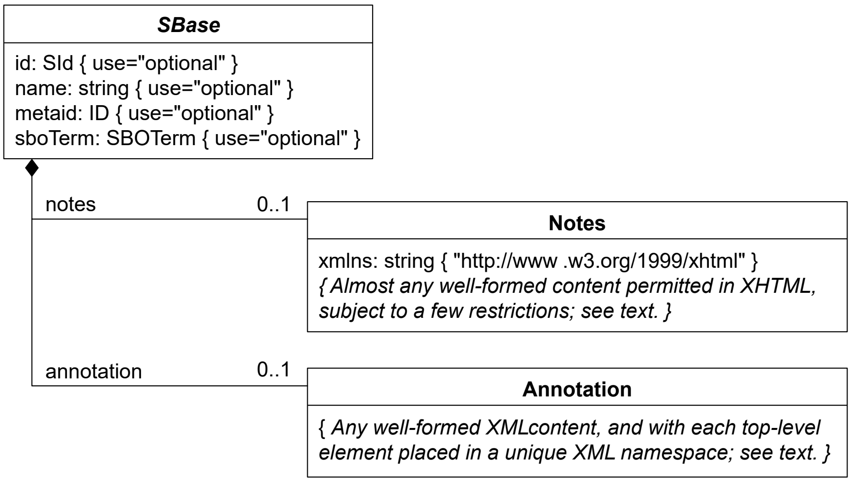

***Figure 8:*** *The definition of abstract class* **SBase***. Please refer to Section 1.4 for a summary of the notation used here.*

***SBase*** contains four attributes and two subobjects, all of which are optional: `id`, `name`, `metaid`, `sboTerm`, **Notes**, and **Annotation**. These are discussed separately in the following subsections.

#### 3.2.1 The id attribute

The id attribute is an optional attribute on the ***SBase*** class. The id attribute value on an object serves as its *identifier*. The data type of id on ***SBase*** is SId (Section 3.1.7 on p. 13). Every SId attribute value in a **Model** object must be unique within that **Model** object. In certain well-specified circumstances, other SBML objects can refer to the object using its identifier.

SBML components may stipulate that id takes on a value taken from a type derived from SId in order to impose additional requirements or restrictions, such as scope restrictions. (For example, this is the case with unit identifiers and UnitSId, Section 3.1.9 on p. 13.) When SBML Level 3 packages define new classes that inherit from ***SBase***, they may also refine the type of the id attribute on those classes. In addition, although id is optional on ***SBase***, object classes derived from ***SBase*** may stipulate that id is a required attribute for those classes. Outside of the specializations of SId, the set of SId values in a model is referred to as the "SId namespace of the model".

In lower Level/Version combinations of SBML, the attributes id and name are defined on individual object subclasses. The placement of these attributes on ***SBase*** in SBML Level 3 Version 2 is a notable change in the internal SBML class hierarchy, although it has no practical effect for any class that has mathematical meaning—all the same objects with meaning (e.g., species, parameters, etc.) have exactly the same id attributes as in SBML Level 3 Version 1. This topic is discussed further in Section 3.3 on p. 18.

#### 3.2.2 The name attribute

The attribute name is an optional attribute on ***SBase*** of type string. In contrast to the id attribute, the name attribute is not intended to be used for cross-referencing purposes within a model. Its purpose instead is to provide a human-readable label for a component. The data type of name is the type string defined in XML Schema (Biron and Malhotra, 2000; Thompson et al., 2000) and discussed further in Section 3.1 on p. 11. SBML imposes no restrictions as to the content of name attributes beyond those restrictions defined by the string type in XML Schema. In addition, there are no restrictions on the uniqueness of name values in a model (unlike the restrictions on id values discussed in Section 3.3.1 on p. 18).

#### 3.2.3 The metaid attribute

The metaid attribute is present for supporting metadata annotations using RDF (Resource Description Format; Lassila and Swick, 1999). It has a data type of XML ID (the XML identifier type; see Section 3.1.6 on p. 12), which means each metaid value must be globally unique within an SBML file. The metaid value serves to identify a model component for purposes such as referencing that component from metadata placed within annotation elements (see Section 3.2.6 on the following page). Such metadata can use RDF description elements, in which an RDF attribute called "rdf:about" points to the metaid identifier of an object defined in the SBML model. This topic is discussed in greater detail in Section 6 on p. 100.

#### 3.2.4 The sboTerm attribute

The attribute called sboTerm is provided on ***SBase*** to support the use of the Systems Biology Ontology (SBO; see Section 5 on p. 91). When a value is given to this attribute, it must conform to the data type SBOTerm (Section 3.1.12 on the previous page). SBO terms are a type of optional annotation, and each different class of SBML object derived from ***SBase*** imposes its own requirements about the values permitted for sboTerm. Specific details on the permitted values are provided with the definitions of SBML classes throughout this specification document, and a broader discussion is provided in Section 5 on p. 91.

#### 3.2.5 Notes

The subcomponent **Notes** in ***SBase*** is a container for XHTML 1.0 (Pemberton et al., 2002) content. It is intended to serve as a place for storing optional information intended to be seen by humans. An example use of **Notes** would be to contain formatted user comments about the model element in which the **Notes** object is enclosed. Every object derived directly or indirectly from type ***SBase*** can have a separate **Notes** object instance, allowing users considerable freedom when adding comments to their models.

*XML namespace requirements for notes*

In XML, the `notes` elements must declare the use of the XHTML XML namespace. This can be done in multiple ways. One way is to place a namespace declaration for the appropriate namespace URI (which is `http://www.w3.org/1999/xhtml`) on the top-level **SBML** object (see Section 4.1 on p. 34) and then reference the namespace in the `notes` content using a prefix. The following example illustrates this approach:

```
<sbml xmlns="http://www.sbml.org/sbml/level3/version2/core" level="3" version="2"
      xmlns:xhtml="http://www.w3.org/1999/xhtml">
  ...
  <notes>
    <xhtml:body>
      <xhtml:center><xhtml:h2>A Simple Mitotic Oscillator</xhtml:h2></xhtml:center>
      <xhtml:p>A minimal cascade model for the mitotic oscillator
      involving cyclin and cdc2 kinase</xhtml:p>
    </xhtml:body>
  </notes>
  ...
```

Another approach is to declare the XHTML namespace within the `notes` content itself, as in the following example:

```
...
<notes>
  <body xmlns="http://www.w3.org/1999/xhtml">
    <center><h2>A Simple Mitotic Oscillator</h2></center>
    <p>A minimal cascade model for the mitotic oscillator involving cyclin and cdc2 kinase</p>
  </body>
</notes>
...
```

The `xmlns="http://www.w3.org/1999/xhtml"` declaration on `body` as shown above changes the default XML namespace within it, such that all of its content is by default in the XHTML namespace. This is a particularly convenient approach because it obviates the need to prefix every element with a namespace prefix (i.e., "`xhtml:`" in the earlier example). Other approaches are also possible.

*The XHTML content of* ***notes***

SBML Level 3 does not require the content of a **Notes** object to be any particular XHTML element; the content simply should be any well-formed XHTML content. There is only one restriction, and it comes from the requirements of XML: the `notes` element must not contain an XML declaration or a DOCTYPE declaration. That is, `notes` must *not* contain

```
<?xml version="1.0" encoding="UTF-8"?>
```

nor the following (where the following is only one specific example of a DOCTYPE declaration):

```
<!DOCTYPE html PUBLIC "-//W3C//DTD XHTML 1.0 Strict//EN"
 "http://www.w3.org/TR/xhtml1/DTD/xhtml1-strict.dtd">
```

#### 3.2.6 Annotation

Whereas **Notes** is a container for content to be shown directly to humans, **Annotation** is a container for optional software-generated content *not* meant to be shown to humans. Every object derived from ***SBase*** can have its own **Annotation** object instance. In XML, the **Annotation** content type is `any`, allowing essentially arbitrary well-formed XML data content. SBML places only a few restrictions on the organization of the content; these are intended to help software tools read and write the data as well as help reduce conflicts between annotations added by different tools.

*The use of XML namespaces in annotations*

At the outset, software developers should keep in mind that multiple software tools may attempt to read and write annotation content. To reduce the potential for collisions between annotations written by different applications, SBML Level 3 Version 2 Core stipulates that tools must use XML namespaces (Bray et al., 1999) to specify the intended vocabulary of every annotation. The namespace may be declared on the root element using that namespace, or on any XML element containing it, up to and including the root `<sbml>` object itself. The application's developers must choose a URI (*Universal Resource Identifier*; Harold and Means 2001; W3C 2000a) reference that uniquely identifies the vocabulary the application will use, and a prefix string for the annotations. Here is an example. Suppose an application uses the URI `http://www.mysim.org/ns` and the prefix `mysim` when writing annotations related to molecules. The content of an annotation might look like the following:

```
<annotation>
    <mysim:molecule xmlns:mysim="http://www.mysim.org/ns"
         mysim:weight="18.02" mysim:atomCount="3"/>
</annotation>
```

In this particularly simple example, the content consists of a single XML element (`molecule`) with two attributes (`weight`, `atomCount`), all of which are prefixed by the string `mysim`. (Presumably this particular content would have meaning to the hypothetical application in question.) The content in this particular example is small, but it should be clear that there could easily have been an arbitrarily large amount of data placed inside the `mysim:molecule` element.

Similarly, if the `mysim` namespace was declared in a containing element such as the `<sbml>` object, the annotation might look like:

```
<annotation>
    <mysim:molecule mysim:weight="18.02" mysim:atomCount="3"/>
</annotation>
```

The key point of both examples above is that application-specific annotation data are entirely contained inside a single *top-level element* within the SBML `annotation` container. SBML Level 3 Version 2 Core places the following restrictions on annotations:

- Within a given `annotation` element, there can only be one top-level element using a given namespace. An annotation element can contain multiple top-level elements but each must be in a different namespace.
- The ordering of top-level elements within a given `annotation` element is *not* significant. An application should not expect that its annotation content appears first in the `annotation` element, nor in any other particular location. Moreover, the ordering of top-level annotation elements may be changed by different applications as they read and write the same SBML file.

The use of XML namespaces in this manner is intended to improve the ability of multiple applications to place annotations on SBML model elements with reduced risks of interference or name collisions. Annotations stored by different simulation packages can therefore coexist in the same model definition. The rules governing the content of `annotation` elements are designed to enable applications to easily add, change, and remove their annotations from SBML elements while simultaneously preserving annotations inserted by other applications when mapping SBML from input to output.

As a further simplification and to improve software interoperability, applications are only required to preserve other annotations (i.e., annotations they do not recognize) when those annotations are self-contained entirely within `annotation`, complete with namespace declarations. The following is an example:

```
<annotation>
    <topLevelElement xmlns="URI">
       ... content in the namespace identified by “URI”...
    </topLevelElement>
</annotation>
```

Some more examples hopefully will make these points more clear. The following example is invalid because it contains two top-level elements using the same XML namespace, `http://www.mysim.org/ns`. Note that it does not matter that these are two different top-level elements (`<molecule>` and `<atom>`); what matters for SBML is that these separate elements are both in the same namespace rather than having been collected and placed inside one overall container element for that namespace:

```
<annotation>
   <mysim:molecule xmlns:mysim="http://www.mysim.org/ns" mysim:weight="18.02" mysim:atoms="3"/>
   <mysim:atom xmlns:mysim="http://www.mysim.org/ns" mysim:weight="18.02" mysim:atoms="3"/>
</annotation>
```

On the other hand, the following example is valid. The elements `molecule`, `bonds`, and `icon` inside the annotation each use a separate XML namespace (i.e., `http://www.mysim.org/ns`, `http://www.struct.org/ns`, and `http://othersim.com`, respectively, declared in the elements where they appear):

```
<annotation>
   <mysim:molecule xmlns:mysim="http://www.mysim.org/ns" mysim:weight="18.02" mysim:atoms="3"/>
   <struct:bonds xmlns:struct="http://www.struct.org/ns" struct:number="2" struct:type="ionic"/>
   <othersim:icon xmlns:othersim="http://www.othersim.com/">WS2002</othersim:icon>
</annotation>
```

For completeness, we note that annotations legally can be empty (but such annotations have no meaning):

```
<annotation />
```

It is worth keeping in mind that although XML namespace names must be URIs, they are (like all XML namespace names) *not required* to be directly usable in the sense of identifying an actual, retrieval document or resource on the Internet (Bray et al., 1999). URIs such as `http://www.mysim.org/` may appear as though they are (e.g.,) Internet addresses, but they are not the same thing. This style of URI strings, using a domain name and other parts, is only a simple and commonly-used way of creating a unique name string.

Finally, note that the namespaces being referred to here are XML namespaces specifically in the context of the `annotation` element on ***SBase***. The namespace issue here is unrelated to the namespaces discussed in Section 3.3.1 in the context of component identifiers in SBML.

##### *Content of annotations and implications for software tools*

**Annotation** exists as a subobject of ***SBase*** in order that software developers may attach optional application-specific data to the elements in an SBML model. However, it is important that this facility is not misused. In particular, it is *critical* that data essential to a model definition or that can be encoded in existing SBML elements is *not* stored in annotations. Parameter values, functional dependencies between model elements, etc., should not be recorded as annotations. It is crucial to keep in mind the fact that data placed in annotations can be freely ignored by software applications. If such data affect the interpretation of a model, then software interoperability is greatly impeded. Recommendations regarding the use of any sort of annotation are given in Section 8.1.4 on p. 147.

### 3.3 The `id` and `name` attributes on ***SBase***

Every object whose class is derived from ***SBase*** may have values for the `id` and `name` attributes. In this section, we elaborate on the use of `id` and `name` and discuss some of the implications.

#### *3.3.1 The `id` attribute and identifier scoping*

A model can contain a large number of components representing different parts. This leads to a problem in deciding the scope of an identifier: in what contexts does a given identifier $X$ represent the same thing? The approaches used in existing simulation software tend to fall into two categories which we may call global and local. The *global* approach places all identifiers into a single global space of identifiers, so that an identifier $X$ represents the same thing wherever it appears in a given model definition. The *local* approach places symbols in separate identifier namespaces, depending on the context, where the context may be, for example, individual reaction rate expressions. The latter approach means that a model may use the same identifier $X$ in different rate expressions and have each instance represent a different quantity.

The scoping rules in SBML Level 3 are intended as a compromise to help support both scenarios:

- The identifier (i.e., the value of the attribute `id`) of every ***SBase***-derived class that does not stipulate otherwise must be unique across the set of all such identifiers in the model. This means, for example, that a reaction and a species definition cannot both have the same identifier.

- The identifier of every **UnitDefinition** must be unique across the set of all such identifiers in the model plus the set of base unit definitions in Table 2 on p. 44. However, unit identifiers live in a separate space of identifiers from other identifiers in the model, by virtue of the fact that the data type of unit identifiers is `UnitSId` (Section 3.1.9 on p. 13) and not `SId`.

- Each **Reaction** instance (see Section 4.11 on p. 68) establishes a separate private local space for local parameters represented by objects of class **LocalParameter**. Within the definition of that reaction, local parameter identifiers override (shadow) identical identifiers from the `SId` namespace of the model outside of that reaction. Conversely, local parameters in a given **KineticLaw** object are not visible outside of that kinetic law's **Reaction**. To emphasize this, a **LocalParameter**'s `id` attribute has type `LocalSId` (Section 3.1.11 on p. 14) rather than `SId`.

- The identifier of every ***SBase***-derived class defined by an SBML Level 3 package is part of the `SId` namespace of the model by default. However, a package may declare the `id` attribute of a new class to have a type derived from `SId`, whether it is defined in SBML Level 3 Core or one defined by that Level 3 package. Elements with an `id` of that type follow the rules of uniqueness defined for the type. For example, the SBML Level 3 Hierarchical Model Composition package defines a `PortSId` type, and **Port** objects have an `id` attribute of that type. The Hierarchical Model Composition package stipulates that all **Port** element `id` values in a given **Model** must be unique only among the other **Port** objects in that **Model**.

#### 3.3.2 Motivations for defining `id` and `name` on SBase

SBML Level 3 Version 2 Core puts the `id` and `name` attributes directly on the ***SBase*** abstract class. This is a departure from previous Level/Version combinations of SBML, in which `id` and `name` were defined on individual SBML component classes. The following were the motivations for this change.

- Object classes in SBML originally were given `id` attributes only if they had meaning for mathematical expressions. (For instance, the concentrations of species, the sizes of compartments, etc.) As SBML evolved, more objects were given `id` attributes but these were not always associated with a mathematical meaning. When SBML Level 3 packages were introduced, not only did further uses of `id` become apparent: it also became possible for Level 3 packages to add nuances of meanings that Level 3 Core did not. Thus, while Level 3 Core may not define a use for an `id` attribute on a given object, a Level 3 package might. Examples exist in the SBML Level 3 Graphical Layout and Hierarchical Model Composition packages.

- The `metaid` attribute on ***SBase*** may seem to fulfill a similar role as an identifier for every object in an SBML document. However, for several reasons it is unsuitable for use as a substitute for `id`. First, owing to the fact that its data type is the XML type `ID`, it has a less restricted syntax (for instance, allowing Unicode characters). Second, its scope cannot be ammended as needed for elements such as the **UnitDefinition** or **LocalParameter**, because XML `ID` is defined to have document-wide uniqueness properties. Finally, having a *model*-wide scope instead of a *document*-wide scope has been found desirable for the SBML Level 3 Hierarchical Model Composition package.

- Because SBML Level 3 Version 2 Core objects may reference objects in SBML Level 3 packages directly, and because package objects themselves may reference Core objects as well as objects from other packages, the simplest means of achieving this is to put the `id` attribute on ***SBase*** directly.

- The `name` attribute is logically paired with `id`, to provide the option of a user-readable moniker for any object with an `id` attribute. With `id` on ***SBase***, it makes sense to put `name` on ***SBase*** too.

This design change (moving `id` and `name`) does not have an impact on the final written XML form of SBML for constructs that had `id` and `name` attributes in SBML Level 3 Version 1 (or earlier versions of SBML).

#### 3.3.3 Use of SBML Level 3 Version 1 packages and implications for identifiers

Packages created and designed for use with SBML Level 3 Version 1 may be used in SBML Level 3 Version 2 documents, and be interpreted in exactly the same manner as before. They may not, however, take full advantage of the new features and constructs in Version 2. This means the following:

- Any object class in an SBML Level 3 Version 1 package that inherits from ***SBase*** will not inherit the new `id` and `name` attributes on ***SBase***.
- An object identifier from a SBML Level 3 Version 1 package may not be used in **Math** constructs in SBML Level 3 Version 2 Core, nor as the value of any `SIdRef` type attribute in a Core construct.
- No object from a SBML Level 3 Version 1 package is considered to have mathematical meaning in core: no such object's `SId` may be used as the target of a rule nor of an assignment, nor may it be used in any MathML outside of that package.

However, if a package defines an `SIdRef` that may refer to any ***SBase***, it is legal for those references to point to SBML Level 3 Version 2 `SIds`. In particular:

- An `SIdRef` defined in a SBML Level 3 Version 1 package may be used to reference an element from Level 3 Version 2 Core that has an `SId`, but did not have one in Version 1.
- An `SIdRef` defined in a SBML Level 3 Version 1 package may be used to reference an element from an SBML Level 3 Version 2 package.
- Any **Math** object defined in a SBML Level 3 Version 1 package may reference any SBML Level 3 Version 2 package `SId` defined as having mathematical meaning.

To take some examples: the `reference` attribute of a SBML Level 3 Version 1 Layout **GeneralGlyph** is of type `SIdRef`, and was designed to be able to point to arbitrary SBML Level 3 Version 1 core and package elements. If used in a Level 3 Version 2 Core document, it could now point to an **AssignmentRule**, which it would not have been able to do in a SBML Level 3 Version 1 document. Alternatively, it could point to an SBML Level 3 Version 2 package element that inherited an `SId` from ***SBase***. Similarly, any **Math** element in an SBML Level 3 Version 1 package must be designed to be able to contain `SIdRefs` to SBML Level 3 Version 1 Core elements with mathematical meaning, but whose meaning might have been changed by a SBML Level 3 Version 1 package. These formulas may now skip the middle man and refer directly to SBML Level 3 Version 2 package `SIds` that have their own mathematical meaning.

### 3.4 Mathematical formulas in SBML Level 3

Mathematical expressions in SBML Level 3 are represented using MathML 2.0 (W3C, 2000b). MathML is an international standard for encoding mathematical expressions using XML. There are two principal facets of MathML, one for encoding content (i.e., the semantic interpretation of a mathematical expression), and another for encoding presentation or display characteristics. SBML only makes direct use of a subset of the content portion of MathML. However, it is not possible to produce a completely smooth and conflict-free interface between MathML and other standards used by SBML (in particular, XML Schema). Two specific issues and their resolutions are discussed in Section 3.4.2 on the following page.

The XML namespace URI for all MathML elements is `http://www.w3.org/1998/Math/MathML`. Everywhere MathML content is allowed in SBML, the MathML elements must be properly placed within the MathML 2.0 namespace. In XML, this can be accomplished in a number of ways, and the examples throughout this specification illustrate the use of this namespace and MathML in SBML. Please refer to the W3C document by Bray et al. (1999) for more technical information about using XML namespaces.

#### *3.4.1 Subset of MathML used in SBML Level 3 Core*

The subset of MathML elements used in SBML Level 3 Version 2 Core is listed below. An SBML Level 3 package may extend this subset (and if so, must define `required`="`true`" on the SBML container Section 4.1).

- *token*: `cn`, `ci`, `csymbol`, `sep`
- *general*: `apply`, `piecewise`, `piece`, `otherwise`, `lambda` (however, as discussed elsewhere, `lambda` is restricted to use in **FunctionDefinition**)

- *relational operators*: `eq`, `neq`, `gt`, `lt`, `geq`, `leq`
- *arithmetic operators*: `plus`, `minus`, `times`, `divide`, `power`, `root`, `abs`, `exp`, `ln`, `log`, `floor`, `ceiling`, `factorial`, `quotient`, `max`, `min`, `rem`
- *logical operators*: `and`, `or`, `xor`, `not`, `implies`
- *qualifiers*: `degree`, `bvar`, `logbase`
- *trigonometric operators*: `sin`, `cos`, `tan`, `sec`, `csc`, `cot`, `sinh`, `cosh`, `tanh`, `sech`, `csch`, `coth`, `arcsin`, `arccos`, `arctan`, `arcsec`, `arccsc`, `arccot`, `arcsinh`, `arccosh`, `arctanh`, `arcsech`, `arccsch`, `arccoth`
- *constants*: `true`, `false`, `notanumber`, `pi`, `infinity`, `exponentiale`
- *MathML annotations*: `semantics`, `annotation`, `annotation-xml`

The inclusion of logical operators, relational operators, `piecewise`, `piece`, and `otherwise` elements facilitates the encoding of discontinuous expressions.

As defined by MathML 2.0, the semantic interpretation of the mathematical functions listed above follows the definitions of the functions laid out by Abramowitz and Stegun (1977) and Zwillinger (1996). Readers are directed to these sources and the MathML specification for information for further information, such as which principal values of the inverse trigonometric functions to use.

Software authors should take particular note of the MathML semantics of the N-ary operators `plus`, `times`, `and`, `or` and `xor`, when they are used with different numbers of arguments. The MathML specification (W3C, 2000b) appendix C.2.3 describes the semantics for these operators with zero, one, and more arguments. However, while `min` and `max` are also defined as N-ary operators, MathML does not define their meaning when used with no arguments. This SBML specification also does not define the meaning of `min` and `max` with zero arguments, though a Level 3 package may add a definition. In the absence of such a package, the use of `min` or `max` with zero arguments is discouraged.

The following are the only attributes permitted on MathML elements in SBML (in addition to the `xmlns` attribute on `math` elements):

- `style`, `class` and `id` on any element;
- `encoding` on `csymbol`, `annotation` and `annotation-xml` elements;
- `definitionURL` on `ci`, `csymbol` and `semantics` elements; and
- `type` and `sbml:units` (see Section 3.4.2) on `cn` elements.

Missing values for the MathML attributes are to be treated in the same way as defined by MathML 2.0. These restrictions on attributes are designed to confine the MathML elements to their default semantics and to avoid conflicts in the interpretation of the type of token elements.

#### 3.4.2 Numbers and `cn` elements

In MathML, literal numbers are written as the content portion of a particular element called `cn`. This element takes an optional attribute, `type`, used to indicate the *type* of the number (such as whether it is meant to be an integer or a floating-point quantity). Here is an example of its use:

```
<math xmlns="http://www.w3.org/1998/Math/MathML">
    <apply>
        <times/> <cn type="integer"> 42 </cn> <cn type="real"> 3.3 </cn>
    </apply>
</math>
```

The content of a `cn` element must be a number. The number can be preceded and succeeded by whitespace (see Section 3.4.5 on p. 24). The following are the only permissible values for the `type` attribute on MathML `cn` elements: "`e-notation`", "`real`", "`integer`", and "`rational`". The value of the `type` attribute defaults to "`real`" if it is not specified on a given `cn` element.

##### Value space restrictions on `cn` content

SBML imposes certain restrictions on the value space of numbers allowed in MathML expressions. According to the MathML 2.0 specification, the values of the content of `cn` elements do not necessarily have to conform to any specific floating-point or integer representations designed for CPU implementation. For example, in strict MathML, the value of a **cn** element could exceed the maximum value that can be stored in an IEEE 64 bit floating-point number (IEEE 754). This is different from the XML Schema type **double** that is used in the definition of floating-point attributes of objects in SBML; the XML Schema **double** *is* restricted to IEEE double-precision 64-bit floating-point type IEEE 754-1985. To avoid an inconsistency that would result between numbers elsewhere in SBML and numbers in MathML expressions, SBML Level 3 Version 2 Core imposes the following restriction on MathML content appearing in SBML:

- Integer values (i.e., the values of **cn** elements having **type**="**integer**" and both values in **cn** elements having **type**="**rational**") must conform to the **int** type (Section 3.1.3 on p. 11)
- Floating-point values (i.e., the content of **cn** elements having **type**="**real**" or **type**="**e-notation**") must conform to the **double** type (Section 3.1.5 on p. 11)

##### *Syntactic differences in the representation of numbers in scientific notation*

It is important to note that MathML uses a style of scientific notation that differs from what is defined in XML Schema, and consequently what is used in SBML attribute values. The MathML 2.0 type "**e-notation**" (as well as the type "**rational**") requires the mantissa and exponent to be separated by one **<sep/>** element. The mantissa must be a real number and the exponent part must be a signed integer. This leads to expressions such as

```
<cn type="e-notation"> 2 <sep/> -5 </cn>
```

for the number $2 \cdot 10^{-5}$. It is especially important to note that the following expression,

```
<cn type="e-notation"> 2e-5 </cn>
```

is *not valid* in MathML 2.0 and therefore cannot be used in MathML content in SBML. However, elsewhere in SBML, when an attribute value is declared to have the data type **double** (a type taken from XML Schema), the compact notation "**2e-5**" is in fact allowed. In other words, within MathML expressions contained in SBML (and *only* within such MathML expressions), numbers in scientific notation must take the form **<cn type="e-notation"> 2 <sep/> -5 </cn>**, and everywhere else they must take the form "**2e-5**" or "**2E-5**".

This is a regrettable difference between two standards that SBML replies upon, but it is not feasible to redefine these types within SBML because the result would be incompatible with parser libraries written to conform to the MathML and XML Schema standards. It is also not possible to use XML Schema to define a data type for SBML attribute values permitting the use of the **<sep/>** notation, because XML attribute values cannot contain XML elements—that is, **<sep/>** cannot appear in an XML attribute value.

##### *Units associated with numbers in MathML* cn *expressions*

What units should be attributed to numbers appearing inside MathML **cn** elements? One answer is to assume that the units should be "whatever units are appropriate in the context where the number appears". This implies that units can always be assigned unambiguously to any number by inspecting the expression in which it appears, and this turns out to be false. Another answer is that numbers should be considered "dimensionless". Many people argue that this is the correct interpretation, but even if it is, there is an overriding practical reason why it cannot be adopted for SBML's domain of application: when numbers appear in expressions in SBML, they are *rarely intended* by the modeler to have the unit "**dimensionless**" even if the unit is not declared—instead, the numbers are *supposed* to have specific units, but the units are usually undeclared. (Being "dimensionless" is not the same as having *undeclared* units!) If SBML defined numbers as being *by default* dimensionless, it would result in many models being technically incorrect without the modeler being aware of it unless their software tools performed dimensional analysis. Many software tools do not perform unit analysis, and so potential errors due to inconsistent units in a model would not be detected until other researchers and database curators attempted to use the model in software packages that *did* check units. We believe the negative impact on interoperability would be too high.

SBML borrows an idea from CellML (Hedley et al., 2001), another model definition language with goals similar to SBML's, and allows an additional attribute to appear on MathML **cn** elements; the value of this attribute can be used to indicate the unit of measurement to be associated with the number in the content of the cn element. The attribute is named units but, because it appears inside MathML element (which is in the XML namespace for MathML and not the namespace for SBML), it must always be prefixed with an XML namespace prefix for the SBML Level 3 Version 2 Core namespace. The value of the attribute must have the data type UnitSIdRef (Section 3.1.10 on p. 13) and can be the identifier of a **UnitDefinition** object in the model or a base unit listed in Table 2 on p. 44. The following example illustrates how this attribute can be used to define a number with value "10" and unit of measurement "second":

```
<math xmlns="http://www.w3.org/1998/Math/MathML"
      xmlns:sbml="http://www.sbml.org/sbml/level3/version2/core">
    <cn type="integer" sbml:units="second"> 10 </cn>
</math>
```

In this example, we chose to use the string "sbml" as the XML namespace prefix for the SBML Level 3 Version 2 Core namespace, which leads to the use of sbml:units as the attribute on the cn element. We could have used another prefix string besides "sbml", and the definition of the prefix could also have appeared on a higher-level element in the model. Section 4.1 on p. 34 provides more information about the XML namespace for SBML Level 3 Version 2 Core.

An alternative approach to specifying units is to avoid using cn elements altogether, and always use ci elements to reference **Parameter** objects having both value and units defined. In the example above, we could have avoided putting the literal number "10" inside the mathematical expression, and instead, defined a parameter in the model, given it the value "10" and unit "second", and finally, referred to that parameter in the math content above. The approach of using named parameters provides additional power and advantages over simply using sbml:units attributes on cn elements; for example, **Parameter** allows the association of terms from the Systems Biology Ontology (SBO; Section 5) as well as MIRIAM annotations (Section 6).

In summary, a literal number within MathML content without an SBML units attribute has no declared unit associated with it. Either of the approaches described above (i.e., avoiding cn in favor of ci elements and **Parameter** objects, or using an sbml:units attribute on cn) leads to formulas whose units can be fully determined, enabling software tools to perform dimensional analysis and, potentially, detect and report problems with the model. Conversely, in the absence of an SBML units attribute on a MathML cn element, no unit is associated with the number within the cn element. If the example above lacked the attribute sbml:units, the value "10" would have no declared unit associated with it.

Finally, although SBML provides ways of associating units with numbers and entities, SBML does not stipulate that implicit unit conversions be performed. Section 3.4.13 explores this topic in more detail.

#### 3.4.3 Use of ci elements in MathML expressions in SBML

The content of a ci element must be an SBML identifier that is declared elsewhere in the model. The identifier can be preceded and succeeded by whitespace within the ci. The set of possible identifiers that can appear in a ci element depends on the containing element in which the ci is used:

- If a ci element appears in the math body of a **FunctionDefinition** object (Section 4.3 on p. 40), the referenced identifier must be either (i) one of the declared arguments to that function, or (ii) the identifier of another **FunctionDefinition** object in the model.
- Otherwise, the identifier referenced by the ci element must belong to an SBML object that (a) is in the SId namespace of the **Model**, (b) has mathematical meaning in SBML, and (c) is within the relevant scope. Table 1 on the next page lists the only possible such identifiers in SBML Level 3 Version 2 Core.

The content of ci elements in MathML formulas outside of a **KineticLaw** or **FunctionDefinition** must always refer to objects declared in the top-level global namespace; i.e., SBML uses *early binding* semantics. Inside of **KineticLaw**, ci elements can additionally refer to identifiers of **LocalParameter** objects defined within that **KineticLaw** instance; see Section 4.11.5 on p. 74 for more information.

If a MathML ci element references an identifier in an SBML namespace that is not recognized by the interpreter reading a given SBML document (that is, if the referent object is defined by an SBML Level 3

*Table 1: The possible interpretations of different SBML component identifiers when they appear in MathML `ci` elements outside the body of a **FunctionDefinition** object. (Inside a **FunctionDefinition** object's mathematical formula, different rules apply, as described in Section 3.4.3 on the previous page.)*

| Identifier kind | Interpretation | Units |
|---|---|---|
| **FunctionDefinition** | a call to the function (using a MathML `apply` element) | See Section 4.3.4 on p. 41 |
| **Compartment** | the size of the compartment | See Section 4.5.4 on p. 48 |
| **Species** | the quantity of the species, which may be either an *amount of substance* or a *concentration*, depending on the value of the **Species** object's attribute `hasOnlySubstanceUnits` | See Section 4.6.5 on p. 51 |
| **Parameter** | the value of the parameter | See Section 4.7.3 on p. 55 |
| **Reaction** | the rate of the reaction | See Section 4.11.7 on p. 78 |
| **SpeciesReference** | the stoichiometry of the indicated reactant or product in the reaction where the **SpeciesReference** object is defined | See Section 4.11.3 on p. 72 |
| **LocalParameter** | the value of the local parameter within the reaction where the **LocalParameter** object is defined | See Section 4.11.6 on p. 75 |
| Object class defined in a Level 3 package | the value defined for that object by the package | See that package's specification |

package not supported by that software), the `math` element in which it appears no longer has a mathematical interpretation and may be ignored by the interpreter. If the interpreter cannot establish whether a referenced object is entirely missing from the model or is merely defined in an SBML namespace not recognized by the interpreter, it may produce a warning to the user. (The latter situation may arise if an SBML package is present in the SBML document with a `package:required` attribute of "`true`".)

#### 3.4.4 Interpretation of `boolean` values

As noted already in Section 3.1.2 on p. 11, there is another unfortunate difference between the XML Schema 1.0 and MathML 2.0 standards that impacts mathematical expressions in SBML: in XML Schema, the value space of type `boolean` includes "`true`", "`false`", "`1`", and "`0`", whereas in MathML, only "`true`" and "`false`" count as Boolean values.

The impact of this difference is, thankfully, minimal because the XML Schema definition is only used for attribute values on SBML objects, and those values turn out never to be accessible from MathML content in SBML—values of `boolean` attributes on SBML objects can never enter into MathML expressions. Nevertheless, software authors and users should be aware of the difference.

SBML also allows models to use Boolean values in numerical contexts, and visa versa. In numerical contexts, the Boolean value `true` becomes the numerical value `1`, and the Boolean value `false` becomes the value `0`. The unit of measurement of `0` and `1` in such cases is considered to be `dimensionless`. In Boolean contexts, the numerical value `0` becomes the Boolean value `false`, and all other numerical values (positive and negative) become `true`. Converted numerical quantities (e.g., parameters) should have the unit `dimensionless` *before* conversion. Note that after conversions between Boolean and numerical values are performed, strict type checking may no longer be possible in some contexts, but the tradeoff is greater flexibility and simplicity for modelers.

#### 3.4.5 Handling of whitespace

MathML 2.0 defines "whitespace" in the same way as XML does, i.e., the space character (Unicode hexadecimal code 0020), horizontal tab (code 0009), newline or line feed (code 000A), and carriage return (code 000D). In MathML, the content of elements such as `cn` and `ci` can be surrounded by whitespace characters. Prior to using the content, this whitespace is “trimmed” from both ends: all whitespace at the beginning and end of the content is removed (Ausbrooks et al., 2003). For example, in `<cn> 42 </cn>`, the amount of white space on either side of the “42” inside the `<cn>` ... `</cn>` container does not matter. Prior to interpreting the content, the whitespace is removed altogether.

#### 3.4.6 Use of `csymbol` elements in MathML expressions in SBML

SBML Level 3 uses the MathML `csymbol` element to denote certain built-in mathematical entities without introducing reserved names into the component identifier namespace. The `encoding` attribute of `csymbol` must be set to “`text`”. The `definitionURL` should be set to one of the following URIs defined by SBML:

- `http://www.sbml.org/sbml/symbols/time`. This represents the current simulation time. See Section 3.4.7 on p. 27 for more information. The unit of measurement associated with time is determined by the value of the attribute `timeUnits` on **Model**.
- `http://www.sbml.org/sbml/symbols/delay`. This represents a delay function. The delay function has the form $delay(x, d)$, taking two MathML expressions as arguments. The function's value is the value of argument $x$, but taken at a time $d$ before the current time. There are no restrictions on the form of $x$. Since the parameter $d$ represents a time value, the unit of measurement associated with $d$ is expected to match the unit of time in the model as specified by the value of the **Model** attribute `timeUnits`. The value of the $d$ parameter, when evaluated, must be numerical (i.e., a number in MathML real, integer, rational, or “e-notation” format) and be greater than or equal to 0. The unit of measurement associated with the return value of the delay function is identical to that of the parameter $x$. See Section 3.4.7 on p. 27 below for additional considerations surrounding the use of this `csymbol`.
- `http://www.sbml.org/sbml/symbols/avogadro`. This represents the numerical value of Avogadro's constant. The value of Avogadro's constant is determined experimentally; for the purposes of SBML Level 3 Version 2, the numerical value is taken to be the one recommended by the 2006 edition of CODATA (Mohr et al., 2008), but the unit of the value is `dimensionless`. In other words, the value of this `csymbol` is equivalent to the following:

$$(6.02214179 \cdot 10^{23}) \cdot \texttt{dimensionless}$$

If the value of the constant is revised by international standards-setting organizations in the future, a future Version of the SBML Level 3 specification may stipulate a new value to be used for this `csymbol` constant. However, all software applications reading models expressed in *this* Version of SBML Level 3 should *always* use the value of Avogadro's constant given above. (In other words, changes will apply only beginning with a possible new Version of SBML Level 3 and not this existing version.)
- `http://www.sbml.org/sbml/symbols/rateOf`. This represents the instantaneous rate of change, with respect to time, of an entity in the model. It is a function that takes a single argument, an identifier of type `SId`. The allowable identifiers for use with *rateOf* in SBML Level 3 Version 2 Core are restricted to those of **Compartment**, **LocalParameter**, **Parameter**, **Species**, and **SpeciesReference** objects in the enclosing model; in addition, SBML Level 3 packages may define entities with mathematical meaning whose *rateOf*'s can be referenced. Note that *rateOf* is not allowed for **Reaction** objects, because their identifiers already represent the rate of change of the reaction, and calculating second derivatives is beyond the scope of this construct. Likewise, there is no sensible meaning to be given to the *rateOf* of a **FunctionDefinition**, **Event**, **Priority**, **Delay**, or other SBML entities.

The intent of this `csymbol` is to provide a means for models to refer to quantities that must naturally be computed as part of doing a dynamical analysis of a model. The *rateOf* `csymbol` is not intended to provide full numerical differentiation capabilities. The value of *rateOf* function for a symbol can be determined more straightforwardly as follows:

1. The *rateOf* for any symbol declared constant is zero. Note that in SBML Level 3 Version 2 Core, **LocalParameter** objects must always be constant; therefore, their *rateOf* value is always zero.
  2. The *rateOf* for a symbol whose `SId` appears as the `variable` of a **RateRule** is the numerical value of that **RateRule**, using the current values of all symbols referenced in the rule's formula.

3. The *rateOf* for the *amount* of a **Species** having attribute `boundaryCondition`="`false`" and appearing in one or more reactions can be calculated from the stoichiometries and **KineticLaw** **Math** of every **Reaction** in which the species appears, plus appropriate `conversionFactor` values (see Section 4.11.7 on p. 77). If the *species quantity* is in terms of its *concentration*, the rate must be converted by the `size` of the **Compartment** in which it appears, which may itself be changing in time. This can be calculated as follows, where $[x]$ is the concentration of species X, $x$ the amount of species X, and $V$ the size of the compartment in which species X is located:

$$\begin{aligned}\frac{d[x]}{dt} &= \frac{d(x/V)}{dt} \\ &= \frac{1}{V}\cdot\frac{dx}{dt} + x\cdot\frac{d(1/V)}{dt} \\ &= \frac{1}{V}\cdot\frac{dx}{dt} - \frac{x}{V^2}\cdot\frac{dV}{dt}\end{aligned}$$

When $dV/dt$ is equal to zero, the final term in the last line is zero.

4. The *rateOf* value for any valid target in SBML for time $t < 0$ will always be zero. This is because the *rateOf* `csymbol` may only point to SBML elements that change due to a **RateRule** or a **Reaction**, and neither of those constructs apply at $t < 0$.

An object whose `SId` identifier appears as the `variable` of an **AssignmentRule**, or which is calculated from an **AlgebraicRule**, may not be referenced by the *rateOf* `csymbol`. Similarly, it is also not valid to use the *rateOf* `csymbol` to reference a **Species** with a `hasOnlySubstanceUnits` attribute value of "`false`" and whose `compartment` appears as the `variable` of an **AssignmentRule** or whose `size` is calculated from an **AlgebraicRule**. In other words, anything whose value is directly or indirectly determined by an algebraic rule or an assignment rule is excluded.

In the event of a discontinuity, such as might happen due to an **Event**, a `piecewise` function, the beginning of a time course simulation (i.e., at $t = 0$), or due to a new construct defined in a package, the rate of change is defined as the right-handed *rateOf* for the symbol, that is, the derivative with respect to time of the symbol moving forward in time from the current time, and not the derivative with respect to time from the recent past up until the current time. Thus, the *rateOf* of a symbol will always be calculable from the set of current values of symbols in the model. No **Event** can affect the *rateOf* for a symbol except indirectly.

In simulations that progress in a stepwise fashion, such as stochastic simulations, the *rateOf* `csymbol` is still calculated as above, from any appropriate **RateRule** or **KineticLaw**. This effectively means that for stepwise simulations, the *rateOf* indicates the expected average rate of change of the corresponding symbol over time, even when the actual rate of change may be zero or discontinuous.

The following examples demonstrate these concepts. The XML fragment below encodes the formula $x + t$, where $t$ stands for time.

```
<math xmlns="http://www.w3.org/1998/Math/MathML">
    <apply>
        <plus/>
        <ci> x </ci>
        <csymbol encoding="text" definitionURL="http://www.sbml.org/sbml/symbols/time">
            t
        </csymbol>
    </apply>
</math>
```

In the fragment above, the use of the token `t` is mostly a convenience for human readers—the string inside the `csymbol` could have been almost anything, because it is essentially ignored by MathML parsers and SBML. It can even be empty. Some MathML and SBML processors will take note of the token and use it when presenting the mathematical formula to users, but the token used has no impact on the interpretation of the model and it does *not* enter into the SBML component identifier namespace. In other words, the SBML model cannot refer to `t` in the example above. The content of the `csymbol` element is for rendering purposes only and can be ignored by the parser.

As a further example, the following XML fragment encodes the equation $k + delay(x, 0.1)$ or, alternatively, $k_t + x_{t-0.1}$:

```
<math xmlns="http://www.w3.org/1998/Math/MathML"
      xmlns:sbml="http://www.sbml.org/sbml/level3/version2/core">
    <apply>
        <plus/>
        <ci> k </ci>
        <apply>
            <csymbol encoding="text" definitionURL="http://www.sbml.org/sbml/symbols/delay" />
            <ci> x </ci>
            <cn sbml:units="second"> 0.1 </cn>
        </apply>
    </apply>
</math>
```

The use of Avogadro's number is illustrated in the following XML fragment, which encodes the formula $avogadro * x$:

```
<math xmlns="http://www.w3.org/1998/Math/MathML">
    <apply>
        <times/>
        <csymbol encoding="text" definitionURL="http://www.sbml.org/sbml/symbols/avogadro" />
        <ci> x </ci>
    </apply>
</math>
```

Finally, the use of a *rateOf* function call is illustrated in the following XML fragment, and encodes the formula $k + rateOf(S1)$:

```
<math xmlns="http://www.w3.org/1998/Math/MathML">
  <apply>
    <plus/>
    <ci> k </ci>
    <apply>
      <csymbol encoding="text" definitionURL="http://www.sbml.org/sbml/symbols/rateOf"/>
      <ci> S1 </ci>
    </apply>
  </apply>
</math>
```

#### 3.4.7 Simulation time

The principal use of SBML is to represent quantitative dynamical models whose behaviors manifest over time. In defining an SBML model using constructs such as reactions, time is most often implicit and does not need to be referred to in the mathematical expressions themselves. However, sometimes an explicit time dependency needs to be stated, and for this purpose, the *time* `csymbol` (described above in Section 3.4.6) may be used. This *time* symbol refers to "instantaneous current time" in a simulation, frequently given the literal name $t$ in one's equations.

An assumption in SBML is that "start time" or "initial time" in a simulation is zero, that is, if $t_0$ is the initial time in the system, $t_0 = 0$. This corresponds to the most common scenario. Initial conditions in SBML take effect at time $t = 0$. There is no mechanism in SBML for setting the initial time to a value other than 0. To refer to a different time in a model, one approach is to define a **Parameter** for a new time variable and use an **AssignmentRule** in which the assignment expression subtracts a value from the `csymbol` *time*. For example, if the desired offset is 2 seconds, the MathML expression would be

```
<math xmlns="http://www.w3.org/1998/Math/MathML"
      xmlns:sbml="http://www.sbml.org/sbml/level3/version2/core">
    <apply>
        <minus/>
        <csymbol encoding="text" definitionURL="http://www.sbml.org/sbml/symbols/time"/>
        <cn sbml:units="second"> 2 </cn>
    </apply>
</math>
```

SBML's assignment rules (Section 4.9.3) can be used to express mathematical statements that hold true at all moments, so using an assignment rule with the expression above will result in the value being equal to $t-2$ at every point in time. A parameter assigned this value could then be used elsewhere in the model.

#### 3.4.8 Initial conditions and special considerations

The identifiers of **Species**, **SpeciesReference**, **Compartment**, **Parameter**, and **Reaction** object instances in a given SBML model refer to the main symbols in a model. Depending on certain attributes of these objects (e.g., the attribute `constant` on species, species references, compartments and parameters—this and other conditions are explained in the relevant sections elsewhere in this document), some of the symbols may have constant values throughout a simulation, and others' values may change. These changes in values over time are determined by the system of equations constructed from the model's reactions, initial assignments, rules, and events.

As described in Section 3.4.7, an SBML model's simulation is assumed to begin at $t = 0$. The availability of the *delay* `csymbol` (Section 3.4.6 on p. 25) introduces the possibility that at $t \geq 0$, mathematical expressions in a model may draw on values of model components from time *prior* to $t = 0$. A simulator may therefore need to compute the values of symbols at time points $t_i \leq 0$ to allow the calculation of values required for the evaluation of delay expressions in the model for $t \geq 0$. If there are no delays in the model, then $t_i = 0$.

The following is how the definitions of the model should be applied:

1. At time $t_i$:
   - Every **Species**, **SpeciesReference**, **Compartment**, **Parameter**, and package element with mathematical meaning whose definition includes an initial value is assigned that value. If an element has `constant`="`false`", its value may be changed by other constructs or reactions in a model according to the steps below; if `constant`="`true`", only an **InitialAssignment** can override the value.
   - All **InitialAssignment** definitions take effect, overriding any initial values on any **Species**, **SpeciesReference**, **Compartment**, **Parameter**, or package element with mathematical meaning.
   - All **AssignmentRule** and **AlgebraicRule** definitions take effect. These rules also override any initial values of any **Species**, **SpeciesReference**, **Compartment**, **Parameter**, or package element with mathematical meaning. Only elements set `constant`="`false`" can be affected in this way. (Note there cannot be both an **AssignmentRule** and an **InitialAssignment** for the same identifier, nor may an **AlgebraicRule** determine the value of any element that has an **InitialAssignment**; see Section 4.9.)
   - The identifier of any **Reaction** has the value of its **KineticLaw**. This cannot yet affect the **Species** referenced by the **Reaction**, but the identifier may appear in other **Math** elements calculated above.
   - The value of any **Event Trigger** is the value of that **Trigger**'s `initialValue` attribute. This cannot be overridden.

2. For time $t_i < t < 0$
   - Any element with mathematical meaning with no **InitialAssignment** or ***Rule*** that targets it continues to have its initial value, as defined by the relevant attribute.
   - Any **InitialAssignment** definition continues to take effect. Since these contain mathematical formulas, different values may be computed at each time step $t$ in $t_i \leq t \leq 0$.
   - Any **AssignmentRule** or **AlgebraicRule** definition continues to take effect, and may not be overridden. Again, different values may be computed at each time step $t$ in $t_i \leq t \leq 0$.
   - The identifier of any **Reaction** continues to be the value of its **KineticLaw**. Again, different values may be computed at each time step $t$ in $t_i \leq t \leq 0$.
   - The value of any **Event Trigger** continues to be the value of that **Trigger**'s `initialValue` attribute.

3. At time $t = 0$:
   - The value of any **Event Trigger** is now calculated according to the **Trigger**'s **Math** child, and may therefore cause the **Event** to *trigger* and its **EventAssignment** children to *execute*. This can happen directly due to its value changing from an `initialValue` of "`false`" to a now-calculated value of "`true`"; it can happen indirectly due to an event cascade initated by a direct change in a different **Event**. (Note that an **Event** cannot be defined to change the value of a symbol that is also the subject of an **AssignmentRule**, nor can it change the value of a symbol whose value is determined by an **AlgebraicRule**; see Section 4.12 on p. 79.)

- The identifier of any **Reaction** continues to be the value of its **KineticLaw**.
- Any element with mathematical meaning with no **InitialAssignment** or ***Rule*** that targets it continues to have its initial value, as defined by the relevant attribute, but may now be overridden by any **EventAssignment**, executed as above.
- Any **InitialAssignment** definition continues to take effect, but may now be overridden by any **EventAssignment**, executed as above.
- Any **AssignmentRule** or **AlgebraicRule** definition continues to take effect, and may not be overridden.
- **Constraint** definitions begin to take effect (and a constraint violation may result; see Section 4.10).

4. For time $t > 0$:
   - The value of any element with mathematical meaning may now be overridden (subject to normal restrictions) by any construct in SBML (though it may retain its original value if no such constructs apply).
   - The value of any element with an **InitialAssignment** may also now be overridden by any construct in SBML (though it may retain the value set by the **InitialAssignment** if no such constructs apply).
   - Any **AssignmentRule** or **AlgebraicRule** definition continues to take effect, and still may not be overridden by any other SBML construct.
   - **RateRule** definitions can begin to take effect.
   - Any **Reaction** can begin to affect its referenced **Species**. Its identifier continues to have the value of its **KineticLaw**.
   - Each **Event** may *fire*, and their **EventAssignment** children *execute*.
   - System simulation proceeds.

To reiterate: in modeling situations that do not involve the use of the *delay* `csymbol`, then $t_i$ becomes $t_i = 0$, but this does not alter the steps numbers 1–4 above.

##### 3.4.9 Underdetermined models

A valid SBML model must not be *overdetermined*: the value of any symbol must not be established by more than one construct in the model. The rules governing SBML constructs such as **InitialAssignment** and ***Rule*** are designed to prevent the creation of overdetermined models because such models are self-contradictory.

The opposite situation, in which a model is *under*determined, is not invalid. An SBML model may contain one or more symbols whose values are not established by the model directly, as when a **Parameter** has no `initialValue` attribute and is not the target of an **InitialAssignment** or a relevant ***Rule*** object; a model may also have multiple solutions, such as when an **AlgebraicRule** object determines either one—but not both—of two different symbols in the model, or when an **AlgebraicRule** object has multiple solutions (such as $0 = x^2 - 4$). Such models cannot be simulated without additional information, but although they are *incomplete* models, they are not *contradictory*, and therefore not invalid.

Underdetermined models may arise for various reasons. For example, a model may be created to reflect the current state of knowledge about a biological system, and that knowledge may itself be incomplete. In other cases, a model may be a work in progress, or a part of an automated model-creation pipeline. In still other situations, a model may be intended for non-numerical simulation such as structural analysis, where having numerical values for all symbols, or mathematical formulas establishing the rates of reactions, may not be required. Finally, it is also possible that the (apparently) missing information (from the perspective of an SBML Level 3 Core model) is actually provided by an SBML Level 3 package. Package information may provide the missing information needed to resolve the system, or provide a new context for the model indicating the type(s) of analyses for which the model was designed. In all these scenarios, practical exigencies demand that these SBML Level 3 Core models be considered valid even if they are incomplete (as long as the parts that *are* present are not overdetermined or invalid for other reasons!).

SBML Level 3 Version 2 Core does not stipulate a particular course of action for handling underdetermined models; software systems may handle them as they see fit. For example, numerical simulation systems could reasonably refuse to process such models (and inform the user why); other types of software may find it more appropriate to take other actions, such as asking the user to fill in the missing information.

#### 3.4.10 Use and interpretation of the 'constant' attribute

All SBML elements with mathematical meaning whose value can be assigned via a ***Rule*** have a `constant` attribute that can be "`true`" or "`false`". One purpose of this attribute is to help model validation: if an element that is meant to be constant appears as the target of (e.g.) a **RateRule** or an **AssignmentRule**, the **Model** has an internal inconsistency that must be corrected before it can be interpreted properly.

Another use of the `constant` attribute is to provide information crucial to constructing systems of equations for models that include **AlgebraicRule**. To produce a unique solution, the number of symbols appearing in **AlgebraicRule** `math` expressions must not exceed the number of **AlgebraicRule** objects. When more symbols appear in a model's set of **AlgebraicRule** `math` expressions than there are **AlgebraicRule** objects to establish their values, the model is underdetermined, and there will not be a single solution to the overall system of equations derived from the model. However, flagging one or more of the symbols as constant sets those symbols' values; when done correctly, it can lead to an equal number of **AlgebraicRule** objects as there are variables that may be determined by those rules. Consequently, the system can have a unique solution again. (See Section 4.9.2 on p. 61 for more details about **AlgebraicRule** and additional requirements.)

The `constant` attribute is also often used by software tools to determine how an element should be displayed to the user. For example, an element marked `constant`="`true`" might be listed in a table along with its (known, fixed) value, while an element marked `constant`="`false`" might be instead be displayed in a graph with its value plotted over the time course of a simulation. Moreover, it may be possible for software to implement more efficient internal handling of constant symbols.

#### 3.4.11 MathML expression data types

MathML operators in SBML return results in one of two possible types: Boolean and numerical. By numerical type, we mean either (1) a number in MathML real, integer, rational, or "e-notation" format; or (2) the `csymbol` for *time*, the `csymbol` for the *delay* function, or the `csymbol` for the *rateOf* function described in Section 3.4.6 on p. 25. However, a Boolean value may be used in a numerical context, and visa versa, as described in Section 3.4.4 on p. 24. It is still important to understand which contexts are considered Boolean and which are considered numeric, so the following guidelines summarize the different possible cases.

The relational operators (`eq`, `neq`, `gt`, `lt`, `geq`, `leq`), the logical operators (`and`, `or`, `xor`, `not`, `implies`), and the Boolean constants (`false`, `true`) always return Boolean values, or `0` and `1` in numerical contexts.

The type of an operator referring to a **FunctionDefinition** is determined by the type of the top-level operator of the expression in the `math` element of the **FunctionDefinition** instance, and can be Boolean or numerical.

All other operators, values and symbols return numerical results.

The roots of the expression trees used in the following contexts will be interpreted as Boolean values:

- the arguments of the MathML logical operators (`and`, `or`, `xor`, `not`, `implies`);
- the second argument of a MathML `piece` operator;
- the `trigger` element of an SBML **Event**; and
- the `math` element of an SBML **Constraint**.

The roots of the expression trees used in the following contexts can yield Boolean or numerical values:

- the arguments to the `eq` and `neq` operators;

- the first arguments of MathML **piece** and **otherwise** operators; and
- the top-level expression of a function definition.

The roots of expression trees in other contexts will be interpreted as numerical values.

The type of expressions should be used consistently. The set of expressions that make up the first arguments of the **piece** and **otherwise** operators within the same **piecewise** operator should all return values of the same type. The arguments of the **eq** and **neq** operators should return the same type.

#### 3.4.12 Consistency of units in mathematical expressions and treatment of unspecified units

Strictly speaking, physical validity of mathematical formulas requires not only that physical quantities added to or equated with each other have the same fundamental dimensions and units of measurement; it also requires that the application of operators and functions to quantities produces sensible results. Yet, in real-life models today, these conditions are often and sometimes legitimately disobeyed.

In a public vote held in late 2007, the SBML community decided to revoke the requirement (present up through Level 2 Version 3) for strict unit consistency in SBML. As a result, SBML Level 3 follows this decision; the units on quantities and the results of mathematical formulas in a model *should* be consistent, but it is not a strict error of SBML model representation if they are not. The following are thus formulated as recommendations that *should* be followed except in special circumstances.

##### *Recommendations for unit consistency of mathematical expressions*

The consistency of units is defined in terms of dimensional analysis applied recursively to every operator and function and every argument to them. The following conditions should hold true in a model (and software developers may wish to consider having their software warn users if one or more of the following conditions is not true):

1. All arguments to the following operators should have the same units (regardless of what those units happen to be): **plus**, **minus**, **eq**, **neq** **gt**, **lt**, **geq**, **leq**, **max**, **min**. If one or more arguments are Boolean and one or more numeric, all the numeric arguments should have units of "**dimensionless**".

2. The units associated with each argument in a call to a **FunctionDefinition** should match the units expected by the **lambda** expression within the **math** expression of that **FunctionDefinition** instance.

3. All of the possible return values from **piece** and **otherwise** subelements of a **piecewise** expression should have the same units, regardless of what they are. (Without this guideline, the **piecewise** expression would return values having different units depending on which case evaluated to true.) If one or more return values are Boolean and one or more numeric, all the numeric arguments should have units of "**dimensionless**".

4. For the *delay* **csymbol** (Section 3.4.6 on p. 25) function, which has the form $delay(x, d)$, the second argument $d$ should match the model's unit of *time* (as determined by the **Model** object's "**timeUnits**" attribute).

5. The units of the value returned by the *delay* **csymbol** (Section 3.4.6 on p. 25) function should match the units associated with the first argument $x$.

6. The units of the value returned by the *rateOf* **csymbol** (Section 3.4.6 on p. 25) function should be units appropriate for a rate of change. The units of $rateOf(x)$ should be equal to $\{$unit of $x\}/\{$unit of *time*$\}$, where the unit of *time* is determined by the value of the attribute **timeUnits** on the enclosing **Model** object. The kinds of identifiers permitted for use as $x$ are explained in Section 3.4.6 on p. 25.

7. The units of each argument to the following operators should be "**dimensionless**" or be Boolean: **exp**, **ln**, **log**, **factorial**, **sin**, **cos**, **tan**, **sec**, **csc**, **cot**, **sinh**, **cosh**, **tanh**, **sech**, **csch**, **coth**, **arcsin**, **arccos**, **arctan**, **arcsec**, **arccsc**, **arccot**, **arcsinh**, **arccosh**, **arctanh**, **arcsech**, **arccsch**, **arccoth**.

8. Numerical values used in Boolean contexts should have units of "**dimensionless**". That is, all the arguments to the MathML logical operators **and**, **or**, **xor**, **not**, and **implies**; the second argument of a

MathML `piece` operator; the `trigger` element of an SBML **Event**; and the `math` element of an SBML **Constraint**, should either be Boolean or have units of `dimensionless`.

9. The two arguments to `power`, which are of the form $power(a, b)$ with the meaning $a^b$, should be as follows: (1) if the second argument is an integer, then the first argument can have any unit; (2) if the second argument $b$ is a rational number $n/m$, it should be possible to derive the $m$-th root of $(a\{\text{unit}\})^n$, where $\{\text{unit}\}$ signifies the unit associated with $a$; otherwise, (3) the unit of the first argument should be "`dimensionless`". The second argument ($b$) should always have the unit of "`dimensionless`".

10. The two arguments to `root`, which are of the form $root(n, a)$ with the meaning $\sqrt[n]{a}$ and where the degree $n$ is optional (defaulting to "2"), should be as follows: (1) if the optional degree qualifier $n$ is an integer, then it should be possible to derive the $n$-th root of $a$; (2) if the optional degree qualifier $n$ is a rational $n/m$ then it should be possible to derive the $n$-th root of $(a\{\text{unit}\})^m$, where $\{\text{unit}\}$ signifies the unit associated with $a$; otherwise, (3) the unit of $a$ should be "`dimensionless`".

11. Where the units of literal numbers have not been specified directly in SBML, it is possible for the unit of a **FunctionDefinition** object's return value to be effectively different in different contexts where it is called (see below). If a **FunctionDefinition**'s mathematical formula contains literal constants (i.e., numbers within MathML `cn` elements with no `sbml:units` attribute), the units of the constants should be identical in all contexts the function is called.

The units of other operators such as `abs`, `floor`, and `ceiling`, can be anything.

Item number 9 above, regarding **FunctionDefinition**, merits additional elaboration. An example may help illustrate the problem. Suppose the formula $x + 5$ is defined as a function, where $x$ is an argument and the literal number 5 has no specified unit. If this function is called with an argument whose unit of measurement is `mole`, the only possible consistent unit for the return value is `mole`. If in another context in the same model, the function is called with an argument whose unit of measurement is `second`, the function return value will have a unit of `second`. Now suppose that a modeler decides to change all uses of seconds to milliseconds in the model. To make the function definition return the same quantity in terms of seconds, the 5 in the formula would need to be changed, but doing so would change the result of the function everywhere it is called—with the wrong consequences in the context where moles were intended. This illustrates the subtle danger of using numbers with unspecified units in function definitions. There are at least two approaches for avoiding this: (1) define separate functions for each case where the units of the constants are supposed to be different, optionally explicitly defining the units of literal numbers; or (2) declare the necessary constants as **Parameter** objects in the model (with declared units!) and pass those parameters as arguments to the function, avoiding the use of literal numbers in the function's formula.

##### *Treatment of unspecified units*

If an expression contains literal numbers and/or SBML components without declared units, the consistency or inconsistency of units may be impossible to determine. In the absence of a verifiable *inconsistency*, an expression in SBML is accepted as-is; the writer of the model is assumed to have written what they intended. However, this is *not* equivalent to assuming the expression *does* have consistent units. The lack of declared units on quantities in an SBML model does not render the model invalid insofar as the SBML specification is concerned, but it reduces the types of consistency checks and useful operations (such as conversions and translations) that software systems can perform.

In some cases, it may be possible to determine that expressions containing unspecified units are inconsistent regardless of what units would be attributed to the unspecified quantities. For example, the expression

$$\frac{dX}{dt} = \frac{[Y] \cdot [Z]^n}{[Z]^m + 1} \cdot V$$

with $X$, $Y$ and $Z$ in units of substance, $V$ in units of volume, and $m \neq n$, cannot ever be consistent, no matter what units the literal 1 takes on. (This also illustrates the need not to stop verifying the units of an expression immediately upon encountering an unspecified quantity—the rest of the expression may still be profitably evaluated and checked for inconsistency.)

##### 3.4.13 SBML does not define implicit unit conversions

Implicit unit conversions do not exist in SBML. Consider the following example. Suppose that in some model, a species $S_1$ has been declared as having a mass of 1 kg, and a second species $S_2$ has been declared as having a mass of 500 g. What should be the result of evaluating an expression such as $S_1 > S_2$? If the numbers alone are considered,

$$1 > 500$$

would evaluate to "`false`", but if the units were implicitly converted by the software tool interpreting the model,

$$1 \text{ kg} > 500 \text{ g}$$

would evaluate to "`true`". This is a trivial example, but the problem for SBML is that implicit unit conversions of this kind can lead to controversial situations where even humans do not agree on the answer. Consequently, SBML only requires that mathematical expressions be evaluated *numerically*. It is up to the model writer to ensure that the units on both sides of an expression match, by inserting explicit unit conversion factors if necessary.

## 4 SBML components

In this section, we define each of the major components of SBML. We use the UML notation described in Section 1.4.3 for defining classes of objects. We also illustrate the use of SBML components by giving partial model definitions in XML. Section 7 provides many complete example models encoded in SBML.

Unless otherwise specified, SBML Level 3 documents must follow the XML specification: anything allowed in XML is allowed in SBML documents, and anything disallowed in XML is not allowed in SBML. All well-formed SBML documents must begin with an *XML declaration*, specifying both the version of XML used and the document character encoding. The declaration begins with the characters `<?xml` followed by the attributes `version` and `encoding`. SBML requires XML version 1.0, and UTF-8 as the document encoding.

### 4.1 The SBML container

Following the XML declaration, the outermost portion of a model expressed in Level 3 consists of an object of class **SBML**, defined in Figure 9. This class contains three required attributes (`level`, `version` and `xmlns`), and an optional `model` element.

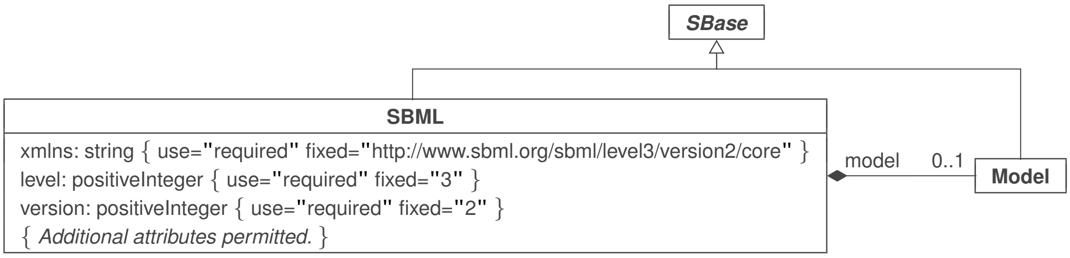

***Figure 9:*** *The definition of class* ***SBML*** *for SBML Level 3 Version 2 Core. The class* ***Model*** *is defined in Section 4.2 on p. 36. Note that* ***SBML*** *and* ***Model*** *are subclasses of* ***SBase****, and therefore inherit the attributes of that abstract class.*

The **SBML** class defines the structure and content of the `sbml` outermost element in an SBML file. The following is an abbreviated example of an **SBML** class object translated into XML form for an SBML Level 3 Version 2 Core document. Here, ellipses ("`...`") are used to indicate content elided from this example:

```
<?xml version="1.0" encoding="UTF-8"?>
<sbml xmlns="http://www.sbml.org/sbml/level3/version2/core" level="3" version="2">
  ...
  <model ...>    ...
  </model>
</sbml>
```

The attribute `xmlns` declares the XML namespace used within the `sbml` element. The URI for SBML Level 3 Version 2 Core is `http://www.sbml.org/sbml/level3/version2/core`. All SBML Level 3 Version 2 Core elements and attributes must be placed in this namespace either by assigning the default namespace as shown in the example above, or using a tag prefix on every element. The `sbml` element may contain additional attributes, in particular, attributes to support the inclusion of SBML Level 3 packages; see Section 4.1.3 on the following page. For purposes of checking conformance to the SBML Level 3 *Core* specification, only the elements and attributes in the SBML Level 3 Core XML namespace are considered.

#### *4.1.1 The `id` and `name` attributes*

Because **SBML** inherits from ***SBase***, it has optional `id`, `name`, `sboTerm` and `metaid` attributes. SBML Level 3 Version 2 Core does not define a purpose for these attributes; moreover, being outside of the **Model** namespace, the `id` attribute is not subject to the uniqueness constraints of `SId` values inside **Model** objects.

#### *4.1.2 The `model` element*

The actual model contained within an SBML document is defined by an instance of the **Model** class element. The structure of this object and its use are described in Section 4.2 on p. 36. An SBML document may contain at most one model definition.

#### 4.1.3 Package declarations

SBML Level 3 is modular, in the sense of having a defined core set of features and optional packages adding features on top of the core. This modular approach means that models can declare which feature-sets they use, and likewise, software tools can declare which packages they support. The mechanism for models to declare which packages they use involves two parts: a standard XML namespace declaration, and an attribute that every package must declare in this namespace.

1. Every SBML Level 3 package is identified uniquely by an XML namespace URI. The use of a given SBML Level 3 package must be declared by a model using the standard XML namespace declaration approach. The declaration is made using the character sequence "`xmlns:`" (without the quotes), followed by additional characters providing a prefix by which elements and attributes in that namespace are known in the rest of the SBML document, and finally followed by the namespace URI as a value. The following is an example of namespace declarations for a package nicknamed "`comp`" and another package nicknamed "`layout`" (and here, ellipses are used to indicate content elided from this example):

```
<sbml xmlns="http://www.sbml.org/sbml/level3/version2/core" level="3" version="2"
      xmlns:comp="http://www.sbml.org/sbml/level3/version2/comp/version2"
      xmlns:layout="http://www.sbml.org/sbml/level3/version2/layout/version2" ...>
  ...
</sbml>
```

There are no restrictions on the prefixes used for XML namespaces referring to SBML Level 3 packages beyond those imposed by the relevant specifications of XML 1.0 and XML namespaces. (In other words, the prefix strings "`comp`" and "`layout`" in the example above are arbitrarily chosen, and could have been something else.)

2. SBML Level 3 requires that every package defines the addition of at least one attribute named `required`. The attribute, being in the namespace of the Level 3 package in question, must be referenced by the XML namespace prefix described in point number 1 above. The value of the `required` attribute indicates whether constructs in that package may change the mathematical interpretation of constructs defined in SBML Level 3 Core. A `required` value of "`true`" indicates that the package may do so. The value is set by the relevant Level 3 package specification, and does *not* depend on the presence or absence of particular package constructs in a given SBML document: in other words, it is a property of the package. For example, if a package defines new object classes with mathematical meanings and with identifiers that may be used in core **Math** elements, that package's `required` attribute must always be set to "`true`" for all SBML documents declaring the use of that package's namespace. The following is a complete example:

```
<sbml xmlns="http://www.sbml.org/sbml/level3/version2/core" level="3" version="2"
      xmlns:comp="http://www.sbml.org/sbml/level3/version2/comp/version2"
      xmlns:layout="http://www.sbml.org/sbml/level3/version2/layout/version2"
      comp:required="true" layout:required="false">
  <model />
</sbml>
```

In this example, because the Hierarchical Model Composition ("`comp`") package can change the mathematical interpretation of a model, the `comp:required` attribute must always be set to "`true`", even if a model is completely empty. The Graphical Layout ("`layout`") package, by contrast, cannot ever change the mathematical interpretation of any model; thus, its `required` attribute must be set to "`false`".

If a package is declared optional (i.e., its `required` flag is set to "`false`"), it means the time-course dynamics of the model can be correctly inferred even if the elements and attributes added by that particular SBML package are ignored. "Ignoring" a package can be accomplished in multiple ways: a reader could either skip those elements or attributes altogether during parsing, or read them but not interpret them, or do something similar. The following are some ways in which packages with `required`="`true`" may change the time-course dynamics of a model:

- An element with a (potential) mathematical meaning may be left undefined in the core specification of SBML Level 3, but may have a value defined by a package.

- An element with mathematical meaning may have a value defined by a package such that the value overrides the value given in the core SBML specification.
- The definition of how an element changes over time may be altered or augmented by a package.
- An element may be defined within a package to have mathematical meaning, and its `SId` may be used to determine one or more mathematical functions in core SBML. (However, no package may ever give mathematical meaning to the `SId` of an element that was defined as not having mathematical meaning: no `SId` of this type may ever appear in any mathematical context.)

The XML namespace declaration for an SBML Level 3 package is an indication that a model makes use of features defined by that package, while the `required` attribute indicates whether the features may be ignored without compromising the mathematical meaning of the model. Both are necessary for a complete reference to an SBML Level 3 package. (On the other hand, no declaration is necessary for the Level 3 Core package, since it is the base package and support for it is required in any case.)

### 4.2 Model

The definition of **Model** is shown in Figure 10 on the following page. Only one instance of a **Model** object is allowed per instance of an SBML Level 3 Version 2 Core document or data stream, and it must be located inside the `<sbml> ... </sbml>` element as described in Section 4.1 on p. 34.

**Model** serves as a container for components of classes **FunctionDefinition**, **UnitDefinition**, **Compartment**, **Species**, **Parameter**, **InitialAssignment**, ***Rule***, **Constraint**, **Reaction** and **Event**. Instances of the classes are placed inside instances of classes **ListOfFunctionDefinitions**, **ListOfUnitDefinitions**, **ListOfCompartments**, **ListOfSpecies**, **ListOfParameters**, **ListOfInitialAssignments**, **ListOfRules**, **ListOfConstraints**, **ListOfReactions**, and **ListOfEvents**. The "list" classes are defined in Figure 10 on the following page. All of the lists are optional, and, if present, may be empty; this is semantically equivalent to omitting the list. The resulting XML data object for a full model containing every possible list would have the following form:

```
<?xml version="1.0" encoding="UTF-8"?>
<sbml xmlns="http://www.sbml.org/sbml/level3/version2/core" level="3" version="2">
    <model id="My_Model">
        <listOfFunctionDefinitions>
            zero or more <functionDefinition> ... </functionDefinition> elements     } optional
        </listOfFunctionDefinitions>
        <listOfUnitDefinitions>
            zero or more <unitDefinition> ... </unitDefinition> elements     } optional
        </listOfUnitDefinitions>
        <listOfCompartments>
            zero or more <compartment> ... </compartment> elements     } optional
        </listOfCompartments>
        <listOfSpecies>
            zero or more <species> ... </species> elements     } optional
        </listOfSpecies>
        <listOfParameters>
            zero or more <parameter> ... </parameter> elements     } optional
        </listOfParameters>
        <listOfInitialAssignments>
            zero or more <initialAssignment> ... </initialAssignment> elements     } optional
        </listOfInitialAssignments>
        <listOfRules>
            zero or more elements of subclasses of Rule     } optional
        </listOfRules>
        <listOfConstraints>
            zero or more <constraint> ... </constraint> elements     } optional
        </listOfConstraints>
        <listOfReactions>
            zero or more <reaction> ... </reaction> elements     } optional
        </listOfReactions>
        <listOfEvents>
            zero or more <event> ... </event> elements     } optional
        </listOfEvents>
    </model>
</sbml>
```

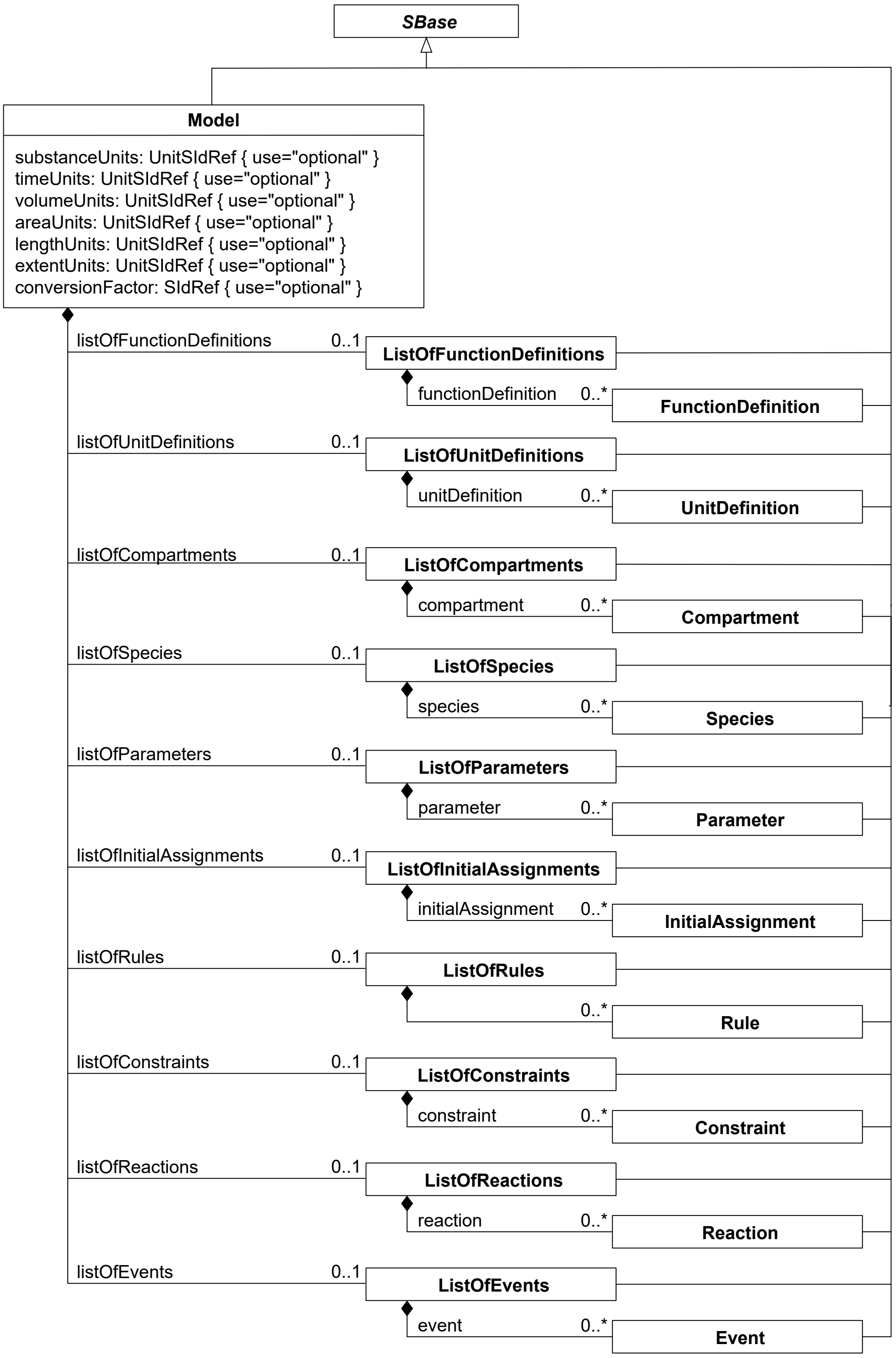

***Figure 10:*** *The definition of* ***Model*** *and the many helper classes* ***ListOfFunctionDefinitions****,* ***ListOfUnitDefinitions****,* ***ListOfCompartments****,* ***ListOfSpecies****,* ***ListOfParameters****,* ***ListOfInitialAssignments****,* ***ListOfRules****,* ***ListOfConstraints****,* ***ListOfReactions****, and* ***ListOfEvents****.*

Although the lists are optional, there are dependencies between SBML components such that defining some components requires defining others. For example, defining a species requires defining a compartment, and defining a species reference in a reaction requires defining a species. Such dependencies are explained throughout this document.

##### 4.2.1 The sboTerm attribute

**Model** inherits an optional sboTerm attribute of type SBOTerm from its parent class ***SBase*** (see Section 3.1.12 and Section 5). When a value is given to this attribute in a **Model** instance, it should be an SBO identifier belonging to the branch for type **Model** indicated in Table 6 on p. 98. The relationship is of the form "the model definition *is-a* X", where X is the SBO term. The term chosen should be the most precise (narrow) one that captures the overall process or phenomenon represented by the overall SBML model.

##### 4.2.2 The substanceUnits attribute

The substanceUnits attribute is used to specify the unit of measurement associated with substance quantities of **Species** objects that do not specify units explicitly. The attribute's value must be of type UnitSIdRef (Section 3.1.10 on p. 13). A list of recommended units is given in Section 8.2.1 on p. 148.

If a given **Species** object definition does not specify its unit of substance quantity via the substanceUnits attribute on **Species** (described in Section 4.6 on p. 49), then the species inherits the value of the **Model** substanceUnits attribute. If the **Model** does not define a value for this attribute, then there is no unit to inherit, and all species that do not specify individual substanceUnits attribute values then have *no* declared units for their quantities. Section 4.6.4 provides more information about the units of species quantities.

Note that when the identifier of a species appears in a model's mathematical expressions, the unit of measurement associated with that identifier is *not solely determined* by setting substanceUnits on **Model** or **Species**. Section 4.6.5 and Section 4.6.8 explain this point in more detail.

##### 4.2.3 The timeUnits attribute

The timeUnits attribute is used to specify the unit in which time is measured in the model. The value of this attribute must be of type UnitSIdRef (Section 3.1.10 on p. 13). A list of recommended units is given in Section 8.2.1 on p. 148.

This attribute on **Model** is the *only* way to specify a unit for time in a model. It is a global attribute; time is measured in the model everywhere in the same way. This is particularly relevant to **Reaction** and **RateRule** objects in a model: all **Reaction** and **RateRule** objects in SBML define per-time values, and the unit of time is given by the timeUnits attribute on the **Model** object instance. If the **Model** timeUnits attribute has no value, it means that the unit of time is not defined for the model's reactions and rate rules. Leaving it unspecified in an SBML model does not result in an invalid model; however, as a matter of best practice, we strongly recommend that all models specify units of measurement for time.

##### 4.2.4 The volumeUnits, areaUnits and lengthUnits attributes

The attributes volumeUnits, areaUnits and lengthUnits together are used to set the units of measurements for the sizes of **Compartment** objects in the model when those objects do not otherwise specify units. The three attributes correspond to the most common cases of compartment dimensions: volumeUnits for compartments having attribute value spatialDimensions="3", areaUnits for compartments having spatialDimensions="2", and lengthUnits for compartments having spatialDimensions="1". The values of these attributes must be of type UnitSIdRef (Section 3.1.10 on p. 13). A list of recommended units is given in Section 8.2.1 on p. 148. The attributes are not applicable to compartments whose spatialDimensions attribute values are not one of "1", "2" or "3".

If a given **Compartment** object instance does not provide a value for its units attribute, then the unit of measurement of that compartment's size is inherited from the value specified by the **Model** volumeUnits, areaUnits or lengthUnits attribute, as appropriate based on the **Compartment** object's spatialDimensions attribute value. If the **Model** object does not define the relevant attribute, then there are no units to inherit, and all compartments that do not set a value for their units attribute then have *no* units associated with their compartment sizes. Section 4.5.4 provides more information about units of compartment sizes.

The use of three separate attributes is a carry-over from SBML Level 2. Note that it is entirely possible for a model to define a value for two or more of the attributes volumeUnits, areaUnits and lengthUnits simultaneously, because SBML models may contain compartments with different numbers of dimensions.

##### 4.2.5 The extentUnits attribute

Reactions are processes that occur over time. These processes involve events of some sort, where a single "reaction event" is one in which some set of entities (known as reactants, products and modifiers in SBML) interact, once. The *extent* of a reaction is a measure of how many times the reaction has occurred, while the time derivative of the extent gives the instantaneous rate at which the reaction is occurring. Thus, what is colloquially referred to as the "rate of the reaction" is in fact equal to the rate of change of reaction extent.

The combination of extentUnits and timeUnits defines the units of kinetic laws in SBML and establishes how the numerical value of each **KineticLaw**'s mathematical formula (Section 4.11.5 on p. 74) is meant to be interpreted in a model. The units of the kinetic laws are taken to be extentUnits divided by timeUnits. A list of recommended units is given in Section 8.2.1 on p. 148.

Note that this embodies an important principle in SBML models: *all reactions in an SBML model must have the same units* for the rate of change of extent. In other words, the units of all reaction rates in the model *must be the same*. There is only one global value for extentUnits and one global value for timeUnits.

##### 4.2.6 The conversionFactor attribute

The attribute conversionFactor defines a global value inherited by all **Species** object instances that do not define separate values for their conversionFactor attributes. The value of this attribute must be of type SIdRef (Section 3.1.8 on p. 13) and refer to a **Parameter** object instance defined in the model. The **Parameter** object in question must be a constant; i.e., it must have its constant attribute value set to "true".

If a given **Species** object definition does not specify a conversion factor via the conversionFactor attribute on **Species** (described in Section 4.6 on p. 49), then the species inherits the conversion factor specified by the **Model** conversionFactor attribute. If the **Model** does not define a value for this attribute, then there is no conversion factor to inherit. Section 4.11.7 on p. 77 describes how to interpret the effects of reactions on species in that situation. More information about conversion factors in SBML is provided in Section 4.6 on p. 49 and Section 4.11 on p. 68.

##### 4.2.7 The ListOf container classes

The various **ListOf**_____ classes defined in Figure 10 on p. 37 are merely containers used for organizing the main components of an SBML document. **ListOfFunctionDefinitions**, **ListOfUnitDefinitions**, **ListOfCompartments**, **ListOfSpecies**, **ListOfParameters**, **ListOfInitialAssignments**, **ListOfRules**, **ListOfConstraints**, **ListOfReactions**, and **ListOfEvents** are all derived from the abstract class ***SBase*** (Section 3.2 on p. 14), and inherit ***SBase***'s various attributes and subelements. The **ListOf**_____ classes do not add any attributes of their own.

There are several motivations for grouping SBML components within XML elements with names of the form listOfClassNames rather than placing all the components directly at the top level. First, the fact that the container classes are derived from ***SBase*** means that software tools can add information about the lists themselves into each list container's **Annotation**, a feature that a number of today's software tools exploit. Second, we believe the grouping leads to a more modular structure that is helpful when working with elements from multiple SBML Level 3 packages. Third, we believe that it makes visual reading of models in XML easier, for situations when humans must inspect and edit SBML directly.

Lists are allowed to be empty for two reasons. First, this allows model writers to add **Annotation** and **Notes** objects to a given list even when the list is empty in a model; this can be useful, for instance, to let a modeler explain *why* the components are absent from the model. Second, it enables SBML Level 3 package specifications to define new elements to be children of these lists, and for these child elements to be used without the requirement that at least one SBML Level 3 Core element always be present.

### 4.3 Function definitions

The **FunctionDefinition** object associates an identifier with a function definition. This identifier can then be used as the function called in subsequent MathML `apply` elements. **FunctionDefinition** is shown in Figure 11.

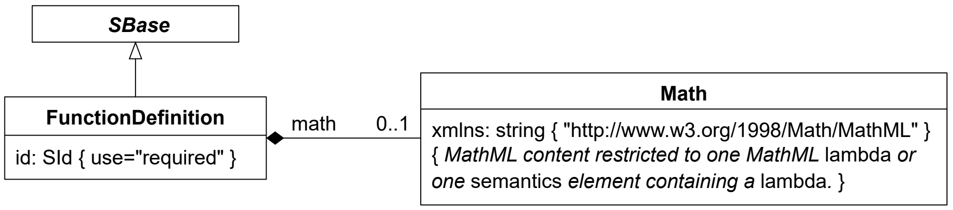

***Figure 11:*** *The definition of class* ***FunctionDefinition****. A* ***Lambda*** *class object must contain a single MathML* `lambda` *expression (or a* `lambda` *surrounded by a* `semantics` *element). A function definition may contain no more than one* `math` *element defined by the* ***Lambda*** *class. Note also that* ***Lambda*** *is not derived from* ***SBase****, which means that the attributes defined on* ***SBase*** *are* not *available on the* `math` *element. A sequence of zero or more instances of* ***FunctionDefinition*** *objects can be located in an instance of* ***ListOfFunctionDefinitions*** *in* ***Model****, as shown in Figure 10 on p. 37.*

Function definitions in SBML (also informally known as "user-defined functions") have purposefully limited capabilities. As is made clearer below, a function cannot reference parameters or other model quantities outside of itself; values must be passed as parameters to the function. Moreover, recursive and mutually-recursive functions are not permitted. The purpose of these limitations is to balance power against complexity of implementation. With the restrictions as they are, function definitions could, if desired, be implemented as textual substitutions. Software implementations therefore do not need the full function-definition machinery typically associated with programming languages.

#### *4.3.1 The `id` attribute*

**FunctionDefinition** inherits the `id` attribute from ***SBase***, but defines `id` as being *required* rather than optional. The `id` attribute otherwise obeys the behavior described in Section 3.3 on p. 18.

A function's identifier can be used in `math` elements as the first target of an `apply` element, but the identifier has no value associated with it, and may not be the target of an **InitialAssignment**, **EventAssignment**, or ***Rule*** object in the model. MathML `ci` elements in an SBML model can refer to the function defined by a **FunctionDefinition** using the value of its `id` attribute.

#### *4.3.2 The `math` element*

The optional `math` element is a container for MathML content that defines the function. The content of this element can only be a MathML `lambda` element or a MathML `semantics` element containing a `lambda` element. **FunctionDefinition** is the only place in SBML Level 3 Core where a `lambda` element can be used. If present, the `lambda` element must begin with zero or more `bvar` elements, followed by any other of the elements in the MathML subset listed in Section 3.4.1 on p. 20 *except* `lambda` (i.e., a `lambda` element cannot contain another `lambda` element).

A further restriction on the content of `math` is it cannot contain references to identifiers other than the symbols declared in the `lambda` itself. That is, the contents of MathML `ci` elements inside the body of the `lambda` can only be one of three kinds of identifiers: (i) the symbols declared by its `bvar` elements, or (ii) the identifiers of other **FunctionDefinition** objects defined in the same model. This restriction also applies to the `csymbol` elements for *time*, *avogadro*, *delay*, and *rateOf*. Functions must be written so that all model symbols they use are passed to them via their parameters.

If the `math` element is not present in the **FunctionDefinition**, the function has no mathematical meaning defined in SBML Level 3 Core. This situation may arise when models are incomplete, or when additional meaning or subobjects are provided by an SBML Level 3 package. (However, from the perspective of a model reader that only understands SBML Level 3 Core, the additional meaning will not be recognized.)

##### 4.3.3 The `sboTerm` attribute

**FunctionDefinition** inherits an optional `sboTerm` attribute of type `SBOTerm` from its parent class ***SBase*** (see Section 3.1.12 and Section 5 on p. 91). When a value is given to this attribute in a **FunctionDefinition** instance, it should be an SBO identifier belonging to the branch for type **FunctionDefinition** indicated in Table 6 on p. 98. The relationship is of the form "the function definition *is-a* X", where X is the SBO term. The term chosen should be the most precise (narrow) one that captures the role of the function in the model.

##### 4.3.4 Calling user-defined functions

Within MathML expressions in an SBML model, all calls to a function defined by a **FunctionDefinition** must use the same number of arguments as specified in the function's definition. The number of arguments is equal to the number of `bvar` elements inside the `lambda` element of the function definition. If the **FunctionDefinition** has no `math` child, SBML Level 3 Core places no restriction on the number of arguments the function may take when used in MathML elsewhere in a model. Additional restrictions may be placed by SBML Level 3 packages. However, without additional content or meaning defined by SBML Level 3 packages, a call to a function that has no `math` content is mathematically meaningless in a model.

Note that **FunctionDefinition** does not have a separate attribute for defining the unit of measurement associated with the value returned by the function. The unit is taken to be whatever results from evaluating the expression when the **FunctionDefinition**'s `math` is applied to the arguments supplied in the call to that function. (See also Section 3.4.12 on p. 31.)

##### 4.3.5 Examples

The following abbreviated SBML example shows a **FunctionDefinition** object instance defining `pow3` as the identifier of a function computing the mathematical expression $x^3$, and after that, the invocation of that function in the mathematical formula of a rate law. Note how the invocation of the function uses its identifier.

```
<model ...>
   <listOfFunctionDefinitions>
       <functionDefinition id="pow3">
           <math xmlns="http://www.w3.org/1998/Math/MathML"
                 xmlns:sbml="http://www.sbml.org/sbml/level3/version2/core">
               <lambda>
                   <bvar><ci> x </ci></bvar>
                   <apply> <power/> <ci> x </ci> <cn sbml:units="dimensionless"> 3 </cn>
                   </apply>
               </lambda>
           </math>
       </functionDefinition>
   </listOfFunctionDefinitions>
   ...
   <listOfReactions>
       <reaction id="reaction_1" reversible="true">
           ...
           <kineticLaw>
               <math xmlns="http://www.w3.org/1998/Math/MathML">
                   <apply> <ci> pow3 </ci> <ci> S1 </ci>
                   </apply>
               </math>
           </kineticLaw>
           ...
       </reaction>
   </listOfReactions>
   ...
</model>
```

### 4.4 Unit definitions

The unit of measurement associated with the value produced by a mathematical formula is whatever arises naturally from the components and mathematical expressions comprising the formula, or in other words, the unit obtained by doing dimensional analysis on the formula. To support this, units may be supplied in a number of contexts in an SBML model and associated with a variety of components, and SBML provides a facility for defining units that can be reused and referenced throughout a model. The unit definition facility uses two classes of objects, **UnitDefinition** and **Unit**. Their definitions are shown in Figure 12 and explained in more detail below.

Before delving further into the definition of SBML units, we must highlight two important and sometimes-confusing points. First, unit declarations in SBML models are *optional*. The consequence of this is that a model must be numerically self-consistent independently of unit declarations, for the benefit of software tools that cannot interpret or manipulate units. Unit declarations in SBML are thus more akin to a type of annotation; they can indicate intentions, and can be used by model readers for checking the consistency of the model, labeling simulation output, etc., but any transformations of values implied by different units must be incorporated *explicitly* into a model. We revisit this topic in Section 4.4.4 on p. 45, and illustrate it again with the example given in Section 7.2 on p. 115.

Second, the vast majority of situations that require new SBML unit definitions involve simple multiplicative combinations of base units and factors. An example is “moles per litre per second”. What distinguishes these sorts of unit definitions from more complex ones is that they may be expressed without the use of an additive offset from a zero point. The use of offsets complicates all unit definition systems, yet in the domain of SBML, the real-life cases requiring offsets are few (and in fact, to the best of our knowledge, only involve temperature). Consequently, the SBML unit system has been consciously designed to simplify implementation of unit support for the most common cases in systems biology. The cost of this simplification is to require units with offsets to be handled explicitly by the modeler. Section 8.2.1 on p. 148 discusses approaches for handling situations requiring units with offsets.

#### 4.4.1 UnitDefinition

The approach to defining units in SBML is compositional; for example, *metre second*$^{-2}$ is constructed by combining a **Unit** object representing *metre* with another **Unit** object representing *second*$^{-2}$. The combination is wrapped inside a **UnitDefinition**, which provides for assigning an identifier and optional name to the combination. These object classes are defined in Figure 12. Once a unit is defined using a **UnitDefinition** object, it can then be referenced from elsewhere in a model.

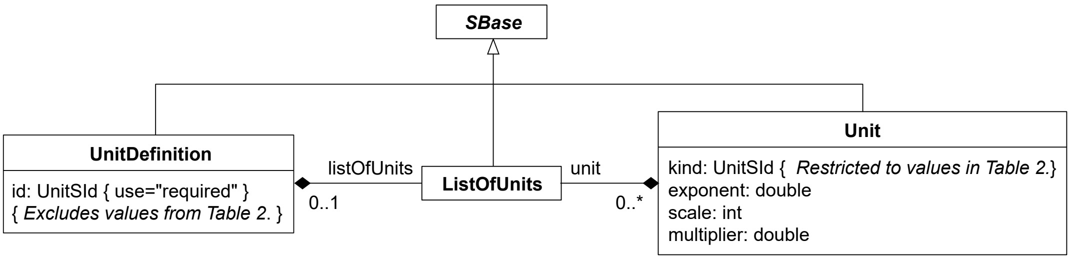

***Figure 12:*** *The definition of classes **UnitDefinition** and **Unit**. A sequence of zero or more instances of **UnitDefinition** can be located in an instance of **ListOfUnitDefinitions** in **Model** (Figure 10). **ListOfUnits** has no attributes (beyond those it inherits from class **SBase**); it merely acts as a container for zero or more instances of **Unit** objects. Note that the only permitted values of* `kind` *on **Unit** are the reserved words in Table 2 on p. 44, but these symbols are excluded from the permitted values of **UnitDefinition**'s* `id` *because SBML's unit system does not allow redefining the base units.*

##### *The* `id` *attribute*

**UnitDefinition** inherits the `id` attribute from ***SBase***, but defines `id` as being required rather than optional, and in addition, refines the data type of `id` to be `UnitSId` instead of `SId`. This is done to provide each unit definition with a unique identifier by which other components of a model may refer to it. (See Section 3.3 on p. 18 for more details.)

There is one important restriction about the use of unit definition `id` values: the `id` of a **UnitDefinition** must *not* be equal to one of the reserved base unit names listed in Table 2 on the following page, the list of reserved base unit names. This constraint simply prevents the redefinition of base units.

##### *The list of Units*

A **UnitDefinition** object may contain a **ListOfUnits** container which may contain zero or more **Unit** objects. Section 4.4.2 explains the meaning and use of **Unit**.

In SBML Level 3 Version 2 Core, the list of units in a **UnitDefinition** object may be empty. This is permitted for the same reasons that other lists are permitted to be empty. (See the discussion in Section 4.2.7 on p. 39.) An empty unit definition is synonymous with an undefined unit. For example, suppose a given component in a model is defined to have a unit of measurement "`u`". If the **UnitDefinition** object with identifier "`u`" has an empty **ListOfUnits** subobject, then this is identical to leaving the unit undefined on the component.

##### *Example*

The following skeleton of a unit definition illustrates an example use of **UnitDefinition**:

```
<model ...>
    <listOfUnitDefinitions>
        <unitDefinition id="unit1">
            <listOfUnits>
                ...
            </listOfUnits>
        </unitDefinition>
        <unitDefinition id="unit2">
            <listOfUnits>
                ...
            </listOfUnits>
        </unitDefinition>
    </listOfUnitDefinitions>
    ...
</model>
```

#### 4.4.2 Unit

A **Unit** object represents a reference to a (possibly transformed) base unit chosen from the list in Table 2 on the next page. The attribute `kind` indicates the base unit, whereas the attributes `exponent`, `scale`, and `multiplier` define how the base unit is being transformed. The attributes are described in detail below.

##### *The `kind` attribute*

The **Unit** attribute `kind` specifies a base unit to serve as the starting point for a new unit definition. The value of the attribute must be taken from the list of reserved words given in Table 2 on the following page. These reserved symbols are defined in the value space of the data type `UnitSId` (Section 3.1.9 on p. 13).

Note that the set of acceptable values for the attribute `kind` does *not* include units defined by **UnitDefinition** objects. This means that the unit definition system in SBML is not hierarchical: user-defined units cannot be built on top of other user-defined units, only on top of base units.

The presence of `avogadro` in Table 2 on the following page warrants an explanation. The Bureau International des Poids et Mesures specifically states, "When the mole is used, the elementary entities must be specified and may be atoms, molecules, ions, electrons, other particles, or specified groups of such particles" (Bureau International des Poids et Mesures, 2006, p. 115)—in other words, the SI unit `mole` is technically *a unit of measure for substance amount*. Although people sometimes use "mole" loosely to refer to other things besides substance amounts (e.g., "a mole of $X$" to mean a number of $X$ equal to Avogadro's number, $6.022 \cdot 10^{23}$), such usage is not strictly correct. We believe it is even less correct in the context of reactions: although in SBML they are called "reactions", there is nothing preventing the SBML **Reaction** construct from being used to represent other kinds of processes not involving substances. Consequently, we avoid using "`mole`" loosely where substances may not be involved, and instead use "Avogadro's number of $X$". In order to make it

*Table 2:* *Base units defined in SBML. These symbols are predefined values of type* `UnitSId` *(Section 3.1.9 on p. 13). All are names of base or derived SI units (Bureau International des Poids et Mesures, 2006), except for "*`avogadro`*", "*`dimensionless`*" and "*`item`*", which are SBML additions. The unit "*`dimensionless`*" is intended for cases where a quantity is a ratio whose units cancel out, the unit "*`avogadro`*" is the unit "*`dimensionless`*" multiplied with Avogadro's number, and "*`item`*" is used for expressing such things as "N items" when "mole" is not an appropriate unit. The gram and litre are not strictly part of SI; however, they are frequently used in SBML's areas of application and therefore are included as predefined unit identifiers. (The standard SI unit of mass is the kilogram, and volume in SI is defined in terms of cubic metres.) Comparisons of these values, like all values of type* `UnitSId`*, must be performed in a case-sensitive manner.*

| | | | | | | | | |
|---|---|---|---|---|---|---|---|---|
| ampere | coulomb | gray | joule | litre | mole | radian | steradian | weber |
| avogadro | dimensionless | henry | katal | lumen | newton | second | tesla | |
| becquerel | farad | hertz | kelvin | lux | ohm | siemens | volt | |
| candela | gram | item | kilogram | metre | pascal | sievert | watt | |

easier for models to define units in these terms, the SBML unit system includes the pseudo-unit "`avogadro`", whose definition is identical to the definition of the *avogadro* `csymbol` described in Section 3.4.6 on p. 25. The numerical value is taken to be the one recommended by CODATA (Mohr et al., 2008), but the unit is `dimensionless`. In other words, it is defined as

$$(6.02214179 \cdot 10^{23}) \cdot \texttt{dimensionless}$$

where the dot $(\cdot)$ indicates simple scalar multiplication. If the value of Avogadro's constant is revised by international standards-setting organizations in the future, a future Version of the SBML Level 3 specification may stipulate a new value to be used for `avogadro`. However, all software reading models expressed in *this* version of SBML Level 3 should *always* use the value of Avogadro's constant given above.

Software tools must use identical numerical values of Avogadro's constant for both the base unit `mole` and the unit `avogadro`.

##### *The* `exponent`*,* `scale` *and* `multiplier` *attributes*

The attributes `exponent`, `scale` and `multiplier` work together to permit the use of **Unit** for expressing new units in terms of the base units listed in Table 2. The formula underlying this definition is the following:

$$u_{\text{new}} = (\texttt{multiplier} \cdot 10^{\texttt{scale}} \cdot u_{\texttt{kind}})^{\texttt{exponent}} \quad (1)$$

This formula defines a new unit, $u_{\text{new}}$, in terms of another unit, $u_{\texttt{kind}}$. The unit $u_{\texttt{kind}}$ is one of the units listed in Table 2; in a given **Unit** object, it is chosen by setting the `kind` attribute. Each of the other components on the right-hand side of Equation 1 corresponds to the remaining attributes in a **Unit** object instance, and their meanings are as follows:

- The `multiplier` attribute can be used to multiply the `kind` unit by a real-numbered factor. This enables the definition of units that are not power-of-ten multiples of SI units. For instance, a `multiplier` of 0.3048 could be used to define "`foot`" as a measure of length in terms of a "`metre`". A value of `multiplier` must always be provided in a **Unit** object instance, but the value can be "`1`".
- The `scale` attribute can be used to set the exponent for a power-of-ten multiplier that rescales the unit. For example, a unit having a `kind` value of "`gram`" and a `scale` value of "`-3`" signifies $10^{-3} \cdot gram$, or milligrams. In those cases where a unit does not need to be scaled by a power of ten, the value of `scale` can be set to "`0`" (zero), because $10^0 = 1$.
- The `exponent` attribute can be used to specify an overall exponent on the unit definition. This provides a way to define units such as "cubic metre" in terms of the base unit "`metre`" (for which an `exponent` value of "`3`" would be appropriate). A value of `exponent` must always be provided.

#### *4.4.3 Semantics of Unit and UnitDefinition*

A single **Unit** object instance takes one of the base units from Table 2 and specifies how it should be transformed. A **UnitDefinition** object instance combines one or more **Unit** objects to define a new, composed unit, $u$. The new unit $u$ created by a **UnitDefinition** is defined as the product of all the **Unit** objects contained in the **ListOfUnits** within the **UnitDefinition** object instance. More formally,

$$u = u_1 \cdot u_2 \cdot \ldots \cdot u_n \tag{2}$$

where the $\{u_i\}$'s are individual **Unit** definitions as defined by Equation 1. Now, let the value of the `multiplier` attribute of a given unit $\{u_i\}$ be represented by the symbol $m_i$. Similarly, let the value of the `scale` attribute be represented by $s_i$, and the value of the `exponent` attribute be represented by $x_i$. Equation 2 can be rewritten in expanded form as

$$\begin{aligned} u &= (m_1 \cdot 10^{s_1} \cdot u_{b_1})^{x_1} \cdot (m_2 \cdot 10^{s_2} \cdot u_{b_2})^{x_2} \cdot \ldots \cdot (m_n \cdot 10^{s_n} \cdot u_{b_n})^{x_n} \\ &= m_1^{x_1} \cdot m_2^{x_2} \cdot \ldots \cdot m_n^{x_n} \cdot 10^{(s_1 x_1 + s_2 x_2 + \ldots + s_n x_n)} \cdot u_{b_1}^{x_1} \cdot u_{b_2}^{x_2} \cdot \ldots \cdot u_{b_n}^{x_n} \\ &= m \cdot 10^{s} \cdot u_{b_1}^{x_1} \cdot u_{b_2}^{x_2} \cdot \ldots \cdot u_{b_n}^{x_n} \end{aligned} \tag{3}$$

where the terms $m$ and $s$ in the last line (Equation 3) are defined as

$$\begin{aligned} m &= m_1^{x_1} \cdot m_2^{x_2} \cdot \ldots \cdot m_n^{x_n} \\ s &= s_1 x_1 + s_2 x_2 + \ldots + s_n x_n \end{aligned}$$

Equation 3 expresses how a **UnitDefinition** object instance combines multiple **Unit** object instances to produce a new unit definition in an SBML model.

#### *Examples*

As a concrete example to illustrate the definitions above, let us define a unit for "`foot`" in terms of the base unit "`metre`". This requires using `multiplier`="`0.3048`", `scale`="`0`", and `exponent`="`1`":

$$\text{foot} = 0.3048 \cdot 10^{0} \cdot \texttt{metre}$$

The following fragment of SBML illustrates how this could be represented in XML:

```
<listOfUnitDefinitions>
    <unitDefinition id="foot">
        <listOfUnits>
            <unit kind="metre" multiplier="0.3048" scale="0" exponent="1"/>
        </listOfUnits>
    </unitDefinition>
</listOfUnitDefinitions>
```

To give another example, the following illustrates the definition of an abbreviation "`mmls`" for the unit millimoles $\text{l}^{-1}$ $\text{s}^{-1}$:

```
<listOfUnitDefinitions>
    <unitDefinition id="mmls">
        <listOfUnits>
            <unit kind="mole"   exponent="1"  scale="-3" multiplier="1"/>
            <unit kind="litre"  exponent="-1" scale="0"  multiplier="1"/>
            <unit kind="second" exponent="-1" scale="0"  multiplier="1"/>
        </listOfUnits>
    </unitDefinition>
</listOfUnitDefinitions>
```

Section 8.2.1 on p. 148 provides suggestions for possible ways of handling cases that involve offsets, which happen in particular with temperature measurements.

#### 4.4.4 Use of units in a model

As already mentioned, unit declarations are optional in SBML. This design decision was a consensus choice among SBML developers and users, driven by the exigencies of non-commercial software development and the realities of models found in existence. It has an important and possibly counterintuitive implication that must be kept in mind when writing and interpreting SBML models: units associated with quantities *do not alter the numerical interpretation* of those quantities.

An example may help make this more clear. We know that one metre equals 1000 millimetres:

$$1\,\mathrm{m} = 1000\,\mathrm{mm}$$

However, the equality above relies on interpreting the units on both sides, and taking the "1" and "1000" to be dimensionless. If readers ignored unit labels altogether or were unable to process them, they would see

$$1 = 1000$$

which is obviously incorrect. In an SBML model, the necessary factor must be included explicitly, even if it is part of the definition of the unit. A ramification of this is that units attached to SBML quantities must be viewed as a kind of independent annotation or label that does not enter into the numerical interpretation of the actual quantity or the mathematical formulas appearing in a model. In the present simple formula, an explicit factor such as the following needs to be inserted (and here we put unit names in { } braces to indicate they are annotations that do not enter into the mathematics):

$$1\,\{\mathrm{m}\} = 1000 \cdot \frac{1\,\{\mathrm{m}\}}{1000\,\{\mathrm{mm}\}}\,\{\mathrm{mm}\} \tag{4}$$

This is despite the fact that a unit definition for millimetres in SBML would take the following form:

```
<listOfUnitDefinitions>
    <unitDefinition id="millimetre">
        <listOfUnits>
            <unit kind="metre" exponent="1" scale="-3" multiplier="1"/>
        </listOfUnits>
    </unitDefinition>
</listOfUnitDefinitions>
```

In other words, the definition *also* includes a factor of 1/1000. While the result is that information is duplicated between the definition of `millimetre` above and the explicit factor inserted into Equation 4, the machinery provided by **UnitDefinition** is still necessary in order to allow units themselves to be properly defined. The result is still useful and powerful: it permits software tools to check the consistency of a model, perform unit conversions, label numbers in the outputs of simulations, and so on.

Finally, as mentioned above, unit definitions in SBML Level 3 Version 2 Core may have empty **ListOfUnits** substructures. A **UnitDefinition** with no child **Unit** elements is not fully defined, and a model component that references this **UnitDefinition** is taken to have *no* defined unit. A model with an empty unit definition may indicate an incomplete model or a model that needs external information.

### 4.5 Compartments

A *compartment* in SBML represents a bounded space in which species are located. Compartments do not necessarily have to correspond to actual structures inside or outside of a biological system, although models are often designed that way. The definition of **Compartment** is shown in Figure 13 on the following page.

It is important to note that although compartments are optional in the overall definition of **Model**, every species in an SBML model must be located in a compartment. This in turn means that if a model defines any species, the model must also define at least one compartment. The reason is simply that species represent physical things, and therefore must exist *somewhere*. Compartments represent the *somewhere*.

#### 4.5.1 The id attribute

**Compartment** inherits the `id` attribute from ***SBase***; however, **Compartment** defines `id` as being required rather than optional. The attribute otherwise behaves as described in Section 3.3 on p. 18.

The identifier (the `id` attribute value) of a **Compartment** object may be used in a model's mathematical expressions. The identifier stands for the value of the compartment's `size` attribute, with a unit of mea-

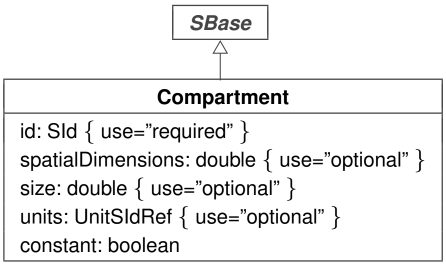

***Figure 13:*** *The definition of class* ***Compartment***. *A sequence of zero or more instances of* ***Compartment*** *objects can be located in an instance of* ***ListOfCompartments*** *in* ***Model***, *as shown in Figure 10 on p. 37.*

surement as defined in Section 4.5.4. A compartment's identifier may be the target of an **InitialAssignment**, **EventAssignment**, or ***Rule*** in the model, to set or redefine the value of the compartment size.

##### *4.5.2 The* `spatialDimensions` *attribute*

A **Compartment** object has an optional floating-point attribute named `spatialDimensions` whose value indicates the number of spatial dimensions possessed by the compartment. Most modeling scenarios involve compartments with integer values of `spatialDimensions`="3" (i.e., a three-dimensional compartment, which is to say, a volume), `spatialDimensions`="2" (i.e., a two-dimensional compartment, a surface), or `spatialDimensions`="1" (i.e., a one-dimensional compartment, which is to say, a line). However, SBML Level 3 does not restrict compartments' `spatialDimensions` values, in order to allow for the possibility of representing structures with fractal dimensions.

In SBML Level 3 Version 2 Core, the number of spatial dimensions possessed by a compartment cannot enter into mathematical formulas, and therefore cannot directly alter the numerical interpretation of a model. However, the value of `spatialDimensions` does affect the interpretation of units. Specifically, the value of `spatialDimensions` is used to select among the **Model** attributes `volumeUnits`, `areaUnits` and `lengthUnits` when a **Compartment** object does not define a value for its `units` attribute. This is described in more detail below in Section 4.5.4 on the following page.

##### *4.5.3 The* `size` *attribute*

The optional **Compartment** attribute `size`, with a data type of `double`, can be used to set the initial size of the compartment. The size may correspond to a volume (if the compartment is a three-dimensional one), or it may be an area (if the compartment is two-dimensional), or a length (if the compartment is one-dimensional).

A compartment's size is set by its `size` attribute exactly once. If the compartment's attribute `constant` has the value "`true`", then the compartment's size is fixed and cannot be changed except by an **InitialAssignment** in the model. The approach of using an **InitialAssignment** differs from setting the `size` attribute in that `size` can only be used to set the compartment size to a literal floating-point number, whereas **InitialAssignment** allows the value to be set using an arbitrary mathematical expression (which, thanks to MathML's expressiveness, may evaluate to a rational number). If the compartment's `constant` attribute is "`false`", the size value may be overridden by an **InitialAssignment** or changed by an **AssignmentRule** or **AlgebraicRule**, and in addition, for simulation time $t > 0$, it may also be changed by a **RateRule** or **Event**. (However, some constructs are mutually exclusive; see Section 4.9 on p. 59 and Section 4.12 on p. 79.) It is not an error to set the value of `size` on a compartment and also redefine the value using an **InitialAssignment**, but the original `size` value in that case is ignored. Section 3.4.8 provides additional information about the semantics of assignments, rules and values for simulation time $t \leq 0$.

It is important to note that in SBML Level 3, a missing `size` value *does not imply that the compartment size is "*`1`*"*. A missing value for `size` for a given compartment signifies that the value either is unknown, or to be obtained from an external source, or determined by an initial assignment (Section 4.8 on p. 56) or other SBML construct elsewhere in the model. Further, due to the fact that alternative methods exist for setting the size of a compartment, the `size` attribute must be defined as optional; however, *it is good*

*practice to specify a value for the size of every compartment in a model*, no matter what method is used, when compartment size values are available. The reasons for this are explained in Section 8.2.2 on p. 150.

#### 4.5.4 The `units` attribute

The measurement unit associated with the value of the compartment's `size` attribute may be specified using the optional attribute `units`. This attribute's value must have the data type `UnitSIdRef` (Section 3.1.10).

The `units` attribute may be left unspecified for a given compartment in a model; in that case, the compartment inherits the unit of measurement specified by one of the attributes on the enclosing **Model** object instance. The applicable attribute on **Model** depends on the value of the compartment's `spatialDimensions` attribute; the relationship is shown in Table 3. If the **Model** object does not define the relevant attribute (`volumeUnits`, `areaUnits` or `lengthUnits`) for a given `spatialDimensions` value, the unit associated with that **Compartment** object's size is undefined. If *both* `spatialDimensions` and `units` are left unset on a given **Compartment** object instance, then no unit can be chosen from among the **Model**'s `volumeUnits`, `areaUnits` or `lengthUnits` attributes (even if the **Model** instance provides values for those attributes), because there is no basis to select between them and there is no default value of `spatialDimensions`. Leaving the units of compartments' sizes undefined in an SBML model does not render the model invalid; however, as a matter of best practice, we strongly recommend that all models specify the units of measurement for all compartment sizes. A discussion of recommended units is given in Section 8.2.1 on p. 148.

The unit of measurement associated with a compartment's size, as defined by the `units` attribute or (if `units` is not set) the inherited value from **Model** according to Table 3, is used in the following ways:

- When the identifier of the compartment appears as a numerical quantity in a mathematical formula expressed in MathML (discussed in Section 3.4.3 on p. 23), it represents the size of the compartment, and the unit associated with the size is the value of the `units` attribute.
- When a **Species** is to be treated in terms of concentrations or density, the unit associated with the spatial size portion of the concentration value (i.e., the denominator in the formula $amount/size$) is specified by the value of the `units` attribute on the compartment in which the species is located.
- The `math` elements of **AssignmentRule**, **InitialAssignment** and **EventAssignment** objects setting the value of the compartment size should all have the same units as the unit associated with the compartment's size (see Section 4.9.3 on p. 62 and Section 4.8 on p. 56).
- In a **RateRule** object that defines a rate of change for a compartment's size (Section 4.9.4 on p. 63), the unit of the rule's `math` element should be identical to the compartment's `units` attribute divided by the model-wide unit of *time*. (In other words, {unit of *compartment size*}/{unit of *time*}.)

***Table 3:*** *When a* ***Compartment*** *object instance does not specify a value for the attribute* `units`*, but* does *specify a value for* `spatialDimensions`*, a value for* `units` *is inherited from the enclosing* ***Model*** *instance according to the rules listed above. The left-hand column indicates the value of the compartment's* `spatialDimensions` *attribute, and the middle column indicates the attribute on* ***Model*** *whose value should be used in that case. The right-hand column lists the kinds of units recommended for use in each case.*

| Value of attribute `spatialDimensions` | Attribute of **Model** used for inheriting the unit | Recommended candidate units |
|---|---|---|
| "3" | `volumeUnits` | units of volume, or `dimensionless` |
| "2" | `areaUnits` | units of area, or `dimensionless` |
| "1" | `lengthUnits` | units of length, or `dimensionless` |
| other | *no units inherited* | *no specific recommendations* |

#### 4.5.5 The `constant` attribute

A **Compartment** also has a mandatory `boolean` attribute called `constant` that indicates whether the compartment's size stays constant or can vary during a simulation. A value of "`false`" indicates the compartment's size can be changed by other constructs in SBML. A value of "true" indicates the compartment's size cannot be changed by any construct except **InitialAssignment**. Section 4.5.3 on p. 47 provides more information.

##### 4.5.6 The sboTerm attribute

**Compartment** inherits an optional sboTerm attribute of type SBOTerm from its parent class ***SBase*** (see Section 3.1.12 on p. 14 and Section 5 on p. 91). When a value is given to this attribute in a **Compartment** instance, it should be an SBO identifier belonging to the branch for type **Compartment** indicated in Table 6 on p. 98. The relationship is of the form "the compartment *is-a* X", where X is the SBO term. The term chosen should be the most precise (narrow) one that captures the role of the compartment in the model.

##### 4.5.7 Examples

The following example illustrates three compartments in an abbreviated SBML example of a model definition. The compartment definitions do not set their units attribute, so these compartments inherit units from the model element attributes areaUnits and volumeUnits.

```
<model areaUnits="area" volumeUnits="litre" ...>
    ...
    <listOfUnitDefinitions>
        <unitDefinition id="area">
            <listOfUnits>
                <unit kind="metre" exponent="2" scale="-6" multiplier="1"/>
            </listOfUnits>
        </unitDefinition>
    </listOfUnitDefinitions>
    ...
    <listOfCompartments>
        <compartment id="Extracellular"  spatialDimensions="3" size="1e-14" constant="true"/>
        <compartment id="PlasmaMembrane" spatialDimensions="2" size="1e-14" constant="true"/>
        <compartment id="Cytosol"        spatialDimensions="3" size="1e-15" constant="true"/>
    </listOfCompartments>
    ...
</model>
```

#### 4.6 Species

A *species* in SBML refers to a pool of entities that (a) are considered indistinguishable from each other for the purposes of the model, (b) may participate in reactions, and (c) are located in a specific *compartment*. The SBML **Species** object class is intended to represent these pools. Its definition is shown in Figure 14 on the next page.

##### 4.6.1 The id attribute

**Species** inherits the id attribute from ***SBase***; however, **Species** defines id as being required rather than optional. The attribute otherwise behaves as described in Section 3.3 on p. 18.

The identifier (the id attribute value) of a **Species** object may be used in a model's mathematical expressions. The identifier stands for the given species' amount or concentration, depending on the value of its hasOnlySubstanceUnits attribute as described below, with units of measurement as described in Section 4.6.4 on the next page. A species' identifier may be the target of **InitialAssignment**, **EventAssignment**, or ***Rule*** objects in a model, to set or redefine the value of the species amount or concentration.

##### 4.6.2 The sboTerm attribute

**Species** inherits an optional sboTerm attribute of type SBOTerm from its parent class ***SBase*** (see Section 3.1.12 and Section 5). Values for this attribute should be SBO identifiers taken the branch for type **Species** indicated

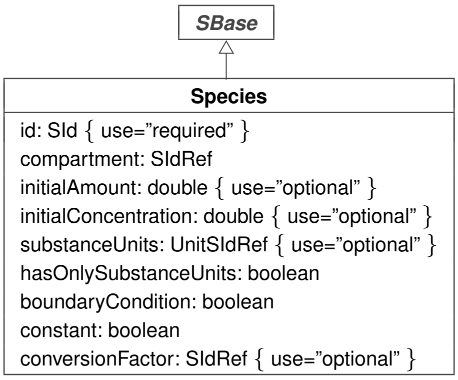

***Figure 14:*** *The definition of class* ***Species****. Zero or more instances of* ***Species*** *objects can be located in an instance of* ***ListOfSpecies*** *in* ***Model****, as shown in Figure 10 on p. 37.*

in Table 6 on p. 98. The relationship is of the form "the species *is-a* X", where X is the SBO term. The term chosen should be the most precise (narrow) one that captures the role of the species in the model.

##### *4.6.3 The `compartment` attribute*

The required attribute `compartment`, of type `SIdRef`, is used to identify the compartment in which the species is located. The attribute's value must be the identifier of an existing **Compartment** object in the model. Note that SBML does not have a concept of a default compartment—every species in an SBML model must be assigned a compartment *explicitly*, by setting the value of the `compartment` attribute. (This also implies that every model with one or more **Species** objects must define at least one **Compartment** object.)

##### *4.6.4 The `initialAmount`, `initialConcentration`, and `substanceUnits` attributes*

The optional attributes `initialAmount` and `initialConcentration`, both having a data type of `double`, can be used to set the initial quantity of the species in the compartment where the species is located. These two attributes are mutually exclusive—either one can be used, but *only one* can have a value on any given instance of a **Species** object. (Setting both is an error.) Missing `initialAmount` and `initialConcentration` values implies that their values either are unknown, or to be obtained from an external source, or determined by an initial assignment (Section 4.8 on p. 56) or other SBML construct elsewhere in the model.

A species' initial quantity is set by the `initialAmount` or `initialConcentration` attributes exactly once. If the `constant` attribute is "`true`", then the value of the species' quantity is fixed and cannot be changed except by an **InitialAssignment**. These methods differ in that the `initialAmount` and `initialConcentration` attributes can only be used to set the species' quantity to a literal floating-point number, whereas the use of an **InitialAssignment** object allows the value to be set using an arbitrary mathematical expression (which, thanks to MathML's expressiveness, may evaluate to a rational number). If the species' `constant` attribute is "`false`", the species' quantity value may be overridden by an **InitialAssignment** or changed by **AssignmentRule** or **AlgebraicRule**, and in addition, for $t > 0$, it may also be changed by a **RateRule**, **Event**, and as a result of being a reactant or product in one or more **Reaction** constructs. (However, some constructs are mutually exclusive; see Section 4.9 on p. 59 and Section 4.12 on p. 79.) It is not an error to define `initialAmount` or `initialConcentration` on a species and also redefine the value using an **InitialAssignment**, but the `initialAmount` or `initialConcentration` setting in that case is ignored. Section 3.4.8 provides additional information about the semantics of assignments, rules and values for simulation time $t \leq 0$.

When the attribute `initialAmount` is set, the unit of measurement associated with its value is specified by the **Species** attribute `substanceUnits`, whose value must have the data type `UnitSIdRef` (Section 3.1.10 on p. 13). When the `initialConcentration` attribute is set, the unit of measurement associated with this concentration value is {unit of *amount*}/{unit of *size*}, where the unit of *amount* is specified by the **Species**

substanceUnits attribute, and the unit of *size* is specified by the units attribute of the **Compartment** object in which the species is located. Note that in either case, a unit of *amount* is involved and determined by the substanceUnits attribute. If the substanceUnits attribute is not set on a given **Species** object instance, then the unit of *amount* for that species is inherited from the substanceUnits attribute on the enclosing **Model** object instance. If that attribute on **Model** is not set either, then the unit associated with the species' quantity is undefined. Leaving units of species quantities undefined in an SBML model does not render the model invalid; however, as a matter of best practice, we strongly recommend that all models specify the units of measurement for all species quantities. A list of recommended units is given in Section 8.2.1 on p. 148.

Simply setting initialAmount or initialConcentration alone does *not* determine whether a species identifier represents an amount or a concentration when it appears elsewhere in an SBML model. Instead, that aspect is controlled by the attribute hasOnlySubstanceUnits, discussed in Section 4.6.5 below.

#### 4.6.5 The hasOnlySubstanceUnits attribute

Independently from how the initial value of a species' quantity is set (Section 4.6.4 on the preceding page), SBML allows choosing the meaning intended for a species' identifier when the identifier appears in mathematical expressions or as the subject of SBML rules or assignments. The interpretation is controlled by the attribute hasOnlySubstanceUnits. If the attribute has the value "false", then the unit of measurement associated with the value of the species is {unit of *amount*}/{unit of *size*} (i.e., concentration or density). If hasOnlySubstanceUnits has the value "true", then the value is interpreted as having a unit of *amount* only.

Although it may seem as though this intention could be determined by either (i) determining whether the initialAmount or initialConcentration attribute is set on a given **Species** object or (ii) examining the particular unit declared by the **Species** or **Model** object's substanceUnits attributes, the fact that all of these attributes are optional means that a separate flag is needed. (Consider the situation where neither is set, and instead the species' quantity is established by an **InitialAssignment** or **AssignmentRule**—something else is then needed to indicate whether the species' identifier represents a concentration or an amount.)

The unit of measurement associated with a species' quantity is used in the following ways in SBML:

- When the species' identifier appears in a MathML formula (discussed in Section 3.4.3 on p. 23), it represents the species' quantity, and the unit of measurement associated with the quantity is as described above.
- The math elements of **AssignmentRule**, **InitialAssignment** and **EventAssignment** objects referring to this species should all have the same units as the unit of measurement associated with the species quantity.
- In a **RateRule** object that defines the rate of change of the species' quantity, the unit associated with the rule's math element should be equal to the unit of the species' quantity (Section 4.6.5) divided by the model-wide unit of *time* (Section 4.2.3); in other words, {unit of *species quantity*}/{unit of *time*}.

#### 4.6.6 The constant and boundaryCondition attributes

The **Species** object has two other mandatory boolean attributes named constant and boundaryCondition, used to indicate whether and how the amount of that species can vary during a simulation. Table 4 on the next page shows how to interpret the combined values of the boundaryCondition and constant attributes.

When a species is a product or reactant of one or more reactions, its amount is determined by those reactions. In SBML, it is possible to indicate that a given species' amount is *not* determined by the set of reactions even when that species occurs as a product or reactant; i.e., the species is on the *boundary* of the reaction system, and its amount is not determined by the reactions. The boolean attribute boundaryCondition with value "true" can be used to indicate this. A value of "false" indicates that the species *is* part of the reaction system.

The constant attribute indicates whether the species' amount can be changed at all, regardless of whether by reactions, rules, or constructs other than **InitialAssignment**. A value of "false" indicates that the species' amount can be changed. This is the most common situation because the purpose of many models is precisely to calculate changes in species quantities over time. Note that the initial quantity of a species can be set by

*Table 4: How to interpret the values of the* `constant` *and* `boundaryCondition` *attributes on* ***Species****. Note that column four is specifically about reactants and products and* not *also about species acting as modifiers; the latter are by definition unchanged by reactions.*

| `constant` value | `boundaryCondition` value | Can have assignment or rate rule? | Can be reactant or product? | What can change the species' amount? |
|---|---|---|---|---|
| true | true | no | yes | (never changes) |
| false | true | yes | yes | rules and events |
| true | false | no | no | (never changes) |
| false | false | yes | yes | reactions *or* rules (but not both), and events |

an **InitialAssignment** irrespective of the value of the `constant` attribute.

In practice, a `boundaryCondition` value of "`true`" means a differential equation derived from the reaction definitions should not be generated for the species. However, the species' amount may still be changed by **AssignmentRule**, **RateRule**, **AlgebraicRule**, **Event**, and **InitialAssignment** constructs if its `constant` attribute is "`false`". Conversely, if both the species' `boundaryCondition` and `constant` attributes are "`true`", then its amount cannot be changed by anything except **InitialAssignment**.

A species having `boundaryCondition`="`false`" and `constant`="`false`" can appear as a product and/or reactant of one or more reactions in the model. If the species is a reactant or product of a reaction, it must not also appear as the target of any **AssignmentRule** or **RateRule** object in the model. If instead the species has `boundaryCondition`="`false`" and `constant`="`true`", then it cannot appear as a reactant or product, or as the target of any **AssignmentRule**, **RateRule** or **EventAssignment** object in the model.

The example model in Section 7.7 on p. 128 contains all four possible combinations of the `boundaryCondition` and `constant` attributes on `species` elements. Section 7.8 on p. 130 gives an example of how one can translate into ODEs a model with species of mixed `boundaryCondition` attribute values.

Finally, it is worth clarifying that while the `constant` and `boundaryCondition` attributes restrict whether and how the species amount changes, the same is not true of a species' concentration. In SBML, the concentration of a species is a quantity that depends on the size of the compartment in which it is located. A compartment's size may change, and therefore, so can the concentration of a species even if the *amount* of the species remains unchanged. A species' concentration may vary even if the **Species** object's `constant`="`true`" in a model.

##### *4.6.7 The* `conversionFactor` *attribute*

The attribute `conversionFactor` defines a conversion factor that applies to a particular species. The value of the attribute must have the data type `SIdRef` and must be the identifier of a **Parameter** object instance defined in the model. That **Parameter** object must be a constant, meaning its `constant` attribute must be set to "`true`". If a given **Species** object definition defines a value for its `conversionFactor` attribute, it takes precedence over any factor defined by the **Model** object's `conversionFactor` attribute.

In SBML, the unit of measurement associated with a species' quantity can be different from the unit of extent of reactions in the model. SBML avoids implicit unit conversions by providing an explicit way to indicate any unit conversion that might be required. The use of a conversion factor in computing the effects of reactions on a species' quantity is explained in Section 4.11.7 on p. 78. Because the value of the `conversionFactor` attribute is the identifier of a **Parameter** object, and because parameters can have units attached to them, the transformation from reaction extent units to species units can be completely specified using this approach.

Note that the unit conversion factor is only applied when calculating the effect of a reaction on a species. It is not used in any rules or other SBML constructs that affect the species, and it is also not used when the value of the species is referenced in a mathematical expression.

##### 4.6.8 Additional considerations for interpreting the numerical value of a species

Species are unique in SBML in that they have a kind of duality: a species identifier may stand for either substance amount (meaning, a count of the number of individual entities) or a concentration or density (meaning, amount divided by a compartment size). The previous sections explain the meaning of a species identifier when it is referenced in a mathematical formula or in rules or other SBML constructs; however, it remains to specify what happens to a species when the compartment in which it is located changes in size.

When a species definition has the attribute value `hasOnlySubstanceUnits`="`false`" and the size of the compartment in which the species is located changes, the default in SBML is to assume that it is the concentration that must be updated to account for the size change. This follows from the principle that, all other things held constant, if a compartment simply changes in size, the size change does not in itself cause an increase or decrease in the number of entities of any species in that compartment. In a sense, the default is that the amount of a species is preserved across compartment size changes. Upon such size changes, the value of the concentration or density must be recalculated from the simple relationship *concentration* = *amount*/*size* if the value of the concentration is needed (for example, if the species identifier appears in a mathematical formula or is otherwise referenced in an SBML construct). There is one exception: if the species' quantity is determined by an **AssignmentRule**, **RateRule**, **AlgebraicRule**, or an **EventAssignment** and the species has the attribute value `hasOnlySubstanceUnits`="`false`", it means that the *concentration* is assigned by the rule or event; in that case, the *amount* must be calculated when the compartment size changes. (Events also require additional care in this situation, because an event with multiple assignments could conceivably reassign both a species quantity and a compartment size simultaneously. Section 4.12.5 on p. 86 describes the handling of species in event assignments.)

Note that the above only matters if a species has the attribute value `hasOnlySubstanceUnits`="`false`", meaning that the species identifier refers to a concentration wherever the identifier appears in a mathematical formula. If instead the attribute's value is "`true`", then the identifier of the species *always* stands for an amount wherever it appears in a mathematical formula or is referenced by an SBML construct. In that case, there is never a question about whether an assignment or event is meant to affect the amount or concentration: it is always the amount.

A particularly confusing situation can occur when the species has attribute value `constant`="`true`" in combination with attribute value `hasOnlySubstanceUnits`="`false`". Suppose this species is given a value for `initialConcentration`. Does `constant`="`true`" mean that the concentration is held constant if the compartment size changes? No; it is still the amount that is kept constant across a compartment size change. The fact that the species was initialized using a concentration value is irrelevant.

##### 4.6.9 Example

The following example shows a species definition within an abbreviated SBML model definition. The example shows that species are listed under the heading `listOfSpecies` in the model:

```
<model ...>
    ...
    <listOfSpecies>
        <species id="Glucose" compartment="cell" initialConcentration="4"
                 hasOnlySubstanceUnits="false" boundaryCondition="false" constant="false"/>
    </listOfSpecies>
    ...
</model>
```

#### 4.7 Parameters

A **Parameter** is used in SBML to define a symbol associated with a value; this symbol can then be used in mathematical formulas in a model. The definition of **Parameter** is shown in Figure 15 on the following page.

The use of the term *parameter* in SBML sometimes leads to confusion among readers who have a particular notion of what something called "parameter" should be. It has been the source of heated debate, but despite this, no one has yet found an adequate replacement term that does not have different connotations to different

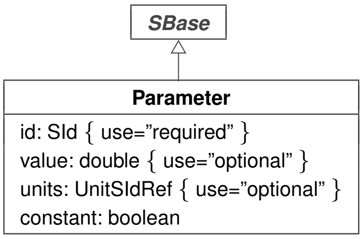

***Figure 15:*** *The definition of class* ***Parameter****. A sequence of zero or more instances of* ***Parameter*** *objects can be located in an instance of* ***ListOfParameters*** *in* ***Model****, as shown in Figure 10 on p. 37.*

people and hence leads to confusion among *some* subset of users. Perhaps it would have been better to have two constructs, one called "constants" and the other called "variables". The current approach in SBML is simply more parsimonious, using a single **Parameter** construct with the `boolean` flag `constant` to indicate which flavor the parameter is. In any case, readers are implored to look past their particular definition of a "parameter" and simply view SBML's **Parameter** as a single mechanism for defining both constants and (additional) variables in a model. (We write *additional* because the species in a model are usually considered to be the central variables.) After all, software tools are not required to expose to users the actual names of particular SBML constructs, and thus tools can present to their users whatever terms their designers feel best matches their target audience.

##### 4.7.1 The `id` attribute

**Parameter** inherits the `id` attribute from ***SBase***, but on **Parameter** it is defined as being required instead of optional. The attribute otherwise behaves as described in Section 3.3 on p. 18.

A parameter's identifier (its `id` attribute value) may be used in a model's mathematical expressions. The identifier stands for the value of the parameter, with a unit of measurement as described in Section 4.7.3 on the following page. It may be the target of **InitialAssignment**, **EventAssignment**, or ***Rule*** objects elsewhere in a model, to set or redefine the value of the parameter.

A **Parameter** `id` will most often represent a `double` value, but the identifier may be used in other contexts. For example, it is possible to have a **Parameter** that is only assigned Boolean values and only used in Boolean contexts. The units of such a **Parameter** should be `dimensionless`. In such cases, it would also be appropriate to set the **Parameter**'s `sboTerm` attribute to the value for "logical parameter" ("`SBO:0000602`").

##### 4.7.2 The `value` attribute

The optional attribute `value` determines the value (of type `double`) assigned to the identifier. A missing `value` implies that the `value` either is unknown, or to be obtained from an external source, or determined by an initial assignment (Section 4.8 on p. 56) or other SBML construct elsewhere in the model.

A parameter's value is set by its `value` attribute exactly once. If the parameter's `constant` attribute (Section 4.7.4) has the value "`true`", then the value is fixed and cannot be changed except by an **InitialAssignment**. These two methods of setting the parameter's value differ in that the `value` attribute can only be used to set it to a literal floating-point number, whereas **InitialAssignment** allows the value to be set using an arbitrary mathematical expression (which, thanks to MathML's expressiveness, may evaluate to a rational number). If the parameter's `constant` attribute has the value "`false`", the parameter's value may be overridden by an **InitialAssignment** or changed by **AssignmentRule** or **AlgebraicRule**, and in addition, for simulation time $t > 0$, it may also be changed by a **RateRule** or **Event**. (However, some of these constructs are mutually exclusive; see Section 4.9 and Section 4.12.) It is not an error to define `value` on a parameter and also redefine the value using an **InitialAssignment**, but the `value` in that case is ignored. Section 3.4.8 on p. 28 provides additional information about the semantics of assignments, rules and values for simulation time $t \leq 0$.

##### 4.7.3 The units attribute

The unit of measurement associated with the value of the parameter can be specified using the optional attribute units. The attribute's value must have the data type UnitSIdRef (Section 3.1.10 on p. 13). There are no constraints on the units that can be assigned to parameters in a model; there are also no units to inherit from the enclosing **Model** object (unlike the case for, e.g., **Species** and **Compartment**).

The unit of measurement associated with a parameter's value is used in the following ways:

- When the identifier of the parameter appears as a numerical quantity in a mathematical formula expressed in MathML (discussed in Section 3.4.3 on p. 23), it represents the value of the parameter, and the unit associated with the value is set by the parameter's units attribute.
- The math elements of **AssignmentRule**, **InitialAssignment** and **EventAssignment** objects setting the value of the parameter should all have the same units as the units attribute value of the parameter.
- In a **RateRule** object that defines the rate of change of the parameter's value (Section 4.9.4 on p. 63), the unit associated with the rule's math element should be equal to the parameter's units attribute value divided by the model-wide unit of *time*. (In other words, {parameter units}/{unit of *time*}.)

The fact that the units attribute value is optional means that models can define parameters with undeclared units. Leaving the units of parameter values undefined in an SBML model does not render the model invalid; however, as mentioned elsewhere, as a matter of best practice, we strongly recommend that all models specify units of measurement for all parameters.

##### 4.7.4 The constant attribute

The **Parameter** object has a mandatory boolean attribute named constant that indicates whether the parameter's value can vary during a simulation. A value of "true" indicates the parameter's value cannot be changed by any construct except **InitialAssignment**. Conversely, if constant="false", other constructs in SBML, such as rules and events, can change the value of the parameter. More information about the effects of constant on value is presented in Section 4.7.2 on the preceding page.

What if a parameter has its constant attribute set to "false", but the model does not contain any rules, events or other constructs that ever change its value over time? Although the model may be suspect (why is the parameter in question not flagged as being constant?), this situation is not strictly an error. A value of "false" for constant only indicates that a parameter *can* change value, not that it *must*.

##### 4.7.5 The sboTerm attribute

**Parameter** inherits an optional sboTerm attribute of type SBOTerm from its parent class ***SBase*** (see Section 3.1.12 on p. 14 and Section 5 on p. 91). When a value is given to this attribute in a **Parameter** instance, it should be an SBO identifier belonging to the branch for type **Parameter** indicated in Table 6 on p. 98. The relationship is of the form "the parameter *is-a* X", where X is the SBO term. The term chosen should be the most precise (narrow) one that captures the role of the parameter in the model.

##### 4.7.6 Example

The following is an example of parameters defined at the **Model** level:

```
<model ...>
    ...
    <listOfParameters>
        <parameter id="tau2" value="3e-2" units="second"        constant="true"/>
        <parameter id="Km1"  value="10.7" units="molesperlitre" constant="true"/>
    </listOfParameters>
    ...
</model>
```

### 4.8 Initial assignments

SBML Level 3 Version 2 Core provides two ways of assigning initial values to entities in a model. The simplest and most basic is to set the values of the appropriate attributes in the relevant components; for example, the initial value of a model parameter (whether it is a constant or a variable) can be assigned by setting its `value` attribute directly in the model definition (Section 4.7). However, this approach is not suitable when the value must be calculated, because the initial value attributes on different components such as species, compartments, and parameters are single values and not mathematical expressions. This is the reason for the existence of **InitialAssignment**: to permit the calculation of the value of a constant or the initial value of a variable from the values of *other* quantities in a model. The definition of **InitialAssignment** is shown in Figure 16.

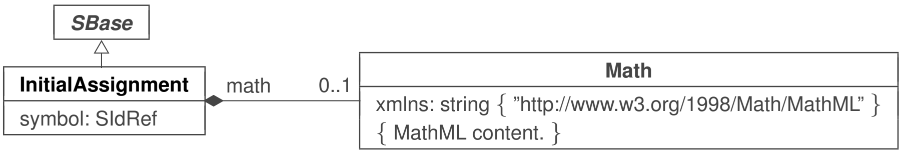

***Figure 16:*** *The definition of class **InitialAssignment**. The contents of the **Math** class can be any MathML permitted in SBML; see Section 3.4.1 on p. 20. A sequence of zero or more instances of **InitialAssignment** objects can be located in an instance of **ListOfInitialAssignments** in **Model**, as shown in Figure 10 on p. 37.*

As explained below, the provision of **InitialAssignment** does not mean that models necessarily must use this construct when defining initial values of quantities. If a value can be set using the relevant attribute of a component in a model, then that approach may be more efficient and more portable to other software tools. **InitialAssignment** should be used when the other mechanism is insufficient for the needs of a particular model.

Initial assignments have some similarities to assignment rules (Section 4.9.3 on p. 62). The main differences are (a) unlike **AssignmentRule**, an **InitialAssignment** definition only applies up to and including the beginning of simulation time, i.e., $t \leq 0$, while an **AssignmentRule** applies at all times; and (b) an **InitialAssignment** can set the value of a constant whereas an **AssignmentRule** cannot.

#### 4.8.1 The `id` attribute

**InitialAssignment** inherits an optional `id` attribute of type `SId` from ***SBase***. The identifier of an **InitialAssignment** has no mathematical meaning in an SBML Level 3 Version 2 Core model.

#### 4.8.2 The `symbol` attribute

**InitialAssignment** contains the mandatory attribute `symbol`, of type `SIdRef`. The purpose of **InitialAssignment** is to define the initial value of the constant or variable referred to by the `symbol` attribute. (The attribute's name is `symbol` rather than `variable` because it may assign values to constants as well as variables in a model; see Section 4.8.5 on the next page.)

The value of the `symbol` attribute must be the identifier of an object in the `SId` namespace of the model; moreover, the object must be of a class that is defined to have mathematical meaning in SBML. In SBML Level 3 Core, the types of objects whose identifiers are permitted as the values of **InitialAssignment** `symbol` attributes are **Compartment**, **Species**, **SpeciesReference** and (global) **Parameter** objects in the model. In addition, classes of objects defined by SBML Level 3 packages to have mathematical meaning may also be defined by those packages to be permissible targets of **InitialAssignment** objects; in other words, **InitialAssignment** `symbol` attributes may also reference identifiers in the `SId` namespace defined by SBML Level 3 packages.

An initial assignment cannot be made to reaction identifiers; that is, the `symbol` attribute value of an **InitialAssignment** cannot be an identifier that is the `id` attribute value of a **Reaction** object in the model. This is identical to a restriction placed on rules (see Section 4.9.5 on p. 64). It may also not reference the `id` of a **FunctionDefinition**.

If the symbol attribute of an **InitialAssignment** object references an object in an SBML namespace that is not recognized by the interpreter reading a given SBML document (that is, if the object is defined by an SBML Level 3 package that the software does not support), the assignment must be ignored—the symbol must not be assigned by the construct if the interpreter cannot understand the package. If an interpreter cannot establish whether a referenced object is missing from the model or instead is defined in an SBML namespace not recognized by the interpreter, it may produce a warning to the user. (The latter situation can only arise if an SBML package is present in the SBML document with a package:required attribute of "true".)

##### 4.8.3 The math element

The math element contains a MathML expression used to calculate the value of the entity referenced by symbol. The unit of measurement associated with the value should match the unit associated with the identifier given in the symbol attribute.

An **InitialAssignment** with no math child leaves undefined what assignment is to be made to the corresponding symbol. The absence of a math element is permitted because it is possible for SBML Level 3 packages to add constructs that extend **InitialAssignment** and define how a value is to be computed. In the absence of such constructs in a given model, no assignments or other changes to the model are carried out when there is no math element. This leaves the model unchanged: any SBML component that had a value will continue to have that value; any component whose value was undefined will continue to have an undefined value. A simulator encountering this situation may choose to produce a warning. No other validation rules are affected by the absence of a math child: it is still invalid to have an **InitialAssignment** and an **AssignmentRule** that assign to the same model element, for example.

##### 4.8.4 The sboTerm attribute

**InitialAssignment** inherits an optional sboTerm attribute of type SBOTerm from its parent class ***SBase*** (see Section 3.1.12 and Section 5). When a value is given to this attribute in a **InitialAssignment** instance, it should be an SBO identifier belonging to the branch for type **InitialAssignment** indicated in Table 6 on p. 98. The relationship is of the form "the initial assignment *is-a* X", where X is the SBO term. The term chosen should be the most precise (narrow) one that captures the role of the initial assignment in the model.

##### 4.8.5 Semantics of initial assignments

The value calculated by an **InitialAssignment** object overrides the value assigned to the given symbol by the object defining that symbol. For example, if a **Compartment**'s size is set in its definition, and the model also contains an **InitialAssignment** having that compartment's id as its symbol value, then the interpretation is that the size assigned in the **Compartment** object definition should be ignored and the value assigned based on the computation defined in the **InitialAssignment**. For SBML Level 3 Core, initial assignments can take place for **Compartment**, **Species**, **SpeciesReference** and global **Parameter** objects regardless of the value of their constant attribute.

This does not mean that a definition of a symbol can be omitted if there is an **InitialAssignment** object for that symbol; the symbols must always be defined even if they are assigned a value separately. For example, there must be a **Parameter** definition for a given parameter if there is an **InitialAssignment** for that parameter.

The actions of all **InitialAssignment** objects are in general terms the same, but differ in the precise details depending on the type of symbol being set:

- *In the case of a species*, an **InitialAssignment** sets the referenced species' initial quantity (*concentration* or *amount*) to the value determined by the formula in math. The unit associated with the value produced by the math formula should be equal to the unit associated with the species' quantity. (See Section 4.6.5 on p. 51 for an explanation of how a species' quantity is determined.)

- *In the case of a species reference*, an **InitialAssignment** sets the initial stoichiometry of the reactant or product referenced by the **SpeciesReference** object to the value determined by the formula in math.

The unit associated with the value produced by the `math` formula should be consistent with the unit `dimensionless`, because reactant and product stoichiometries in reactions are dimensionless quantities.

- *In the case of a compartment*, an **InitialAssignment** sets the referenced compartment's initial size to the size determined by the formula in `math`. The unit associated with the value produced by the `math` formula should be the same as that specified for the compartment's size. (See Section 4.5.4 on p. 48 for more information about compartment units.)

- *In the case of a parameter*, an **InitialAssignment** sets the parameter's initial value to the value of the formula in `math`. The unit associated with the value produced by the `math` formula should be the same as parameter's `units` attribute value. (See Section 4.7.3 on p. 55 for more information about parameter units.)

- *In the case of an object from an SBML Level 3 package*, an **InitialAssignment** sets the referenced object's initial value (however such values are defined by the package) to the value of the formula in `math`. The unit of measurement associated with the value produced by the formula should be the same as that object's `units` attribute value (if it has such an attribute), or be equal to the units of model components of that type (if objects of that class are defined by the package as having the same units).

In the context of a simulation, initial assignments establish values that are in effect prior to and including the start of simulation time, i.e., $t \leq 0$. Section 3.4.8 on p. 28 provides information about the interpretation of assignments, rules, and entity values for simulation time up to and including the start time $t = 0$; this is important for establishing the initial conditions of a simulation if the model involves expressions containing the *delay* `csymbol` (Section 3.4.6 on p. 25).

There cannot be two initial assignments for the same symbol in a model; that is, a model must not contain two or more **InitialAssignment** objects that both have the same identifier as their `symbol` attribute value. A model must also not define initial assignments *and* assignment rules for the same entity. That is, there cannot be *both* an **InitialAssignment** and an **AssignmentRule** for the same symbol in a model, because both kinds of constructs apply prior to and at the start of simulated time—allowing both to exist for a given symbol would result in indeterminism. (See also Section 4.9.5 on p. 64.)

The ordering of **InitialAssignment** objects in a model is not significant. The collection of **InitialAssignment**, **AssignmentRule** and **KineticLaw** objects forms a set of assignment statements that must be considered as a whole. The combined set of assignment statements should not contain algebraic loops: a chain of dependency between these statements should terminate. (More formally, consider the directed graph of assignment statements where nodes are a model's assignment statements and directed arcs exist for each occurrence of a symbol in an assignment statement `math` attribute. The directed arcs in this graph start from statements assigning the symbol and end at statements containing the symbol in their `math` elements. Such a graph must be acyclic.) Examples of valid and invalid set of assignment statements are given in Section 4.9.5 on p. 64.

Finally, it is worth being explicit about the expected behavior in the following situation. Suppose (1) a given symbol has a value $x$ assigned to it in its definition, (2) there is an initial assignment having the identifier as its `symbol` value and reassigning the value to $y$, and (3) the identifier is also used in the mathematical formula of a *second* initial assignment. What value should the second initial assignment use? It is $y$, the value assigned to the symbol by the first initial assignment, not whatever value was given in the symbol's definition. This follows directly from the behavior at the defined at the beginning of this section and in Section 3.4.8 on p. 28: if an **InitialAssignment** object exists for a given symbol, then the symbol's value is overridden by that initial assignment.

#### 4.8.6 Example

The following example shows how the species "`x`" can be assigned the initial value $2 \cdot y$, where "`y`" is an identifier defined elsewhere in the model:

```
<listOfSpecies>
    <species id="x" compartment="C" substanceUnits="mole"
             hasOnlySubstanceUnits="true" boundaryCondition="false"
```

```
                     constant="false"/>
        </listOfSpecies>
        ...
        <listOfInitialAssignments>
            <initialAssignment symbol="x">
                <math xmlns="http://www.w3.org/1998/Math/MathML"
                      xmlns:sbml="http://www.sbml.org/sbml/level3/version2/core">
                    <apply>
                        <times/>
                        <ci> y </ci>
                        <cn sbml:units="dimensionless"> 2 </cn>
                    </apply>
                </math>
            </initialAssignment>
        </listOfInitialAssignments>
```

The next example illustrates the more complex behavior discussed above, when a symbol has a value assigned in its definition but there also exists an **InitialAssignment** for it *and* another **InitialAssignment** uses its value in its mathematical formula.

```
        <listOfSpecies>
            <species id="x" initialAmount="50" compartment="C" substanceUnits="item"
                     hasOnlySubstanceUnits="true" boundaryCondition="false" constant="false"/>
        </listOfSpecies>
        ...
        <listOfInitialAssignments>
            <initialAssignment symbol="x">
                <math xmlns="http://www.w3.org/1998/Math/MathML"
                      xmlns:sbml="http://www.sbml.org/sbml/level3/version2/core">
                    <cn sbml:units="item"> 10 </cn>
                </math>
            </initialAssignment>
            <initialAssignment symbol="othersymbol">
                <math xmlns="http://www.w3.org/1998/Math/MathML"
                      xmlns:sbml="http://www.sbml.org/sbml/level3v/version2/core">
                    <apply>
                        <times/>
                        <ci> x </ci>
                        <cn sbml:units="dimensionless"> 2 </cn>
                    </apply>
                </math>
            </initialAssignment>
        </listOfInitialAssignments>
```

The value of "`othersymbol`" in the SBML fragment above will be "`20`". The case illustrates the rule of thumb that if there is an initial assignment for a symbol, the value assigned to the symbol in its definition (here, the value of `initialAmount`) must be ignored and the value created by the initial assignment used instead.

### 4.9 Rules

In SBML, *Rules* provide additional ways to define the values of variables in a model, their relationships, and the dynamical behaviors of those variables. *Rules* enable the encoding of relationships that cannot be expressed using reactions alone (Section 4.11 on p. 68) nor by the assignment of an initial value to a variable in a model (Section 4.8 on p. 56).

SBML separates rules into three subclasses for the benefit of model analysis software. The three subclasses are based on the following three different possible functional forms (where $x$ is a variable, $f$ is some arbitrary function returning a numerical result, $\mathbf{V}$ is a vector of symbols that does not include $x$, and $\mathbf{W}$ is a vector of symbols that may include $x$):

| | | |
|---|---|---|
| *Algebraic* | left-hand side is zero: | $0 = f(\mathbf{W})$ |
| *Assignment* | left-hand side is a scalar: | $x = f(\mathbf{V})$ |
| *Rate* | left-hand side is a rate-of-change: | $dx/dt = f(\mathbf{W})$ |

In their general form given above, there is little to distinguish between *assignment* and *algebraic* rules. They are treated as separate cases for the following reasons:

- *Assignment* rules can simply be evaluated to calculate intermediate values for use in numerical methods;
- SBML needs to place restrictions on assignment rules, for example the restriction that assignment rules cannot contain algebraic loops (discussed further in Section 4.9.5 on p. 64);
- Many simulators do not contain numerical solvers capable of solving unconstrained algebraic equations, and providing more direct forms such as assignment rules may enable those simulators to process models they could not process if the same assignments were put in the form of general algebraic equations;
- Those simulators that *can* solve these algebraic equations make a distinction between the different categories listed above; and
- Some specialized numerical analyses of models may only be applicable to models that do not contain *algebraic* rules.

The approach taken to covering these cases in SBML is to define an abstract ***Rule*** class containing an element, `math`, to hold the right-hand side expression, then to derive subclasses of ***Rule*** that add attributes to distinguish the cases of algebraic, assignment and rate rules. Figure 17 gives the definitions of ***Rule*** and the subclasses derived from it. The figure shows there are three subclasses, **AlgebraicRule**, **AssignmentRule** and **RateRule** derived directly from ***Rule***. These correspond to the cases *Algebraic*, *Assignment*, and *Rate* described above, respectively.

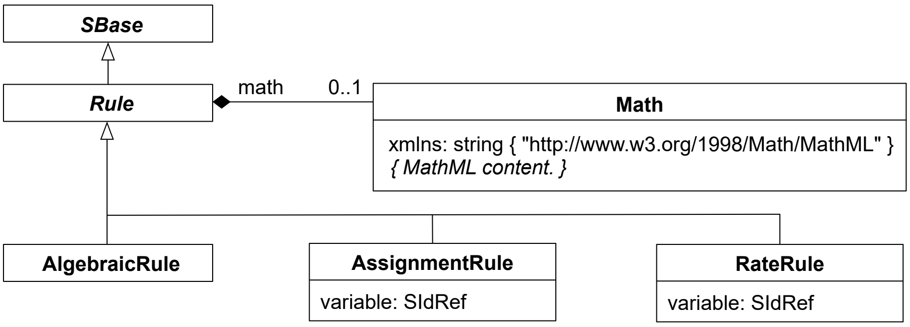

***Figure 17:*** *The definition of* **Rule** *and derived types* ***AlgebraicRule***, ***AssignmentRule*** *and* ***RateRule***.

#### *4.9.1 Common attributes in Rule*

The classes derived from ***Rule*** inherit `math` and the attributes and elements that ***Rule*** itself inherits from ***SBase***, including `id`, `name`, and `sboTerm`.

##### *The* `id` *attribute*

***Rule*** inherits an optional `id` attribute from ***SBase***, of type `SId`. The identifier of a ***Rule***-derived object has no mathematical meaning in an Level 3 Version 2 Core model.

##### *The* `math` *element*

A ***Rule*** object has an optional element called `math`, containing a MathML expression defining the mathematical formula of the rule. This MathML formula must return a numerical value. The formula can be an arbitrary expression referencing the variables and other entities in an SBML model. The interpretation of `math` and its associated unit of measurement are described in more detail in Section 4.9.2, Section 4.9.3 and Section 4.9.4.

A **Rule** with no `math` child element leaves undefined how the rule behaves mathematically. The **Rule** `math` element is defined as optional in SBML Level 3 Core because it is possible for SBML Level 3 packages to add constructs that extend **Rule** and define the missing behavior. In the absence of such constructs in a given model, no assignments or other changes to the model are carried out when there is no `math` element. This leaves the model unchanged: any SBML component that had a value will continue to have that value; any component whose value was undefined will continue to have an undefined value. A simulator encountering this situation may choose to produce a warning. No other validation rules are affected by the absence of a **Rule** `math` element: it is still invalid to have an **InitialAssignment** and an **AssignmentRule** that assign to the same model element, for example.

*The* `sboTerm` *attribute*

**Rule** inherits an optional `sboTerm` attribute of type `SBOTerm` from its parent class **SBase** (see Section 3.1.12 on p. 14 and Section 5 on p. 91). When a value is given to this attribute in an **AlgebraicRule**, **AssignmentRule**, or **RateRule** instance, it should be an SBO identifier belonging to the branch for type **AlgebraicRule**, **AssignmentRule**, or **RateRule** indicated in Table 6 on p. 98. The relationship is of the form "the rule *is-a* X", where X is the SBO term. The term chosen should be the most precise (narrow) one that captures the role of the rule in the model.

#### 4.9.2 AlgebraicRule

The rule type **AlgebraicRule** is used to express equations that are neither assignments of model variables nor rates of change. The **AlgebraicRule** class does not add any attributes to the basic **Rule**; its role is simply to distinguish this case from the other cases. An example of the use of **AlgebraicRule** is given in Section 7.6.

In the context of a simulation, algebraic rules are in effect at all times, $t \geq 0$. To allow evaluating expressions that involve the *delay* `csymbol` (Section 3.4.6), algebraic rules are considered to apply also at $t \leq 0$. Section 3.4.8 describes the semantics of assignments, rules, and entity values for simulation time $t \leq 0$.

An SBML model must not be overdetermined. The ability to define arbitrary algebraic expressions in an SBML model introduces the possibility that a model is mathematically overdetermined by the overall system of equations constructed from its rules, reactions and events. Therefore, if an algebraic rule is introduced in a model, for at least one of the entities referenced in the rule's `math` element the value of that entity must not be completely determined by other constructs in the model. This means that at least this entity must not have the attribute `constant`="`true`" and there must also not be a rate rule or assignment rule for it. Furthermore, if the entity is a **Species** object, its value must not be determined by reactions, which means that it must either have the attribute `boundaryCondition`="`false`" or else not be involved in any reaction at all. These restrictions are explained in more detail in Section 4.9.5 below.

Reaction identifiers can be referenced in the `math` expression of an algebraic rule, but reaction rates can never be *determined* by algebraic rules. This is true even when a reaction does not contain a **KineticLaw** element. (In such cases of missing **KineticLaw** elements, the model is valid but incomplete; the rates of reactions lacking kinetic laws are simply undefined, and not determined by the algebraic rule.)

Finally, any symbol that appears as the target of a *rateOf* `csymbol` may not be determined by an **AlgebraicRule**. This is because the *rateOf* `csymbol` is defined as applying only to symbols whose rates of change are easily determinable.

Although the rules above directly stipulate the symbols that may *not* be determined by the **AlgebraicRule** construct, note that they can also be used to discover the symbol that *is* determined by a given **AlgebraicRule** object. For instance, if three symbols appear in the `math` portion of **AlgebraicRule**, and the first has `constant`="`true`" and the second symbol is a **Reaction** identifier, one may deduce that the **AlgebraicRule** is being used to determine the value of the third symbol that appears in the mathematical expression. This is, in fact, one of the principal uses (in addition to model validation) of the `constant` attribute: it allows software to identify the dependent variables in a model's **AlgebraicRule** objects.

##### 4.9.3 AssignmentRule

The rule type **AssignmentRule** is used to express equations that set the values of variables. The left-hand side (the required `variable` attribute) of an assignment rule is of type `SIdRef`, and must refer to an SBML object in the `SId` namespace with mathematical meaning and the ability to be assigned. In SBML Level 3 Core, this consists of **Species**, **SpeciesReference**, **Compartment**, and global **Parameter** objects in the model (but not reactions nor function definitions). The entity identified must have its `constant` attribute set to the value "`false`". The effects of an **AssignmentRule** are in general terms the same, but differ in the precise details depending on the type of variable being set:

- *In the case of a species*, an **AssignmentRule** sets the referenced species' quantity (whether a *concentration* or *amount*) to the value determined by the formula in `math`. The unit associated with the value produced by the `math` formula should be equal to the unit associated with the species' quantity. (See Section 4.6.5 on p. 51 for an explanation of how a species' quantity is determined.)

*Restrictions*: There must not be both an **AssignmentRule** `variable` attribute and a **SpeciesReference** `species` attribute having the same value, unless that species has its `boundaryCondition` attribute set to "`true`". In other words, an assignment rule cannot be defined for a species that is created or destroyed in a reaction unless that species is defined as a boundary condition in the model.

- *In the case of a species reference*, an **AssignmentRule** sets the stoichiometry of the corresponding reactant or product to the value determined by the formula in `math`. The unit associated with the value produced by the `math` formula should be consistent with the unit `dimensionless`, because reactant and product stoichiometries in reactions are dimensionless quantities.

- *In the case of a compartment*, an **AssignmentRule** sets the referenced compartment's size to the size determined by the formula in `math`. The unit associated with the value produced by the `math` formula should be the same as that specified for the compartment's size. (See Section 4.5.4 on p. 48 for more information about compartment units.)

- *In the case of a parameter*, an **AssignmentRule** sets the referenced parameter's value to the value of the formula in `math`. The unit associated with the value produced by the formula should be the same as parameter's `units` attribute value. (See Section 4.7.3 for more information about parameter units.)

- *In the case of an object from an SBML Level 3 package*, an **AssignmentRule** sets the referenced object's value (as defined by that package) to the value of the formula in `math`. The unit of measurement associated with the value produced by the formula should be the same as that object's `units` attribute value (if it has such an attribute), or be equal to the units of model components of that type (if objects of that class are defined by the package as having the same units).

If the `variable` attribute of an **AssignmentRule** object references an object in an SBML namespace not recognized by the interpreter reading a given SBML document (that is, if the object is defined by an SBML Level 3 package that the software does not support), the assignment rule must be ignored—the object's value must not be assigned by the rule if the interpreter cannot understand the package. If an interpreter cannot establish whether a referenced object is missing from the model or instead is defined in an SBML namespace not recognized by the interpreter, it may produce a warning to the user. (The latter situation can only arise if an SBML package is present in the SBML document with a `package:required` attribute of "`true`".)

In the context of a simulation, assignment rules are in effect at all times, $t \geq 0$. For purposes of evaluating expressions that involve the *delay* `csymbol` (Section 3.4.6), assignment rules are considered to apply also at $t \leq 0$. Section 3.4.8 on p. 28 provides additional information about how $t \leq 0$ should be handled.

A model must not contain more than one **AssignmentRule** or **RateRule** object having the same value of `variable`; in other words, in the set of all assignment rules and rate rules in an SBML model, each variable appearing in the left-hand sides can only appear once. This simply follows from the fact that an indeterminate system would result if a model contained more than one assignment rule for the same variable or both an assignment rule and a rate rule for the same variable.

Similarly, a model must also not contain *both* an **AssignmentRule** and an **InitialAssignment** for the same variable, because both kinds of constructs apply prior to and at the start of simulation time, i.e., $t \leq 0$. If a model contained both an initial assignment and an assignment rule for the same variable, an indeterminate system would result. (See also Section 4.8.5 on p. 57.)

The value calculated by an **AssignmentRule** object overrides the value assigned to the given symbol by the object defining that symbol. For example, if a **Compartment**'s `size` is set in its definition, and the model also contains an **AssignmentRule** having that compartment's `id` as its `variable` value, then the `size` assigned in the **Compartment** definition is ignored and the value assigned based on the computation defined in the **AssignmentRule**. This does *not* mean that a definition for a given symbol can be omitted if there is an **AssignmentRule** object for it. For example, there must be a **Parameter** definition for a given parameter if there is an **AssignmentRule** for that parameter.

#### 4.9.4 RateRule

The rule type **RateRule** is used to express equations that determine the rates of change of variables. The left-hand side (the required `variable` attribute) of a rate rule has type `SIdRef`, and must refer to an SBML object in the `SId` namespace with mathematical meaning and the ability to be assigned. In Level 3 Core, this consists of **Species**, **SpeciesReference**, **Compartment**, and global **Parameter** objects in the model (but not reactions nor function definitions). The entity identified must have its `constant` attribute set to "`false`". The effects of a **RateRule** are in general terms the same, but differ in the precise details depending on which variable is being set:

- *In the case of a species*, a **RateRule** sets the rate of change of the species' quantity (*concentration* or *amount*) to the value determined by the formula in `math`. The unit associated with the rule's `math` element should be equal to the unit of the species' quantity (Section 4.6.5 on p. 51) divided by the model-wide unit of *time* (Section 4.2.3 on p. 38), or in other words, {unit of *species quantity*}/{unit of *time*}.

*Restrictions*: There must not be both a **RateRule** `variable` attribute and a **SpeciesReference** `species` attribute having the same value, unless that species has its `boundaryCondition` attribute is set to "`true`". This means a rate rule cannot be defined for a species that is created or destroyed in a reaction, unless that species is defined as a boundary condition in the model.

- *In the case of a species reference*, a **RateRule** sets the rate of change of the stoichiometry of the referenced reactant or product to the value determined by the formula in `math`. The unit associated with the value produced by the formula should be consistent with {unit derived from `dimensionless`}/{unit of *time*}.

- *In the case of a compartment*, a **RateRule** sets the rate of change of the compartment's size to the value determined by the formula in `math`. The unit of the rule's `math` element should be identical to the compartment's `units` attribute divided by the model-wide unit of *time*. (In other words, {unit of *compartment size*}/{unit of *time*}.)

- *In the case of a parameter*, a **RateRule** sets the rate of change of the parameter's value to that determined by the formula in `math`. The unit associated with the rule's `math` element should be equal to the parameter's `units` attribute value divided by the model-wide unit of *time*. (In other words, {parameter `units`}/{unit of *time*}.)

- *In the case of an object from an SBML Level 3 package*, a **RateRule** sets the rate of change of the referenced object's value (as defined by that package) to the value of the formula in `math`. The unit of measurement associated with the value produced by the formula should be the same as that object's `units` attribute value (if it has such an attribute) divided by the model-wide unit of *time*, or be equal to the units of model components of that type (if objects of that class are defined by the package as having the same units) divided by the model-wide unit of *time*.

If the `variable` attribute of a **RateRule** object references an object in an SBML namespace that is not recognized by the interpreter reading a given SBML document (that is, if the object is defined by an SBML Level 3 package that the software does not support), the rate rule must be ignored—the object's value must not be assigned by the rule if the interpreter cannot understand the package. If an interpreter cannot establish whether a referenced object is missing from the model or instead is defined in an SBML namespace not recognized by the interpreter, it may produce a warning to the user. (The latter situation can only arise if an SBML package is present in the SBML document with a `package:required` attribute of "`true`".)

In the context of a simulation, rate rules are in effect for simulation time $t > 0$. Other types of rules and initial assignments are in effect at different times; Section 3.4.8 on p. 28 describes these conditions.

As mentioned in Section 4.9.3 for **AssignmentRule**, a model must not contain more than one **RateRule** or **AssignmentRule** object having the same value of `variable`; in other words, in the set of all assignment rules and rate rules in an SBML model, each variable appearing in the left-hand sides can only appear once. This simply follows from the fact that an indeterminate system would result if a model contained more than one assignment rule for the same variable or both an assignment rule and a rate rule for the same variable.

#### *4.9.5 Additional restrictions on rules*

An important design goal of SBML rule semantics is to ensure that a model's simulation and analysis results will not be dependent on when or how often rules are evaluated. To achieve this, SBML needs to place two additional restrictions on rule use in addition to the conditions described above regarding the use of **AlgebraicRule**, **AssignmentRule** and **RateRule**. The first concerns algebraic loops in the system of assignments in a model, and the second concerns overdetermined systems.

*The model must not contain algebraic loops*

The combined set of **InitialAssignment**, **AssignmentRule** and **KineticLaw** objects constitute a set of assignment statements that should be considered as a whole. (A **KineticLaw** object is counted as an assignment because it assigns a value to the symbol contained in the `id` attribute of the **Reaction** object in which it is defined.) This combined set of assignment statements must not contain algebraic loops—dependency chains between these statements must terminate. To put this more formally, consider a directed graph in which nodes are assignment statements and directed arcs exist for each occurrence of an SBML species, species reference, compartment or parameter symbol in an assignment statement's `math` element. Let the directed arcs point from the statement assigning the symbol to the statements that contain the symbol in their `math` element expressions. This graph must be acyclic.

Similarly, the combined set of **RateRule** and **Reaction** objects constitute a set of definitions for the rates of change of various model entities (namely, the objects identified by the values of the `variable` attributes of the **RateRule** objects, and the `species` attributes of the **SpeciesReference** objects in each **Reaction**). These rates of change may be referenced directly using the *rateOf* `csymbol`, but may not thereby contain algebraic loops—dependency chains between these statements must terminate. More formally, consider a directed graph in which the nodes are the definitions of different variables' rates of change, and directed arcs exist for each occurrence of a variable referenced by a *rateOf* `csymbol` from any **RateRule** or **KineticLaw** object in the model. Let the directed arcs point from the variable referenced by the *rateOf* `csymbol` (call it $x$) to the variable(s) determined by the `math` expression in which $x$ appears. This graph must be acyclic.

SBML does not specify when or how often rules should be evaluated. Eliminating algebraic loops ensures that assignment statements can be evaluated any number of times without the result of those evaluations changing. As an example, consider the following equations:

$$x = x + 1, \quad y = z + 200, \quad z = y + 100$$

If this set of equations were interpreted as a set of assignment statements, it would be invalid because the rule for $x$ refers to $x$ (exhibiting one type of loop), and the rule for $y$ refers to $z$ while the rule for $z$ refers back to $y$ (exhibiting another type of loop).

Conversely, the following set of equations would constitute a valid set of assignment statements:

$$x = 10, \quad y = z + 200, \quad z = x + 100$$

*The model must not be overdetermined*

An SBML model must not be overdetermined; that is, a model must not define more equations than there are unknowns in a model. An SBML model without **AlgebraicRule** objects cannot be overdetermined.

Assessing whether a given continuous, deterministic, mathematical model is overdetermined does not require dynamic analysis; it can be done by analyzing the system of equations created from the model. It should be noted that when a model contains both reactions and events, there are several sets of equations to consider in order to assess whether a model is overdetermined. The set of equations derived from the combined set of rules and reactions and, for each event, the set of equations derived from the combined set of rules and event assignments for the particular event.

One approach is to construct a bipartite graph in which one set of vertices represents the variables and the other set of vertices represents the equations. The approach involves placing edges between vertices such that variables in the system are linked to the equations that determine them. A mathematical model is overdetermined if the maximal matchings (Chartrand, 1977) of the bipartite graph contain disconnected vertexes representing equations. (If one maximal matching has this property, then all the maximal matchings will have this property; i.e., it is only necessary to find one maximal matching.) Appendix Section B describes a method of applying this procedure to specific SBML data objects. In some cases it is possible to avoid the use of an **AlgebraicRule**. This is discussed in more detail in Section 8.2.3 on p. 151.

#### *4.9.6 Example of rule use*

This section contains an example set of rules. Consider the following set of equations:

$$k = \frac{k_3}{k_2}, \quad s_2 = \frac{k \cdot x}{1 + k_2}, \quad A = 0.10 \cdot x$$

This can be encoded by the following scalar rule set (where the definitions of `x`, `s`, `k`, `k2`, `k3` and `A` are assumed to be located elsewhere in the model and not shown in this abbreviated example):

```
<listOfRules>
    <assignmentRule variable="k">
        <math xmlns="http://www.w3.org/1998/Math/MathML">
            <apply>
                <divide/>
                <ci> k3 </ci>
                <ci> k2 </ci>
            </apply>
        </math>
    </assignmentRule>
    <assignmentRule variable="s2">
        <math xmlns="http://www.w3.org/1998/Math/MathML">
            <apply>
                <divide/>
                    <apply>
                        <times/>
                        <ci> k </ci>
                        <ci> x </ci>
                    </apply>
                    <apply>
                        <plus/>
                        <cn> 1 </cn>
                        <ci> k2 </ci>
                    </apply>
            </apply>
        </math>
    </assignmentRule>
    <assignmentRule variable="A">
        <math xmlns="http://www.w3.org/1998/Math/MathML">
            <apply>
                <times/>
                <cn> 0.10 </cn>
                <ci> x </ci>
            </apply>
        </math>
    </assignmentRule>
</listOfRules>
```

### 4.10 Constraints

The **Constraint** object is a mechanism for stating the assumptions under which a model is designed to operate. The *constraints* are statements about permissible values of different quantities in a model. Figure 18 shows the definition of the **Constraint** object class.

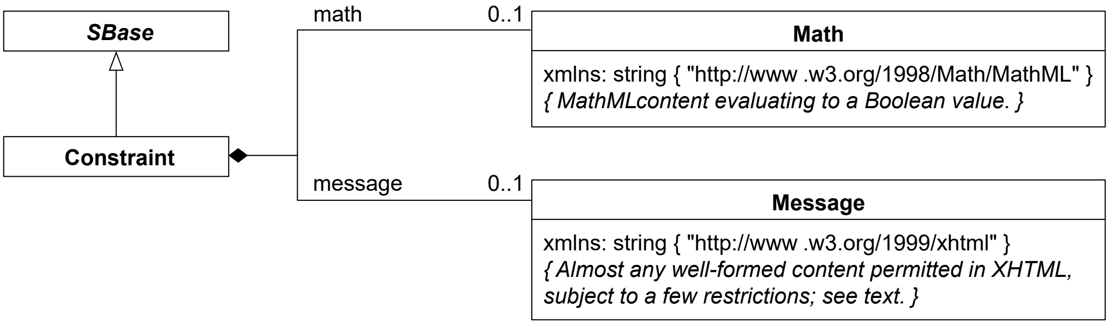

***Figure 18:** The definition of class **Constraint**. The contents of the **Math** class can be any MathML permitted in SBML, and will be interpreted as a Boolean value. As shown above, an instance of **Constraint** can also contain zero or one instances of **Message** objects; this class of object is simply a wrapper (in the XML form, `<message> ... </message>`) for XHTML content. The same guidelines for XHTML content as explained in Section 3.2.5 on p. 15 for notes on **SBase** also apply to the XHTML within messages in a **Constraint**. A sequence of zero or more instances of **Constraint** objects can be located in an instance of **ListOfConstraints** in **Model**, as shown in Figure 10 on p. 37.*

The essential meaning of a constraint is this: if a dynamical analysis of a model (such as a simulation) reaches a state in which a constraint is no longer satisfied, the results of the analysis are deemed invalid beginning with that point in time. The exact behavior of a software tool, upon encountering a constraint violation, is left up to the software; *however*, a software tool must somehow indicate to the user when a model's constraints are no longer satisfied. (Otherwise, a user may not realize that the analysis has reached an invalid state and is potentially producing nonsense results.) If a software tool does not have support for constraints, it should indicate this to the user when encountering a model containing constraints.

*The `id` attribute*

**Constraint** inherits an optional `id` attribute from ***SBase***, of type `SId`. The identifier of a **Constraint** object has no mathematical meaning in an SBML Level 3 Version 2 Core model.

*The `sboTerm` attribute*

**Constraint** inherits an optional `sboTerm` attribute of type `SBOTerm` from its parent class ***SBase*** (see Section 3.1.12 on p. 14 and Section 5 on p. 91). When a value is given to this attribute in a **Constraint** instance, it should be an SBO identifier belonging to the branch for type **Constraint** indicated in Table 6 on p. 98. The relationship is of the form "the constraint *is-a* X", where X is the SBO term. The term chosen should be the most precise (narrow) one that captures the role of the constraint in the model.

#### *4.10.1 The `math` element*

**Constraint** has one optional subelement, `math`, containing a MathML formula defining the condition of the constraint. This formula must return a Boolean value of "`true`" (or any non-zero numerical value) when the model is in a *valid* state. The formula can be an arbitrary expression referencing the variables and other entities in an SBML model. The evaluation of `math` and behavior of constraints are described in more detail in Section 4.10.3 below.

A **Constraint** with no `math` child does not define a mathematical constraint. The absence of a `math` element is permitted because it is possible for SBML Level 3 packages to add constructs that extend **Constraint** and define how a value is to be computed. In the absence of any such construct, no restriction on the model's behavior is implied. A simulator encountering this situation may choose to produce a warning.

#### 4.10.2 Message

A **Constraint** object can contain an optional element named `message` whose content is determined by object class **Message**. This element can contain a message in XHTML format that may be displayed to the user when the condition of the constraint in `math` evaluates to a value of "`false`". Software tools are not required to display the message, but it is recommended that they do so as a matter of best practice.

The XHTML content within a **Message** object must follow the same restrictions as for **Notes** objects described in Section 3.2.5 on p. 15. In particular, the element must declare the use of the XHTML XML namespace, and must not contain an XML declaration nor a DOCTYPE declaration.

#### 4.10.3 Semantics of constraints

In the context of a simulation, a **Constraint** has effect at all times $t \geq 0$. Each **Constraint**'s `math` element is first evaluated after any **InitialAssignment** definitions in a model at $t = 0$ and can conceivably trigger at that point. (In other words, a simulation could fail a constraint immediately.)

The results of a simulation of a model containing a constraint are invalid from any simulation time at and after a point when the function given by the `math` returns a value of "`false`". Invalid simulation results do not make a prediction of the behavior of the biochemical reaction network represented by the model. The precise behavior of simulation tools is left undefined with respect to constraints. If invalid results are detected with respect to a given constraint, the contents of the **Message** subobject (Section 4.10.2) may optionally be displayed to the user. The simulation tool may also halt the simulation or clearly delimit in output data the simulation time point at which the simulation results become invalid.

There are no restrictions on duplicate **Constraint** definitions or the order of evaluation of **Constraint** objects in a model. It is possible for a model to define multiple constraints all with the same `math` element. Since the failure of any constraint indicates the simulation has entered an invalid state, a system is not required to attempt detecting whether other constraints in the model have failed once any one constraint has failed.

#### 4.10.4 Example

As an example, the following SBML fragment demonstrates the constraint that species "`S1`" should only have values between 1 and 100:

```
<model ...>
    ...
    <listOfConstraints>
        <constraint>
            <math xmlns="http://www.w3.org/1998/Math/MathML"
                  xmlns:sbml="http://www.sbml.org/sbml/level3/version2/core">
                <apply>
                    <and/>
                        <apply> <lt/> <cn sbml:units="mole"> 1 </cn> <ci> S1 </ci>
                        </apply>
                        <apply> <lt/> <ci> S1 </ci> <cn sbml:units="mole"> 100 </cn>
                        </apply>
                </apply>
            </math>
            <message>
                <p xmlns="http://www.w3.org/1999/xhtml"> Species S1 is out of range. </p>
            </message>
        </constraint>
    </listOfConstraints>
    ...
</model>
```

### 4.11 Reactions

A *reaction* in SBML represents any kind of process that can change the quantity of one or more species in a model. Examples of such processes can include transformation, transport, molecular interactions, and more. In SBML, the notion of a reaction is generalized to allow entities that may not be chemical substances; thus, a reaction in SBML does not necessarily have to be a biochemical reaction—a biochemical reaction is just one possible kind of process.

At minimum, to describe a reaction in SBML, it is necessary to define its *structural* properties, specifically the participating reactants and/or products (and their corresponding stoichiometries) and the reversibility of the process. In addition, an SBML reaction can also contain a *quantitative* description of the rate of the reaction; this aspect consists of a mathematical formula expressing describing the rate at which the reaction process takes place, together with an optional list of modifier species and parameters influencing the reaction rate. The various parts of a reaction are recorded in the SBML **Reaction** object class and other supporting data classes, defined in Figure 19.

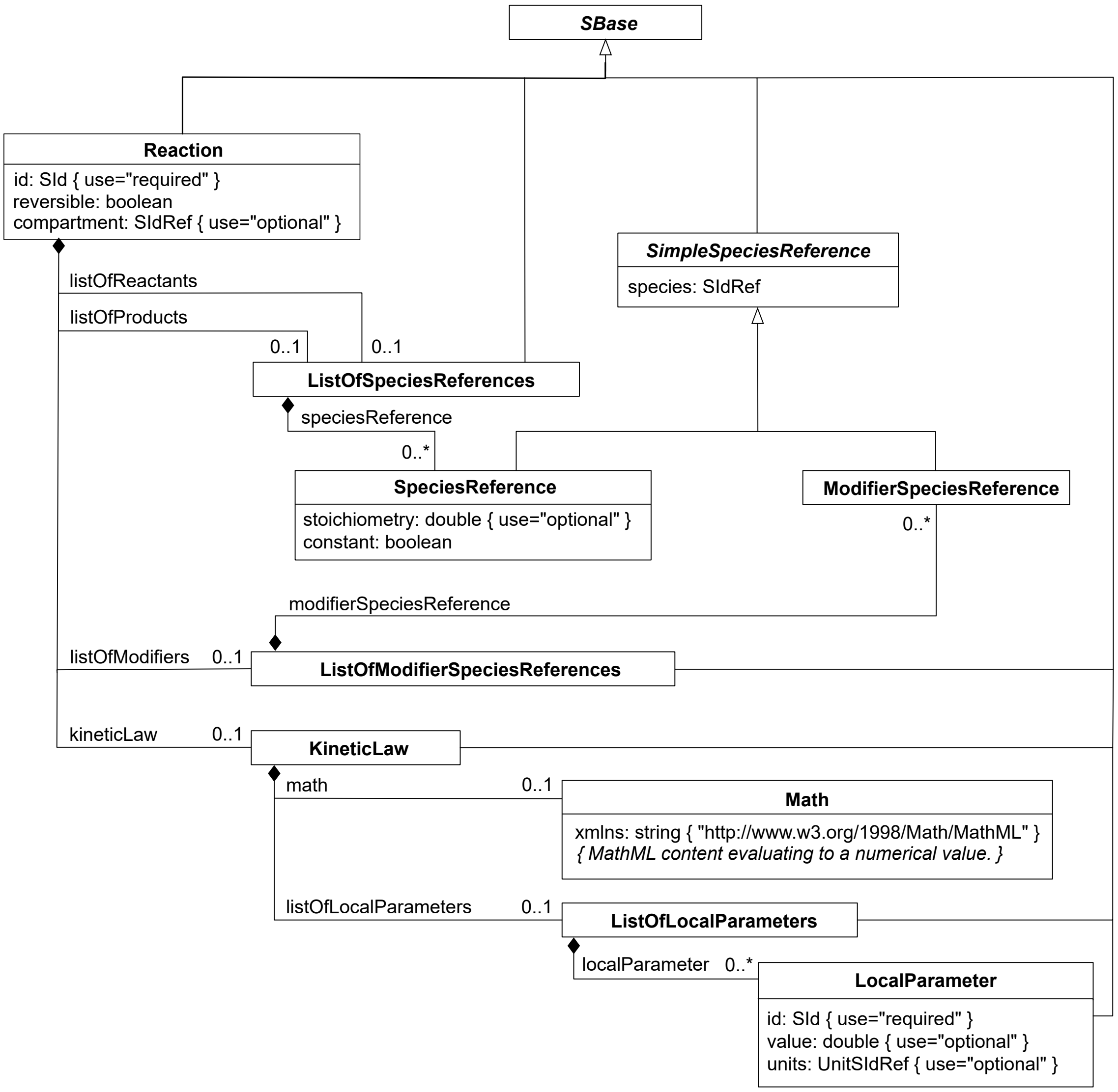

***Figure 19:*** *The definitions of* ***Reaction***, ***KineticLaw***, ***SpeciesReference***, ***ModifierSpeciesReference***, ***LocalParameter****, as well as the container classes* ***ListOfSpeciesReferences***, ***ListOfModifierSpeciesReferences****, and* ***ListOfLocalParameters****. Note:* **SimpleSpeciesReference** *is an abstract class only used to provide some common attributes to derived classes.*

#### 4.11.1 Reaction

Each reaction in an SBML model is defined using an instance of a **Reaction** object. As shown in Figure 19 on the previous page, it contains several scalar attributes and several lists of other objects.

*The* `id` *attribute*

**Reaction** inherits the `id` attribute from ***SBase***; however, **Reaction** defines `id` as being required rather than optional. The attribute otherwise behaves as described in Section 3.3 on p. 18.

The identifier (the `id` attribute value) of a **Reaction** object may be used in mathematical expressions in a model. The identifier stands for the reactions rate; this role and the units of measurement associated with the reaction identifier are explained in more detail in Section 4.11.8 on p. 79. A reaction's identifier cannot be the target of an **InitialAssignment**, **EventAssignment**, or ***Rule*** object, nor may its value be determined by an **AlgebraicRule** object in a model.

*The lists of reactants, products and modifiers*

Each species participating as a reactant, product, and/or modifier in a reaction must be declared using a **SpeciesReference** and/or **ModifierSpeciesReference** object stored in the list elements `listOfReactants`, `listOfProducts` and `listOfModifiers`. The object classes **SpeciesReference** and **ModifierSpeciesReference** are described in more detail in Section 4.11.3 and Section 4.11.4 below. Throughout this text, we use the informal expressions "list of reactants", "list of products" and "list of modifiers" to mean, respectively, the list of species identified by **SpeciesReference** objects within a **Reaction** `listOfReactants` element, the list of species identified by **SpeciesReference** objects within a **Reaction** `listOfProducts` element, and the list of species identified by **ModifierSpeciesReference** objects within a **Reaction** `listOfModifiers` element.

Certain restrictions are placed on the appearance of species in reaction definitions:

- The ability of a species to appear as a reactant or product of any reaction in a model is governed by certain combinations of the attributes `constant` and `boundaryCondition` on the **Species** object instance; see Section 4.6.6 on p. 51 for more information.

- Any species appearing in the `math` element of the `kineticLaw` of a **Reaction** instance must be declared in at least one of that **Reaction**'s lists of reactants, products, and/or modifiers. It is an error for a reaction's kinetic law formula to refer to species that have not been declared for that reaction.

- A reaction definition can contain an empty list of reactants *or* an empty list of products, but it must have at least one reactant or product; in other words, a reaction without any reactant or product species is not permitted. (This restriction does not apply to modifier species, which are always optional.)

*The* `kineticLaw` *element*

A **Reaction** object can contain up to one **KineticLaw** object, in the `kineticLaw` element. This "kinetic law" defines the speed at which the process defined by the reaction takes place. A more detailed description of **KineticLaw** is left to Section 4.11.5 on p. 74 below.

The inclusion of a **KineticLaw** object in an instance of a **Reaction** is optional. For some modeling purposes, models containing reactions without defined rates are an acceptable alternative (and may even be the only possible option, such as when the kinetics of the reactions are unknown). However, missing kinetic laws preclude the application of many model analysis techniques, including simulation. In the absence of any additional definition, some simulators choose to give an error and refuse to simulate models that have a **Reaction** with no **KineticLaw**. Others assume that the effective rate of a **Reaction** with no **KineticLaw** is zero. Still others define this value to be "not a number" (`NaN`). This behavior is not standardized, and should not be relied on when exchanging models for simulation.

##### *The* `reversible` *attribute*

The mandatory `boolean` attribute `reversible` on **Reaction** indicates whether the reaction is reversible. To say that a reaction is *reversible* is to say it can proceed in either the forward or the reverse direction. This information may be redundant in cases where the reversibility of the reaction can be deduced by inspecting the rate formula given in the kinetic law. However, a reaction is not required to have a kinetic law, and besides, when a rate expression is present, it may not always be possible to deduce the reversibility by inspecting it. Having a separate attribute for `reversible` allows certain kinds of structural analysis, such as elementary mode analysis, even in these cases.

Mathematically, the `reversible` attribute on **Reaction** has no impact on the construction of the equations for change of the species' quantities. However, labeling a reaction as irreversible is interpreted as an assertion that the rate expression will not have negative values during a simulation. Software developers may wish to provide their software systems with a means of testing that this condition holds.

The presence of reversibility information in two places (i.e., the rate expression in the kinetic law, and the `reversible` flag) leaves open the possibility that a model could contain contradictory information, but this would be considered to be an error of the encoded model, rather than an invalid SBML encoding.

##### *The* `fast` *attribute (removed)*

In SBML Level 3 Version 2, the `fast` attribute has been removed: every **Reaction** in a Level 3 Version 2 Core model is equivalent to an SBML Level 3 Version 1 **Reaction** with a `fast` value of "`false`". This means that for Level 3 Version 2 Core, the speed of every **Reaction** will always be determined by its **KineticLaw**. To achieve the same or similar effects as setting the `fast` attribute to "`true`" in a previous version of SBML, the **KineticLaw** should be constructed to produce a value in the desired time scale, or the reaction can be replaced with an **AssignmentRule** or **AlgebraicRule** object as in the example of Section 7.5 on p. 124.

##### *The* `compartment` *attribute on* ***Reaction***

The optional attribute `compartment`, of data type `SIdRef`, can be used to indicate the compartment in which the reaction is assumed to take place. If the attribute is present, its value must be the identifier of a **Compartment** object defined in the enclosing **Model** object.

Similar to the `reversible` attribute, the value of the `compartment` attribute has no direct impact on the construction of mathematical equations for the SBML model. When a reaction has a kinetic law, the compartment location may already be implicit in the kinetic law (though this cannot always be guaranteed). Nevertheless, software tools may find the `compartment` attribute value useful for such purposes as analyzing the structure of the model, guiding the modeler in constructing correct rate formulas, and visualization.

##### *The* `sboTerm` *attribute on* ***Reaction***

**Reaction** inherits an optional `sboTerm` attribute of type `SBOTerm` from its parent class ***SBase*** (see Section 3.1.12 on p. 14 and Section 5 on p. 91). When a value is given to this attribute in a **Reaction** instance, it should be an SBO identifier belonging to the branch for type **Reaction** indicated in Table 6 on p. 98. The relationship is of the form "the reaction *is-a* X", where X is the SBO term. The term chosen should be the most precise (narrow) one that captures the role of the reaction in the model.

#### *4.11.2 SimpleSpeciesReference*

As mentioned above, every species that enters into a given reaction must appear in that reaction's lists of reactants, products and/or modifiers. In an SBML model, all species that may participate in any reaction are listed in the **ListOfSpecies** object of the top-level **Model** object instance (see Section 4.2 on p. 36). The lists of products, reactants and modifiers in **Reaction** objects do not introduce new species, but rather, they refer back to those listed in the model's top-level **ListOfSpecies** object. For reactants and products, the connection is made using a **SpeciesReference** object; for modifiers, it is made using a **ModifierSpeciesReference** object. ***SimpleSpeciesReference***, defined in Figure 19 on p. 68, is an abstract type that serves as the parent class of both **SpeciesReference** and **ModifierSpeciesReference**. It is used simply to hold the attributes and elements that are common to the latter two objects.

*The* `species` *attribute*

The ***SimpleSpeciesReference*** object class has a required attribute, `species`, of data type `SIdRef`, inherited by **SpeciesReference** and **ModifierSpeciesReference**. The value of `species` must be the identifier of a species defined in the enclosing **Model**; the referenced species is thereby declared as participating in the reaction being defined. The precise role of that species as a reactant, product, or modifier in the reaction is determined by the subtype of ***SimpleSpeciesReference*** (i.e., either **SpeciesReference** or **ModifierSpeciesReference**) in which the identifier appears and by the specific list of species references in which the **SpeciesReference** appears.

*The* `sboTerm` *attribute*

***SimpleSpeciesReference*** inherits an optional `sboTerm` attribute of type `SBOTerm` from its parent class ***SBase*** (see Section 3.1.12 and Section 5). When a value is given to this attribute in a ***SimpleSpeciesReference*** instance, it should be an SBO identifier belonging to the branch for type ***SimpleSpeciesReference*** indicated in Table 6 on p. 98. The relationship is of the form "the species reference *is-a* X", where X is the SBO term. The term chosen should be the most precise (narrow) one that captures the role of the species reference in the model.

#### 4.11.3 SpeciesReference

**Reaction** provides a way to express which species act as reactants and which species act as products in a reaction, and to declare their stoichiometries. This is done using **SpeciesReference** objects. As mentioned above in Section 4.11.2 on the previous page, **SpeciesReference** inherits the mandatory attribute `species` and optional attributes `id`, `name`, and `sboTerm` from the parent type ***SimpleSpeciesReference***. It also defines the optional attribute `stoichiometry` and the mandatory attribute `constant`, described below.

*The* `id` *attribute*

The `id` attribute that **SpeciesReference** inherits from ***SBase*** is still optional, and behaves as described in Section 3.3 on p. 18. It also acquires the mathematical meaning of the value of its `stoichiometry` attribute, and may be the target of an **InitialAssignment**, **EventAssignment**, or ***Rule*** object defined in the enclosing model.

*The* `stoichiometry` *attribute*

The *stoichiometry* of a species in a reaction describes how much of the species changes when a reaction event takes place. In SBML, product and reactant stoichiometries are specified using the optional `stoichiometry` on **SpeciesReference** object. The `stoichiometry` attribute is of type `double`. A missing `stoichiometry` implies that the stoichiometry is either unknown, or to be obtained from an external source, or determined by an initial assignment (Section 4.8 on p. 56) or other SBML construct elsewhere in the model.

A species reference's stoichiometry is set by its `stoichiometry` attribute exactly once. If the **SpeciesReference** object's `constant` attribute (see below) has the value "`true`", then the stoichiometry is fixed and cannot be changed except by an **InitialAssignment**. These two methods of setting the stoichiometry (i.e., using `stoichiometry` directly, or using an **InitialAssignment**) differ in that the `stoichiometry` attribute can only be set to a literal floating-point number, whereas **InitialAssignment** allows the value to be set using an arbitrary mathematical expression. (As an example, the approach could be used to set the stoichiometry to a rational number of the form $p/q$, where $p$ and $q$ are integers, something that is occasionally useful in the context of biochemical reaction networks.) If the species reference's `constant` attribute has the value "`false`", the species reference's value may be overridden by an **InitialAssignment** or changed by **AssignmentRule** or **AlgebraicRule**, and in addition, for simulation time $t > 0$, it may also be changed by a **RateRule** or **Event**. (However, some of these constructs are mutually exclusive; see Section 4.9 on p. 59 and Section 4.12 on p. 79.) It is not an error to define `stoichiometry` on a species reference and also redefine the stoichiometry using an **InitialAssignment**, but the `stoichiometry` attribute in that case is ignored. Section 3.4.8 on p. 28 provides additional information about the semantics of assignments, rules and values for simulation time $t \leq 0$. Real-world examples of variable stoichiometries in models include genome-scale models (O'Brien et al., 2013), and can generally be found in searches such as `https://scholar.google.com/scholar?q=model+variable+stoichiometry`.

An explanation of how exactly the stoichiometry is used in the mathematical interpretation of the model is given in Section 4.11.7 on p. 77.

##### *The* `constant` *attribute*

The **SpeciesReference** attribute `constant` is a mandatory `boolean` attribute used to indicate whether the `stoichiometry` value can vary during a simulation. If `constant`="`true`", the corresponding species' stoichiometry in the reaction cannot be changed by other constructs elsewhere in the model except by an **InitialAssignment**. A value of "`false`" means the stoichiometry can be changed by other SBML constructs such as rules (see Section 4.9 on p. 59), as described above in the section on the `stoichiometry` attribute.

##### *Use of species reference identifiers in mathematical expressions*

The value of the `id` attribute of a **SpeciesReference** can be used as the content of a `ci` element in MathML formulas elsewhere in the model. When the identifier appears in a `ci` element, it represents the stoichiometry of the corresponding species in the reaction where the **SpeciesReference** object instance appears. More specifically, it represents the value of the `stoichiometry` attribute on the **SpeciesReference** object.

##### *The unit of measurement associated with a **SpeciesReference**'s stoichiometry value*

The unit associated with the value of a species' stoichiometry is always considered to be `dimensionless`. This has the following implications:

- When a species reference's identifier appears in mathematical formulas elsewhere in the model, the unit associated with that value is `dimensionless`.
- The units of the `math` elements of **AssignmentRule**, **InitialAssignment** and **EventAssignment** objects setting the stoichiometry of the species reference should be `dimensionless`.
- If a species reference's identifier is the subject of a **RateRule**, the unit associated with the **RateRule** object's value should be `dimensionless`/*time*, where *time* is the model-wide unit of `time` (Section 4.2.3 on p. 38).

##### *Examples*

The following is a simple example of a species reference for species "`X0`", with stoichiometry "`2`", in a list of reactants within a reaction having the identifier "`J1`":

```
<model ...>
    ...
    <listOfReactions>
        <reaction id="J1" reversible="false">
            <listOfReactants>
                <speciesReference species="X0" stoichiometry="2" constant="true"/>
            </listOfReactants>
            ...
        </reaction>
        ...
    </listOfReactions>
    ...
</model>
```

The following is a more complex example of a species reference with an id "`sr01`" and an initial assignment that assigns a rational number to the stoichiometry:

```
<model ...>
    ...
    <listOfInitialAssignments>
        <initialAssignment symbol="sr01">
            <math xmlns="http://www.w3.org/1998/Math/MathML"
                  xmlns:sbml="http://www.sbml.org/sbml/level3/version2/core">
                <cn type="rational" sbml:units="dimensionless"> 3 <sep/> 2 </cn>
            </math>
        </initialAssignment>
        ...
    </listOfInitialAssignments>
        ...
    <listOfReactions>
        <reaction id="J1" reversible="true">
            <listOfReactants>
                <speciesReference id="sr01" species="X0" constant="true"/>
            </listOfReactants>
            ...
        </reaction>
        ...
    </listOfReactions>
    ...
</model>
```

A species can occur more than once in the lists of reactants and products of a given **Reaction** instance. The effective stoichiometry for the species is the sum of the stoichiometry values given in the **SpeciesReference** objects in the list of products *minus* the sum of stoichiometry values given in the **SpeciesReference** objects in the list of reactants. A positive value indicates the species is effectively a product and a negative value indicates the species is effectively a reactant. SBML places no restrictions on the effective stoichiometry of a species in a reaction; for example, it can be zero. In the following SBML fragment, the two reactions have the same effective stoichiometry for all their species:

```
<reaction id="x" reversible="false">
    <listOfReactants>
        <speciesReference species="a" stoichiometry="1" constant="true"/>
        <speciesReference species="a" stoichiometry="1" constant="true"/>
        <speciesReference species="b" stoichiometry="1" constant="true"/>
    </listOfReactants>
    <listOfProducts>
        <speciesReference species="c" stoichiometry="1" constant="true"/>
        <speciesReference species="b" stoichiometry="1" constant="true"/>
    </listOfProducts>
</reaction>
<reaction id="y" reversible="false">
    <listOfReactants>
        <speciesReference species="a" stoichiometry="2" constant="true"/>
    </listOfReactants>
    <listOfProducts>
        <speciesReference species="c" stoichiometry="1" constant="true"/>
    </listOfProducts>
</reaction>
```

#### 4.11.4 ModifierSpeciesReference

Sometimes a species appears in the kinetic rate formula of a reaction but is neither created nor destroyed in that reaction (for example, because it acts as a catalyst or inhibitor). In SBML, all such species are simply called *modifiers* without regard to the detailed role of those species in the model (though a model could use SBO terms to clarify the roles; see Section 5 on p. 91). The **Reaction** object class provides a way to express which species act as modifiers in a given reaction. This is the purpose of the list of modifiers available in **Reaction**. The list contains instances of **ModifierSpeciesReference** object.

Because its sibling class **SpeciesReference** has mathematical meaning, it is probably worth noting that no meaning is assigned to the identifier of **ModifierSpeciesReference** object instances in SBML Level 3 Version 2 Core, but the identifiers are available for possible use by SBML Level 3 packages. Note that modifiers in reactions also have no stoichiometries and therefore do not possess a `stoichiometry` attribute.

The value of the species attribute must be the identifier of a species defined in the enclosing **Model**; this species is designated as a modifier for the current reaction. A reaction may have any number of modifiers. It is permissible for a modifier species to appear simultaneously in the list of reactants and products of the same reaction where it is designated as a modifier, as well as to appear in the list of reactants, products and modifiers of other reactions in the model.

#### 4.11.5 KineticLaw

The **KineticLaw** object class is used to describe the rate at which the process defined by the **Reaction** takes place. As shown in Figure 19 on p. 68, **KineticLaw** has elements called math and listOfLocalParameters, in addition to the attributes and elements it inherits from ***SBase***.

##### The id attribute

**KineticLaw** inherits an optional id attribute from ***SBase***, of type SId. Despite having a math child, the id of a **KineticLaw** takes on no mathematical meaning; the value of that math element is instead associated with the enclosing **Reaction** object's identifier.

##### The math element

As shown in Figure 19 on p. 68, **KineticLaw** has an element called math for holding a MathML formula defining the rate of the reaction. The expression in math may refer to global identifiers defined in the model as well as **LocalParameter** object identifiers from the **KineticLaw**'s list of local parameters (see below). However, the only **Species** identifiers that can be used in math are those declared in the lists of reactants, products and modifiers in the **Reaction** object (see Section 4.11.2, Section 4.11.3 and Section 4.11.4).

Section 4.11.7 provides important discussions about the meaning and interpretation of SBML "kinetic laws".

A **KineticLaw** with no math child leaves undefined the mathematics of the corresponding **Reaction**. The absence of a math element is permitted because it is possible for SBML Level 3 packages to add constructs that extend **KineticLaw** and define other ways for a value to be computed. In the absence of such package constructs, the model behaves mathematically as if the **KineticLaw** is not defined. This can be sometime useful, for example, when a model needs to define one or more **LocalParameter** objects (which are placed within **KineticLaw** objects) but has no mathematical formula for the kinetics of the reaction involved.

##### The list of local parameters

An instance of **KineticLaw** can contain a list of zero or more **LocalParameter** objects (Section 4.11.6 on the next page) defining new parameters whose identifiers can be used in the math formula. These "local parameters" are optional—a kinetic law can always refer to global **Parameter** objects. The local parameter facility simply provides a way to add additional parameters that may be relevant only to a specific reaction, and that may therefore be better handled by encapsulating their definitions within that kinetic law.

As discussed in Section 3.3.1 on p. 18, reactions introduce local namespaces for local parameter identifiers, and within a **KineticLaw** object (as well as within the parent **Reaction**), a local parameter whose identifier is identical to a global identifier defined in the model takes precedence over the value associated with the global identifier. Note that this introduces the potential for a local parameter definition to shadow a global identifier. SBML does not separate symbols by class of object; consequently, inside the kinetic law mathematical formula, the value of a local parameter having the same identifier as a species, compartment, parameter or other global model entity will override the global value. Modelers and software developers may wish to take precautions to avoid this happening accidentally.

##### The sboTerm attribute

**KineticLaw** inherits an optional sboTerm attribute of type SBOTerm from its parent class ***SBase*** (see Section 3.1.12 on p. 14 and Section 5 on p. 91). When a value is given to this attribute in a **KineticLaw** instance, it should be an SBO identifier belonging to the branch for type **KineticLaw** indicated in Table 6 on p. 98. The relationship is of the form "the kinetic law *is-a* X", where X is the SBO term. The term chosen should be the most precise (narrow) one that captures the role of the kinetic law in the model.

##### 4.11.6 LocalParameter

The **KineticLaw** object within a **Reaction** object can contain a **ListOfLocalParameters** object containing the definitions of *local parameter* that are only accessible within the scope of that particular reaction. The list contains **LocalParameter** objects, each of which associates an identifier with a value. This identifier can then be used in the kinetic law. The definition of **LocalParameter** is shown in Figure 19 on p. 68.

###### *The* `id` *attribute*

**LocalParameter** inherits the `id` attribute from ***SBase***; however, **LocalParameter** defines `id` as being required rather than optional, and in addition, its type is defined to be the derived type of `LocalSId` instead of `SId`. It otherwise behaves as described in Section 3.3 on p. 18.

The identifier (the `id` attribute value) of a **LocalParameter** may be used in the mathematical expression within the enclosing **KineticLaw** object. The identifier stands for the value of the parameter's `value` attribute, and within the scope of a **KineticLaw** object and within the scope of the **Reaction** itself, a local parameter's identifier shadows any identical identifiers from the `SId` namespace of the model. Because of its limited local scope, the identifier also cannot be the target of an **InitialAssignment**, **EventAssignment**, or ***Rule*** object in the model. The units of measurement associated with the identifier are described in Section 4.11.6.

A **LocalParameter** must not have the same `id` value as a **Species** referenced by any of the parent **Reaction**'s **SpeciesReference** objects. (If this were permitted, the local scoping would mean that the **SpeciesReference** would refer to a **LocalParameter**, which is not a **Species**—which would create an inconsistency.)

The effective scope of where a **LocalParameter** `id` can be used in SBML Level 3 Core is restricted to the `math` of the **KineticLaw**, since no other construct within a **Reaction** can reference an `SId`. However, SBML Level 3 packages may introduce new ways of referencing `SId` values within a **Reaction** that could also refer to **LocalParameter** objects. Packages could therefore introduce cases where a **KineticLaw** can exist without a `math` element but with a new package construct that (for example) uses a **ListOfLocalParameters** to provide the package construct with locally-scoped parameters to reference.

###### *The* `value` *attribute*

The optional attribute `value` determines the value (of type `double`) assigned to the identifier. A missing `value` attribute implies that the value either is unknown, or to be obtained from an external source. (Note that, unlike the case with global **Parameter** objects, there is no way in SBML Level 3 Version 2 Core for **InitialAssignment** or other SBML constructs to be used for setting the value of **LocalParameter** objects, because local parameters are local to reactions.)

###### *The* `units` *attribute*

The unit of measurement associated with the value of the parameter can be specified using the optional attribute `units`. The attribute's value must have the data type `UnitSIdRef` (Section 3.1.10 on p. 13). There are no constraints on the units that can be assigned to local parameters in a model; there are also no units to inherit from the enclosing **Model** object (unlike the case for, e.g., **Species** and **Compartment**).

The `units` attribute is used in the following way: when a local parameter's identifier appears in the content of the `math` element of the enclosing **KineticLaw** object, the unit of measurement associated with the local parameter's value is determined by the **LocalParameter** object's `units` attribute.

##### *The* `sboTerm` *attribute*

**LocalParameter** inherits an optional `sboTerm` attribute of type `SBOTerm` from its parent class ***SBase*** (see Section 3.1.12 and Section 5). When a value is given to this attribute in a **LocalParameter** instance, it should be an SBO identifier belonging to the branch for type **LocalParameter** indicated in Table 6 on p. 98. The relationship is of the form "the local parameter *is-a* X", where X is the SBO term. The term chosen should be the most precise (narrow) one that captures the role of the local parameter in the model.

##### *Example*

The following is an example of a **Reaction** object that defines a reaction with identifier $J_1$, in which $X_0 \rightarrow S_1$ at a rate given by $k \cdot [X_0] \cdot [S_2]$, where $S_2$ is a catalyst and $k$ is a parameter, and the square brackets symbolizes that the species quantities are in terms of concentrations. The reaction is assumed to take place all in one compartment identified as "`c1`". The example demonstrates the use of species references, **KineticLaw** objects and local parameters. The units associated with the species identifiers here are $amount/volume$ (see Section 4.6 on p. 49), and so the rate expression $k \cdot [X_0] \cdot [S_2]$ needs to be multiplied by the compartment volume (represented by its identifier, "`c1`") to produce the desired units of $amount/time$ for the rate expression.

```
<model timeUnits="second" extentUnits="mole" substanceUnits="mole">
    <listOfUnitDefinitions>
        <unitDefinition id="per_concent_per_time">
            <listOfUnits>
                <unit kind="litre"  exponent="1"  scale="0" multiplier="1"/>
                <unit kind="mole"   exponent="-1" scale="0" multiplier="1"/>
                <unit kind="second" exponent="-1" scale="0" multiplier="1"/>
            </listOfUnits>
        </unitDefinition>
    </listOfUnitDefinitions>
    <listOfCompartments>
        <compartment id="c1" units="litre" size="0.001" spatialDimensions="3" constant="true"/>
    </listOfCompartments>
    <listOfSpecies>
        <species id="S1" compartment="c1" initialConcentration="2.0"
                 hasOnlySubstanceUnits="false" boundaryCondition="false" constant="false"/>
        <species id="S2" compartment="c1" initialConcentration="0.5"
                 hasOnlySubstanceUnits="false" boundaryCondition="false" constant="false"/>
        <species id="X0" compartment="c1" initialConcentration="1.0"
                 hasOnlySubstanceUnits="false" boundaryCondition="false" constant="false"/>
    </listOfSpecies>
    <listOfReactions>
        <reaction id="J1" reversible="false">
            <listOfReactants>
                <speciesReference species="X0" stoichiometry="1" constant="true"/>
            </listOfReactants>
            <listOfProducts>
                <speciesReference species="S1" stoichiometry="1" constant="true"/>
            </listOfProducts>
            <listOfModifiers>
                <modifierSpeciesReference species="S2"/>
            </listOfModifiers>
            <kineticLaw>
                <math xmlns="http://www.w3.org/1998/Math/MathML">
                    <apply>
                        <times/> <ci> k </ci> <ci> S2 </ci> <ci> X0 </ci> <ci> c1 </ci>
                    </apply>
                </math>
                <listOfLocalParameters>
                    <localParameter id="k" value="0.1" units="per_concent_per_time"/>
                </listOfLocalParameters>
            </kineticLaw>
        </reaction>
    </listOfReactions>
</model>
```

##### 4.11.7 Mathematical interpretation of SBML reactions and kinetic laws

In SBML, *reactions* are the central mechanism for describing processes that change the quantities of species in a model. The *kinetic law* of an SBML reaction provides a quantitative description of the speed with which this happens. In this section, we provide an interpretation of SBML kinetic laws in the framework of a system of ordinary differential equations (ODEs). However, the choice of ODEs as the framework is only for exposition purposes here, in order to allow us to present a concrete mathematical expression of the model in terms that many readers will be familiar with; it is equally possible to translate a model into other frameworks, and some formulations, such as discrete stochastic systems, are indeed quite common.

###### Semantics of rate law and stoichiometry

The *stoichiometry* of a species $S$ in a reaction describes the proportion, relative to other species participating in that reaction, of $S$ involved in each reaction event. For example, in a reaction $S_1 + 2S_2 \rightarrow S_3$, twice as many entities of $S_2$ as entities of $S_1$ are involved each time a reaction event is counted. The value of the expression in the **KineticLaw**'s `math` element describes the *rate* at which the reaction takes place. The product of the reaction rate (of a given reaction) and the stoichiometry (of a given species in the reaction) describes the reaction's contribution to the rate of change of the species' quantity in the overall system.

It is important to make clear that a "kinetic law" in SBML is *not* identical to a traditional rate law. When modeling species as continuous amounts (e.g., concentrations), the rate laws used are traditionally expressed in terms of *concentration per time*. Unfortunately, this approach only works well in cases where certain assumptions hold. Three assumptions in particular are incompatible with generalized multicompartmental modeling; they are listed in Table 5 along with the problems they entail.

***Table 5:*** *Assumptions behind "traditional" rate laws, and the problems they imply for general multicompartmental modeling.*

| **Assumption** | **Problem** |
|---|---|
| All species that participate in a given reaction are located in one compartment | SBML must support reaction processes (e.g., transport) that move species between compartments |
| Compartments are three-dimensional volume containers | SBML must support models where reactions may take place at interfaces (e.g., 2-D membranes) between compartments, thus involving compartments with different dimensions |
| Compartment volumes are constant over time | SBML must support systems with compartments that can change size over time |

A simple example can illustrate the problems that arise when describing reactions between multiple volumes using *concentration*/*time* units (which is to say, *amount*/*volume*/*time*). Suppose we have two species pools $S_1$ and $S_2$, with $S_1$ located in a compartment having volume $V_1$, and $S_2$ located in a compartment having volume $V_2$. Let the volume $V_2 = 3V_1$. Now consider a transport reaction $S_1 \rightarrow S_2$ in which the species $S_1$ is moved from the first compartment to the second. Assume we only want to model the overall effect, without getting into the molecular details (which might in reality involve such things as pores in a membrane between the compartments). Let us use the simplest type of chemical kinetics, in which the rate of the transport reaction is controlled by the activity of $S_1$ and this rate is equal to some constant $k$ times the activity of $S_1$. For the sake of simplicity, assume $S_1$ is in a diluted solution and thus that the activity of $S_1$ can be taken to be equal to its concentration $[S_1]$. The rate expression will therefore be $k \cdot [S_1]$, with $k$ having the unit $1/time$. Then:

$$\frac{d[S_2]}{dt} = -\frac{d[S_1]}{dt} = k \cdot [S_1]$$

So far, this looks normal—until we consider the number of molecules of $S_1$ that disappear from the compartment of volume $V_1$ and appear in the compartment of volume $V_2$. The number of molecules of $S_1$ (call this $n_{S_1}$) is given by $[S_1] \cdot V_1$ and the number of molecules of $S_2$ (call this $n_{S_2}$) is given by $[S_2] \cdot V_2$. Since our volumes have the relationship $V_2/V_1 = 3$, the relationship above implies that $n_{S_1} = k \cdot [S_1] \cdot V_1$ molecules disappear from the first compartment per unit of time and $n_{S_2} = 3 \cdot k \cdot [S_1] \cdot V_1$ molecules appear in the second compartment. In other words, we have created matter out of nothing!

The problem lies in the use of concentrations as the measure of what is transferred by the reaction, because concentrations depend on volumes and the scenario involves multiple unequal volumes. The problem is not limited to using concentrations or volumes; the same problem also exists when using density, i.e., *mass/volume*, as well as dependency on other spatial distributions (i.e., areas or lengths). What must be done instead is to consider the number of "items" being acted upon by a reaction process irrespective of their distribution in space (volume, area or length). An "item" in this context may be a molecule, particle, mass, or other "thing", as long as the substance measurement is independent of the size of the space in which the items are located and the processes take place.

In multicompartmental models, to be able to specify a rate law only once and then use it unmodified in equations for different species, the rate law needs to be expressed in terms of an intensive property, that is, *species quantity/time*, rather than the extensive property typically used, *species quantity/size/time*. As a result, modelers and software tools in general cannot insert traditional textbook rate laws unmodified into the `math` element of a **KineticLaw**. The unusual term "kinetic law" was chosen to alert users to this difference.

#### *Constructing rate-of-change equations for the species*

A consequence of the approach to "kinetic laws" discussed in the previous section is this: when constructing equations describing the time-rates of change of different species defined by an SBML model, the equations are assumed to be in terms of time-rates of changes to *amounts, not concentrations* (or more generally *densities*, i.e., amount per size of compartment). A kinetic law does *not* describe how often a reaction would take place in a compartment of unit size, but rather how often it takes place (per time unit) given the actual size of the compartment. The dimension of the kinetic law is therefore *number of reaction events per time.*

When constructing a system of equations dictating the rates of change of the species in an SBML model, we only need to consider species having attribute values `constant`="`false`" and `boundaryCondition`="`false`", because as discussed in Section 4.6.6 on p. 51, these are the only species affected by the reactions in the model. (Other species not meeting these criteria may be affected by other SBML constructs, but here, we are focusing specifically on the implications of reactions.)

Assume now a model in which $N$ species $S_1, S_2, \ldots, S_N$ having attribute values `constant`="`false`" and `boundaryCondition`="`false`" participate in $M$ reactions $R_1, R_2, \ldots, R_M$. Let $v_{R_j}$ represent the rate or velocity of reaction $R_j$ as given by the formula in the `math` element of **KineticLaw** object for $R_j$. The unit of measurement associated with this rate expression is *extent/time*, where the extent and time units are specified by the `extentUnits` and `timeUnits` attributes on the **Model** object, respectively. Let $\text{stoich}_{S_i,R_j}$ represent the effective stoichiometry of species $S_i$ in reaction $R_j$. (By "effective stoichiometry", we mean the number that results from taking the sum of the stoichiometry values of all references to $S_i$ in $R_j$'s `listOfReactants` and subtracting the sum of the stoichiometric values of all references to $S_i$ in $R_j$'s `listOfProducts`.) If $S_i$ is neither a reactant nor product in some reaction $R_x$, then $\text{stoich}_{S_i,R_x} = 0$. Finally, let $n_{S_i}$ represent the amount of species $S_i$ in the model (and note that this value is *not* a concentration or density).

There are three possible cases to consider when constructing rate-of-change equations for the species:

1. *No conversion factors defined.* If neither the **Species** object for $S_i$ nor the **Model** object define values for their respective `conversionFactor` attributes, then the rate of change of the species amount is determined as follows (and note the implication that the unit of reaction extent should be identical to the unit in which the amount of species $S_i$ is measured):

$$\frac{dn_{S_i}}{dt} = \sum_{j=1}^{M} \text{stoich}_{S_i,R_j} \cdot v_{R_j}$$

2. *Global conversion factor defined.* If the **Model** object instance defines a value for its `conversionFactor` attribute, *and* the **Species** object for $S_i$ does *not* define a value for its `conversionFactor`, then the global conversion factor is used to convert between the unit of reaction extent in the model and the unit in which the amount of species $S_i$ is measured. Let $c_{\text{model}}$ represent the value of the parameter object identified by the conversionFactor attribute value on **Model** (see Section 4.2.6 on p. 39). The formula for the rate of change of $S_i$'s amount then becomes the following:

$$\frac{dn_{S_i}}{dt} = c_{\text{model}} \cdot \sum_{j=1}^{M} \text{stoich}_{S_i,R_j} \cdot v_{R_j}$$

3. *Conversion factor defined for the species.* If the **Species** object instance for $S_i$ defines a value for its conversionFactor attribute, then this factor is used to convert between the unit of reaction extent in the model and the unit in which the amount of species $S_i$ is measured. (The factor defined by the individual species overrides any value that may exist for the **Model** object's conversionFactor.) Let $c_{S_i}$ represent the value of the parameter object identified by $S_i$'s conversionFactor attribute value (see Section 4.6.7 on p. 52). The formula for the rate of change of $S_i$'s amount then becomes the following:

$$\frac{dn_{S_i}}{dt} = c_{S_i} \cdot \sum_{j=1}^{M} \text{stoich}_{S_i,R_j} \cdot v_{R_j}$$

In Section 8.2.4, we present some recommendations for how to encode rate laws and models in SBML.

##### *Species with negative or zero values*

In many models, a **Species** will represent an amount of a physical entity. In those cases, the level of that species should never go negative. Similarly, many reactions represent physical conversion of physical entities. In those cases, if the entities to be converted have reached a level of zero, no conversion can take place. However, in both cases, it is the duty of the modeler to ensure that the **KineticLaw** of the **Reaction** is encoded in such a way that these physical restrictions are obeyed. Other systems and models use the **Species** construct to represent quantities that can go negative, such as charge. Thus, mathematically, if a model dictates that a **Species** level goes negative, or if a **Reaction** process acts on zero- or negative-level **Species**, proper interpretation of that model requires that to happen.

#### 4.11.8 Use of reaction identifiers in mathematical expressions

The value of the id attribute of a **Reaction** can be used as the content of a ci element in MathML formulas elsewhere in the model. Such a ci element or symbol represents the rate of the given reaction as given by the reaction's **KineticLaw** object. As explained above, the unit of measurement associated with the mathematical expression in a **KineticLaw** object is *extent*/*time*; therefore, this this is the unit associated with the id attribute of a **Reaction** when the identifier appears in MathML expressions.

A **KineticLaw** object in effect forms an assignment statement assigning the evaluated value of the math element to the symbol value contained in the **Reaction** id attribute. No other object can assign a value to such a reaction symbol; i.e., the variable or symbol attributes of **InitialAssignment**, **RateRule**, **AssignmentRule** and **EventAssignment** objects cannot contain the value of a **Reaction** id attribute.

The combined set of **InitialAssignment**, **AssignmentRule** and **KineticLaw** objects form a set of assignment statements that should be considered as a whole. The combined set of assignment rules should not contain algebraic loops: a chain of dependency between these statements should terminate. (More formally, consider the directed graph of assignment statements where nodes are statements and directed arcs exist for each occurrence of a symbol in an assignment statement math element. The directed arcs start from the statement defining the symbol to the statements that contain the symbol in their math elements. Such a graph must be acyclic.) Examples of valid and invalid set of assignment statements are given in Section 4.9.5 on p. 64.

### 4.12 Events

**Model** has an optional list of **Event** objects that describe the time and form of instantaneous, discontinuous state changes in the model. For example, an event may describe that a certain species quantity in a model is halved when another species' quantity exceeds a given threshold value.

An SBML **Event** object defines when the event can occur, the variables that are affected by it, how the variables are affected, and the event's relationship to other events. The effect of the event can optionally be delayed after the occurrence of the condition which invokes it. Conceptually, the operation of every event is divided into two phases (even when it is not delayed): the first phase when the event is *triggered* and the second phase when the event is *executed*. The object classes **Event**, **Trigger**, **Delay**, **Priority**, **EventAssignment** and **ListOfEventAssignments** are derived from ***SBase*** (see Section 3.2 on p. 14) and are defined in Figure 20. An example of a model which uses events is given in Section 7 on p. 113.

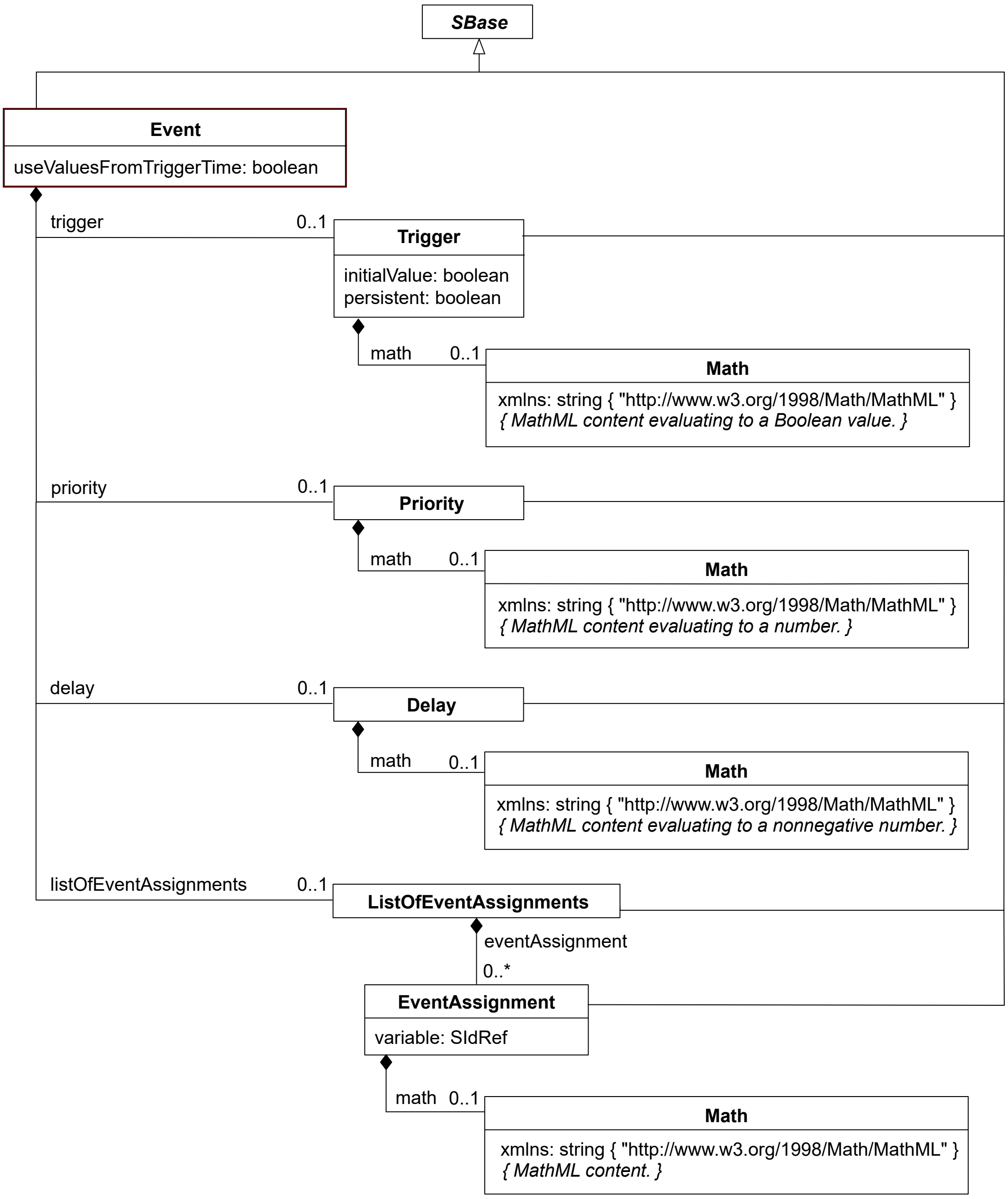

***Figure 20:*** *The definitions of* ***Event****,* ***Trigger****,* ***Delay****,* ***Priority****,* ***EventAssignment****, and* ***ListOfEventAssignments****.*

#### 4.12.1 Event

In addition to the attributes and children it inherits from ***SBase***, an **Event** definition has one required attribute, `useValuesFromTriggerTime`, and five optional subobjects, **Trigger**, **Delay**, **Priority**, **ListOfEventAssignments**, and **EventAssignment**. These various features of **Event** are described below.

##### The `id` attribute

**Event** inherits an optional `id` attribute of type `SId` from ***SBase***. The identifier of an **Event** has no mathematical meaning in an SBML Level 3 Version 2 Core model.

##### The optional `sboTerm` attribute on Event

**Event** inherits an optional `sboTerm` attribute of type `SBOTerm` from ***SBase*** (see Section 3.1.12 on p. 14 and Section 5 on p. 91). When this attribute is present on a given **Event** object instance, its value should be an SBO identifier belonging to the branch for type **Event** indicated in Table 6 on p. 98. The relationship is of the form "the event *is-a* X", where X is the SBO term. The term chosen should be the most precise (narrow) one that captures the role of the event in the model.

##### The `useValuesFromTriggerTime` attribute

The possibility of defining an optional **Delay** within **Event**, and the potential for multiple simultaneously-triggered events, means there are two times to consider when interpreting an event: the moment at which the event *triggered*, and the moment at which its assignments are *executed*. (If a **Delay** subobject is present, these moments are separated by simulation time. If multiple events are triggered simultaneously, these moments are separated by the sequential execution of the event assignments.) Similarly, it is also possible to distinguish between the moment at which the mathematical expression of an **EventAssignment** object is evaluated, and the moment at which this value is assigned to the entity referenced by the **EventAssignment**'s `variable` attribute. A model could intend the **EventAssignment** expression to be evaluated either at the moment the event is triggered, or at the moment the event assignments are executed. (In the former case, a model interpreter would have to save the calculated values until the moment of execution.)

The attribute `useValuesFromTriggerTime` allows a model to indicate the moment at which the event's assignments are to be evaluated. A value of "`true`" indicates the values assignments are to be computed at the moment the event is *triggered*. Conversely, `useValuesFromTriggerTime`="`false`" means the assignments are to be computed at the moment the event is *executed*. The attribute has no default value.

#### 4.12.2 Trigger

As shown in Figure 20 on the preceding page, an **Event** object may contain exactly one object of class **Trigger**. This object in turn must contain two attributes, `persistent` and `initialValue`, and may contain a MathML `math` element. The expression in the `math` element will be interpreted as a value of type `boolean`. The exact moment at which this expression evaluates to "`true`" during a simulation is taken to be the time point when the **Event** is *triggered*.

An event only triggers when the expression within its **Trigger** object makes the transition in value from "`false`" to "`true`". The event will trigger again at any subsequent moments when the trigger makes the transition from "`false`" to "`true`"; in other words, an event can trigger multiple times during a simulation if its trigger condition makes the transition from "`false`" to "`true`" more than once. The behavior at the very start of simulation time (i.e., $t = 0$, where $t$ stands for time) is determined in part by the `boolean` flag `initialValue`, discussed below.

If an instance of an **Event** contains no **Trigger** child, that **Event** has no way of being triggered or executed in a model according to the SBML Level 3 Version 2 Core specification. The **Event** may nevertheless be present in a model as part of (e.g.) a partially unknown system, for annotation purposes, or as part of constructs defined by an SBML Level 3 package.

A **Trigger** with no `math` child leaves undefined when the corresponding **Event** will trigger. The absence of a `math` element is permitted because it is possible for SBML Level 3 packages to add constructs that extend **Trigger** and define how a value is to be computed. In the absence of such constructs, the event is never triggered. A simulator encountering this situation may choose to produce a warning.

##### *The* `id` *attribute on* ***Trigger***

**Trigger** inherits an optional `id` attribute from ***SBase***, of type `SId`. The identifier has no mathematical meaning in an SBML Level 3 Version 2 Core model.

##### *The* `persistent` *attribute on* ***Trigger***

In the interval between when an **Event** object *triggers* (i.e., its **Trigger** object expression transitions in value from "`false`" to "`true`") and when its assignments are to be *executed*, conditions in the model may change such that the trigger expression transitions back from "`true`" to "`false`". Should the event's assignments still be made if this happens? Answering this question is the purpose of the `persistent` attribute on **Trigger**.

If the `boolean` attribute `persistent` has a value of "`true`", then once the event is triggered, all of its assignments are always performed when the time of execution is reached. The name "persistent" is meant to evoke the idea that the trigger expression does not have to be re-checked after it triggers if `persistent`="`true`". Conversely, if the attribute value is "`false`", then the trigger expression is not assumed to persist: if the expression transitions in value back to "`false`" at any time between when the event triggered and when it is to be executed, the event is no longer considered to have triggered and its assignments are not executed. (If the trigger expression transitions once more to "`true`" after that point, then the event is triggered, but this then constitutes a whole new event trigger-and-execute sequence.)

The `persistent` attribute can be especially useful when **Event** objects contain **Delay** objects, but it is relevant even in a model without delays if the model contains two or more events. As explained in the introduction to this section, the operation of all events in SBML (delayed or not) is conceptually divided into two phases, *triggering* and *execution*; however, unless events have priorities associated with them (see Section 4.12.3 on the next page), SBML does not mandate a particular ordering of event execution in the case of simultaneous events (see Section 4.12.7 on p. 89). Models with multiple events can lead to situations where the execution of one event affects another event's trigger expression value. If that other event has `persistent`="`false`", and its trigger expression evaluates to "`false`" before it is to be executed, the event must not be executed after all.

##### *The* `initialValue` *attribute on* ***Trigger***

As mentioned above, an event *triggers* when the mathematical expression in its **Trigger** object transitions in value from "`false`" to "`true`". An unanswered question concerns what happens at the start of a simulation: can event triggers make this transition at $t = 0$, where $t$ stands for time?

In order to determine whether an event may trigger at $t = 0$, it is necessary to know what value the **Trigger** object's `math` expression had immediately prior to $t = 0$. This starting value of the trigger expression is determined by the value of the `boolean` attribute `initialValue`. A value of "`true`" means the trigger expression is taken to have the value "`true`" immediately prior to $t = 0$. In that case, the trigger cannot transition in value from "`false`" to "`true`" at the moment simulation begins (because it has the value "`true`" both before and after $t = 0$), and can only make the transition from "`false`" to "`true`" sometime *after* $t = 0$. (To do that, it would also first have to transition to "`false`" before it could make the transition from "`false`" back to "`true`".) Conversely, if `initialValue`="`false`", then the trigger expression is assumed to start with the value "`false`", and therefore may trigger at $t = 0$ if the expression evaluates to "`true`" at that moment.

##### *The optional* `sboTerm` *attribute on* ***Trigger***

**Trigger** inherits an optional `sboTerm` attribute of type `SBOTerm` from its parent class ***SBase*** (see Section 3.1.12 and Section 5). The value given to this attribute should be an SBO identifier belonging to the branch for type **Trigger** indicated in Table 6 on p. 98. The relationship is of the form "the trigger *is-a* X", where X is the SBO term. The term chosen should be the most precise (narrow) one that captures the role of the trigger in the model.

#### 4.12.3 Priority

As shown in Figure 20 on p. 80, an **Event** object can contain an optional **Priority** subobject. The **Priority** object class, like **Delay**, is derived from ***SBase*** and contains a MathML formula stored in the element `math`. This formula is used to compute a dimensionless numerical value that influences the order in which a simulator is to perform the assignments of two or more events that happen to be executed simultaneously. The formula may evaluate to any `double` value (and thus may be a positive or negative number, or zero), with positive numbers taken to signifying a higher priority than zero or negative numbers. If no **Priority** object is present on a given **Event** object, no priority is defined for that event.

A **Priority** with no `math` child leaves the priority of the event undefined. The absence of a `math` element is permitted because it is possible for SBML Level 3 packages to add constructs that extend **Priority** and define how a value is to be computed. In the absence of such constructs, the event behaves as if it does not have a priority. A simulator encountering this situation may choose to produce a warning.

##### *The interpretation of priorities on events in a model*

For the purposes of SBML, *simultaneous event execution* is defined as the situation in which multiple events have identical times of execution. The time of execution is calculated as the sum of the time at which a given event's **Trigger** is *triggered* plus its **Delay** duration, if any. Here, "identical times" means *mathematically equal* instants in time. (In practice, simulation software adhering to this specification may have to rely on numerical equality instead of strict mathematical equality; robust models will ensure that this difference will not cause significant discrepancies from expected behavior.)

If no **Priority** subobjects are defined for two or more **Event** objects, then those events are still executed simultaneously but their order of execution is *undefined by this SBML specification*. A software implementation may choose to execute such simultaneous events in any order, as long as each event is executed only once and the requirements of checking the `persistent` attribute (and acting accordingly) are satisfied. See Section 4.12.2 on the preceding page for more information about the attribute `persistent`.

If **Priority** subobjects are defined for two or more simultaneously-triggered events, the order in which those particular events must be executed is dictated by their **Priority** objects, as follows. If the values calculated using the two **Priority** objects' `math` expressions differ, then the event having the higher priority value must be executed before the event with the lower value. If, instead, the two priority values are mathematically equal, then the two events must be triggered in a *random* order. It is important to note that a *random order is not the same as an undefined order*: given multiple runs of the same model with identical conditions, an undefined ordering would permit a system to execute the events in (for example) the same order every time (according to whatever scheme may have been implemented by the system), whereas the explicit requirement for random ordering means that the order of execution in different simulation runs depends on random chance. In other words, given two events "A" and "B", a randomly-determined order must lead to an equal chance of executing "A" first or "B" first, every time those two events are executed simultaneously.

A model may contain a mixture of events, some of which have **Priority** subobjects and some do not. Should a combination of simultaneous events arise in which some events have priorities defined and others do not, the set of events with defined priorities must trigger in the order determined by their **Priority** objects, and the set of events without **Priority** objects must be executed in an *undefined* order with respect to each other and with respect to the events with **Priority** subobjects. (Note that *undefined order* does not necessarily mean random order, although a random ordering would be a valid implementation of this requirement.)

The following example may help further clarify these points. Suppose a model contains four events that should be executed simultaneously, with two of the events having **Priority** objects with the same value and the other two events having **Priority** objects with the same, but different, value. The two events with the higher priorities must be executed first, in a random order with respect to each other, and the remaining two events must be executed after them, again in a random order, for a total of four possible and equally-likely event executions: A-B-C-D, A-B-D-C, B-A-C-D, and B-A-D-C. If, instead, the model contains four events all having the same **Priority** values, there are 4! or 24 possible orderings, each of which must be equally likely to be chosen. Finally, if none of the four events has a **Priority** subobject defined, or even if exactly one of the four events has a defined **Priority**, there are again 24 possible orderings, but the likelihood of choosing any particular ordering is undefined; the simulator can choose between events as it wishes. (The SBML specification only defines the effects of priorities on **Event** objects with respect to *other* **Event** objects with priorities. Putting a priority on a *single* **Event** object in a model does not cause it to fall within that scope.)

Section 4.12.7 on p. 89 includes additional discussion of these topics.

##### *Evaluation of **Priority** expressions*

An event's **Priority** object `math` expression must be evaluated at the time the **Event** is to be *executed*. During a simulation, all simultaneous events have their **Priority** values calculated, and the event with the highest priority is selected for next execution. Note that it is possible for the execution of one **Event** object to cause the **Priority** value of another simultaneously-executing **Event** object to change (as well as to trigger other events, as already noted). Thus, after executing one event, and checking whether any other events in the model have been triggered, all remaining simultaneous events that *either* (i) have **Trigger** objects with attributes `persistent`="`false`" *or* (ii) have **Trigger** expressions that did not transition from "`true`" to "`false`", must have their **Priority** expression reevaluated. The highest-priority remaining event must then be selected for execution next. Section 8.2.5 on p. 153 provides further discussion about implementing support for events.

##### *Units of **Priority** object's mathematical expressions*

The unit associated with the value of a **Priority** object's `math` expression should be `dimensionless`. This is because the priority expression only serves to provide a relative ordering between different events, and only has meaning with respect to other **Priority** object expressions. The value of **Priority** objects is not comparable to any other kind of object in an SBML model.

##### *The `id` attribute on **Priority***

**Priority** inherits an optional `id` attribute from ***SBase***, of type `SId`. The identifier has no mathematical meaning in an SBML Level 3 Version 2 Core model.

##### *The optional `sboTerm` attribute on **Priority***

**Priority** inherits an optional `sboTerm` attribute of type `SBOTerm` from its parent class ***SBase*** (see Section 3.1.12 and Section 5). When a value is given to this attribute in a **Priority** instance, it should be an SBO identifier belonging to the branch for type **Priority** indicated in Table 6 on p. 98. The relationship is of the form "the priority *is-a* X", where X is the SBO term. The term chosen should be the most precise (narrow) one that captures the role of the priority in the model.

#### *4.12.4 Delay*

As shown in Figure 20 on p. 80, an **Event** object can contain an optional **Delay** object. The **Delay** class is derived from ***SBase*** and contains a mathematical formula stored in `math`. The formula is used to compute the length of time between when the event has *triggered* and when the event's assignments (see below) are actually *executed*. If no delay is present on a given **Event**, no delay is defined for that event.

Similarly, a **Delay** with no `math` child leaves the delay of the event undefined. The absence of a `math` element is permitted because it is possible for SBML Level 3 packages to add constructs that extend **Delay** and define how a value is to be computed. In the absence of such constructs, the event is considered to execute as if it had no **Delay**. A simulator encountering this situation may choose to produce a warning.

The expression in the **Delay** object's `math` element must be evaluated at the time the event is *triggered*. The expression must always evaluate to a nonnegative number (otherwise, a nonsensical situation could arise where an event is defined to execute before it is triggered!).

##### *Units of delay expressions*

The unit associated with the value of a **Delay** object's `math` expression should match the model's unit of *time* (see Section 4.2.3 on p. 38). Note that, as in other cases of MathML expressions in SBML, units are *not* automatically predefined or assumed. As discussed in Section 3.4.12 on p. 31, expressions containing only literal numbers and/or **Parameter** objects without declared units are considered to have unspecified units. In such cases, the correspondence between the needed entity units and the (unknown) unit for the **Delay**'s `math` expression cannot be proven, and while such expressions are not considered inconsistent, all that can be assumed by model interpreters (whether software or human) is that the units *may* be consistent.

The following **Event** example fragment helps illustrate this:

```
<model timeUnits="second" ...>
    ...
    <listOfEvents>
        <event useValuesFromTriggerTime="true">
            ...
            <delay>
                <math xmlns="http://www.w3.org/1998/Math/MathML">
                    <cn> 10 </cn>
                </math>
            </delay>
            ...
        </event>
    </listOfEvents>
    ...
</model>
```

Note that the "<cn> 10 </cn>" within the mathematical formula has no specified unit attached to it. The model is not invalid because of this, but a recipient of the model may justifiably be concerned about what "10" really means. (Ten seconds? What if the global unit of time on the model were changed from seconds to milliseconds? Would the modeler remember to change "10" to "10 000"?) A better approach would be to declare the unit explicitly, as in the following example:

```
<model timeUnits="second" ...>
    ...
    <listOfEvents>
        <event useValuesFromTriggerTime="true">
            ...
            <delay>
                <math xmlns="http://www.w3.org/1998/Math/MathML"
                      xmlns:sbml="http://www.sbml.org/sbml/level3/version2/core">
                    <cn sbml:units="second"> 10 </cn>
                </math>
            </delay>
            ...
        </event>
    </listOfEvents>
    ...
</model>
```

While this approach will not solve the problem of updating the value if the model's global of unit of time is changed, it will at least inform readers of the intended duration of the delay itself as well as make it possible for software tools to potentially detect unit inconsistencies if the tools can perform unit analysis.

Another, different approach is to define a global **Parameter** object for the time delay (with an appropriate unit attached), and to replace the `cn` element above with a `ci` element containing the parameter's identifier. This has advantages because **Parameter** objects can have annotations and SBO terms associated with them.

*The* `id` *attribute on* ***Delay***

**Delay** inherits an optional `id` attribute from ***SBase***, of type `SId`. The identifier has no mathematical meaning in an SBML Level 3 Version 2 Core model.

*The optional* `sboTerm` *attribute on* ***Delay***

**Delay** inherits an optional `sboTerm` attribute of type `SBOTerm` from its parent class ***SBase*** (see Section 3.1.12 and Section 5). When a value is given to this attribute in a **Delay** instance, it should be an SBO identifier belonging to the branch for type **Delay** indicated in Table 6 on p. 98. The relationship is of the form "the delay *is-a* X", where X is the SBO term. The term chosen should be the most precise (narrow) one that captures the role of the delay in the model.

#### 4.12.5 EventAssignment

**Event** contains an optional element called `listOfEventAssignments`, of class **ListOfEventAssignments**. In every instance of an event definition in a model, the object's `listOfEventAssignments` element may have zero or more `eventAssignment` elements of class **EventAssignment**. The object class **EventAssignment** has one required attribute, `variable`, and the optional element, `math`. Being derived from ***SBase***, it also has all the usual attributes and elements of its parent class.

An "event assignment" has effect when the event is *executed*. (As noted above, the operation of event is divided conceptually into two phases: the first phase when the event is *triggered* and the second phase when the event is *executed*.) See Section 4.12.7 on p. 89 below for more information about events and event assignments.

*The* `variable` *attribute*

The **EventAssignment** required attribute `variable` has type `SIdRef`. The value of the attribute must be the identifier of an object in the `SId` namespace of the model with mathematical meaning and the ability to be assigned. In SBML Level 3 Core, the permitted objects classes are **Compartment**, **Species**, **SpeciesReference**, and **Parameter**. The set also includes SBML Level 3 package objects with identifiers in the `SId` namespace of the model and mathematical meaning defined to allow assignment.

When the event is executed, the value of the model component identified by `variable` is changed by the **EventAssignment** to the value computed by the `math` element. For components defined by SBML Level 3 Core, this means that a species' quantity, species reference's stoichiometry, compartment's size or parameter's value are reset to the value computed by `math`.

Certain restrictions are placed on what can appear in `variable`:

- The object identified by the value of the `variable` attribute must not have its `constant` attribute set to "`true`". (Constants cannot be affected by events.)
- The `variable` attribute must not contain the identifier of a reaction; in core, only species, species references, compartment and parameter values may be set by an **Event**.
- The value of every `variable` attribute must be unique among the set of **EventAssignment** objects within a given **Event** instance. In other words, a single event cannot have multiple **EventAssignment** children assigning the same variable. (All of them would be performed at the same time, when that particular **Event** triggers, resulting in indeterminacy.) Separate **Event** instances can refer to the same variable.
- A variable cannot be assigned a value in an **EventAssignment** object instance and also be assigned a value by an **AssignmentRule**, i.e., the value of the `variable` attribute in an **EventAssignment** instance cannot be the same as the value of a `variable` attribute in an **AssignmentRule** instance. (Assignment rules hold at all times, therefore it would be inconsistent to also define an event that reassigns the value of the same variable.)

If the variable attribute of an **EventAssignment** object references an object in an SBML namespace that is not recognized by the interpreter reading a given SBML document (that is, if the object is defined by an SBML Level 3 package that the software does not support), the event assignment must be ignored—the object's value must not be assigned if the interpreter cannot understand the package. If an interpreter cannot establish whether a referenced object is missing from the model or instead is defined in an SBML namespace not recognized by the interpreter, it may produce a warning to the user. (The latter situation can only arise if an SBML package is present in the SBML document with a package:required attribute of "true".)

Note that the time of assignment of the object identified by the value of variable is always the time at which the **Event** is *executed*, not when it is *triggered*. The timing is controlled by the optional **Delay**. The time of assignment is not affected by the **Event** attribute useValuesFromTriggerTime—that attribute affects the time at which the **EventAssignment**'s math expression is evaluated. In other words, SBML allows decoupling the time at which the variable is assigned from the time at which its value expression is calculated.

#### *The optional* sboTerm *attribute on* ***EventAssignment***

**EventAssignment** inherits an optional sboTerm attribute of type SBOTerm from its parent class ***SBase*** (see Section 3.1.12 on p. 14 and Section 5 on p. 91). When a value is given to this attribute in a **EventAssignment** instance, it should be an SBO identifier belonging to the branch for type **EventAssignment** indicated in Table 6 on p. 98. The relationship is of the form "the event assignment *is-a* X", where X is the SBO term. The term chosen should be the most precise (narrow) one that captures the role of the event assignment in the model.

#### *The optional* id *attribute on* ***EventAssignment***

**EventAssignment** inherits an optional id attribute from ***SBase***, of type SId. The identifier has no mathematical meaning in an SBML Level 3 Version 2 Core model.

#### ***EventAssignment****'s* math

The math element contains a MathML expression that defines the new value to be given to the object identified by the **EventAssignment** attribute variable.

As mentioned above, the time at which the expression in math is evaluated is determined by the attribute useValuesFromTriggerTime on **Event**. If the attribute value is "true", the expression must be evaluated when the event is *triggered*; more precisely, the values of identifiers occurring in MathML ci elements in the **EventAssignment**'s math expression are the values they have at the point when the event *triggered*. If, instead, useValuesFromTriggerTime's value is "false", it means the values at *execution* time should be used; that is, the values of identifiers occurring in MathML ci attributes in the **EventAssignment**'s math expression are the values they have at the point when the event *executed*.

An **EventAssignment** with no math child leaves undefined what assignment is to be made to the corresponding symbol. The absence of a math element is permitted because it is possible for SBML Level 3 packages to add constructs that extend **EventAssignment** and define how a value is to be computed. In the absence of any such construct, no assignment is carried out when there is no math element. This leaves the model unchanged: any element that had a value will continue to have that value; any element whose value was undefined will continue to have its value undefined. A simulator encountering this situation may choose to produce a warning. No other validation rules are affected by the absence of a math child: it is still invalid to have an **EventAssignment** and an **AssignmentRule** that assign to the same model element, for example.

#### *Units of the* math *formula in* ***EventAssignment***

In all cases, as would be expected, the unit of measurement associated with value of the formula contained in the math element of an **EventAssignment** object should be consistent with the unit associated with the object identified by the variable attribute value. More precisely, for objects defined by SBML Level 3 Core:

- *In the case of a species*, an **EventAssignment** sets the referenced species' quantity (*concentration* or *amount*) to the value determined by the formula in `math`. The unit associated with the value produced by the `math` formula should be equal to the unit associated with the species' quantity. (See Section 4.6.5 on p. 51 for an explanation of how a species' quantity is determined.)

- *In the case of a species reference*, an **EventAssignment** sets the stoichiometry of the reactant or product referenced by the **SpeciesReference** object to the value determined by the formula in `math`. The unit associated with the value produced by the `math` formula should be `dimensionless`, because reactant and product stoichiometries in reactions are dimensionless quantities.

- *In the case of a compartment*, an **EventAssignment** sets the referenced compartment's size to the size determined by the formula in `math`. The unit associated with the value produced by the `math` formula should be the same as that specified for the compartment's size. (See Section 4.5.4 on p. 48 for more.)

- *In the case of a parameter*, an **EventAssignment** sets the parameter's value to the value of the formula in `math`. The unit associated with the value produced by the `math` formula should be the same as parameter's `units` attribute value. (Section 4.7.3 on p. 55 for more information about parameter units.)

- *In the case of an object from an SBML Level 3 package*, an **EventAssignment** sets the referenced object's value (as defined by that package) to the value of the formula in `math`. The unit of measurement associated with the value produced by the formula should be the same as that object's `units` attribute value (if it has such an attribute), or be equal to the units of model components of that type (if objects of that class are defined by the package as having the same units).

Note that the formula in `math` has no assumed unit of measurement associated with it. The consistency of the units between the formula and the entity affected by the assignment should be established explicitly.

#### 4.12.6 Example Event definitions

The following is an example of an event. This structure makes the assignment $k_2 = 0$ when $P_1 \leq P_2$:

```
<model>
    ...
    <listOfUnitDefinitions>
        <unitDefinition id="per_second">
            <listOfUnits>
                <unit kind="second" exponent="-1" multiplier="1" scale="0"/>
            </listOfUnits>
        </unitDefinition>
    </listOfUnitDefinitions>
    ...
    <listOfParameters>
        <parameter id="k2" value="0.05" units="per_second" constant="false"/>
        <parameter id="k2reset" value="0.0" units="per_second" constant="true"/>
    </listOfParameters>
    ...
    <listOfEvents>
        <event useValuesFromTriggerTime="true">
            <trigger initialValue="false" persistent="true">
                <math xmlns="http://www.w3.org/1998/Math/MathML">
                    <apply> <leq/> <ci> P_1 </ci> <ci> P_2 </ci> </apply>
                </math>
            </trigger>
            <listOfEventAssignments>
                <eventAssignment variable="k2">
                    <math xmlns="http://www.w3.org/1998/Math/MathML">
                        <ci> k2reset </ci>
                    </math>
                </eventAssignment>
            <listOfEventAssignments>
        </event>
    </listOfEvents>
</model>
```

A complete example of a model using events is given in Section 7.11 on p. 135.

#### 4.12.7 Detailed semantics of events

Any transition of a **Trigger** object's `math` formula from the value "`false`" to "`true`" will cause the enclosing **Event** object to *trigger*. Such a transition is not possible at the very start of a simulation (i.e., at time $t = 0$) unless the **Trigger** object's `initialValue` attribute has a value of "`false`"; this defines the value of the trigger formula to be "`false`" immediately prior to the start of simulation, thereby giving it the potential to change in value from "`false`" to "`true`" when the formula is evaluated at $t = 0$. If `initialValue`="`true`", then the trigger expression cannot transition from "`false`" to "`true`" at $t = 0$ but may do so at some time $t > 0$.

Consider an **Event** object definition $E$ with delay $d$ in which the **Trigger** object's `math` formula makes a transition in value from "`false`" to "`true`" at times $t_1$ and $t_2$. The **EventAssignment** within the **Event** object will have effect at $t_1 + d$ and $t_2 + d$ irrespective of the relative times of $t_1$ and $t_2$. For example, events can "overlap" so that $t_1 < t_2 < t_1 + d$ still causes an event assignments to occur at $t_1 + d$ and $t_2 + d$.

It is entirely possible for two events to be executed simultaneously, and it is possible for events to trigger other events (i.e., an event assignment can cause an event to trigger). This leads to several points:

- A software package should retest all event triggers after executing the assignments from a given event in order to account for the possibility that the assignments cause another event trigger to transition from "`false`" to "`true`". This check should be made after each individual **Event** object's execution, even when several events are to be executed simultaneously.

- Any **Event** object whose **Trigger** `persistent` attribute has the value "`false`" must have its trigger expression reevaluated continuously between when the event has been triggered and when it is executed. If its trigger expression ever evaluates to "`false`", it must be removed from the queue of events pending execution and treated as any other event whose trigger expression evaluates to "`false`".

- Although the precise time at which events are executed is not resolved beyond the given execution point in simulated time, it is assumed that the order in which the events occur *is* resolved. This order can be significant in determining the overall outcome of a given simulation. When an event $X$ *triggers* another event $Y$ and event $Y$ has zero delay, then event $Y$ is added to the existing set of simultaneous events that are pending *execution*. Events $X$ and $Y$ form a cascade of events at the same point in simulation time. An event such as $Y$ may have a special priority if it contains a **Priority** subobject.

- All events in a model are open to being in a cascade. The position of an event in the event queue does not affect whether it can be in the cascade: event $Y$ can be triggered whether it is before or after $X$ in the queue of events pending execution. A cascade of events can be potentially infinite (never terminate); when this occurs a simulator should indicate this has occurred—it is incorrect for a simulator to break a cascade arbitrarily and continue the simulation without at least indicating that the infinite cascade occurred.

- Simultaneous events having no defined priorities are executed in an undefined order. This does not mean that the behavior of the simulation is completely undefined; merely that the *order* of execution of these particular events is undefined. A given simulator may use any algorithm to choose an order as long as every event is executed exactly once. (See also Section 4.12.3 on p. 83.)

- Events with defined priorities are executed in the order implied by their **Priority** `math` formula values, with events having higher priorities being executed ahead of events with lower priorities, and events with identical priorities being executed in a random order with respect to one another (as determined at run-time by some random algorithm equivalent to coin-flipping). Newly-triggered events that are to be executed immediately (i.e., if they define no delays) should be inserted into the queue of events pending execution according to their priorities: events with higher priority values value must be inserted ahead of events with lower priority values and after any pending events with even higher priorities, and inserted randomly among pending events with the same priority values. Events without **Priority** objects must be inserted into the queue in some fashion, but the algorithm used to place it in the queue is undefined. Similarly, there is no restriction on the order of a newly-inserted event with a defined **Priority** with respect to any other pending **Event** without a defined **Priority**. (See Section 4.12.3 on p. 83.)

- A model variable that is the target of one or more event assignments can change more than once when simultaneous events are processed at some time point $t$. The model's behavior (output) for such a variable is the value of the variable at the end of processing all the simultaneous events at time $t$.

## 5 The Systems Biology Ontology and the sboTerm attribute

The values of id attributes on SBML components allow the components to be cross-referenced within a model. The values of name attributes on SBML components provide the opportunity to assign them meaningful labels suitable for display to humans (Section 3.3 on p. 18). The specific identifiers and labels used in a model necessarily must be unrestricted by SBML, so that software and users are free to pick whatever they need. However, this freedom makes it more difficult for software tools to determine, without additional human intervention, the semantics of models more precisely than the semantics provided by the SBML object classes defined in other sections of this document. For example, there is nothing inherent in a parameter with identifier "k" that would indicate to a software tool it is a first-order rate constant (if that's what "k" happened to be in some given model). However, one may need to convert a model between different representations (e.g., Henri-Michaelis-Menten vs. elementary steps), or to use it with different modeling approaches (discrete or continuous). One may also need to relate the model components with other description formats such as SBGN (http://www.sbgn.org/) using deeper semantics. Although an advanced software tool *might* be able to deduce the semantics of some model components through detailed analysis of the kinetic rate expressions and other parts of the model, this quickly becomes infeasible for any but the simplest of models.

An approach to solving this problem is to associate model components with terms from carefully curated controlled vocabularies (CVs). This is the purpose of the optional sboTerm attribute provided on the SBML class ***SBase***. The sboTerm attribute always refers to terms belonging to the Systems Biology Ontology (SBO, (Courtot et al., 2011)). In this section, we discuss the sboTerm attribute, SBO, the motivations and theory behind their introduction, and guidelines for their use.

SBO is not part of SBML; it is being developed separately, to allow the modeling community to evolve the ontology independently of SBML. However, the terms in the ontology are being designed keeping SBML components in mind, and are classified into subsets that can be directly related with SBML components such as reaction rate expressions, parameters, and a few others, see below. The use of sboTerm attributes is optional, and the presence of sboTerm on an element does not change the way the model is *interpreted*. Annotating SBML elements with SBO terms adds additional semantic information that may be used to *convert* the model into another model, or another format. Although SBO support provides an important source of information to understand the meaning of a model, software does not need to support sboTerm to be considered SBML-compliant.

### 5.1 Principles

Labeling model components with terms from shared controlled vocabularies allows a software tool to identify each component using identifiers that are not tool-specific. An example of where this is useful is the desire by many software developers to provide users with meaningful names for reaction rate equations. Software tools with editing interfaces frequently provide these names in menus or lists of choices for users. However, without a standardized set of names or identifiers shared between developers, a given software package cannot reliably interpret the names or identifiers of reactions used in models written by other tools.

The first solution that might come to mind is to stipulate that certain common reactions always have the same name (e.g., "Michaelis-Menten"), but this is simply impossible to do: not only do humans often disagree on the names themselves, but it would not allow for correction of errors or updates to the list of predefined names except by issuing new releases of the SBML specification—to say nothing of many other limitations with this approach. Moreover, the parameters and variables that appear in rate expressions also need to be identified in a way that software tools can interpret mechanically, implying that the names of these entities would also need to be regulated.

The Systems Biology Ontology provides terms for identifying most elements of SBML. The relationship implied by an sboTerm on an SBML model component is "is-a" between the characteristic of the component meant to be described by SBO on this element and the SBO term identified by the value of the sboTerm. By adding SBO term references on the components of a model, a software tool can provide additional details using shared vocabularies that can enable *other* software tools to recognize precisely what the component is meant to be. Those tools can then act on that information. For example, if the SBO identifier SBO:0000049 is assigned to the concept of "first-order irreversible mass-action kinetics, continuous framework", and a given **KineticLaw** object in a model has an `sboTerm` attribute with this value, then regardless of the identifier and name given to the reaction itself, a software tool could use this to inform users that the reaction is a first-order irreversible mass-action reaction. This kind of reverse engineering of the meaning of reactions in a model would be difficult to do otherwise, especially for more complex reaction types.

The presence of SBO labels on **Compartment**, **Species**, and **Reaction** objects in SBML can help map those entities to equivalent concepts in other standards, such as (but not limited to) BioPAX (`https://www.biopax.org/`), PSI-MI (`http://www.psidev.info/index.php?q=node/60`), or the Systems Biology Graphical Notation (SBGN, `http://www.sbgn.org/`). Such mappings can be used in conversion procedures, or to build interfaces, with SBO becoming a kind of "glue" between standards of representation.

The presence of the label on a kinetic expression can also allow software tools to make more intelligent decisions about reaction rate expressions. For example, an application could recognize certain types of reaction formulas as being ones it knows how to solve with optimized procedures. The application could then use internal, optimized code implementing the rate formula indexed by identifiers such as `SBO:0000049` ("mass action rate law for first order irreversible reactions, continuous scheme") appearing in SBML models.

Finally, SBO labels may be very valuable when it comes to model integration, by helping identify interfaces, convert mathematical expressions and parameters etc.

Although the use of SBO can be beneficial, it is critical to keep in mind that the presence of an `sboTerm` value on an object *must not change the fundamental mathematical meaning* of the model. An SBML model must be defined such that it stands on its own and does not depend on additional information added by SBO terms for a correct mathematical interpretation. SBO term definitions will not imply any alternative mathematical semantics for any SBML object labeled with that term. Two important reasons motivate this principle. First, it would be too limiting to require all software tools to be able to understand the SBO vocabularies in addition to understanding SBML. Supporting SBO is not only additional work for the software developer; for some kinds of applications, it may not make sense. If SBO terms on a model are optional, it follows that the SBML model *must* remain unambiguous and fully interpretable without them, because an application reading the model may ignore the terms. Second, we believe allowing the use of `sboTerm` to alter the mathematical meaning of a model would allow too much leeway to shoehorn inconsistent concepts into SBML objects, ultimately reducing the interoperability of the models.

### 5.2 Using SBO and `sboTerm`

The `sboTerm` attribute data type is always `SBOTerm`, defined in Section 3.1.12 on p. 14. When present in a given model object instance, the attribute's value must be an identifier that refers to a single SBO term that best defines the entity encoded by the SBML object in question. An example of the type of relationship intended is: *the KineticLaw in reaction R1 is a first-order irreversible mass action rate law.*

Note the careful use of the words "defines" and "entity encoded by the SBML object" in the paragraph above. As mentioned, the relationship between the SBML object and the URI is:

> The "thing" encoded by this SBML object has a characteristic that is an instance of the "thing" represented by the referenced SBO term.

The characteristic relevant for each SBML object is described in the second column of Table 6 on p. 98.

#### *5.2.1 The structure of the Systems Biology Ontology*

The goal of SBO labeling for SBML is to clarify to the fullest possible extent the nature of each element in a model. The approach taken in the Systems Biology Ontology begins with a hierarchically-structured set of controlled vocabularies with seven main divisions: (1) physical entity representation, (2) participant role, (3) systems description parameter, (4) modeling framework, (5) mathematical expression, (6) occurring entity representation, and (7) metadata representation. Figure 21 on the following page illustrates the highest level of SBO.

Each of the seven branches of Figure 21 on the next page has a hierarchy of terms underneath them. At this time, we can only begin to list some initial concepts and terms in SBO; what follows is not meant to be complete, comprehensive or even necessarily consistent with future versions of SBO. It reflects SBO as it existed in December, 2015. The web site for SBO (`http://biomodels.net/sbo/`) should be consulted for the current version of the ontology. Section 5.4.1 on p. 98 describes how the impact of SBO changes on software applications is minimized.

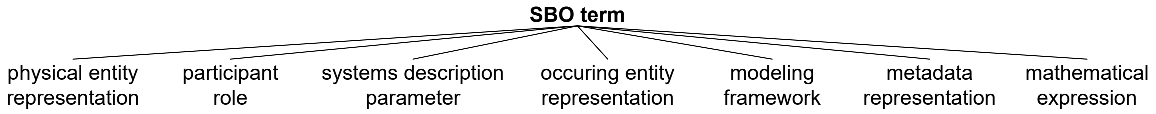

***Figure 21:*** *The seven controlled vocabularies (CVs) that make up the main branches of SBO.*

Figure 22 shows the structure for the *physical entity representation* branch, which reflects the hierarchical groupings of the types of entities that can be represented by a **Compartment** or **Species** object. Note that the values taken by the `sboTerm` attribute on those elements should refer to SBO terms belonging to the *material entity* branch, so as to distinguish whether the element represents a macromolecule, a simple chemical, etc. Indeed, this information remains valid for the whole model. The term should not belong to the *functional entity* branch, representing the function of the entity within a certain functional context. If one wants to use this information, one should refer to the SBO terms using a controlled RDF annotation instead (Section 6 on p. 100), carefully choosing the qualifiers (Section 6.5 on p. 103) to reflect the fact that a given **Species** object, for instance, can fulfill different functions within a given model (e.g., EGF receptor is a receptor and an enzyme).

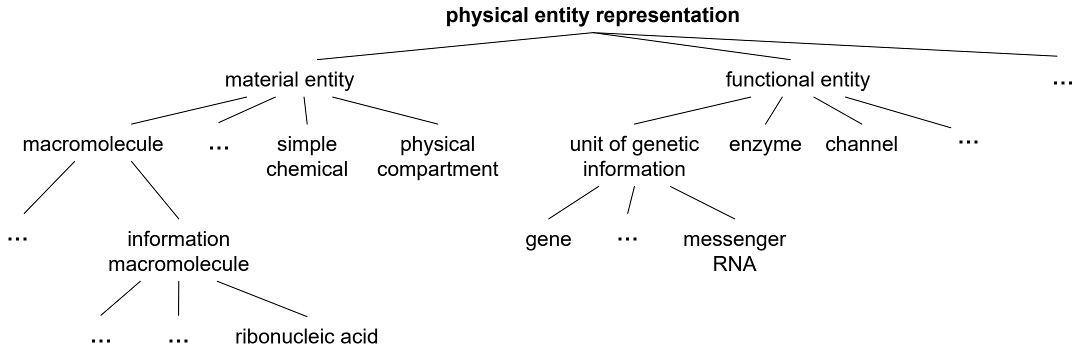

***Figure 22:*** *Partial expansion of some of the terms in the* entity *branch of SBO.*

Figure 23 shows the structure for the *participant role* branch, also grouping the concepts in a hierarchical manner. For example, in reaction rate expressions, there are a variety of possible modifiers. Some classes of modifiers can be further subdivided and grouped. All of this is easy to capture in the ontology. As more agreement is reached in the modeling community about how to define and name modifiers for different cases, the ontology can grow to accommodate it.

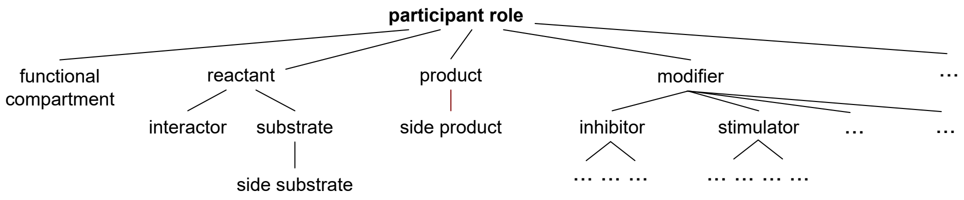

***Figure 23:*** *Partial expansion of some of the terms in the* participant role *branch of SBO.*

The controlled vocabulary for quantitative parameters is illustrated in Figure 24. Note the separation of *kinetic constant* into separate terms for unimolecular, bimolecular, etc. reactions, as well as for forward and reverse reactions. The need to have separate terms for forward and reverse rate constants arises in reversible mass-action reactions. This distinction is not always necessary for all quantitative parameters; for example, there is no comparable concept for the Michaelis constant. Another distinction for some quantitative parameters is decomposition into different versions based on the modeling framework being assumed. For example, different terms for continuous and discrete formulations of kinetic constants represent specializations of the constants for particular simulation frameworks. Not all quantitative parameters will need to be distinguished along this dimension.

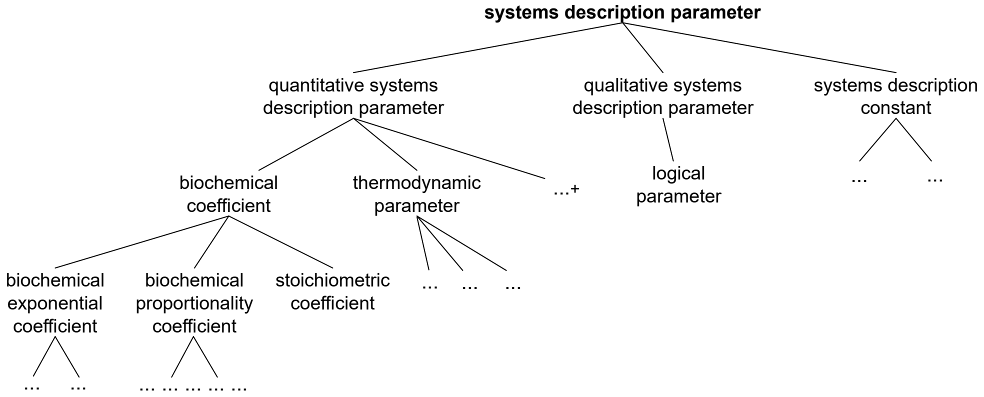

***Figure 24:*** *Partial expansion of some of the terms in the* quantitative parameter *branch.*

The terms of the SBO quantitative systems description parameter branch contain mathematical formulas that are encoded using MathML 2.0; these formulas define the parameter value using other SBO parameters. The main use of this approach is to avoid listing all the variants of a mathematical expression, escaping a combinatorial explosion.

The *modeling framework* controlled vocabulary is needed to elucidate how to simulate a mathematical expression used in models. Figure 25 illustrates the structure of this branch, which is at this point fairly simple, but we expect that more terms will evolve in the future.

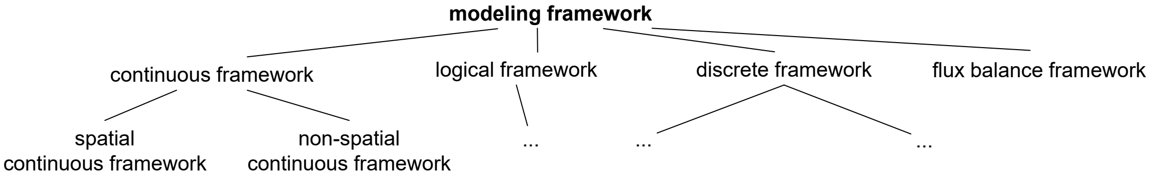

***Figure 25:*** *Partial expansion of some of the terms in the* modeling framework *branch.*

The *mathematical expression* vocabulary encompasses the various mathematical expressions that constitute a model. Figure 26 on the next page illustrates a portion of the hierarchy. Rate law or conservation law formulas are part of the mathematical expression hierarchy, and subdivided by successively more refined distinctions until the leaf terms represent precise statements of common reaction or rule types. Other types of mathematical expressions may be included in the future in order to be able to further characterize mathematical components of a model, such as initial assignments, assignment rules, rate rules, algebraic rules, constraints, and event triggers and assignments.

The leaf terms of the mathematical expression branch contain the mathematical formulas encoded using MathML 2.0. There are many potential uses for this. One is to allow a software application to obtain the formula corresponding to a term and use it as the basis of an expression to insert into a model. In effect, the formulas given in the CV act as templates for what to put into an SBML construct such as **KineticLaw** or

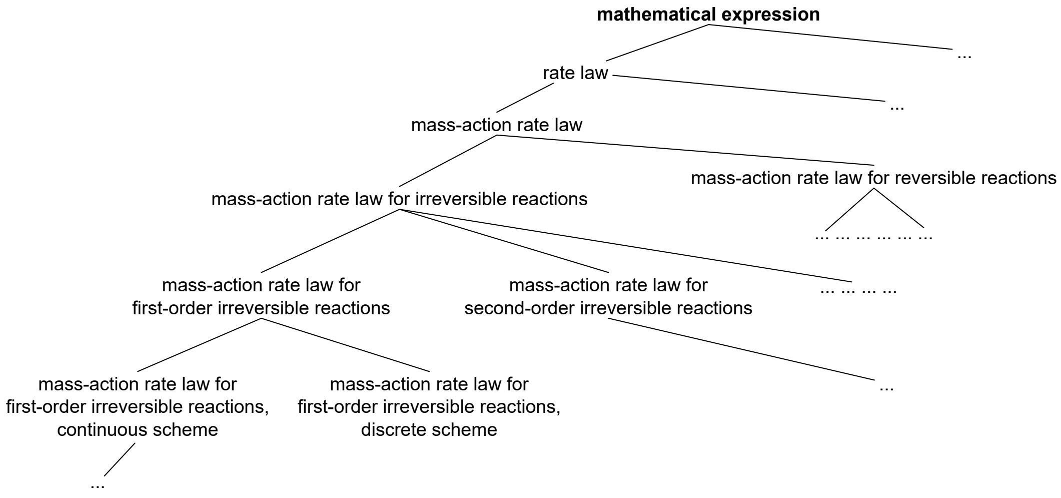

***Figure 26:*** *Partial expansion of some of the terms in the* mathematical expression *branch.*

***Rule***. The MathML definition also acts as a precise statement about the rate law in question. In particular, it carries information about the modeling framework to use in order to interpret the formula. Some of the non-leaf terms also contain formulas encoded using MathML 2.0. In that case, the formulas contained in the children terms are specific versions of the formula contained in the parent term. Those formulas may be generic, containing MathML constructs not yet supported by SBML, and need to be expanded into the MathML subset allowed in SBML before they can be used in conjunction with SBML models.

To make this discussion concrete, here is an example definition of an entry in the SBO rate law hierarchy at the time of this writing. This term represents second-order, irreversible, mass-action rate laws with one reactant, formulated for use in a continuous modeling framework:

*ID*: `SBO:0000052`

*Name*: mass-action rate law for second-order irreversible reactions, one reactant, continuous scheme

*Definition*: Reaction scheme where the products are created from the reactants and the change of a product quantity is proportional to the product of reactant activities. The reaction scheme does not include any reverse process that creates the reactants from the products. The change of a product quantity is proportional to the square of one reactant quantity. It is to be used in a reaction modeled using a continuous framework.

*Parent(s)*:
`SBO:0000050`: mass-action rate law for second-order irreversible reactions, one reactant (*is-a*).
`SBO:0000163`: mass-action rate law for irreversible reactions, continuous sceheme (*is-a*).

*MathML*:

```
<math xmlns="http://www.w3.org/1998/Math/MathML">
   <semantics definitionURL="http://biomodels.net/sbo/#SBO:0000062">
      <lambda>
         <bvar><ci definitionURL="http://biomodels.net/sbo/#SBO:0000036">k</ci></bvar>
         <bvar><ci definitionURL="http://biomodels.net/sbo/#SBO:0000509">R</ci></bvar>
         <apply>
            <times/>
            <ci>k</ci>
            <ci>R</ci>
            <ci>R</ci>
         </apply>
      </lambda>
   </semantics>
</math>
```

In the MathML definition of the term shown above, the bound variables in the `lambda` expression are tagged with references to terms in the SBO *systems description parameter* branch (for `k` and `R`). This makes it possible for software applications to interpret the intended meanings of the parameters in the expression. This also permits to convert an expression into another, by using the MathML 2.0 formula contained in the SBO terms associated with the parameters.

The *occurring entity representation* branch of SBO defines types of biological processes, events or relationship involving entities. It lists the types of biochemical reactions, such as binding, conformational transition, or cleavage, and also the different controls that modify a biochemical reaction, such as inhibition, catalysis, etc.

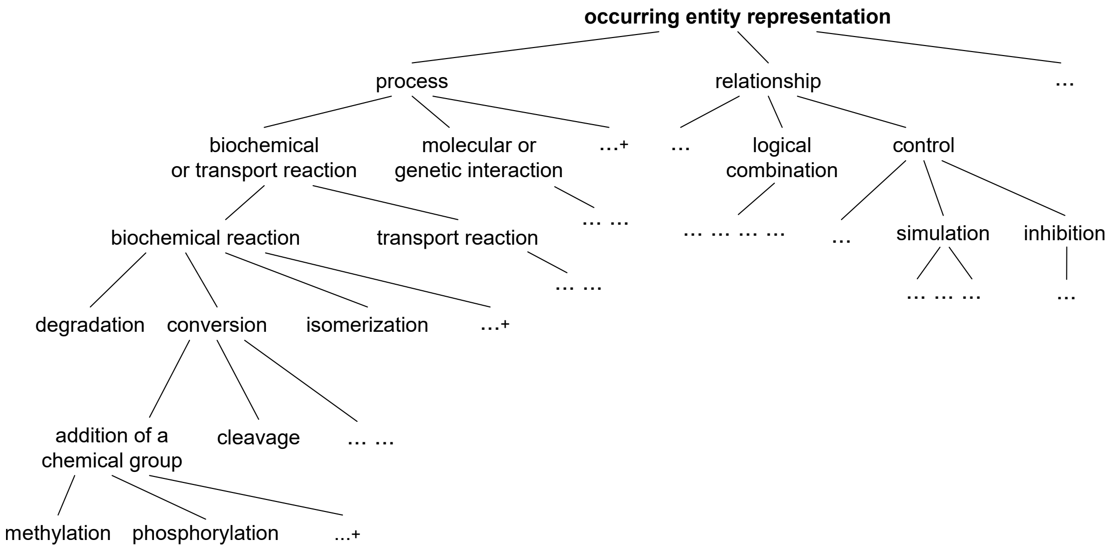

***Figure 27:*** *Partial expansion of some of the terms in the* occurring entity representation *branch.*

One of the goals of SBO is to permit a tool to traverse up and down the hierarchy in order to find equivalent terms in different frameworks. The hope is that when a software tool encounters a given rate formula in a model, the formula will be a specific form (say, "mass-action rate law, second order, one reactant, for discrete simulation"), but by virtue of the consistent organization of the reaction rate CV into framework-specific definitions, and the declaration of every parameters involved in each expression, the tool should in principle be able to determine the definitions for other frameworks (say, "mass-action rate law, second order, one reactant for *continuous* simulation"). If the software tool is designed for continuous simulation and it encounters an SBML model with rate laws formulated for discrete simulation, it could in principle look up the rate laws' identifiers in the CV and search for alternative definitions intended for discrete simulation. And of course, the converse is true, for when a tool designed for discrete simulation encounters a model with rate laws formulated for continuous simulation.

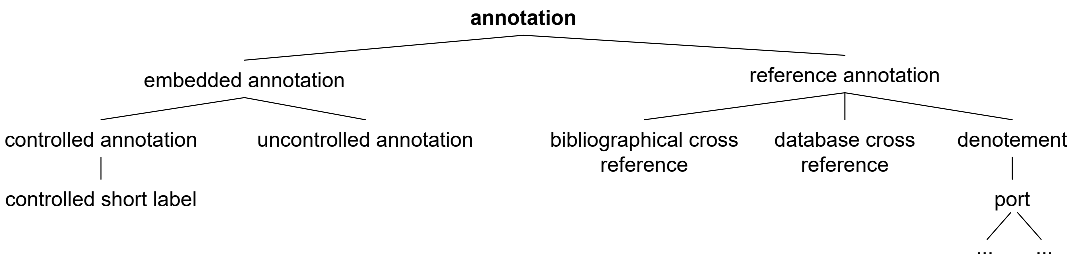

***Figure 28:*** *Current expansion of the terms in the* annotation *branch of SBO (under* metadata representation*).*

The controlled vocabulary for annotations is illustrated in Figure 28 on the preceding page, the single child of the *metadata representation* branch of SBO. As this branch is for annotating annotations themselves, its branches cannot usually be used for ***SBase***-derived objects, as those generally depict basic model information, and not annotations of that model. However, there are packages which may be able to use this branch of SBO: the "`port`" element exactly corresponds to the **Port** class from the SBML Level 3 Hierarchical Model Composition package, for example.

##### 5.2.2 Tradeoffs in using SBO terms

The SBO-based approach to annotating SBML components with controlled terms has the following strengths:

1. The syntax is minimally intrusive and maximally simple, requiring only one string-valued attribute.
2. It supports a significant fraction of what SBML users have wanted to do with controlled vocabularies.
3. It does not interfere with any other scheme. The more general annotation-based approach described in Section 6 on p. 100 can still be used simultaneously in the same model.

The scheme has the following weaknesses:

1. An object can only have one `sboTerm` attribute; therefore, it can only be related to a single term in SBO. (This also impacts the design of SBO: it must be structured such that a class of SBML elements can logically only be associated with one class of terms in the ontology.)
2. The only relationship that can be expressed by `sboTerm` is "is a". It is not possible to represent different relationships (known as *verbs* in ontology-speak). This limits what can be expressed using SBO.

The weaknesses are not shared by the annotation scheme described in Section 6 on p. 100.

##### 5.2.3 Relationships between individual SBML components and SBO terms

The `sboTerm` attribute is defined on the abstract class ***SBase*** and can be used in all derived elements. However, not all SBO terms should be used to annotate all SBML elements. Table 6 on the next page summarizes the relationships between SBML components and the branches within SBO that apply to that component. (There are currently no specific SBO term that correspond to the **SBML**, **UnitDefinition**, **Unit**, and various **ListOf**______ list classes.)

The parent identifiers shown in Table 6 on the following page are provided for reference. They are the highest-level terms in their respective branch; however, these are *not* the terms that would be used to annotate an element in SBML, because there are more specific terms underneath the parents shown here. A software tool should use the most specific SBO term available for a given concept rather than using the top-level identifier acting as the root of that particular vocabulary.

#### 5.3 Relationships to the SBML `annotation` element

Another way to provide this information would be to place SBO terms inside the ***SBase*** `annotation` element (Section 3.2 on p. 14 and Section 6 on p. 100). However, in the interest of making the use of SBO in SBML as interoperable as possible between software tools, the best-practice recommendation is to place SBO references in the `sboTerm` attribute rather than inside the `annotation` element of an object. If instead the approach of using `annotation` is taken, the qualifiers (Section 6.5 on p. 103) linking the SBML element and SBO term should be chosen extremely carefully, since it will no longer be possible to assume an "instance to class" relationship.

Although `sboTerm` is just another kind of optional annotation in SBML, SBO references are separated into their own attribute on SBML components, both to simplify their use for software tools and because doing so asserts a stronger and more focused connection in a more regimented fashion. SBO references are intended to allow a modeler to make a statement of the form "this object is identical in meaning and intention to the object defined in the term X of SBO", and do so in a way that a *software tool can interpret unambiguously.*

*Table 6:* SBML components and the main types of SBO terms that may be assigned to them. The identifiers of the highest-level SBO terms in each branch are provided for guidance, but actual values used for `sboTerm` attributes should be more specific child terms within these branches. Note that the important aspect here is the set of specific SBO identifiers, not the SBO term names, because the names may change as SBO continues to evolve. See text for further explanations.

| SBML Component | SBO Branch | Branch Identifier |
|---|---|---|
| SBML | modeling framework | SBO:0000004 |
| Model | occurring entity representation *or* modeling framework | SBO:0000231 *or* SBO:0000004 |
| FunctionDefinition | mathematical expression | SBO:0000064 |
| Compartment | physical entity representation | SBO:0000236 |
| Species | physical entity representation | SBO:0000236 |
| Reaction | occurring entity representation | SBO:0000231 |
| Parameter | systems description parameter | SBO:0000545 |
| SpeciesReference | participant role | SBO:0000003 |
| ModifierSpeciesReference | participant role | SBO:0000003 |
| KineticLaw | rate law | SBO:0000001 |
| LocalParameter | systems description parameter | SBO:0000545 |
| InitialAssignment | mathematical expression | SBO:0000064 |
| AlgebraicRule | mathematical expression | SBO:0000064 |
| AssignmentRule | mathematical expression | SBO:0000064 |
| RateRule | mathematical expression | SBO:0000064 |
| Constraint | mathematical expression | SBO:0000064 |
| Event | occurring entity representation | SBO:0000231 |
| Trigger | mathematical expression | SBO:0000064 |
| Priority | mathematical expression | SBO:0000064 |
| Delay | mathematical expression | SBO:0000064 |
| EventAssignment | mathematical expression | SBO:0000064 |

Some software applications may have their own vocabulary of terms similar in purpose to SBO. For maximal software interoperability, the best-practice recommendation in SBML is nonetheless to use SBO terms in preference to using application-specific annotation schemes. Software applications should therefore attempt to translate their private terms to and from SBO terms when writing and reading SBML, respectively.

### 5.4 Discussion

Here we discuss some additional points about the SBO-based approach.

#### *5.4.1 Frequency of change in the ontology*

The SBO development approach follows conventional ontology development approaches in bioinformatics. One of the principles being followed is that identifiers and meanings of terms in the CVs never change and the terms are never deleted. Where some terms are deemed obsolete, the introduction of new terms refine or supersede existing terms, but the existing identifiers are left in the CV. Thus, references never end up pointing to nonexistent entries. In the case where synonymous terms are merged after agreement that multiple terms are identical, the term identifiers are again left in the CV and they still refer to the same concept as before. Out-of-date terms cached or hard-coded by an application remain usable in all cases. (Moreover, machine-readable CV encodings and appropriate software design should render possible the development of API libraries that automatically map older terms to newer terms as the CVs evolve.) Therefore, a model is never in danger of ending up with SBO identifiers that cannot be dereferenced. If an application finds an old model with a term `SBO:0000065`, it can be assured that it will be able to find this term in SBO, even if it has been superseded by other, more preferred terms.

#### *5.4.2 Consistency of information*

If you have a means of linking (say) a reaction rate formula to a term in a CV, it is possible to have an inconsistency between the formula in the SBML model and the one defined for the CV term. However, this is not a new problem; it arises in other situations involving SBML models already. The guideline for these situations is that the model must be self-contained and stand on its own. Therefore, in cases where they differ, the definitions in the SBML model take precedence over the definitions referenced by the CV. In other words, the model (and its MathML) is authoritative.

##### 5.4.3 Implications for network access

A software tool does not need to have the ability to access the network or read the CV every time it encounters a model or otherwise works with SBML. Since the SBO will likely stabilize and change infrequently once a core set of terms is defined, applications can cache the controlled vocabulary, and not make network accesses to the master SBO copy unless something forces them to (e.g., detecting a reference in a model to an SBO term that the application does not recognize). Applications could have user preference settings indicating how often the CV definitions should be refreshed (similar to how modern applications provide a setting dictating how often they should check for new versions of themselves). Simple applications may go further and hard code references to terms in SBO that have reached stability and community consensus. SBO is available for download under different formats (`http://biomodels.net/sbo/`). Web services are also available to provide programmatic access to the ontology.

## 6 A standard format for the annotation element

This section describes the recommended non-proprietary format for the content of **Annotation** objects in SBML when (a) referring to controlled vocabulary terms and database identifiers that define and describe biological and biochemical entities, and (b) describing the creator of a model and its modification history. Such a structured format should facilitate the generation of models compliant with the MIRIAM guidelines for model curation (Le Novère et al., 2005).

The format described in this section is intended to be the form of one of the top-level elements that could reside in an **Annotation** object attached to an SBML object derived from ***SBase***. The element is named `rdf:RDF`. The format described here is compliant with the constraints placed on the form of annotation elements described in Section 3.2.6 on p. 16. We refer readers to Section 3.2.6 for important information on the structure and organization of application-specific annotations; these are not described here.

The annotations described in this section are optional on a model, but if present, they must conform to the details specified here in order to be considered valid annotations in this format. If they do not conform to the format described here, it does not render the overall SBML model invalid, but the annotations are then considered to be in a proprietary format rather than being *SBML MIRIAM annotations*.

### 6.1 Motivation

The SBML structures described elsewhere in this document do not have any biochemical or biological semantics. This section provides a scheme for linking SBML structures to external resources so that those structures can be given semantics. The motivation for the introduction of this scheme is similar to that given for the introduction of `sboTerm`; however, the general annotation scheme here is more flexible.

It is generally not recommended that this format be used to refer to SBO terms. In most cases, the SBO terms should be assigned using the attribute `sboTerm` on objects derived from ***SBase*** (Section 3.2.4 on p. 15). However in certain situations, for instance to be able to add additional information about the functional role of a species, it is necessary to add the information using the annotation format described here.

Annotations only add additional qualifying information and never change existing information. They can be ignored without changing the (broader) meaning of the model. The same is true of nested annotations (described below), which qualify their parent annotation but never change the meaning of that annotation.

### 6.2 XML namespaces in the standard annotation

This format uses a restricted form of Dublin Core (Dublin Core Metadata Initiative, 2005) and BioModels.net qualifier elements (see `https://co.mbine.org/standards/qualifiers`) embedded in the XML form of RDF (W3C, 2004b). The scheme defined here uses a number of external XML standards and associated XML namespaces. Table 7 lists these namespaces and relevant documentation on those namespaces. The format constrains the order of elements in these namespaces beyond the constraints defined in the standard definitions for those namespaces. For each standard listed, the format only uses a subset of the possible syntax defined by the given standard. Thus, it is possible for an `annotation` element to include XML that is compliant with those external standards but is not compliant with the format described here.

***Table 7:*** *The XML standards used in the SBML standard format for annotations. The namespace prefixes are only shown to indicate the prefix used in the main text; the prefixes are not required to be the specific strings shown here.*

| Prefix used | Namespace URI | Reference/description |
|---|---|---|
| `rdf` | `http://www.w3.org/1999/02/22-rdf-syntax-ns#` | W3C (2004a) |
| `dcterms` | `http://purl.org/dc/terms/` | Kokkelink and Schwänzl (2002), DCMI Usage Board (2005) |
| `vcard4` | `http://www.w3.org/2006/vcard/ns#` | Perreault (2011) |
| `bqbiol` | `http://biomodels.net/biology-qualifiers/` | `https://co.mbine.org/standards/qualifiers` |
| `bqmodel` | `http://biomodels.net/model-qualifiers/` | `https://co.mbine.org/standards/qualifiers` |

Note that `vCard` 3 has been deprecated in favor of `vCard` 4. Models using `vCard` 3 will continue to be legal, but tools may issue a warning that `vCard` 4 is preferred. Future versions of SBML will drop `vCard` 3 as part of the officially supported annotation 'standard format'.

#### 6.3 General syntax for the standard annotation

This standard format for an SBML annotation is placed in a single `rdf:RDF` element contained within the SBML `annotation` element. It can contain other elements in any order as described in Section 3.2.6 on p. 16. The format described in this section only defines the form of the `rdf:RDF` element. The containing SBML ***SBase*** element must have a `metaid` attribute value (and note that, because it is of XML type `ID`, its value must be unique to the entire SBML document). An outline of the format's syntax is shown below.

```
<SBML_ELEMENT +++ metaid="SBML_META_ID" +++ >
  +++
  <annotation>
    +++
    <rdf:RDF xmlns:rdf="http://www.w3.org/1999/02/22-rdf-syntax-ns#"
             xmlns:dcterms="http://purl.org/dc/terms/"
             xmlns:vcard4="http://www.w3.org/2006/vcard/ns#"
             xmlns:bqbiol="http://biomodels.net/biology-qualifiers/"
             xmlns:bqmodel="http://biomodels.net/model-qualifiers/" >
      <rdf:Description rdf:about="#SBML_META_ID">
        [HISTORY]
        <RELATION_ELEMENT>
          <rdf:Bag>
            <rdf:li rdf:resource="URI" />
            ...
            [NESTED CONTENT]
          </rdf:Bag>
        </RELATION_ELEMENT>
        ...
      </rdf:Description>
      +++
    </rdf:RDF>
    +++
  </annotation>
  +++
</SBML_ELEMENT>
```

The outline above shows the expected order of the elements. The capitalized identifiers refer to generic strings of specific types, as follows: `SBML_ELEMENT` refers to any SBML element name that can contain an `annotation` element; `SBML_META_ID` is an XML `ID` string; ***RELATION_ELEMENT*** refers to element names in either the namespace `http://biomodels.net/biology-qualifiers/` or `http://biomodels.net/model-qualifiers/` (see Section 6.5); `URI` is a URI identifying a resource (see Section 6.4); and `[HISTORY]` refers to optional content described in Section 6.6.

`[NESTED CONTENT]` has the same format and restrictions as ***RELATION_ELEMENT***:

```
<RELATION_ELEMENT>
  <rdf:Bag>
    <rdf:li rdf:resource="URI" />
    ...
    [NESTED CONTENT]
  </rdf:Bag>
</RELATION_ELEMENT>
```

`[NESTED CONTENT]` is used to describe a clarification or another annotation about the ***RELATION_ELEMENT*** in which it appears, using such elements as `bqbiol:isDescribedBy`. Nested content permits, for example, describing protein modifications on species, or adding evidence codes for an annotation. Nested content relates to its containing ***RELATION_ELEMENT***, not the other way around. It qualifies, but does not change, its meaning. Ignoring a `[NESTED CONTENT]` does not affect the information provided by the containing ***RELATION_ELEMENT***. These elements may be nested arbitrarily deep, with each successive layer describing or clarifying the annotation it is embedded within.

The string '+++' is a placeholder for either no content or valid XML content that is not defined by the annotation scheme described here but is consistent with the relevant standards for the enclosing elements. Finally, the string '...' is a placeholder for zero or more elements of the same form as the immediately preceding element. The precise form of whitespace and the XML namespace prefix definitions is not constrained; however, the elements and attributes must be in the namespaces shown. The rest of this section describes the format formally in English.

The first element of the rdf:RDF element must be an rdf:Description element with an rdf:about attribute. The value of the rdf:about attribute must be of the form #<string> where the string component is equal to the value of the metaid attribute of the containing SBML element. This format doesn't define the form of subsequent subelements of the rdf:RDF element. In particular, the unique rdf:RDF element contained in the annotation can contain other rdf:Description, which content can be any valid RDF.

The rdf:Description element can contain only an optional history section (see Section 6.6) followed by zero or more BioModels.net relation elements. The specific relation elements used will depend on the intended relationship between the SBML component and referenced information or resource. Although Section 6.5 describes the detailed semantics of each of the relation element types, the content of these elements follows the same form shown in the template above. A BioModels.net relation element must only contain a single rdf:Bag element which in turn must contain one or more rdf:li elements. The rdf:li elements must only have a rdf:resource attribute containing a URI referring to an information resource (see Section 6.4). The relation element may also contain nested content (indicated by [NESTED CONTENT] in the diagram above) with annotations *about* the contents of the rdf:Bag.

Note that the various namespaces (xmlns:rdf, xmlns:dcterms, etc.) may be declared in any order, and that only the namespaces that are actually used need be declared. If no vcard4 terms are used in a particular annotation, for example, the line xmlns:vcard4="http://www.w3.org/2006/vcard/ns#" is optional.

### 6.4 Use of URIs

The SBML MIRIAM annotation format allows the expression of relationships between SBML elements on the one hand, and resources referred to by values of rdf:resource attributes on the other. The BioModels.net relation elements (see Section 6.5 on the following page) simply define the nature of the relationship.

The value of a rdf:resource attribute is a URI that uniquely identifies both the resource and the data within the resource. Since a URI is not a URL, it does not have to map to a physical Web object; it simply needs to identify, uniquely, a controlled vocabulary term or database object. It is essentially just a label. For instance, an actual URL for an Internet resource might be https://www.uniprot.org/uniprot/P12999, and this might correspond to the URI https://identifiers.org/uniprot/P12999 (Juty et al., 2012).

It is important that the portion of a rdf:resource value that identifies a data entry is always a perennial identifier. For example, a **Species** object representing a protein could be annotated with a reference to the database UniProt by the resource identifier https://identifiers.org/uniprot/P12999, thereby identifying exactly the intended protein. This identifier maps to a unique entry in UniProt which is never deleted from the database. In the case of UniProt, this is known as the "accession" portion of the entry. When the entry is merged with another one, both "accession" entries are conserved. A UniProt entry also possesses an "entry name" (the Swiss-Prot "identifier"), a "protein name", "synonyms", and other parts, but only the "accession" is perennial, and that is what should be used.

SBML does not define how to interpret URIs. There may be several ways of transforming a URI into a physical URL; e.g., https://identifiers.org/go/GO:0007268 can be translated into any of the following:

- http://amigo.geneontology.org/amigo/term/GO:0007268
- https://www.ebi.ac.uk/QuickGO/GTerm?id=GO:0007268
- http://www.informatics.jax.org/vocab/gene_ontology/GO:0007268

Similarly, https://identifiers.org/ec-code/3.5.4.4 can refer to any of the following (among many):

- https://www.ebi.ac.uk/intenz/query?cmd=SearchEC&ec=3.5.4.4
- https://www.metanetx.org/cgi-bin/mnxweb/search?query=3.5.4.4
- https://enzyme.expasy.org/EC/3.5.4.4

To enable interoperability of URIs between software systems, the community has standardized the URI rules for use within the SBML MIRIAM annotation format. These URIs are not part of the SBML standard per se, and will grow independently from specific SBML levels and versions. As the set changes, existing URIs will not be modified, although the physical resources associated with each one may change (for example, to use updated URLs). The form of the URIs will always have the form *resource:identifier*. An up-to-date list and explanations of the URIs are available online at the address `https://www.ebi.ac.uk/miriam/main/collections`. Each entry lists the database in question, the URI to be used to reference that database, and example URIs for referencing particular entries in those databases. The URI rule list will evolve with the evolution of databases and resources.

Note this means that all `rdf:resource` *must* be MIRIAM URIs and thus cannot refer to, for example, other elements in the model. While it would be possible to place such information in RDF content elsewhere (e.g., after the first `rdf:Description` element), the information will be outside the scope of the simple annotation scheme described here, and as such, there is no guarantee that other software could understand it.

### 6.5 Relation elements

Different BioModels.net qualifier elements encode different types of relationships. As described above, when appearing in an annotation, each qualifier element encloses a set of `rdf:li` elements. Its appearance in a relation element implies a specific relationship between the enclosing SBML object and the resources referenced by the `rdf:li` elements. When several relation elements with the same name are placed in the same SBML element's annotation, they represent alternatives. For example, two `bqbiol:hasPart` elements within a **Species** object represent two different sets of references to the parts making up the biological entity represented by the species. (The species is not made up of *all* the entities represented by all the references combined; they are alternatives.)

Table 8 on the next page lists the elements defined at the time of this writing. The list is divided into two symbol namespaces: `http://biomodels.net/model-qualifiers/` is for model qualifiers (for which we use the prefix `bqmodel` in examples shown in this section). The other namespace is for biological qualifiers; this has the URI `http://biomodels.net/biology-qualifiers/` (for which we use the prefix `bqbiol`). The list will only grow; i.e., no element will be removed from the list.

*Table 8:* BioModels.net qualifiers at the time of this writing, and a summary of their meanings. The complete list of the qualifier elements is documented online at http://biomodels.net/qualifiers/. (The definitions given above are slightly modified compared to the originals, to reflect the SBML-specific nature of this SBML specification document.)

| Qualifier element | Meaning |
|---|---|
| `bqmodel:hasInstance` | The subject of the referenced resource is an instance of the modeling object. This could be used, for example, to link a generic model with its specific forms. |
| `bqmodel:is` | The modeling object encoded by the SBML component is the subject of the referenced resource. This might be used, e.g., to link the model to an entry in a model database. |
| `bqmodel:isDerivedFrom` | The modeling object represented by the component of the encoded model is derived from the modeling object represented by the referenced resource. For instance, they can be the fruit of a refinement or their adaptation for use in a different context. |
| `bqmodel:isDescribedBy` | The modeling object encoded by the SBML component is described by the referenced resource. This could link a component (e.g., a reaction) to a publication describing it. |
| `bqmodel:isInstanceOf` | The modeling object represented by the model element is an instance of the subject of the referenced resource. |
| `bqbiol:encodes` | The biological entity represented by the model component encodes, either directly or by virtue of transitivity, the subject of the referenced resource. |
| `bqbiol:hasPart` | The biological entity represented by the SBML component includes the subject of the referenced resource, either physically or logically. This relation might be used to link a complex to a description of its components. |
| `bqbiol:hasProperty` | The subject of the referenced resource is a property of the biological entity represented by the model component. This relation might be used when a biological entity has a given activity or exerts a specific function. |
| `bqbiol:hasVersion` | The subject of the referenced resource is a version or an instance of the biological entity represented by the SBML component. |
| `bqbiol:is` | The biological entity represented by the SBML component is the subject of the referenced resource. This could serve to link a reaction to its counterpart in (e.g.) the ChEBI or Reactome databases. |
| `bqbiol:isDescribedBy` | The biological entity represented by the SBML component is described by the referenced resource. This relation could be used, for example, to link a species or a parameter to a publication describing the quantity of the species or the value of the parameter. |
| `bqbiol:isEncodedBy` | The biological entity represented by the model component is encoded, either directly or by virtue of transitivity, by the subject of the referenced resource. |
| `bqbiol:isHomologTo` | The biological entity represented by the SBML component is homolog, to the subject of the referenced resource, i.e., they share a common ancestor. |
| `bqbiol:isPartOf` | The biological entity represented by the SBML component is a physical or logical part of the subject of the referenced resource. This relation might be used to link a component to the description of the complex to which it belongs. |
| `bqbiol:isPropertyOf` | The biological entity represented by the SBML component is a property of the referenced resource. |
| `bqbiol:isVersionOf` | The biological entity represented by the SBML component is a version or an instance of the subject of the referenced resource. |
| `bqbiol:occursIn` | The biological entity represented by the SBML component takes place in the subject of the reference resource. |
| `bqbiol:hasTaxon` | The biological entity represented by the SBML component is taxonomically restricted, where the restriction is the subject of the referenced resource. This relation may be used to ascribe a species restriction to a biochemical reaction. |

### 6.6 History

The SBML MIRIAM annotation format described in Section 6.3 on p. 101 can include additional elements to describe the history of the *SBML encoding of the model* or its individual components. (Note the emphasis on the SBML encoding—the history of the conceptual model underlying the encoding is not addressed by this scheme.) If this history data is present, it must occur immediately before the first BioModels.net relation elements of an annotation. The history encodes information about the creator(s) of the encoding and a sequence of dates recording the dates of creation and subsequent modifications of the SBML model encoding. The syntax for these elements is outlined below.

```
+++
<vCard4:fn>
  <vCard4:text> FULL_NAME </vCard4:text>
</vCard4:fn>
+++
[<vCard4:hasEmail> EMAIL_ADDRESS </vCard4:hasEmail>]
+++
[<vCard4:organization-name> ORGANIZATION_NAME </vCard4:organization-name>]
+++
```

Or, to more closely match vCard 3, **vCard4:hasName** may be used instead of **vCard4:fn**:

```
+++
<vCard4:hasName>
  <vCard4:family-name> FAMILY_NAME </vCard4:family-name>
  <vCard4:given-name> GIVEN_NAME </vCard4:given-name>
</vCard4:hasName>
+++
[<vCard4:hasEmail> EMAIL_ADDRESS </vCard4:hasEmail>]
+++
[<vCard4:organization-name> ORGANIZATION_NAME </vCard4:organization-name>]
+++
```

The order of elements must be as shown above, except that elements of the format contained in the light gray box (**vCard4:fn** or **vCard4:hasName**, plus **vCard4:hasEmail**, and **vCard4:organization-name**) can occur in any order. The elements of the format contained between [ and ] (**vCard4:organization-name**, and **vCard4:hasEmail**) are optional, but everything else is required. The precise form of the whitespace, and the specific XML namespace prefixes used ("**dcterms**", "**rdf**", and "**vcard4**") are not constrained.

The **dcterms:creator** element describes the person(s) who created the SBML encoding of the model or model component. It must contain a single **rdf:Bag** element. The **rdf:Bag** element can contain any number of elements; however, the first such element must be **rdf:li**. The **rdf:li** element can in turn contain any number of elements in any order. Among those elements can be the following: **vCard4:fn** or **vCard4:hasName**, plus **vCard4:hasEmail**, and **vCard4:organization-name**. The **dcterms:created**, and **dcterms:modified** elements must have the attribute **rdf:parseType** set to the literal value "**Resource**".

Note that **dcterms:creator** has been added to **http://purl.org/dc/terms/** relatively recently, but the same term (with the same meaning) exists in the **http://purl.org/dc/elements/1.1/** namespace. It is legal to continue using the old namespace (called "**dc**" in previous versions of the SBML specifications), but since all the terms defined there are now also defined in **http://purl.org/dc/terms/**, we recommend using the latter.

The content placeholders FAMILY_NAME and GIVEN_NAME stand for the family name (surname) and the first (given) name, respectively, of a person who created the model; when using **vCard4**, FULL_NAME stands for the full name of that person. EMAIL_ADDRESS is the email address of the same person who created the model; and ORGANIZATION_NAME is the name of the organization with which the same person who created the model is affiliated. The string DATE is a date in W3C date format (Wolf and Wicksteed, 1998), which is a profile of (i.e., a restricted form of) ISO 8601. Finally, as in the overall template shown in Section 6.3 on p. 101, '+++'

is a placeholder for either no content or valid XML syntax that is not defined by this scheme but is consistent with the relevant standards for the enclosing elements, and ellipses (‘`...`’) are placeholders for zero or more elements of the same form as the immediately preceding element.

Section 6.7 below provides an example of using these history elements in the SBML MIRIAM annotation format.

### 6.7 Examples

The following shows the annotation of a model with model creation data and links to external resources:

```
<model metaid="_180340" id="GMO" name="Goldbeter1991_MinMitOscil">
  <annotation>
    <rdf:RDF
            xmlns:rdf="http://www.w3.org/1999/02/22-rdf-syntax-ns#"
            xmlns:dcterms="http://purl.org/dc/terms/"
            xmlns:vcard4="http://www.w3.org/2001/vcard-rdf/3.0#"
            xmlns:bqbiol="http://biomodels.net/biology-qualifiers/"
            xmlns:bqmodel="http://biomodels.net/model-qualifiers/"
    >
      <rdf:Description rdf:about="#_180340">
        <dcterms:creator>
          <rdf:Bag>
            <rdf:li rdf:parseType="Resource">
              <vcard4:fn><vcard4:text>Bruce Shapiro</vcard4:text></vcard4:fn>
              <vcard4:email>bshapiro@jpl.nasa.gov</vcard4:email>
              <vcard4:organization-name>
                  NASA Jet Propulsion Laboratory
              </vcard4:organization-name>
            </rdf:li>
          </rdf:Bag>
        </dcterms:creator>
        <dcterms:created rdf:parseType="Resource">
          <dcterms:W3CDTF>2005-02-06T23:39:40+00:00</dcterms:W3CDTF>
        </dcterms:created>
        <dcterms:modified rdf:parseType="Resource">
          <dcterms:W3CDTF>2005-09-13T13:24:56+00:00</dcterms:W3CDTF>
        </dcterms:modified>
        <bqmodel:is>
          <rdf:Bag>
            <rdf:li rdf:resource="https://identifiers.org/biomodels.db/BIOMD0000000003"/>
          </rdf:Bag>
        </bqmodel:is>
        <bqmodel:isDescribedBy>
           <rdf:Bag>
             <rdf:li rdf:resource="https://identifiers.org/pubmed/1833774"/>
           </rdf:Bag>
        </bqmodel:isDescribedBy>
        <bqbiol:isVersionOf>
          <rdf:Bag>
            <rdf:li rdf:resource="https://identifiers.org/wikipathways/WP179"/>
            <rdf:li rdf:resource="https://identifiers.org/reactome/REACT_152"/>
          </rdf:Bag>
        </bqbiol:isVersionOf>
      </rdf:Description>
    </rdf:RDF>
  </annotation>
</model>
```

The following example shows a **Reaction** object annotated with a reference to its exact Reactome counterpart.

```
<reaction id="cdc2Phospho" metaid="jb007" reversible="true">
  <annotation>
    <rdf:RDF
      xmlns:bqbiol="http://biomodels.net/biology-qualifiers/"
      xmlns:rdf="http://www.w3.org/1999/02/22-rdf-syntax-ns#"
    >
      <rdf:Description rdf:about="#jb007">
```

```
            <bqbiol:is>
              <rdf:Bag>
                <rdf:li rdf:resource="https://identifiers.org/reactome/REACT_6327"/>
              </rdf:Bag>
            </bqbiol:is>
          </rdf:Description>
        </rdf:RDF>
      </annotation>
      <listOfReactants>
        <speciesReference species="cdc2" stoichiometry="1"/>
      </listOfReactants>
      <listOfProducts>
        <speciesReference species="cdc2-Y15P" stoichiometry="1"/>
      </listOfProducts>
      <listOfModifiers>
        <modifierSpeciesReference species="wee1"/>
      </listOfModifiers>
    </reaction>
```

The following example describes a species that represents a complex between the protein calmodulin and calcium ions:

```
    <species id="Ca_calmodulin" metaid="cacam" compartment="C"
             hasOnlySubstanceUnits="false" boundaryCondition="false"
             constant="false">
      <annotation>
        <rdf:RDF
          xmlns:rdf="http://www.w3.org/1999/02/22-rdf-syntax-ns#"
          xmlns:bqbiol="http://biomodels.net/biology-qualifiers/"
        >
          <rdf:Description rdf:about="#cacam">
            <bqbiol:hasPart>
              <rdf:Bag>
                <rdf:li rdf:resource="https://identifiers.org/uniprot/P62158"/>
                <rdf:li rdf:resource="https://identifiers.org/chebi/CHEBI:29108"/>
              </rdf:Bag>
            </bqbiol:hasPart>
          </rdf:Description>
        </rdf:RDF>
      </annotation>
    </species>
```

The following example describes a species that represents either “Calcium/calmodulin-dependent protein kinase type II alpha chain” or “Calcium/calmodulin-dependent protein kinase type II beta chain”. This is the case, for example, in the somatic cytoplasm of striatal medium-size spiny neurons, where both are present but they cannot be functionally differentiated.

```
    <species id="calcium_calmodulin" metaid="cacam" compartment="C"
             hasOnlySubstanceUnits="false" boundaryCondition="false"
             constant="false">
      <annotation>
        <rdf:RDF
          xmlns:rdf="http://www.w3.org/1999/02/22-rdf-syntax-ns#"
          xmlns:bqbiol="http://biomodels.net/biology-qualifiers/"
        >
          <rdf:Description rdf:about="#cacam">
            <bqbiol:hasVersion>
              <rdf:Bag>
                <rdf:li rdf:resource="https://identifiers.org/uniprot/Q9UQM7"/>
                <rdf:li rdf:resource="https://identifiers.org/uniprot/Q13554"/>
              </rdf:Bag>
            </bqbiol:hasVersion>
          </rdf:Description>
        </rdf:RDF>
      </annotation>
    </species>
```

The above approach should not be used to describe “any Calcium/calmodulin-dependent protein kinase type II chain", because such an annotation requires referencing the products of other genes such as gamma or delta. All the known proteins could be enumerated, but such an approach would almost surely lead to inaccuracies because biological knowledge continues to evolve. Instead, the annotation should refer to generic information such as Ensembl family ENSFM00250000000111 "CALCIUM/CALMODULIN DEPENDENT KINASE TYPE II CHAIN" or PIR superfamily PIRSF000594 "Calcium/calmodulin-dependent protein kinase type II".

The following two examples show how to use the qualifier `isVersionOf`. The first example is the relationship between a reaction and an EC code. An EC code describes an enzymatic activity and an enzymatic reaction involving a particular enzyme can be seen as an instance of this activity. For example, the following reaction represents the phosphorylation of a glutamate receptor by a complex calcium/calmodulin kinase II.

```
<reaction id="NMDAR_phosphorylation" metaid="thx1138"
          reversible="true">
  <annotation>
    <rdf:RDF
      xmlns:bqbiol="http://biomodels.net/biology-qualifiers/"
      xmlns:rdf="http://www.w3.org/1999/02/22-rdf-syntax-ns#"
    >
      <rdf:Description rdf:about="#thx1138">
        <bqbiol:isVersionOf>
          <rdf:Bag>
            <rdf:li rdf:resource="https://identifiers.org/ec-code/2.7.1.17"/>
          </rdf:Bag>
        </bqbiol:isVersionOf>
      </rdf:Description>
    </rdf:RDF>
  </annotation>
  <listOfReactants>
    <speciesReference species="NMDAR" stoichiometry="1"/>
  </listOfReactants>
  <listOfProducts>
    <speciesReference species="P-NMDAR" stoichiometry="1"/>
  </listOfProducts>
  <listOfModifiers>
    <modifierSpeciesReference species="CaMKII"/>
  </listOfModifiers>
  <kineticLaw>
    <math xmlns="http://www.w3.org/1998/Math/MathML">
      <apply>
        <times/>
        <ci>CaMKII</ci>
        <ci>kcat</ci>
        <apply>
          <divide/>
          <ci>NMDAR</ci>
          <apply> </times> <ci>NMDAR</ci> <ci>Km</ci> </apply>
        </apply>
      </apply>
    </math>
    <listOfLocalParameters>
      <localParameter id="kcat" value="1"/>
      <localParameter id="Km" value="5e-10"/>
    </listOfLocalParameters>
  </kineticLaw>
</reaction>
```

The second example of the use of `isVersionOf` is the complex between Calcium/calmodulin-dependent protein kinase type II alpha chain and Calcium/calmodulin, that is only one of the "calcium- and calmodulin-dependent protein kinase complexes" described by the Gene Ontology term GO:0005954. (Note also how the GO identifier is written—we return to this point below.)

```
<species id="CaCaMKII" metaid="C8H10N4O2" compartment="C"
         hasOnlySubstanceUnits="false" boundaryCondition="false"
         constant="false">
  <annotation>
```

```
            <rdf:RDF
              xmlns:rdf="http://www.w3.org/1999/02/22-rdf-syntax-ns#"
              xmlns:bqbiol="http://biomodels.net/biology-qualifiers/"
            >
              <rdf:Description rdf:about="#C8H10N4O2">
                <bqbiol:isVersionOf>
                  <rdf:Bag>
                    <rdf:li rdf:resource="https://identifiers.org/go/GO:0005954"/>
                  </rdf:Bag>
                </bqbiol:isVersionOf>
              </rdf:Description>
            </rdf:RDF>
          </annotation>
        </species>
```

In the example above, the URN for the GO term is written as `GO%3A0005954`, yet in reality, the actual GO identifier is `GO:0005954`. The reason for this rests in the definition of RDF/XML and URNs. The essential point is that the colon character ("`:`") is a reserved character representing the component separator in URNs. Thus, when an identifier contains a colon character as part of it (as GO, ChEBI, and certain other identifiers do), the colon characters must be percent-encoded. The sequence "`%3A`" is the percent-encoded form of "`:`". Applications must percent-encode "`:`" characters that appear in entity identifiers (whether from ECO, ChEBI, GO, or other) when writing them in MIRIAM URIs, and percent-decode the identifiers when reading the URIs. More examples of this appear throughout the rest of this section.

The previous case is different from the following one, although they could seem similar at first sight. The "Calcium/calmodulin-dependent protein kinase type II alpha chain" is a part of the above mentioned "calcium- and calmodulin-dependent protein kinase complex".

```
        <species id="CaMKIIalpha" metaid="C10H14N2" compartment="C"
                 hasOnlySubstanceUnits="false" boundaryCondition="false"
                 constant="false">
          <annotation>
            <rdf:RDF
              xmlns:rdf="http://www.w3.org/1999/02/22-rdf-syntax-ns#"
              xmlns:bqbiol="http://biomodels.net/biology-qualifiers/"
            >
              <rdf:Description rdf:about="#C10H14N2">
                <bqbiol:isPartOf>
                  <rdf:Bag>
                    <rdf:li rdf:resource="https://identifiers.org/go/GO:0005954"/>
                  </rdf:Bag>
                </bqbiol:isPartOf>
              </rdf:Description>
            </rdf:RDF>
          </annotation>
        </species>
```

It is possible describe a component with several alternative sets of qualified annotations. For example, the following species represents a pool of GMP, GDP and GTP. We annotate it with the three corresponding KEGG compound identifiers but also with the three corresponding ChEBI identifiers. The two alternative annotations are encoded in separate `hasVersion` qualifier elements.

```
        <species id="GXP" metaid="GXP" compartment="C"
                 hasOnlySubstanceUnits="false" boundaryCondition="false"
                 constant="false">
          <annotation>
            <rdf:RDF
              xmlns:rdf="http://www.w3.org/1999/02/22-rdf-syntax-ns#"
              xmlns:bqbiol="http://biomodels.net/biology-qualifiers/"
            >
              <rdf:Description rdf:about="#GXP">
                <bqbiol:hasVersion>
                  <rdf:Bag>
                    <rdf:li rdf:resource="https://identifiers.org/chebi/CHEBI:17345"/>
                    <rdf:li rdf:resource="https://identifiers.org/chebi/CHEBI:17552"/>
                    <rdf:li rdf:resource="https://identifiers.org/chebi/CHEBI:17627"/>
```

```
            </rdf:Bag>
          </bqbiol:hasVersion>
          <bqbiol:hasVersion>
            <rdf:Bag>
              <rdf:li rdf:resource="https://identifiers.org/kegg.compound/C00035"/>
              <rdf:li rdf:resource="https://identifiers.org/kegg.compound/C00044"/>
              <rdf:li rdf:resource="https://identifiers.org/kegg.compound/C00144"/>
            </rdf:Bag>
          </bqbiol:hasVersion>
        </rdf:Description>
      </rdf:RDF>
    </annotation>
  </species>
```

The following example presents a reaction being actually the combination of three different elementary molecular reactions. We annotate it with the three corresponding KEGG reactions, but also with the three corresponding enzymatic activities. Again the two `hasPart` elements represent two alternative annotations. The process represented by the **Reaction** structure is composed of three parts, and not six parts.

```
  <reaction id="adenineProd" metaid="adeprod" reversible="true">
    <annotation>
      <rdf:RDF
        xmlns:bqbiol="http://biomodels.net/biology-qualifiers/"
        xmlns:rdf="http://www.w3.org/1999/02/22-rdf-syntax-ns#"
      >
        <rdf:Description rdf:about="#adeprod">
          <bqbiol:hasPart>
            <rdf:Bag>
              <rdf:li rdf:resource="https://identifiers.org/ec-code/2.5.1.22"/>
              <rdf:li rdf:resource="https://identifiers.org/ec-code/3.2.2.16"/>
              <rdf:li rdf:resource="https://identifiers.org/ec-code/4.1.1.50"/>
            </rdf:Bag>
          </bqbiol:hasPart>
          <bqbiol:hasPart>
            <rdf:Bag>
              <rdf:li rdf:resource="https://identifiers.org/kegg.reaction/R00178"/>
              <rdf:li rdf:resource="https://identifiers.org/kegg.reaction/R01401"/>
              <rdf:li rdf:resource="https://identifiers.org/kegg.reaction/R02869"/>
            </rdf:Bag>
          </bqbiol:hasPart>
        </rdf:Description>
      </rdf:RDF>
    </annotation>
  </reaction>
```

It is possible to mix different URIs in a given set. The following example presents two alternative annotations of the human hemoglobin, the first with ChEBI heme and the second with KEGG heme.

```
  <species id="heme" metaid="heme" compartment="C"
           hasOnlySubstanceUnits="false" boundaryCondition="false"
           constant="false">
    <annotation>
      <rdf:RDF
        xmlns:rdf="http://www.w3.org/1999/02/22-rdf-syntax-ns#"
        xmlns:bqbiol="http://biomodels.net/biology-qualifiers/"
      >
       <rdf:Description rdf:about="#heme">
         <bqbiol:hasPart>
           <rdf:Bag>
             <rdf:li rdf:resource="https://identifiers.org/uniprot/P69905"/>
             <rdf:li rdf:resource="https://identifiers.org/uniprot/P68871"/>
             <rdf:li rdf:resource="https://identifiers.org/chebi/CHEBI:17627"/>
           </rdf:Bag>
         </bqbiol:hasPart>
         <bqbiol:hasPart>
           <rdf:Bag>
             <rdf:li rdf:resource="https://identifiers.org/uniprot/P69905"/>
             <rdf:li rdf:resource="https://identifiers.org/uniprot/P68871"/>
```

```
            <rdf:li rdf:resource="https://identifiers.org/kegg.compound/C00032"/>
          </rdf:Bag>
        </bqbiol:hasPart>
      </rdf:Description>
    </rdf:RDF>
  </annotation>
</species>
```

As formally defined above it is possible to use different qualifiers in the same annotation element. The following phosphorylation is annotated by its exact KEGG counterpart and by the generic GO term "phosphorylation".

```
<reaction id="phosphorylation" metaid="phosphorylation"
          reversible="true">
  <annotation>
    <rdf:RDF
      xmlns:bqbiol="http://biomodels.net/biology-qualifiers/"
      xmlns:rdf="http://www.w3.org/1999/02/22-rdf-syntax-ns#"
    >
      <rdf:Description rdf:about="#phosphorylation">
        <bqbiol:is>
          <rdf:Bag>
            <rdf:li rdf:resource="https://identifiers.org/kegg.reaction/R03313"/>
          </rdf:Bag>
        </bqbiol:is>
        <bqbiol:isVersionOf>
          <rdf:Bag>
            <rdf:li rdf:resource="https://identifiers.org/go/GO:0016310"/>
          </rdf:Bag>
        </bqbiol:isVersionOf>
      </rdf:Description>
    </rdf:RDF>
  </annotation>
</reaction>
```

The following example demonstrates the use of nested terms to describe both that a species is in a particular compartment, and the evidence that the species belongs there:

```
<species id="S1" metaid="_000004" compartment="lysosome"
         hasOnlySubstanceUnits="false" boundaryCondition="false"
         constant="false">
  <annotation>
    <rdf:RDF xmlns:rdf="http://www.w3.org/1999/02/22-rdf-syntax-ns#"
             xmlns:bqbiol="http://biomodels.net/biology-qualifiers/"
             xmlns:bqmodel="http://biomodels.net/model-qualifiers/">
      <rdf:Description rdf:about="#_000004">
        <bqbiol:occursIn>
          <rdf:Bag>
            <rdf:li rdf:resource="https://identifiers.org/go/GO:0005764"/>
            <bqbiol:isDescribedBy>
              <rdf:Bag>
                <rdf:li rdf:resource="https://identifiers.org/pubmed/1111111"/>
              </rdf:Bag>
            </bqbiol:isDescribedBy>
            <bqbiol:isDescribedBy>
              <rdf:Bag>
                <rdf:li rdf:resource="https://identifiers.org/eco/ECO:0000004"/>
              </rdf:Bag>
            </bqbiol:isDescribedBy>
          </rdf:Bag>
        </bqbiol:occursIn>
      </rdf:Description>
    </rdf:RDF>
  </annotation>
</species>
```

In descriptive terms, the SBML species "`S1`" (with metaid "`_000004`") occurs in "`go/GO:0005764`" (the lysosome). This is described by the publication "`pubmed/1111111`", and is believed to be true because of the evidence "`eco/ECO:0000004`" (cell fractionation evidence).

The following example demonstrates the use of nested terms to describe that the annotated species represents a phosphorylated form of a particular protein:

```
<species id="S1" metaid="_000004" compartment="cell"
         hasOnlySubstanceUnits="false" boundaryCondition="false"
         constant="false">
  <annotation>
    <rdf:RDF xmlns:rdf="http://www.w3.org/1999/02/22-rdf-syntax-ns#"
             xmlns:bqbiol="http://biomodels.net/biology-qualifiers/"
      <rdf:Description rdf:about="#_000004" >
        <bqbiol:isVersionOf>
          <rdf:Bag>
            <rdf:li rdf:resource="https://identifiers.org/uniprot/P04551"/>
            <bqbiol:isVersionOf>
              <rdf:Bag>
                <rdf:li rdf:resource="https://identifiers.org/psimod/MOD:00047"/>
              </rdf:Bag>
            </bqbiol:isVersionOf>
          </rdf:Bag>
        </bqbiol:isVersionOf>
      </rdf:Description>
    </rdf:RDF>
  <annotation>
</species>
```

## 7 Example models expressed in XML using SBML

In this section, we present several examples of complete models encoded in XML using SBML Level 3.

### 7.1 A simple example application of SBML

Consider the following representation of an enzymatic reaction:

$$E + S \underset{k_{\mathrm{off}}}{\overset{k_{\mathrm{on}}}{\rightleftharpoons}} ES \xrightarrow{k_{\mathrm{cat}}} E + P$$

In our model, we use the following initial species amounts:

$$E = 5 \cdot 10^{-21} \text{ mole}$$
$$S = 10^{-20} \text{ mole}$$
$$P = 0 \text{ mole}$$
$$ES = 0 \text{ mole}$$

Note that the species quantities are initialized in terms of substance amounts rather than concentrations. We also define the following values for the kinetic constants:

$$k_{\mathrm{on}} = 1\,000\,000 \text{ litre mole}^{-1} \text{ second}^{-1}$$
$$k_{\mathrm{off}} = 0.2 \text{ second}^{-1}$$
$$k_{\mathrm{cat}} = 0.1 \text{ second}^{-1}$$

We place everything in a single compartment we call “comp” whose volume is $10^{-14}$ litres. The following is a minimal but complete SBML document encoding this model:

```
<?xml version="1.0" encoding="UTF-8"?>
<sbml xmlns="http://www.sbml.org/sbml/level3/version2/core" level="3" version="2">
  <model extentUnits="mole" timeUnits="second">
     <listOfUnitDefinitions>
       <unitDefinition id="per_second">
          <listOfUnits>
             <unit kind="second" exponent="-1" scale="0" multiplier="1"/>
          </listOfUnits>
       </unitDefinition>
       <unitDefinition id="litre_per_mole_second">
          <listOfUnits>
             <unit kind="mole"   exponent="-1" scale="0" multiplier="1"/>
             <unit kind="litre"  exponent="1"  scale="0" multiplier="1"/>
             <unit kind="second" exponent="-1" scale="0" multiplier="1"/>
          </listOfUnits>
       </unitDefinition>
     </listOfUnitDefinitions>
     <listOfCompartments>
       <compartment id="comp" size="1e-14" spatialDimensions="3" units="litre" constant="true"/>
     </listOfCompartments>
     <listOfSpecies>
       <species compartment="comp" id="E"  initialAmount="5e-21" boundaryCondition="false"
                hasOnlySubstanceUnits="false" substanceUnits="mole" constant="false"/>
       <species compartment="comp" id="S"  initialAmount="1e-20" boundaryCondition="false"
                hasOnlySubstanceUnits="false" substanceUnits="mole" constant="false"/>
       <species compartment="comp" id="P"  initialAmount="0"     boundaryCondition="false"
                hasOnlySubstanceUnits="false" substanceUnits="mole" constant="false"/>
       <species compartment="comp" id="ES" initialAmount="0"     boundaryCondition="false"
                hasOnlySubstanceUnits="false" substanceUnits="mole" constant="false"/>
     </listOfSpecies>
     <listOfReactions>
       <reaction id="veq" reversible="true">
         <listOfReactants>
            <speciesReference species="E"  stoichiometry="1" constant="true"/>
            <speciesReference species="S"  stoichiometry="1" constant="true"/>
         </listOfReactants>
```

```
         <listOfProducts>
            <speciesReference species="ES" stoichiometry="1" constant="true"/>
         </listOfProducts>
         <kineticLaw>
            <math xmlns="http://www.w3.org/1998/Math/MathML">
              <apply>
                 <times/>
                 <ci>comp</ci>
                 <apply>
                    <minus/>
                    <apply>
                      <times/>
                      <ci>kon</ci>
                      <ci>E</ci>
                      <ci>S</ci>
                    </apply>
                    <apply>
                      <times/>
                      <ci>koff</ci>
                      <ci>ES</ci>
                    </apply>
                 </apply>
              </apply>
            </math>
            <listOfLocalParameters>
              <localParameter id="kon"  value="1000000" units="litre_per_mole_second"/>
              <localParameter id="koff" value="0.2"     units="per_second"/>
            </listOfLocalParameters>
         </kineticLaw>
       </reaction>
       <reaction id="vcat" reversible="false">
          <listOfReactants>
             <speciesReference species="ES" stoichiometry="1" constant="true"/>
          </listOfReactants>
          <listOfProducts>
             <speciesReference species="E"  stoichiometry="1" constant="true"/>
             <speciesReference species="P"  stoichiometry="1" constant="true"/>
          </listOfProducts>
          <kineticLaw>
             <math xmlns="http://www.w3.org/1998/Math/MathML">
               <apply>
                  <times/>
                  <ci>comp</ci>
                  <ci>kcat</ci>
                  <ci>ES</ci>
               </apply>
             </math>
             <listOfLocalParameters>
               <localParameter id="kcat" value="0.1" units="per_second"/>
             </listOfLocalParameters>
          </kineticLaw>
       </reaction>
     </listOfReactions>
  </model>
</sbml>
```

The model features local parameter definitions in each reaction. In this case, the three parameters (`kon`, `koff`, `kcat`) all have unique identifiers and they could also have just as easily been declared global parameters. Local parameters frequently become more useful in larger models, where it may become tedious to assign unique identifiers for all the different parameters.

The example above also demonstrates the use of unit specifications throughout the model. The `model` components define the units of kinetic laws as being mole/second by virtue of the values of the attributes `extentUnits` and `timeUnits`. In the rest of the model, species, parameters and compartments are defined with appropriate units so that the mathematical formulas inside the `kineticLaw` elements work out to be mole/second.

### 7.2 A simple example using the `conversionFactor` attribute

This example involves the same enzymatic reaction as in the example of Section 7.1 on p. 113:

$$E + S \underset{k_{\text{off}}}{\overset{k_{\text{on}}}{\rightleftharpoons}} ES \xrightarrow{k_{\text{cat}}} E + P$$

In this new version of the model, we deliberately define the species with different units from the unit of reaction extent in the model. This leads to two illustrative problems: (1) the reaction rate expressions must be changed in order to reconcile the differences between the species units and the unit of reaction extent in the model, and (2) the formulas constructed for species' rate-of-change equations must use conversion factors to reconcile the differences between the units of the reaction rate expressions and the units in which the species quantities are measured.

We begin with the following new **Species** object definitions:

$$\begin{aligned} E &= 5 \cdot 10^{-18} \text{ millimole} \\ S &= 10^{-17} \text{ millimole} \\ P &= 0 \text{ gram} \\ ES &= 0 \text{ millimole} \end{aligned}$$

We keep the units of extent and time in the model the same as in the example of Section 7.1 on p. 113; that is, the overall unit of extent in the model is mole and the unit of time is second, set by assigning appropriate values to the attributes `extentUnits` and `timeUnits`, respectively, on the **Model** object definition. The differences between these and the units of the species means that we have to adjust the reaction rate expressions from their original versions in the model. In what follows, we illustrate one approach to doing so, and in Section 7.3 on p. 118 we illustrate a second approach. The method in the present section involves changing the values of the kinetic rate constants in the reaction rate formulas, while the example of Section 7.3 does not change the kinetic constants but does require the introduction of additional parameters.

The reaction rate formulas (i.e., the formulas in the `math` elements of **KineticLaw** objects) were previously

$$v_{\text{veq}} = V_{\text{comp}} \cdot (k_{\text{on}} \cdot [E] \cdot [S] - k_{\text{off}} \cdot [ES]) \tag{5}$$

$$v_{\text{vcat}} = V_{\text{comp}} \cdot k_{\text{cat}} \cdot [ES] \tag{6}$$

where $V_{\text{comp}}$ stands for the size of compartment "`comp`" in the SBML model. Recalling the values of the parameters $k_{\text{on}}$, $k_{\text{off}}$, and $k_{\text{cat}}$,

$$\begin{aligned} k_{\text{on}} &= 1\,000\,000 \text{ litre mole}^{-1} \text{ second}^{-1} \\ k_{\text{off}} &= 0.2 \text{ second}^{-1} \\ k_{\text{cat}} &= 0.1 \text{ second}^{-1} \end{aligned}$$

it becomes clear that, with the values of $E$, $S$ and $ES$ all in millimoles, Equations 5 and 6 no longer lead to units of mole/second for the reaction rates. To compensate, we change the values of the constants $k_{\text{on}}$, $k_{\text{off}}$, and $k_{\text{cat}}$ using the following simple transformations:

$$k^*_{\text{on}} = k_{\text{on}} \cdot \left(\frac{1 \text{ mole}}{1000 \text{ millimoles}}\right)^2 = 1 \text{ litre mole millimole}^{-2} \text{ second}^{-1}$$

$$k^*_{\text{off}} = k_{\text{off}} \cdot \frac{1 \text{ mole}}{1000 \text{ millimoles}} = 0.0002 \text{ mole millimole}^{-1} \text{ second}^{-1}$$

$$k^*_{\text{cat}} = k_{\text{cat}} \cdot \frac{1 \text{ mole}}{1000 \text{ millimoles}} = 0.0001 \text{ mole millimole}^{-1} \text{ second}^{-1}$$

The "mole/millimole" portion of the units are admittedly unconventional for mass-action kinetic rate constants. They are unlikely to correspond to values found in textbooks or databases. The logic of this approach is that in an actual experimental situation, with the units of the species as given in the model (presumably representing how the species are being measured), the kinetic rate constants are likely to be measured in terms of the units above. However, if that is not the case, then the approach of Section 7.3 on p. 118 may be more appropriate.

Taking these new $k^*_{\text{on}}$, $k^*_{\text{off}}$ and $k^*_{\text{cat}}$ parameters and replacing the original parameters in the reaction rate equations finally leads to the following:

$$v_{\text{veq}} = V_{\text{comp}} \cdot (k^*_{\text{on}} \cdot [E] \cdot [S] - k^*_{\text{off}} \cdot [ES]) \tag{7}$$
$$v_{\text{vcat}} = V_{\text{comp}} \cdot k^*_{\text{cat}} \cdot [ES] \tag{8}$$

Next, we turn to the rates-of-change equations for the species. There are two cases: species $S$, whose unit of substance is millimole, and species $P$, whose unit of substance is gram. We use SBML Level 3's conversion factor mechanism to effectuate the necessary transformations, following the guidelines described in Section 4.11.7 on p. 77. In the model text below, we define a default conversion factor by setting the value of the **Model** object's `conversionFactor` attribute to a parameter whose values is

$$\frac{1000 \text{ millimole}}{1 \text{ mole}}$$

Let $c_{\text{model}}$ stand for the **Model** object's `conversionFactor` attribute with the value above. The rate-of-change equation for $S$ is the following:

$$\frac{dn_S}{dt} = -c_{\text{model}} \cdot \boxed{V_{\text{comp}} \cdot (k^*_{\text{on}} \cdot [E] \cdot [S] - k^*_{\text{off}} \cdot [ES])} \tag{9}$$

The portion inside the gray box in Equation 9 is simply Equation 7, and its value will have the unit mole/second. Multiplying this by $c_{\text{model}}$ will produce a result in millimole/second. The stoichiometry of species $S$ in the reaction is "`1`", but it is a reactant, thus the need for the negative sign.

For species $P$, we need a different conversion factor, to convert between the units of gram and mole. We accomplish this by setting a value for the **Species** object's `conversionFactor` attribute. By virtue of being defined on the **Species** object for $P$, this conversion factor value overrides the global value defined on the **Model** object. Let $c_P$ represent this conversion factor. The equation for the rate-of-change of $P$ is the following:

$$\frac{dn_P}{dt} = c_P \cdot \boxed{V_{\text{comp}} \cdot k^*_{\text{cat}} \cdot [ES]} \tag{10}$$

The portion inside the gray box in Equation 10 is simply Equation 8, with a value in mole/second. Multiplying by the conversion factor "`convertToGram`" defined in the model below will yield gram/second. The species $P$ is a product, and its stoichiometry is "`1`"; thus, the right-hand side has a positive sign.

The following is the SBML encoding of this model:

```
<?xml version="1.0" encoding="UTF-8" ?>
<sbml xmlns="http://www.sbml.org/sbml/level3/version2/core" level="3" version="2">
 <model extentUnits="mole" timeUnits="second" conversionFactor="convertToMilliMole">
   <listOfUnitDefinitions>
     <unitDefinition id="mole_per_millimole_second">
       <listOfUnits>
        <unit kind="mole"   exponent="1"  scale="0"  multiplier="1" />
        <unit kind="mole"   exponent="-1" scale="-3" multiplier="1" />
        <unit kind="second" exponent="-1" scale="0"  multiplier="1" />
       </listOfUnits>
     </unitDefinition>
     <unitDefinition id="mole_litre_per_millimole_sq_second">
       <listOfUnits>
         <unit kind="mole"   exponent="1"  scale="0"  multiplier="1" />
         <unit kind="litre"  exponent="1"  scale="0"  multiplier="1" />
         <unit kind="mole"   exponent="-2" scale="-3" multiplier="1" />
         <unit kind="second" exponent="-1" scale="0"  multiplier="1" />
        </listOfUnits>
     </unitDefinition>
     <unitDefinition id="millimole">
       <listOfUnits>
```

```
      <unit kind="mole" exponent="1" scale="-3" multiplier="1"/>
    </listOfUnits>
  </unitDefinition>
  <unitDefinition id="gram_per_mole">
    <listOfUnits>
     <unit kind="gram" exponent="1"  scale="0" multiplier="1"/>
     <unit kind="mole" exponent="-1" scale="0" multiplier="1"/>
    </listOfUnits>
  </unitDefinition>
  <unitDefinition id="millimole_per_mole">
    <listOfUnits>
     <unit kind="mole" exponent="1"  scale="-3" multiplier="1"/>
     <unit kind="mole" exponent="-1" scale="0" multiplier="1"/>
    </listOfUnits>
  </unitDefinition>
</listOfUnitDefinitions>
<listOfCompartments>
  <compartment id="comp" size="1e-14" spatialDimensions="3" units="litre" constant="true" />
</listOfCompartments>
<listOfSpecies>
  <species compartment="comp" id="ES" initialAmount="0" boundaryCondition="false"
           hasOnlySubstanceUnits="false" substanceUnits="millimole" constant="false" />
  <species compartment="comp" id="P" initialAmount="0" boundaryCondition="false"
           hasOnlySubstanceUnits="false" substanceUnits="gram" constant="false"
           conversionFactor="convertToGram"/>
  <species compartment="comp" id="S" initialAmount="1e-17" boundaryCondition="false"
           hasOnlySubstanceUnits="false" substanceUnits="millimole" constant="false" />
  <species compartment="comp" id="E" initialAmount="5e-18" boundaryCondition="false"
           hasOnlySubstanceUnits="false" substanceUnits="millimole" constant="false" />
</listOfSpecies>
<listOfParameters>
  <parameter id="convertToMilliMole" value="1000" units="millimole_per_mole" constant="true"/>
  <parameter id="convertToGram"      value="180"  units="gram_per_mole"      constant="true"/>
</listOfParameters>
<listOfReactions>
  <reaction id="veq" reversible="true">
    <listOfReactants>
      <speciesReference species="E" stoichiometry="1" constant="true" />
      <speciesReference species="S" stoichiometry="1" constant="true" />
    </listOfReactants>
    <listOfProducts>
      <speciesReference species="ES" stoichiometry="1" constant="true" />
    </listOfProducts>
    <kineticLaw>
      <math xmlns="http://www.w3.org/1998/Math/MathML">
        <apply>
          <times />
          <ci>comp</ci>
          <apply>
            <minus />
            <apply> <times /> <ci> kon </ci> <ci> E </ci> <ci> S </ci> </apply>
            <apply> <times /> <ci> koff </ci> <ci> ES </ci> </apply>
          </apply>
        </apply>
      </math>
      <listOfLocalParameters>
        <localParameter id="kon"  value="1"      units="mole_litre_per_millimole_sq_second" />
        <localParameter id="koff" value="0.0002" units="mole_per_millimole_second" />
      </listOfLocalParameters>
    </kineticLaw>
  </reaction>
  <reaction id="vcat" reversible="false">
    <listOfReactants>
      <speciesReference species="ES" stoichiometry="1" constant="true" />
    </listOfReactants>
    <listOfProducts>
      <speciesReference species="E" stoichiometry="1" constant="true" />
      <speciesReference species="P" stoichiometry="1" constant="true" />
    </listOfProducts>
```

```
          <kineticLaw>
            <math xmlns="http://www.w3.org/1998/Math/MathML">
              <apply>
                <times /> <ci> comp </ci> <ci> kcat </ci> <ci> ES </ci>
              </apply>
            </math>
            <listOfLocalParameters>
              <localParameter id="kcat" value="0.0001" units="mole_per_millimole_second" />
            </listOfLocalParameters>
          </kineticLaw>
        </reaction>
      </listOfReactions>
    </model>
  </sbml>
```

#### 7.3 An alternative formulation of the conversionFactor example

Here we present an alternative formulation of the model from the previous section. Once again, it involves the same enzymatic reaction as in the example of Section 7.1 on p. 113:

$$E + S \underset{k_{\text{off}}}{\overset{k_{\text{on}}}{\rightleftharpoons}} ES \xrightarrow{k_{\text{cat}}} E + P$$

As in Section 7.2 on p. 115, we define the overall unit of extent on the model to be mole and the unit of time to be second; this means the unit of reaction rates is mole/second as before. We also set the initial amounts and units as in the previous section:

$$E = 5 \cdot 10^{-18} \text{ millimole}$$
$$S = 10^{-17} \text{ millimole}$$
$$P = 0 \text{ gram}$$
$$ES = 0 \text{ millimole}$$

Unlike in the previous section's model, however, here we retain the values of the kinetic constants as they were originally in the model of Section 7.1 on p. 113:

$$k_{\text{on}} = 1\,000\,000 \text{ litre mole}^{-1} \text{ second}^{-1}$$
$$k_{\text{off}} = 0.2 \text{ second}^{-1}$$
$$k_{\text{cat}} = 0.1 \text{ second}^{-1}$$

We take a different approach to adjusting the reaction rate expressions to account for the fact that the concentrations of the species as they appear in the **KineticLaw** elements are in units of millimole/litre, while the unit of reaction extent is mole and reaction rates are in mole/second. Our approach here is to introduce constants into the reaction rate expressions to convert the substance units to mole and multiply each occurence of a concentration by that constant. A separate constant is necessary for each **Species** object appearing in a **KineticLaw** object, although it turns out that in the particular situation under consideration here, the constants are all identical:

$$c_E = c_S = c_{ES} = 10^{-3} \text{ mole millimole}^{-1}$$

Applying this approach, the reaction rate equations become the following:

$$v_{\text{veq}} = V_{\text{comp}} \cdot (k_{\text{on}} \cdot [E] \cdot c_E \cdot [S] \cdot c_S - k_{\text{off}} \cdot [ES] \cdot c_{ES})$$
$$v_{\text{vcat}} = V_{\text{comp}} \cdot k_{\text{cat}} \cdot [ES] \cdot c_{ES}$$

where again $V_{\text{comp}}$ stands for the size of compartment called "comp" in the SBML model. We construct the rate-of-change equations for the each species using the guidelines described in Section 4.11.7 on p. 77, and in this case, the equations for species $S$ and $P$ are

$$\frac{dn_S}{dt} = -c_{\text{model}} \cdot V_{\text{comp}} \cdot (k_{\text{on}} \cdot [E] \cdot c_E \cdot [S] \cdot c_S - k_{\text{off}} \cdot [ES] \cdot c_{ES})$$

$$\frac{dn_P}{dt} = c_P \cdot V_{\text{comp}} \cdot k_{\text{cat}} \cdot [ES] \cdot c_{ES}$$

where again $c_{\text{model}}$ stands for the value of the **Model** object's `conversionFactor` attribute and $c_P$ is the value of the `conversionFactor` attribute of the **Species** object definition for $P$.

The SBML encoding of this model is given below:

```
<?xml version="1.0" encoding="UTF-8" ?>
<sbml xmlns="http://www.sbml.org/sbml/level3/version2/core" level="3" version="2">
 <model extentUnits="mole" timeUnits="second" conversionFactor="convertToMilliMole">
   <listOfUnitDefinitions>
     <unitDefinition id="per_second">
       <listOfUnits>
        <unit kind="second" exponent="-1" scale="0"  multiplier="1" />
       </listOfUnits>
     </unitDefinition>
     <unitDefinition id="litre_per_mole_second">
       <listOfUnits>
         <unit kind="mole"   exponent="-1" scale="0"  multiplier="1" />
         <unit kind="litre"  exponent="1"  scale="0"  multiplier="1" />
         <unit kind="second" exponent="-1" scale="0"  multiplier="1" />
        </listOfUnits>
     </unitDefinition>
     <unitDefinition id="millimole">
       <listOfUnits>
         <unit kind="mole" exponent="1" scale="-3" multiplier="1"/>
        </listOfUnits>
     </unitDefinition>
     <unitDefinition id="gram_per_mole">
       <listOfUnits>
        <unit kind="gram" exponent="1"  scale="0" multiplier="1"/>
        <unit kind="mole" exponent="-1" scale="0" multiplier="1"/>
       </listOfUnits>
     </unitDefinition>
     <unitDefinition id="mole_per_millimole">
       <listOfUnits>
        <unit kind="mole" exponent="1"  scale="0"  multiplier="1"/>
        <unit kind="mole" exponent="-1" scale="-3" multiplier="1"/>
       </listOfUnits>
     </unitDefinition>
     <unitDefinition id="millimole_per_mole">
       <listOfUnits>
        <unit kind="mole" exponent="1"  scale="-3" multiplier="1"/>
        <unit kind="mole" exponent="-1" scale="0"  multiplier="1"/>
       </listOfUnits>
     </unitDefinition>
   </listOfUnitDefinitions>
   <listOfCompartments>
     <compartment id="comp" size="1e-14" spatialDimensions="3" units="litre" constant="true" />
   </listOfCompartments>
   <listOfSpecies>
     <species compartment="comp" id="ES" initialAmount="0" boundaryCondition="false"
              hasOnlySubstanceUnits="false" substanceUnits="millimole" constant="false" />
     <species compartment="comp" id="P" initialAmount="0" boundaryCondition="false"
              hasOnlySubstanceUnits="false" substanceUnits="gram" constant="false"
              conversionFactor="convertToGram"/>
     <species compartment="comp" id="S" initialAmount="1e-17" boundaryCondition="false"
              hasOnlySubstanceUnits="false" substanceUnits="millimole" constant="false" />
     <species compartment="comp" id="E" initialAmount="5e-18" boundaryCondition="false"
              hasOnlySubstanceUnits="false" substanceUnits="millimole" constant="false" />
   </listOfSpecies>
   <listOfParameters>
     <parameter id="convertToMilliMole" value="1000" units="millimole_per_mole" constant="true"/>
     <parameter id="convertToGram"      value="180"  units="gram_per_mole"      constant="true"/>
     <parameter id="c_e"   value="1e-3"    units="mole_per_millimole" constant="true"/>
     <parameter id="c_s"   value="1e-3"    units="mole_per_millimole" constant="true"/>
     <parameter id="c_es"  value="1e-3"    units="mole_per_millimole" constant="true"/>
   </listOfParameters>
   <listOfReactions>
     <reaction id="veq" reversible="true">
       <listOfReactants>
```

```
        <speciesReference species="E" stoichiometry="1" constant="true" />
        <speciesReference species="S" stoichiometry="1" constant="true" />
      </listOfReactants>
      <listOfProducts>
        <speciesReference species="ES" stoichiometry="1" constant="true" />
      </listOfProducts>
      <kineticLaw>
        <math xmlns="http://www.w3.org/1998/Math/MathML">
          <apply>
            <times />
            <ci>comp</ci>
            <apply>
              <minus />
              <apply>
                <times />
                <ci> kon </ci>
                <ci> E </ci>
                <ci> c_e </ci>
                <ci> S </ci>
                <ci> c_s </ci>
              </apply>
              <apply>
                <times />
                <ci> koff </ci>
                <ci> ES </ci>
                <ci> c_es </ci>
              </apply>
            </apply>
          </apply>
        </math>
        <listOfLocalParameters>
          <localParameter id="kon"  value="1000000" units="litre_per_mole_second" />
          <localParameter id="koff" value="0.2"     units="per_second" />
        </listOfLocalParameters>
      </kineticLaw>
    </reaction>
    <reaction id="vcat" reversible="false">
      <listOfReactants>
        <speciesReference species="ES" stoichiometry="1" constant="true" />
      </listOfReactants>
      <listOfProducts>
        <speciesReference species="E" stoichiometry="1" constant="true" />
        <speciesReference species="P" stoichiometry="1" constant="true" />
      </listOfProducts>
      <kineticLaw>
        <math xmlns="http://www.w3.org/1998/Math/MathML">
          <apply>
            <times />
            <ci> comp </ci>
            <ci> kcat </ci>
            <ci> ES </ci>
            <ci> c_es </ci>
          </apply>
        </math>
        <listOfLocalParameters>
          <localParameter id="kcat" value="0.1" units="per_second" />
        </listOfLocalParameters>
      </kineticLaw>
    </reaction>
  </listOfReactions>
</model> </sbml>
```

### 7.4 Example of a discrete version of a simple dimerization reaction

*(SBO annotations for this model contributed by Lukas Endler, EMBL-EBI, Cambridge, UK.)*

This example illustrates subtle differences between models formulated for use in a continuous simulation framework (e.g., using differential equations) and those intended for a discrete simulation framework. The model shown here is suitable for use with a discrete stochastic simulation algorithm of the sort developed by Gillespie (1977). In such an approach, species are described in terms of molecular counts and simulation proceeds by computing the probability of the time and identity of the next reaction, then updating the species amounts appropriately.

The model involves a simple dimerization reaction for a protein named $P$:

$$2P \leftrightarrow P_2$$

The SBML representation is shown below. There are several notable points. First, species $P$ and $P_2$ (represented by "`P`" and "`P2`", respectively) are declared to be always in terms of discrete amounts by using the flag `hasOnlySubstanceUnits`="`true`" on the **Species** object definitions. This indicates that when the species identifiers appear in mathematical formulas, their values have units of *substance amount*, not $\{$*substance amount*$\}/$*size*. A second point is that, as a result, the corresponding **KineticLaw** formulas do not need volume corrections. In Gillespie's approach, the constants in the rate expressions (here, $c_1$ and $c_2$, represented in the SBML model by `c1` and `c2`, respectively) contain a contribution from the kinetic constants of the reaction and the size of the compartment in which the reactions take place. This is a convention commonly adopted by stochastic modelers, but is in no way essential—it is perfectly reasonable to factor volume out of the rate constants, and in certain situations it may be desirable to do so (e.g., for models having time-varying compartment volume), but due to the use of substance units, it must be done differently compared to the deterministic case. Third, although the reaction is reversible, it is encoded as two separate irreversible reactions, one each for the forward and reverse directions, as averaging over the reactions will affect the stochasticity. Finally, note that the rate expression for the forward reaction is a second-order mass-action reaction, but it is the *discrete* formulation of such a reaction rate (Gillespie, 1977).

```
<?xml version="1.0" encoding="UTF-8"?>
<sbml xmlns="http://www.sbml.org/sbml/level3/version2/core" level="3" version="2">
    <model id="dimerization" substanceUnits="item" timeUnits="second"
           volumeUnits="litre" extentUnits="item">
        <listOfUnitDefinitions>
            <unitDefinition id="per_second">
                <listOfUnits>
                    <unit kind="second" exponent="-1" scale="0" multiplier="1"/>
                </listOfUnits>
            </unitDefinition>
            <unitDefinition id="per_item_per_second">
                <listOfUnits>
                    <unit kind="item"   exponent="-1" scale="0" multiplier="1"/>
                    <unit kind="second" exponent="-1" scale="0" multiplier="1"/>
                </listOfUnits>
            </unitDefinition>
        </listOfUnitDefinitions>
        <listOfCompartments>
            <compartment id="Cell" size="1e-15" spatialDimensions="3"
                         constant="true" sboTerm="SBO:0000290"/>
        </listOfCompartments>
        <listOfSpecies>
            <species id="P"  compartment="Cell" initialAmount="301"
                             hasOnlySubstanceUnits="true" boundaryCondition="false"
                             constant="false" sboTerm="SBO:0000252"/>
            <species id="P2" compartment="Cell" initialAmount="0"
                             hasOnlySubstanceUnits="true" boundaryCondition="false"
                             constant="false" sboTerm="SBO:0000420"/>
        </listOfSpecies>
        <listOfReactions>
            <reaction id="Dimerization" reversible="false" sboTerm="SBO:0000177">
                <listOfReactants>
                    <speciesReference species="P" stoichiometry="2" constant="true"
                                      sboTerm="SBO:0000010"/>
                </listOfReactants>
                <listOfProducts>
                    <speciesReference species="P2" stoichiometry="1" constant="true"
                                      sboTerm="SBO:0000011"/>
                </listOfProducts>
```

```
                    <kineticLaw sboTerm="SBO:0000142">
                        <math xmlns="http://www.w3.org/1998/Math/MathML"
                              xmlns:sbml="http://www.sbml.org/sbml/level3/version2/core">
                            <apply>
                                <divide/>
                                <apply>
                                    <times/>
                                    <ci> c1 </ci>
                                    <ci> P </ci>
                                    <apply>
                                        <minus/>
                                        <ci> P </ci>
                                        <cn type="integer" sbml:units="item"> 1 </cn>
                                    </apply>
                                </apply>
                                <cn type="integer" sbml:units="dimensionless"> 2 </cn>
                            </apply>
                        </math>
                        <listOfLocalParameters>
                            <localParameter id="c1" value="0.00166" units="per_item_per_second"
                                            sboTerm="SBO:0000067"/>
                        </listOfLocalParameters>
                    </kineticLaw>
                </reaction>
                <reaction id="Dissociation" reversible="false" sboTerm="SBO:0000180">
                    <listOfReactants>
                        <speciesReference species="P2" stoichiometry="1" constant="true"
                                          sboTerm="SBO:0000010"/>
                    </listOfReactants>
                    <listOfProducts>
                        <speciesReference species="P" stoichiometry="2" constant="true"
                                          sboTerm="SBO:0000011"/>
                    </listOfProducts>
                    <kineticLaw sboTerm="SBO:0000141">
                        <math xmlns="http://www.w3.org/1998/Math/MathML">
                            <apply>
                                <times/>
                                <ci> c2 </ci>
                                <ci> P </ci>
                            </apply>
                        </math>
                        <listOfLocalParameters>
                            <localParameter id="c2" value="0.2" units="per_second"
                                            sboTerm="SBO:0000066"/>
                        </listOfLocalParameters>
                    </kineticLaw>
                </reaction>
            </listOfReactions>
        </model>
    </sbml>
```

This example also illustrates the need to provide additional information in a model so that software tools using different mathematical frameworks can properly interpret it. In this case, a simulation tool designed for continuous ODE-based simulation would likely misinterpret the model (in particular the reaction rate formulas), unless it deduced that a discrete stochastic simulation was intended. One of the purposes of SBO annotations (Section 5 on p. 91) is to enable such interpretation without the need for deduction. However, the interpretation of the model is essentially the same irrespective of whether the model is to be simulated in a deterministic or stochastic manner, and a properly SBML-compliant deterministic simulator will in most cases correctly simulate the continuous deterministic approximation of the stochastic model even if it has no stochastic simulation capability.

The interpretation of rate laws for stochastic models is similar to, yet different from, that of deterministic models. Taking the first reaction as an example, the rate law is $c_1 P(P-1)/2$ reaction events per second. In the continuous deterministic case, the interpretation of this is that the extent of the reaction in time $dt$ is $[c_1 P(P-1)/2]dt$ (and this leads naturally to the usual ODE formulation of the model). In the stochastic case, the interpretation is that the *propensity* (or *rate*, or *hazard*) of the reaction is $c_1 P(P-1)/2$. That is, the *probability* of a single reaction event occurring in time $dt$ is $[c_1 P(P-1)/2]dt$ (and note that the *expected* extent of the reaction will be $[c_1 P(P-1)/2]dt$). This interpretation leads to a Markov jump process for the system dynamics, where the inter-event times are exponentially distributed. Such dynamics can be simulated using a discrete event simulation algorithm such as the *Gillespie algorithm*. In this case, the algorithm for simulating the model can be described as follows:

1. Initialize $t := 0,\ c_1 := 0.00166,\ c_2 := 0.2,\ P := 301,\ P_2 := 0$
2. Compute $h_1 := c_1 P(P-1)/2,\ h_2 := c_2 P_2$
3. Compute $h_0 = h_1 + h_2$
4. Simulate $t' \sim \text{Exp}(h_0)$ and set $t := t + t'$
5. With probability $h_1/h_0$ set $P := P - 2,\ P_2 := P_2 + 1$, otherwise set $P := P + 2,\ P_2 := P_2 - 1$.
6. Output $t,\ P,\ P_2$
7. If $t < T_{\text{max}}$, return to step 2, otherwise stop.

Although this is a simulation algorithm is a very practical way of describing how to construct exact realizations of the Markov jump process corresponding to the discrete stochastic kinetic model, it is not a concise mathematical description. Such a description can be provided by writing the model as a time change of a pair of independent unit Poisson processes. Let $N_1(t)$ and $N_2(t)$ be the counting functions of these processes, so that for each $i = 1, 2,\ t > 0,\ N_i(t) \sim \text{Poisson}(t)$. Then, writing $P(t)$ and $P_2(t)$ for the numbers of molecules of $P$ and $P_2$ at time $t$, respectively, we have that the stochastic process $\{P(t), P_2(t) \,|\, t > 0\}$ satisfies the stochastic integral equation

$$
\begin{aligned}
P_2(t) &= N_1\left(\int_0^t c_1 \frac{P(\tau)[P(\tau)-1]}{2} d\tau\right) - N_2\left(\int_0^t c_2 P_2(\tau) d\tau\right) \\
P(t) &= 301 - 2P_2(t).
\end{aligned}
$$

The above representation is arguably the most useful for mathematical analysis of the stochastic model; see Ball et al. (2006) for details. Another popular representation is the so-called chemical Master equation (CME) for the probability distribution of the possible states at all times (Gillespie, 1992). In this case, since there are 151 possible states of the system (corresponding to the 151 possible values of $P_2$), the CME consists of 151 coupled ODEs,

$$
\frac{d}{dt} p(P, P_2, t) = \begin{cases}
-\frac{c_1}{2} \times 301 \times 299 p(301, 0, t) + c_2 p(299, 1, t), & P = 301,\ P_2 = 0, \\
\frac{c_1}{2}(P+2)(P+1)p(P+2, P_2 - 1, t) - \frac{c_1}{2} P(P-1)p(P, P_2, t) & P = 301 - x,\ P_2 = x, \\
\quad + c_2(P_2+1)p(P-2, P_2+1, t) - c_2 P_2 p(P, P_2, t), & \quad x = 1, 2, \ldots, 149, \\
\frac{c_1}{2} \times 2 \times 3 p(3, 149, t) - c_2 \times 150 p(1, 150, t), & P = 1,\ P_2 = 150,
\end{cases}
$$

where $p(P, P_2, t)$ denotes the probability that there are $P$ molecules of $P$ and $P_2$ molecules of $P_2$ at time $t$, and the ODEs are subject to the initial conditions

$$
p(301, 0, 0) = 1,\ p(301 - 2x, x, 0) = 0,\ x = 1, 2, \ldots, 150.
$$

See Evans et al. (2008) for further examples of discrete stochastic kinetic models encoded in SBML and Wilkinson (2006) for an introduction to discrete stochastic modeling using SBML.

### 7.5 Example involving assignment rules

This section contains a model that simulates a system containing a fast reaction. This model uses rules to express the mathematics of the fast reaction explicitly rather than using the old `fast` attribute on a reaction element. The system modeled is

$$X_0 \xrightarrow{k_1[X_0]} S_1$$

$$S_1 \xleftrightarrow{k_\mathrm{f}[S_1]-k_\mathrm{r}[S_2]} S_2$$

$$S_2 \xrightarrow{k_2[S_2]} X_1$$

$$k_1 = 0.1, \quad k_2 = 0.15, \quad k_\mathrm{f} = K_\mathrm{eq}10000, \quad k_\mathrm{r} = 10000, \quad K_\mathrm{eq} = 2.5.$$

where $[X_0]$, $[X_1]$, $[S_1]$, and $[S_2]$ are species in concentration units, and $k_1$, $k_2$, $k_\mathrm{f}$, $k_\mathrm{r}$, and $K_\mathrm{eq}$ are parameters. This system of reactions can be approximated with the following new system:

$$X_0 \xrightarrow{k_1[X_0]} T$$

$$T \xrightarrow{k_2[S_2]} X_1$$

$$[S_1] = \frac{[T]}{1 + K_\mathrm{eq}}$$

$$[S_2] = K_\mathrm{eq}[S_1]$$

where $T$ is a new species. The following example SBML model encodes the second system.

```xml
<?xml version="1.0" encoding="UTF-8"?>
<sbml xmlns="http://www.sbml.org/sbml/level3/version2/core" level="3" version="2">
    <model volumeUnits="litre" substanceUnits="mole" timeUnits="second" extentUnits="mole">
        <listOfUnitDefinitions>
            <unitDefinition id="per_second">
                <listOfUnits>
                    <unit kind="second" exponent="-1" scale="0" multiplier="1"/>
                </listOfUnits>
            </unitDefinition>
        </listOfUnitDefinitions>
        <listOfCompartments>
            <compartment id="cell" size="1" spatialDimensions="3" constant="true"/>
        </listOfCompartments>
        <listOfSpecies>
            <species id="X0" compartment="cell" initialConcentration="1" constant="false"
                     hasOnlySubstanceUnits="false" boundaryCondition="false"/>
            <species id="X1" compartment="cell" initialConcentration="0" constant="false"
                     hasOnlySubstanceUnits="false" boundaryCondition="false"/>
            <species id="T"  compartment="cell" initialConcentration="0" constant="false"
                     hasOnlySubstanceUnits="false" boundaryCondition="false"/>
            <species id="S1" compartment="cell" initialConcentration="0" constant="false"
                     hasOnlySubstanceUnits="false" boundaryCondition="false"/>
            <species id="S2" compartment="cell" initialConcentration="0" constant="false"
                     hasOnlySubstanceUnits="false" boundaryCondition="false"/>
        </listOfSpecies>
        <listOfParameters>
            <parameter id="Keq" value="2.5" units="dimensionless" constant="true"/>
        </listOfParameters>
        <listOfRules>
            <assignmentRule variable="S1">
                <math xmlns="http://www.w3.org/1998/Math/MathML"
                      xmlns:sbml="http://www.sbml.org/sbml/level3/version2/core">
                    <apply>
                        <divide/>
```

```
                        <ci> T </ci>
                        <apply>
                            <plus/>
                            <cn sbml:units="dimensionless"> 1 </cn>
                            <ci> Keq </ci>
                        </apply>
                    </apply>
                </math>
            </assignmentRule>
            <assignmentRule variable="S2">
                <math xmlns="http://www.w3.org/1998/Math/MathML">
                    <apply>
                        <times/>
                        <ci> Keq </ci>
                        <ci> S1 </ci>
                    </apply>
                </math>
            </assignmentRule>
        </listOfRules>
        <listOfReactions>
            <reaction id="in" reversible="false">
                <listOfReactants>
                    <speciesReference species="X0" stoichiometry="1" constant="true"/>
                </listOfReactants>
                <listOfProducts>
                    <speciesReference species="T" stoichiometry="1" constant="true"/>
                </listOfProducts>
                <kineticLaw>
                    <math xmlns="http://www.w3.org/1998/Math/MathML">
                        <apply>
                            <times/>
                            <ci> k1 </ci>
                            <ci> X0 </ci>
                            <ci> cell </ci>
                        </apply>
                    </math>
                    <listOfLocalParameters>
                        <localParameter id="k1" value="0.1" units="per_second"/>
                    </listOfLocalParameters>
                </kineticLaw>
            </reaction>
            <reaction id="out" reversible="false">
                <listOfReactants>
                    <speciesReference species="T" stoichiometry="1" constant="true"/>
                </listOfReactants>
                <listOfProducts>
                    <speciesReference species="X1" stoichiometry="1" constant="true"/>
                </listOfProducts>
                <listOfModifiers>
                    <modifierSpeciesReference species="S2"/>
                </listOfModifiers>
                <kineticLaw>
                    <math xmlns="http://www.w3.org/1998/Math/MathML">
                        <apply>
                            <times/>
                            <ci> k2 </ci>
                            <ci> S2 </ci>
                            <ci> cell </ci>
                        </apply>
                    </math>
                    <listOfLocalParameters>
                        <localParameter id="k2" value="0.15" units="per_second"/>
                    </listOfLocalParameters>
                </kineticLaw>
            </reaction>
        </listOfReactions>
    </model>
</sbml>
```

### 7.6 Example involving algebraic rules

This section contains an example model that contains two **AlgebraicRule** objects that are necessary to determine the values of two variables within the model. In this particular case, the rules cannot be rewritten in terms of **AssignmentRule**. This example illustrates a more rigorous analysis of the enzymatic reaction given in the example of Section 7.1 on p. 113.

$$E + S \underset{k1_{\mathrm{off}}}{\overset{k1_{\mathrm{on}}}{\rightleftharpoons}} ES \xrightarrow{k2} E + P$$

In this example, we describe a quasi-steady-state approximation of the enzymatic reaction equation shown above. It is based on two assumptions. First, the rate at which the concentration of the substrate bound enzyme ($[ES]$) changes is assumed to be slow compared to the rate of change of concentration of both the substrate ($[S]$) and product ($[P]$). Second, the total concentration of the enzyme is assumed to stay constant over time. This means we can assume the concentration of $[ES]$ and $[E]$ are not governed by the reactions, and so some other equations must be used to determine the values of these concentrations in order to be able to simulate the model.

Applying the first assumption means that the rate of change of $[ES]$ should be set to zero:

$$\frac{d[ES]}{dt} = k1_{\mathrm{on}} \cdot [E] \cdot [S] - (k1_{\mathrm{off}} + k2) \cdot [ES] = 0$$

The second assumption can be written as

$$[E_{\mathrm{total}}] = [E] + [ES]$$

which, after rearranging, becomes

$$[E_{\mathrm{total}}] - ([E] + [ES]) = 0$$

Thus, we have two algebraic rules that must be applied to determine the values of $[E]$ and $[ES]$. The SBML encoding of this model is given below. Note that the species $E$ and $ES$ have their `boundaryCondition` attribute set to "`true`". This means that a simulation tool should not construct equations for them based on the reactions in the system. Their values are instead set using the rules in the model. Also, the model uses a dummy species $E_{\mathrm{total}}$ with its `constant` attribute set to "`true`"; its role is to assign the total concentration of the enzyme in the model. This could just as easily have been done using a parameter instead of a constant dummy species, but we use the latter approach as an illustration.

```
<?xml version="1.0" encoding="UTF-8" ?>
<sbml xmlns="http://www.sbml.org/sbml/level3/version2/core" level="3" version="2">
  <model substanceUnits="mole" volumeUnits="litre" timeUnits="second" extentUnits="mole">
    <listOfUnitDefinitions>
    <unitDefinition id="per_second">
      <listOfUnits>
        <unit kind="second" exponent="-1" scale="0" multiplier="1"/>
      </listOfUnits>
    </unitDefinition>
    <unitDefinition id="mole_per_litre">
      <listOfUnits>
        <unit kind="mole"  exponent="1"  scale="0" multiplier="1"/>
        <unit kind="litre" exponent="-1" scale="0" multiplier="1"/>
      </listOfUnits>
    </unitDefinition>
    <unitDefinition id="litre_per_mole_per_second">
      <listOfUnits>
        <unit kind="litre"  exponent="1"  scale="0" multiplier="1"/>
        <unit kind="mole"   exponent="-1" scale="0" multiplier="1"/>
        <unit kind="second" exponent="-1" scale="0" multiplier="1"/>
      </listOfUnits>
    </unitDefinition>
    </listOfUnitDefinitions>
    <listOfCompartments>
      <compartment id="cell" size="1" spatialDimensions="3" constant="true"/>
```

```
</listOfCompartments>
<listOfSpecies>
  <species id="E" compartment="cell" initialConcentration="0.5" constant="false"
           hasOnlySubstanceUnits="false" boundaryCondition="true"/>
  <species id="S"  compartment="cell" initialConcentration="1.0" constant="false"
           hasOnlySubstanceUnits="false" boundaryCondition="false"/>
  <species id="ES" compartment="cell" initialConcentration="0.5" constant="false"
           hasOnlySubstanceUnits="false" boundaryCondition="true"/>
  <species id="P" compartment="cell" initialConcentration="0" constant="false"
           hasOnlySubstanceUnits="false" boundaryCondition="false"/>
  <species id="E_total" compartment="cell" initialConcentration="1.0" constant="true"
           hasOnlySubstanceUnits="false" boundaryCondition="true"/>
</listOfSpecies>
<listOfParameters>
  <parameter id="k1_on"  value="1"   units="litre_per_mole_per_second" constant="true"/>
  <parameter id="k1_off" value="0.5" units="per_second" constant="true"/>
  <parameter id="k2"     value="0.5" units="per_second" constant="true"/>
</listOfParameters>
<listOfRules>
  <algebraicRule>
    <math xmlns="http://www.w3.org/1998/Math/MathML">
      <apply>
      <minus/>
        <apply>
          <times/> <ci> k1_on </ci> <ci> E </ci> <ci> S </ci>
        </apply>
        <apply>
          <times/>
          <apply> <plus/> <ci> k1_off </ci> <ci> k2 </ci> </apply>
          <ci> ES </ci>
        </apply>
      </apply>
    </math>
  </algebraicRule>
  <algebraicRule>
    <math xmlns="http://www.w3.org/1998/Math/MathML">
      <apply>
      <minus/>
        <apply> <plus/> <ci> E </ci> <ci> ES </ci> </apply>
        <ci> E_total </ci>
      </apply>
    </math>
  </algebraicRule>
</listOfRules>
<listOfReactions>
  <reaction id="r1" reversible="true">
    <listOfReactants>
      <speciesReference species="E" stoichiometry="1" constant="true"/>
      <speciesReference species="S" stoichiometry="1" constant="true"/>
    </listOfReactants>
    <listOfProducts>
      <speciesReference species="ES" stoichiometry="1" constant="true"/>
    </listOfProducts>
    <kineticLaw>
      <math xmlns="http://www.w3.org/1998/Math/MathML">
        <apply>
        <times/>
          <ci> cell </ci>
          <apply>
          <minus/>
            <apply> <times/> <ci> k1_on </ci> <ci> E </ci> <ci> S </ci> </apply>
            <apply> <times/> <ci> k1_off </ci> <ci> ES </ci> </apply>
          </apply>
        </apply>
      </math>
    </kineticLaw>
  </reaction>
  <reaction id="r2" reversible="false">
    <listOfReactants>
```

```
            <speciesReference species="ES" stoichiometry="1" constant="true"/>
          </listOfReactants>
          <listOfProducts>
            <speciesReference species="E" stoichiometry="1" constant="true"/>
            <speciesReference species="P" stoichiometry="1" constant="true"/>
          </listOfProducts>
          <kineticLaw>
            <math xmlns="http://www.w3.org/1998/Math/MathML">
              <apply> <times/> <ci> cell </ci> <ci> k2 </ci> <ci> ES </ci> </apply>
            </math>
          </kineticLaw>
        </reaction>
      </listOfReactions>
    </model>
  </sbml>
```

### 7.7 Example with combinations of boundaryCondition and constant values on Species with RateRule objects

In this section, we discuss a model that includes four species, each with a different combination of values for their `boundaryCondition` and `constant` attributes. The model represents a hypothetical system containing one reaction,

$$S_1 + S_2 \xrightarrow{k_1[S_1][S_2][S_3]} S_4$$

where $S_3$ is a species that catalyzes the conversion of species $S_1$ and $S_2$ into $S_4$. Species $S_1$ and $S_2$ are on the boundary of the system (i.e., $S_1$ and $S_2$ are reactants but their values are not determined by kinetic laws). The value of $S_1$ in the system is determined over time by the rate rule:

$$\frac{d[S_1]}{dt} = k_2$$

The species $S_2$ and $S_3$ are not affected by the either the reaction or the rate rule, and have the following initial concentration values:

$$[S_2] = 1, \quad [S_3] = 2$$

The values of constant parameters in the system are:

$$k_1 = 0.5, \quad k_2 = 0.1$$

and the initial values of varying species are:

$$[S_1] = 0, \quad [S_4] = 0$$

The value of $[S_1]$ varies over time and it is on the boundary, so in the SBML representation, `S1` has a `constant` attribute with a value of "`false`" and a `boundaryCondition` attribute with a value of "`true`". The value of $[S_2]$ is fixed and it is also on the boundary, so `S2` has a `constant` attribute value of "`false`" and a `boundaryCondition` attribute value of "`true`". $[S_3]$ is fixed but not on the boundary, so the `constant` attribute is "`true`" and the `boundaryCondition` attribute is "`false`". Finally, $[S_4]$ is a product whose value is determined by a kinetic law and therefore in the SBML representation has "`false`" for both its `boundaryCondition` and `constant` attributes.

The following is the SBML rendition of the model shown above:

```
<?xml version="1.0" encoding="UTF-8"?>
<sbml xmlns="http://www.sbml.org/sbml/level3/version2/core" level="3" version="2">
    <model id="BoundaryCondExampleModel"
           volumeUnits="litre" substanceUnits="mole" timeUnits="second" extentUnits="mole">
        <listOfUnitDefinitions>
           <unitDefinition id="mole_per_litre_per_second">
              <listOfUnits>
                  <unit kind="mole" exponent="1" scale="0" multiplier="1"/>
```

```
                <unit kind="litre" exponent="-1" scale="0" multiplier="1"/>
                <unit kind="second" exponent="-1" scale="0" multiplier="1"/>
            </listOfUnits>
         </unitDefinition>
         <unitDefinition id="litre_sq_per_mole_sq_per_second">
            <listOfUnits>
                <unit kind="mole" exponent="-2" scale="0" multiplier="1"/>
                <unit kind="litre" exponent="2" scale="0" multiplier="1"/>
                <unit kind="second" exponent="-1" scale="0" multiplier="1"/>
            </listOfUnits>
         </unitDefinition>
      </listOfUnitDefinitions>
      <listOfCompartments>
         <compartment id="compartmentOne" size="1" spatialDimensions="3" constant="true"/>
      </listOfCompartments>
      <listOfSpecies>
        <species id="S1" initialConcentration="0" compartment="compartmentOne" constant="false"
                         hasOnlySubstanceUnits="false" boundaryCondition="true"/>
        <species id="S2" initialConcentration="1" compartment="compartmentOne" constant="false"
                         hasOnlySubstanceUnits="false" boundaryCondition="true"/>
        <species id="S3" initialConcentration="3" compartment="compartmentOne" constant="false"
                         hasOnlySubstanceUnits="false" boundaryCondition="false"/>
        <species id="S4" initialConcentration="0" compartment="compartmentOne" constant="false"
                         hasOnlySubstanceUnits="false" boundaryCondition="false"/>
      </listOfSpecies>
      <listOfParameters>
         <parameter id="k1" value="0.5" units="litre_sq_per_mole_sq_per_second"
                    constant="true"/>
         <parameter id="k2" value="0.1" units="mole_per_litre_per_second"
                    constant="true"/>
      </listOfParameters>
      <listOfRules>
         <rateRule variable="S1">
             <math xmlns="http://www.w3.org/1998/Math/MathML">
                 <ci> k2 </ci>
             </math>
         </rateRule>
      </listOfRules>
      <listOfReactions>
         <reaction id="reaction_1" reversible="false">
            <listOfReactants>
                <speciesReference species="S1" stoichiometry="1" constant="true"/>
                <speciesReference species="S2" stoichiometry="1" constant="true"/>
            </listOfReactants>
            <listOfProducts>
                <speciesReference species="S4" stoichiometry="1" constant="true"/>
            </listOfProducts>
            <listOfModifiers>
                <modifierSpeciesReference species="S3"/>
            </listOfModifiers>
            <kineticLaw>
                <math xmlns="http://www.w3.org/1998/Math/MathML">
                    <apply>
                        <times/>
                        <ci> k1 </ci>
                        <ci> S1 </ci>
                        <ci> S2 </ci>
                        <ci> S3 </ci>
                        <ci> compartmentOne </ci>
                    </apply>
                </math>
            </kineticLaw>
        </reaction>
      </listOfReactions>
  </model>
</sbml>
```

### 7.8 Example of translation from a multi-compartmental model to ODEs

This section contains a model with two compartments and four reactions. The model is derived from Lotka-Volterra, with the addition of a reversible transport step. When observed in a time-course simulation, three of this model's species display damped oscillations.

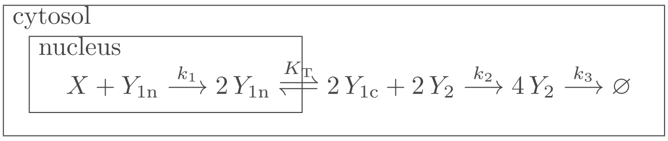

***Figure 29:*** *An example multi-compartmental model.*

Figure 29 illustrates the arrangement of compartments and reactions in the model `LotkaVolterra_transport`. The reaction between the compartments called `cytosol` and `nucleus` is a transport reaction whose mechanisms are not modeled here; in particular, the reaction does not take place on the membrane between the compartments, and is modeled here simply as a process that spans the two three-dimensional compartments.

The text of the SBML representation of the model is shown below, and it is followed by its complete translation into ordinary differential equations. As usual, in this SBML model, the reaction rate equations in the kinetic laws are in substance per time units. The reactions have also been simplified to reduce common stoichiometric factors in the original system depicted in Figure 29. The species variables in this SBML representation are in concentration units; their initial quantities are declared using the attribute `initialAmount` on the `species` definitions, but since the attribute `hasOnlySubstanceUnits` is *not* set to true, the identifiers of the species represent their concentrations when those identifiers appear in mathematical expressions elsewhere in the model. Note that the species whose identifier is "`X`" is a boundary condition, as indicated by the attribute `boundaryCondition`="`true`" in its definition.

```
<?xml version="1.0" encoding="UTF-8"?>
<sbml xmlns="http://www.sbml.org/sbml/level3/version2/core" level="3" version="2">
    <model name="LotkaVolterra_tranport" substanceUnits="mole" volumeUnits="litre"
           extentUnits="mole" timeUnits="second">
        <listOfUnitDefinitions>
            <unitDefinition id="per_second">
                <listOfUnits>
                    <unit kind="second" exponent="-1" scale="0" multiplier="1"/>
                </listOfUnits>
            </unitDefinition>
            <unitDefinition id="litre_per_mole_per_second">
                <listOfUnits>
                    <unit kind="mole"   exponent="-1" scale="0" multiplier="1"/>
                    <unit kind="litre"  exponent="1"  scale="0" multiplier="1"/>
                    <unit kind="second" exponent="-1" scale="0" multiplier="1"/>
                </listOfUnits>
            </unitDefinition>
        </listOfUnitDefinitions>
        <listOfCompartments>
            <compartment id="cytoplasm" size="5" constant="true" spatialDimensions="3"/>
            <compartment id="nucleus"   size="1" constant="true" spatialDimensions="3"/>
        </listOfCompartments>
        <listOfSpecies>
            <species id="X"   compartment="nucleus" initialAmount="1" constant="false"
                              boundaryCondition="true"  hasOnlySubstanceUnits="false"/>
            <species id="Y1n" compartment="nucleus" initialAmount="1" constant="false"
                              boundaryCondition="false" hasOnlySubstanceUnits="false"/>
            <species id="Y1c" compartment="cytoplasm"   initialAmount="0" constant="false"
                              boundaryCondition="false" hasOnlySubstanceUnits="false"/>
            <species id="Y2"  compartment="cytoplasm"   initialAmount="1" constant="false"
                              boundaryCondition="false" hasOnlySubstanceUnits="false"/>
        </listOfSpecies>
        <listOfParameters>
            <parameter id="k1" value="2500"  units="litre_per_mole_per_second" constant="true"/>
```

```
            <parameter id="k2" value="2500"  units="litre_per_mole_per_second" constant="true"/>
            <parameter id="KT" value="25000" units="per_second"                constant="true"/>
            <parameter id="k3" value="2500"  units="per_second"                constant="true"/>
        </listOfParameters>
        <listOfReactions>
            <reaction id="production" reversible="false">
                <listOfReactants>
                    <speciesReference species="X"   stoichiometry="1" constant="true"/>
                    <speciesReference species="Y1n" stoichiometry="1" constant="true"/>
                </listOfReactants>
                <listOfProducts>
                    <speciesReference species="Y1n" stoichiometry="1" constant="true"/>
                    <speciesReference species="Y1n" stoichiometry="1" constant="true"/>
                </listOfProducts>
                <kineticLaw>
                    <math xmlns="http://www.w3.org/1998/Math/MathML">
                        <apply>
                            <times/>
                            <ci>nucleus</ci>
                            <ci>k1</ci>
                            <ci>X</ci>
                            <ci>Y1n</ci>
                        </apply>
                    </math>
                </kineticLaw>
            </reaction>
            <reaction id="transport" reversible="true">
                <listOfReactants>
                    <speciesReference species="Y1n" stoichiometry="1" constant="true"/>
                </listOfReactants>
                <listOfProducts>
                    <speciesReference species="Y1c" stoichiometry="1" constant="true"/>
                </listOfProducts>
                <kineticLaw>
                    <math xmlns="http://www.w3.org/1998/Math/MathML">
                        <apply>
                            <times/>
                            <ci>cytoplasm</ci>
                            <ci>KT</ci>
                            <apply>
                                <minus/>
                                <ci>Y1n</ci>
                                <ci>Y1c</ci>
                            </apply>
                        </apply>
                    </math>
                </kineticLaw>
            </reaction>
            <reaction id="transformation" reversible="false">
                <listOfReactants>
                    <speciesReference species="Y1c" stoichiometry="1" constant="true"/>
                    <speciesReference species="Y2"  stoichiometry="1" constant="true"/>
                </listOfReactants>
                <listOfProducts>
                    <speciesReference species="Y2"  stoichiometry="2" constant="true"/>
                </listOfProducts>
                <kineticLaw>
                    <math xmlns="http://www.w3.org/1998/Math/MathML">
                        <apply>
                            <times/>
                            <ci>cytoplasm</ci>
                            <ci>k2</ci>
                            <ci>Y1c</ci>
                            <ci>Y2</ci>
                        </apply>
                    </math>
                </kineticLaw>
            </reaction>
            <reaction id="degradation" reversible="false">
```

```
                    <listOfReactants>
                        <speciesReference species="Y2" stoichiometry="1" constant="true"/>
                    </listOfReactants>
                    <kineticLaw>
                        <math xmlns="http://www.w3.org/1998/Math/MathML">
                            <apply>
                                <times/>
                                <ci>cytoplasm</ci>
                                <ci>k3</ci>
                                <ci>Y2</ci>
                            </apply>
                        </math>
                    </kineticLaw>
                </reaction>
            </listOfReactions>
        </model>
    </sbml>
```

The ODE translation of this model is as follows. First, we give the values of the constant parameters:

$$\begin{aligned} k_1 &= 2500 \text{ litre mole}^{-1} \text{ second}^{-1} \\ k_2 &= 2500 \text{ litre mole}^{-1} \text{ second}^{-1} \\ K_3 &= 25000 \text{ second}^{-1} \\ K_T &= 25000 \text{ second}^{-1} \end{aligned}$$

Now on to the initial conditions of the variables. In the following, the terms $[X]$, $[Y_{1n}]$, $[Y_{1c}]$, and $[Y_2]$ refer to the species' concentrations. Note that the corresponding species identifiers `X`, `Y_1n`, `Y_1c` and `Y_2` in the model are in concentration units, even though all the values in the model are initialized in terms of amounts. (The reason the species identifiers in the model are still in concentration units goes back to the meaning of the `hasOnlySubstanceUnits` attribute on a **Species**; if the attribute is set to a value of "`false`", a species' symbol in a model is interpreted as a concentration or density regardless of whether its initial value is set using `initialAmount` or `initialConcentration`.) We use $V_n$ to represent the size of compartment "`nucleus`" and $V_c$ the size of compartment "`cytoplasm`":

$$\begin{aligned} V_n &= 1 \text{ litre} \\ V_c &= 5 \text{ litre} \\ X &= 1 \text{ mole} \\ Y_{1n} &= 1 \text{ mole} \\ Y_{1c} &= 0 \text{ mole} \\ Y_2 &= 1/5 \text{ mole} \end{aligned}$$

And finally, here are the differential equations:

$$\frac{d[X]}{dt} = 0$$

$$V_n \frac{d[Y_{1n}]}{dt} = k_1[X][Y_{1n}]V_n - K_T\big([Y_{1n}] - [Y_{1c}]\big)V_c \qquad \text{reactions production and transport}$$

$$V_c \frac{d[Y_{1c}]}{dt} = K_T\big([Y_{1n}] - [Y_{1c}]\big)V_c - k_2[Y_{1c}][Y_2]V_c \qquad \text{reactions transport and transformation}$$

$$V_c \frac{d[Y_2]}{dt} = k_2[Y_{1c}][Y_2]V_c - k_3[Y_2]V_c \qquad \text{reactions transformation and degradation}$$

As formulated here, this example assumes constant volumes. If the sizes of the compartments "`cytoplasm`" or "`nucleus`" could change during simulation, then it would be preferable to use a different approach to constructing the differential equations. In this alternative approach, the ODEs would compute substance change rather than concentration change, and the concentration values would be computed using separate equations. This approach is used in Section 4.11.7 on p. 77.

### 7.9 Example involving function definitions

This section contains a model that uses the function definition feature of SBML. Consider the following hypothetical system:

$$S_1 \xrightarrow{f([S_1])} S_2$$

where

$$f(x) = 2x$$

The following is the XML document that encodes the model shown above:

```
<?xml version="1.0" encoding="UTF-8"?>
<sbml xmlns="http://www.sbml.org/sbml/level3/version2/core" level="3" version="2">
    <model id="Example" substanceUnits="mole" volumeUnits="litre"
           timeUnits="second" extentUnits="mole">
        <listOfUnitDefinitions>
            <unitDefinition id="conc">
                <listOfUnits>
                    <unit kind="mole" multiplier="1" scale="0" exponent="1"/>
                    <unit kind="litre" multiplier="1" scale="0" exponent="-1"/>
                </listOfUnits>
            </unitDefinition>
        </listOfUnitDefinitions>
        <listOfFunctionDefinitions>
            <functionDefinition id="f">
                <math xmlns="http://www.w3.org/1998/Math/MathML"
                      xmlns:sbml="http://www.sbml.org/sbml/level3/version2/core">
                    <lambda>
                        <bvar>
                            <ci> x </ci>
                        </bvar>
                        <apply>
                            <times/>
                            <ci> x </ci>
                            <cn sbml:units="dimensionless"> 2 </cn>
                        </apply>
                    </lambda>
                </math>
            </functionDefinition>
        </listOfFunctionDefinitions>
        <listOfCompartments>
            <compartment id="compartmentOne" size="1" spatialDimensions="3" constant="true"/>
        </listOfCompartments>
        <listOfSpecies>
            <species id="S1" initialConcentration="1" compartment="compartmentOne"
                     hasOnlySubstanceUnits="false" boundaryCondition="false"
                     constant="false"/>
            <species id="S2" initialConcentration="0" compartment="compartmentOne"
                     hasOnlySubstanceUnits="false" boundaryCondition="false"
                     constant="false"/>
        </listOfSpecies>
        <listOfParameters>
            <parameter id="t" value = "1" constant="true"/>
        </listOfParameters>
        <listOfReactions>
            <reaction id="reaction_1" reversible="false">
                <listOfReactants>
                    <speciesReference species="S1" stoichiometry="1" constant="true"/>
                </listOfReactants>
                <listOfProducts>
                    <speciesReference species="S2" stoichiometry="1" constant="true"/>
                </listOfProducts>
                <kineticLaw>
                    <math xmlns="http://www.w3.org/1998/Math/MathML">
                        <apply>
                        <divide/>
                            <apply>
```

```
                                <times/>
                                <apply>
                                    <ci> f </ci>
                                    <ci> S1 </ci>
                                </apply>
                                <ci> compartmentOne </ci>
                            </apply>
                            <ci> t</ci>
                        </apply>
                    </math>
                </kineticLaw>
            </reaction>
        </listOfReactions>
    </model>
</sbml>
```

### 7.10 Example involving *delay* functions

The following is a simple model illustrating the use of $delay$ to represent a gene that suppresses its own expression. The model can be expressed in a single rule:

$$\frac{d[P]}{dt} = \frac{\dfrac{1}{1+m[P_{\text{delayed}}]^q} - [P]}{\tau}$$

where

$$\begin{aligned} [P_{\text{delayed}}] &\ \text{is delay}([P], \Delta_t) \text{ or } [P] \text{ at } t-\Delta_t \\ [P] &\ \text{is protein concentration} \\ \tau &\ \text{is the response time} \\ m &\ \text{is a multiplier or equilibrium constant} \\ q &\ \text{is the Hill coefficient} \end{aligned}$$

and the species quantities are in concentration units. The text of an SBML encoding of this model is given below:

```
<?xml version="1.0" encoding="UTF-8"?>
<sbml xmlns="http://www.sbml.org/sbml/level3/version2/core" level="3" version="2">
    <model substanceUnits="mole" volumeUnits="litre"
           extentUnits="mole" timeUnits="second">
        <listOfUnitDefinitions>
          <unitDefinition id="conc">
            <listOfUnits>
              <unit kind="mole" multiplier="1" scale="0" exponent="1"/>
              <unit kind="litre" multiplier="1" scale="0" exponent="-1"/>
            </listOfUnits>
          </unitDefinition>
          <unitDefinition id="conc_sq">
            <listOfUnits>
              <unit kind="mole" multiplier="1" scale="0" exponent="2"/>
              <unit kind="litre" multiplier="1" scale="0" exponent="-2"/>
            </listOfUnits>
          </unitDefinition>
        </listOfUnitDefinitions>
        <listOfCompartments>
            <compartment id="cell" size="1" spatialDimensions="3" constant="true"/>
        </listOfCompartments>
        <listOfSpecies>
            <species id="P" compartment="cell" initialConcentration="0"
                     hasOnlySubstanceUnits="false" boundaryCondition="false"
                     constant="false"/>
        </listOfSpecies>
        <listOfParameters>
            <parameter id="tau"     value="1"   units="second"        constant="true"/>
            <parameter id="m"       value="0.5" units="dimensionless" constant="true"/>
            <parameter id="q"       value="1"   units="dimensionless" constant="true"/>
            <parameter id="delta_t" value="1"   units="second"        constant="true"/>
```

```
        </listOfParameters>
        <listOfRules>
            <rateRule variable="P">
                <math xmlns="http://www.w3.org/1998/Math/MathML"
                      xmlns:sbml="http://www.sbml.org/sbml/level3/version2/core">
                  <apply>
                    <divide/>
                    <apply>
                      <minus/>
                      <apply>
                        <divide/>
                        <cn sbml:units="conc_sq"> 1 </cn>
                        <apply>
                          <plus/>
                          <cn sbml:units="conc"> 1 </cn>
                          <apply>
                            <times/>
                            <ci> m </ci>
                            <apply>
                              <power/>
                              <apply>
                                <csymbol
                                     encoding="text"
                                     definitionURL="http://www.sbml.org/sbml/symbols/delay">
                                    delay
                                </csymbol>
                                <ci> P </ci>
                                <ci> delta_t </ci>
                              </apply>
                              <ci> q </ci>
                            </apply>
                          </apply>
                        </apply>
                      </apply>
                      <ci> P </ci>
                    </apply>
                    <ci> tau </ci>
                  </apply>
                </math>
            </rateRule>
        </listOfRules>
    </model>
</sbml>
```

### 7.11 Example involving events

This section presents a simple model system that demonstrates the use of events in SBML. Consider a system with two genes, $G_1$ and $G_2$. $G_1$ is initially on and $G_2$ is initially off. When turned on, the two genes lead to the production of two products, $P_1$ and $P_2$, respectively, at a fixed rate. When $P_1$ reaches a given concentration, $G_2$ switches on. This system can be represented mathematically as follows:

$$\begin{aligned}
\frac{d[P_1]}{dt} &= k_1\big([G_1] - [P_1]\big) \\
\frac{d[P_2]}{dt} &= k_2\big([G_2] - [P_2]\big) \\
[G_2] &= \begin{cases} 0 & \text{when } [P_1] \leq \tau, \\ 1 & \text{when } [P_1] > \tau. \end{cases}
\end{aligned}$$

The initial values are:

$$[G_1] = 1, \quad [G_2] = 0, \quad \tau = 0.25, \quad P_1 = 0, \quad P_2 = 0, \quad k_1 = k_2 = 1.$$

The SBML Level 3 representation of this is as follows:

```
<?xml version="1.0" encoding="UTF-8"?>
<sbml xmlns="http://www.sbml.org/sbml/level3/version2/core" level="3" version="2">
      xmlns:math="http://www.w3.org/1998/Math/MathML"
      xmlns:sbml="http://www.sbml.org/sbml/level3/version2/core">
    <model substanceUnits="mole" volumeUnits="litre" timeUnits="second"
           extentUnits="mole">
        <listOfUnitDefinitions>
            <unitDefinition id="per_second">
                <listOfUnits>
                    <unit kind="second" exponent="-1" scale="0" multiplier="1"/>
                </listOfUnits>
            </unitDefinition>
            <unitDefinition id="concentration">
                <listOfUnits>
                    <unit kind="mole"  exponent="1"  scale="0" multiplier="1"/>
                    <unit kind="litre" exponent="-1" scale="0" multiplier="1"/>
                </listOfUnits>
            </unitDefinition>
        </listOfUnitDefinitions>
        <listOfCompartments>
            <compartment id="cell" size="1" spatialDimensions="3" constant="true"/>
        </listOfCompartments>
        <listOfSpecies>
            <species id="P1" compartment="cell" initialConcentration="0"
                     hasOnlySubstanceUnits="false" boundaryCondition="false"
                     constant="false"/>
            <species id="P2" compartment="cell" initialConcentration="0"
                     hasOnlySubstanceUnits="false" boundaryCondition="false"
                     constant="false"/>
        </listOfSpecies>
        <listOfParameters>
            <parameter id="k1"   value="1"    units="per_second"    constant="true"/>
            <parameter id="k2"   value="1"    units="per_second"    constant="true"/>
            <parameter id="tau"  value="0.25" units="concentration" constant="true"/>
            <parameter id="G1"   value="1"    units="concentration" constant="false"/>
            <parameter id="G2"   value="0"    units="concentration" constant="false"/>
        </listOfParameters>
        <listOfRules>
            <rateRule variable="P1">
                <math:math>
                    <math:apply>
                        <math:times/>
                        <math:ci> k1 </math:ci>
                        <math:apply>
                            <math:minus/>
                            <math:ci> G1 </math:ci>
                            <math:ci> P1 </math:ci>
                        </math:apply>
                    </math:apply>
                </math:math>
            </rateRule>
            <rateRule variable="P2">
                <math:math>
                    <math:apply>
                        <math:times/>
                        <math:ci> k2 </math:ci>
                        <math:apply>
                            <math:minus/>
                            <math:ci> G2 </math:ci>
                            <math:ci> P2 </math:ci>
                        </math:apply>
                    </math:apply>
                </math:math>
            </rateRule>
        </listOfRules>
        <listOfEvents>
            <event useValuesFromTriggerTime="true">
                <trigger persistent="false" initialValue="true">
```

```
                    <math:math>
                        <math:apply>
                            <math:gt/>
                            <math:ci> P1 </math:ci>
                            <math:ci> tau </math:ci>
                        </math:apply>
                    </math:math>
                </trigger>
                <listOfEventAssignments>
                    <eventAssignment variable="G2">
                        <math:math>
                            <math:cn sbml:units="concentration"> 1 </math:cn>
                        </math:math>
                    </eventAssignment>
                </listOfEventAssignments>
            </event>
            <event useValuesFromTriggerTime="true">
                <trigger persistent="false" initialValue="true">
                    <math:math>
                        <math:apply>
                            <math:leq/>
                            <math:ci> P1 </math:ci>
                            <math:ci> tau </math:ci>
                        </math:apply>
                    </math:math>
                </trigger>
                <listOfEventAssignments>
                    <eventAssignment variable="G2">
                        <math:math>
                            <math:cn sbml:units="concentration"> 0 </math:cn>
                        </math:math>
                    </eventAssignment>
                </listOfEventAssignments>
            </event>
        </listOfEvents>
    </model>
</sbml>
```

### 7.12 Example involving two-dimensional compartments

The following example is a model that uses a two-dimensional compartment. It is a fragment of a larger model of calcium regulation across the plasma membrane of a cell. The model includes a calcium influx channel, "Ca_channel", and a calcium-extruding PMCA pump, "Ca_Pump". It also includes two cytosolic proteins that buffer calcium via the "CalciumCalbindin_gt_BoundCytosol" and "CalciumBuffer_gt_BoundCytosol" reactions. Finally, the rate expressions in this model do not include explicit factors of the compartment volumes; instead, the various rate constants are assumed to include any necessary corrections for volume.

```
<?xml version="1.0" encoding="UTF-8"?>
<sbml xmlns="http://www.sbml.org/sbml/level3/version2/core" level="3" version="2">
   <model id="facilitated_ca_diffusion" substanceUnits="substance"
          areaUnits="area" volumeUnits="litre" timeUnits="second" extentUnits="substance">
       <listOfUnitDefinitions>
           <unitDefinition id="substance">
               <listOfUnits>
                   <unit kind="mole" exponent="1" scale="-6" multiplier="1"/>
               </listOfUnits>
           </unitDefinition>
           <unitDefinition id="area">
               <listOfUnits>
                   <unit kind="metre" exponent="2" scale="-6" multiplier="1"/>
               </listOfUnits>
           </unitDefinition>
           <unitDefinition id="per_second">
               <listOfUnits>
                   <unit kind="second" exponent="-1" scale="0" multiplier="1"/>
               </listOfUnits>
           </unitDefinition>
           <unitDefinition id="litre_per_mole_per_second">
```

```
                <listOfUnits>
                    <unit kind="mole"   exponent="-1" scale="-6" multiplier="1"/>
                    <unit kind="litre"  exponent="1"  scale="0"  multiplier="1"/>
                    <unit kind="second" exponent="-1" scale="0"  multiplier="1"/>
                </listOfUnits>
            </unitDefinition>
            <unitDefinition id="subs_per_vol">
                <listOfUnits>
                    <unit kind="mole"  exponent="1"  scale="-6" multiplier="1"/>
                    <unit kind="litre" exponent="-1" scale="0"  multiplier="1"/>
                </listOfUnits>
            </unitDefinition>
        </listOfUnitDefinitions>
        <listOfCompartments>
            <compartment id="Extracellular"
                         spatialDimensions="3" size="1" constant="true"/>
            <compartment id="PlasmaMembrane"
                         spatialDimensions="2" size="1"
                         constant="true"/>
            <compartment id="Cytosol"
                         spatialDimensions="3" size="1"
                         constant="true"/>
        </listOfCompartments>
        <listOfSpecies>
            <species id="CaBPB_C" compartment="Cytosol" initialConcentration="47.17"
                     hasOnlySubstanceUnits="false" boundaryCondition="false"
                     constant="false"/>
            <species id="B_C" compartment="Cytosol" initialConcentration="396.04"
                     hasOnlySubstanceUnits="false" boundaryCondition="false"
                     constant="false"/>
            <species id="CaB_C" compartment="Cytosol" initialConcentration="3.96"
                     hasOnlySubstanceUnits="false" boundaryCondition="false"
                     constant="false"/>
            <species id="Ca_C" name="Ca" compartment="Cytosol" initialConcentration="0.1"
                     hasOnlySubstanceUnits="false" boundaryCondition="false"
                     constant="false"/>
            <species id="Ca_EC" name="Ca" compartment="Extracellular"
                     initialConcentration="1000"
                     hasOnlySubstanceUnits="false" boundaryCondition="false"
                     constant="false"/>
            <species id="CaCh_PM" compartment="PlasmaMembrane" initialConcentration="1"
                     hasOnlySubstanceUnits="false" boundaryCondition="false"
                     constant="false"/>
            <species id="CaPump_PM" compartment="PlasmaMembrane" initialConcentration="1"
                     hasOnlySubstanceUnits="false" boundaryCondition="false"
                     constant="false"/>
            <species id="CaBP_C" compartment="Cytosol" initialConcentration="202.83"
                     hasOnlySubstanceUnits="false" boundaryCondition="false"
                     constant="false"/>
        </listOfSpecies>
        <listOfReactions>
            <reaction id="CalciumCalbindin_gt_BoundCytosol" reversible="true">
                <listOfReactants>
                    <speciesReference species="CaBP_C" stoichiometry="1" constant="true"/>
                    <speciesReference species="Ca_C"   stoichiometry="1" constant="true"/>
                </listOfReactants>
                <listOfProducts>
                    <speciesReference species="CaBPB_C" stoichiometry="1" constant="true"/>
                </listOfProducts>
                <kineticLaw>
                    <notes>
                        <p xmlns="http://www.w3.org/1999/xhtml">
                           (((Kf_CalciumCalbindin_BoundCytosol * CaBP_C) * Ca_C) -
                              (Kr_CalciumCalbindin_BoundCytosol * CaBPB_C))
                        </p>
                    </notes>
                    <math xmlns="http://www.w3.org/1998/Math/MathML">
                        <apply>
                            <times/>
```

```
                <ci> Cytosol </ci>
                <apply>
                    <minus/>
                    <apply>
                        <times/>
                        <ci> Kf_CalciumCalbindin_BoundCytosol </ci>
                        <ci> CaBP_C </ci>
                        <ci> Ca_C </ci>
                    </apply>
                    <apply>
                        <times/>
                        <ci> Kr_CalciumCalbindin_BoundCytosol </ci>
                        <ci> CaBPB_C </ci>
                    </apply>
                </apply>
            </apply>
        </math>
        <listOfLocalParameters>
            <localParameter id="Kf_CalciumCalbindin_BoundCytosol" value="20.0"
                       units="litre_per_mole_per_second"/>
            <localParameter id="Kr_CalciumCalbindin_BoundCytosol" value="8.6"
                       units="per_second"/>
        </listOfLocalParameters>
    </kineticLaw>
</reaction>
<reaction id="CalciumBuffer_gt_BoundCytosol" reversible="true">
    <listOfReactants>
        <speciesReference species="Ca_C" stoichiometry="1" constant="true"/>
        <speciesReference species="B_C"  stoichiometry="1" constant="true"/>
    </listOfReactants>
    <listOfProducts>
        <speciesReference species="CaB_C" stoichiometry="1" constant="true"/>
    </listOfProducts>
    <kineticLaw>
        <notes>
            <p xmlns="http://www.w3.org/1999/xhtml">
            (((Kf_CalciumBuffer_BoundCytosol * Ca_C) * B_C) -
                (Kr_CalciumBuffer_BoundCytosol * CaB_C))
            </p>
        </notes>
        <math xmlns="http://www.w3.org/1998/Math/MathML">
        <apply>
            <times/>
            <ci> Cytosol</ci>
            <apply>
                <minus/>
                <apply>
                    <times/>
                    <ci> Kf_CalciumBuffer_BoundCytosol </ci>
                    <ci> Ca_C </ci>
                    <ci> B_C </ci>
                </apply>
                <apply>
                    <times/>
                    <ci> Kr_CalciumBuffer_BoundCytosol </ci>
                    <ci> CaB_C </ci>
                </apply>
            </apply>
        </apply>
        </math>
        <listOfLocalParameters>
            <localParameter id="Kf_CalciumBuffer_BoundCytosol" value="0.1"
                       units="litre_per_mole_per_second"/>
            <localParameter id="Kr_CalciumBuffer_BoundCytosol" value="1.0"
                       units="per_second"/>
        </listOfLocalParameters>
    </kineticLaw>
</reaction>
<reaction id="Ca_Pump" reversible="true">
```

```
                <listOfReactants>
                    <speciesReference species="Ca_C" stoichiometry="1" constant="true"/>
                </listOfReactants>
                <listOfProducts>
                    <speciesReference species="Ca_EC" stoichiometry="1" constant="true"/>
                </listOfProducts>
                <listOfModifiers>
                    <modifierSpeciesReference species="CaPump_PM"/>
                </listOfModifiers>
                <kineticLaw>
                    <notes>
                        <p xmlns="http://www.w3.org/1999/xhtml">
                        ((Vmax * kP * ((Ca_C - Ca_Rest) / (Ca_C + kP)) /
                            (Ca_Rest + kP)) * CaPump_PM)
                        </p>
                    </notes>
                    <math xmlns="http://www.w3.org/1998/Math/MathML">
                    <apply>
                        <times/>
                        <ci> PlasmaMembrane</ci>
                        <apply>
                            <divide/>
                            <apply>
                                <times/>
                                <ci> Vmax </ci>
                                <ci> kP </ci>
                                <ci> CaPump_PM </ci>
                                <apply>
                                    <minus/>
                                    <ci> Ca_C </ci>
                                    <ci> Ca_Rest </ci>
                                </apply>
                            </apply>
                            <apply>
                                <times/>
                                <apply>
                                    <plus/>
                                    <ci> Ca_C </ci>
                                    <ci> kP </ci>
                                </apply>
                                <apply>
                                    <plus/>
                                    <ci> Ca_Rest </ci>
                                    <ci> kP </ci>
                                </apply>
                            </apply>
                        </apply>
                    </apply>
                    </math>
                    <listOfLocalParameters>
                        <localParameter id="Vmax" value="4000" units="per_second"/>
                        <localParameter id="kP" value="0.25" units="subs_per_vol"/>
                        <localParameter id="Ca_Rest" value="0.1" units="subs_per_vol"/>
                    </listOfLocalParameters>
                </kineticLaw>
            </reaction>
            <reaction id="Ca_channel" reversible="true">
                <listOfReactants>
                    <speciesReference species="Ca_EC" stoichiometry="1" constant="true"/>
                </listOfReactants>
                <listOfProducts>
                    <speciesReference species="Ca_C" stoichiometry="1" constant="true"/>
                </listOfProducts>
                <listOfModifiers>
                    <modifierSpeciesReference species="CaCh_PM"/>
                </listOfModifiers>
                <kineticLaw>
                    <notes>
                        <p xmlns="http://www.w3.org/1999/xhtml">
```

```
                                (J0 * Kc * (Ca_EC - Ca_C) / (Kc + Ca_C) * CaCh_PM)
                            </p>
                        </notes>
                        <math xmlns="http://www.w3.org/1998/Math/MathML">
                        <apply>
                            <times/>
                            <ci> PlasmaMembrane </ci>
                            <apply>
                                <divide/>
                                <apply>
                                    <times/>
                                    <ci> CaCh_PM </ci>
                                    <ci> J0 </ci>
                                    <ci> Kc </ci>
                                    <apply>
                                        <minus/>
                                        <ci> Ca_EC </ci>
                                        <ci> Ca_C </ci>
                                    </apply>
                                </apply>
                                <apply>
                                    <plus/>
                                    <ci> Kc </ci>
                                    <ci> Ca_C </ci>
                                </apply>
                            </apply>
                        </apply>
                        </math>
                        <listOfLocalParameters>
                            <localParameter id="J0" value="0.014" units="litre_per_mole_per_second"/>
                            <localParameter id="Kc" value="0.5" units="subs_per_vol"/>
                        </listOfLocalParameters>
                    </kineticLaw>
                </reaction>
            </listOfReactions>
        </model>
    </sbml>
```

### 7.13 Example of a reaction located at a membrane

This section describes a model containing one single enzymatic reaction where substrate and product are located in the same compartment but the enzyme is localized at the membrane surrounding the compartment.

$$R\colon S \xrightarrow{[E]} P$$

The model contains two compartments, a three-dimensional one called "cytosol" and a two-dimensional one called "membrane" that is assumed to be the boundary of the cell. The reaction $R$ has a substrate $S$ and a product $P$ that are both located in the cytosol. The enzyme $E$ that catalyzes the reactions is located at the membrane. The kinetic law of reaction $R$ is

$$v = A \cdot \frac{k_{\mathrm{cat}} \cdot [E] \cdot [S]}{K_{\mathrm{M}} + [S]}$$

where $A$ is the area of the membrane (measured in $\mu\mathrm{m}^2$), $[E]$ is the density of the enzyme on the membrane (in $\mu\mathrm{mol}\ \mu\mathrm{m}^{-2}$), $[S]$ is the concentration of the substrate (in $\mu\mathrm{mol}\ l^{-1}$), $K_{\mathrm{M}}$ the Michaelis-Menten constant (also in $\mu\mathrm{mol}\ l^{-1}$), and $k_{\mathrm{cat}}$ the rate constant (in $\mathrm{min}^{-1}$). The units of the result of the kinetic law are in $\mu\mathrm{mol}\ \mathrm{min}^{-1}$. Since the units for the amounts of all species ($S$, $P$, and $E$) and for the reaction extent are the same (μmol) the model does not require unit conversion factors.

The kinetic law as it is given here scales correctly for changes in cytosol volume, membrane area, or enzyme density. This means that if one of these values is changed (even if it varies during a simulation) the rate expression remains valid.

The following is the text of the model's SBML representation.

```xml
<?xml version="1.0" encoding="UTF-8"?>
<sbml xmlns="http://www.sbml.org/sbml/level3/version2/core" level="3" version="2">
  <model id="Model_1" name="Reaction on membrane" substanceUnits="micromole"
         timeUnits="minute" extentUnits="micromole">
    <listOfFunctionDefinitions>
      <functionDefinition id="MM_enzyme" name="MM_enzyme">
        <math xmlns="http://www.w3.org/1998/Math/MathML">
          <lambda>
            <bvar> <ci> size </ci> </bvar>
            <bvar> <ci> k </ci> </bvar>
            <bvar> <ci> enz </ci> </bvar>
            <bvar> <ci> subs </ci> </bvar>
            <bvar> <ci> Km </ci> </bvar>
            <apply>
              <divide/>
              <apply>
                <times/>
                <ci> size </ci>
                <ci> k </ci>
                <ci> enz </ci>
                <ci> subs </ci>
              </apply>
              <apply>
                <plus/>
                <ci> Km </ci>
                <ci> subs </ci>
              </apply>
            </apply>
          </lambda>
        </math>
      </functionDefinition>
    </listOfFunctionDefinitions>
    <listOfUnitDefinitions>
      <unitDefinition id="minute">
        <listOfUnits>
          <unit kind="second" exponent="1" scale="0" multiplier="60"/>
        </listOfUnits>
      </unitDefinition>
      <unitDefinition id="per_minute">
        <listOfUnits>
          <unit kind="second" exponent="-1" scale="0" multiplier="60"/>
        </listOfUnits>
      </unitDefinition>
      <unitDefinition id="micromole">
        <listOfUnits>
          <unit kind="mole" exponent="1" scale="-6" multiplier="1"/>
        </listOfUnits>
      </unitDefinition>
      <unitDefinition id="micromole_per_l">
        <listOfUnits>
          <unit kind="mole" exponent="1" scale="-6" multiplier="1"/>
          <unit kind="litre" exponent="-1" scale="0" multiplier="1"/>
        </listOfUnits>
      </unitDefinition>
      <unitDefinition id="sqrmicrometre">
        <listOfUnits>
          <unit kind="metre" exponent="2" scale="-6" multiplier="1"/>
        </listOfUnits>
      </unitDefinition>
    </listOfUnitDefinitions>
    <listOfCompartments>
      <compartment id="cyt" name="Cytosol"
                   spatialDimensions="3" units="litre"
                   size="1e-15" constant="true"/>
      <compartment id="mem" name="Membrane"
                   spatialDimensions="2" units="sqrmicrometre"
                   size="1" constant="true"/>
    </listOfCompartments>
```

```
        <listOfSpecies>
          <species id="species_1" name="substrate" compartment="cyt"
                   hasOnlySubstanceUnits="false" boundaryCondition="false"
                   initialConcentration="1" constant="false"/>
          <species id="species_2" name="product" compartment="cyt"
                   hasOnlySubstanceUnits="false" boundaryCondition="false"
                   initialConcentration="1" constant="false"/>
          <species id="species_3" name="enzyme" compartment="mem"
                   hasOnlySubstanceUnits="false" boundaryCondition="false"
                   initialConcentration="1" constant="false"/>
        </listOfSpecies>
        <listOfReactions>
          <reaction id="reaction_1" name="Reaction" reversible="false"
                    compartment="mem">
            <listOfReactants>
              <speciesReference species="species_1" stoichiometry="1" constant="true"/>
            </listOfReactants>
            <listOfProducts>
              <speciesReference species="species_2" stoichiometry="1" constant="true"/>
            </listOfProducts>
            <listOfModifiers>
              <modifierSpeciesReference species="species_3"/>
            </listOfModifiers>
            <kineticLaw>
              <math xmlns="http://www.w3.org/1998/Math/MathML">
                <apply>
                  <ci> MM_enzyme </ci>
                  <ci> mem </ci>
                  <ci> k </ci>
                  <ci> species_3 </ci>
                  <ci> species_1 </ci>
                  <ci> Km </ci>
                  </apply>
              </math>
              <listOfLocalParameters>
                <localParameter id="k" value="0.1" units="per_minute"/>
                <localParameter id="Km" value="0.1" units="micromole_per_l"/>
              </listOfLocalParameters>
            </kineticLaw>
          </reaction>
        </listOfReactions>
      </model>
    </sbml>
```

### 7.14 Example using an event with a non-persistent trigger and a delay

This example illustrates the syntax and use of the **Trigger** object on **Event**, particularly the `persistent` attribute, as well as the optional **Delay** object on **Event**. In the model below, the event has a trigger expression that tests the value of species "`a`" in the model, and if the value comes within the range $0.999 \leq a \leq 1.001$, the event triggers and reassigns the value of "`c`" after a delay of 3 seconds.

```
    <?xml version="1.0" encoding="UTF-8"?>
    <sbml xmlns="http://www.sbml.org/sbml/level3/version2/core" level="3" version="2">
      <model id="PersistentEvent" timeUnits="second">
        <listOfUnitDefinitions>
          <unitDefinition id="mol_per_l">
            <listOfUnits>
              <unit kind="mole"  exponent="1"  scale="0" multiplier="1"/>
              <unit kind="litre" exponent="-1" scale="0" multiplier="1"/>
            </listOfUnits>
          </unitDefinition>
          <unitDefinition id="l_per_s">
            <listOfUnits>
              <unit kind="litre"  exponent="1"  scale="0" multiplier="1"/>
              <unit kind="second" exponent="-1" scale="0" multiplier="1"/>
            </listOfUnits>
          </unitDefinition>
        </listOfUnitDefinitions>
```

```
<listOfCompartments>
  <compartment id="compartment" size="1" units="litre"
               spatialDimensions="3" constant="true"/>
</listOfCompartments>
<listOfSpecies>
  <species id="d" initialConcentration="0" boundaryCondition="true"
           compartment="compartment" substanceUnits="mole"
           hasOnlySubstanceUnits="false" constant="false"/>
  <species id="a" initialConcentration="2" boundaryCondition="false"
           compartment="compartment" substanceUnits="mole"
           hasOnlySubstanceUnits="false" constant="false"/>
  <species id="b" initialConcentration="0" boundaryCondition="false"
           compartment="compartment" substanceUnits="mole"
           hasOnlySubstanceUnits="false" constant="false"/>
  <species id="c" initialConcentration="0" boundaryCondition="false"
           compartment="compartment" substanceUnits="mole"
           hasOnlySubstanceUnits="false" constant="false"/>
  <species id="e" initialConcentration="0" boundaryCondition="false"
           compartment="compartment" substanceUnits="mole"
           hasOnlySubstanceUnits="false" constant="false"/>
</listOfSpecies>
<listOfParameters>
  <parameter id="k1" value="0.2" constant="true" units="l_per_s"/>
</listOfParameters>
<listOfRules>
  <assignmentRule variable="e">
    <math xmlns="http://www.w3.org/1998/Math/MathML">
      <cn type="integer" sbml:units="mol_per_l"> 1 </cn>
    </math>
  </assignmentRule>
</listOfRules>
<listOfReactions>
  <reaction id="_J0" reversible="false">
    <listOfReactants>
      <speciesReference species="a" constant="true" stoichiometry="1"/>
    </listOfReactants>
    <listOfProducts>
      <speciesReference species="d" constant="true" stoichiometry="1"/>
    </listOfProducts>
    <kineticLaw>
      <math xmlns="http://www.w3.org/1998/Math/MathML">
        <apply>
          <times/>
          <ci> k1 </ci>
          <ci> a </ci>
        </apply>
      </math>
    </kineticLaw>
  </reaction>
</listOfReactions>
<listOfEvents>
  <event useValuesFromTriggerTime="true">
    <!-- If persistent is 'false', c won't be set to '6', but if it's 'true', it will. -->
    <trigger persistent="false" initialValue="true">
      <math xmlns="http://www.w3.org/1998/Math/MathML">
        <apply>
          <and/>
          <apply>
            <leq/>
            <ci> a </ci>
            <cn> 1.001 </cn>
          </apply>
          <apply>
            <geq/>
            <ci> a </ci>
            <cn> 0.999 </cn>
          </apply>
        </apply>
      </math>
```

```
        </trigger>
        <delay>
          <math xmlns="http://www.w3.org/1998/Math/MathML">
            <cn type="integer" sbml:units="second"> 3 </cn>
          </math>
        </delay>
        <listOfEventAssignments>
          <eventAssignment variable="c">
            <math xmlns="http://www.w3.org/1998/Math/MathML">
              <cn type="integer" sbml:units="mol_per_l"> 6 </cn>
            </math>
          </eventAssignment>
        </listOfEventAssignments>
      </event>
    </listOfEvents>
  </model>
</sbml>
```

## 8 Recommended practices

In this section, we recommend a number of practices for using and interpreting various SBML constructs. These recommendations are non-normative, but we advocate them strongly; ignoring them will not render a model invalid, but may hinder interoperability between different software systems exchanging SBML content.

### 8.1 Recommended practices concerning common SBML attributes and objects

Many SBML components share some or all of the following attributes and objects. We describe recommendations concerning them here, separately from discussing the specific SBML components. In Section 8.2 on p. 148, we turn to the specific SBML components, but the recommendations described here also apply to them.

#### 8.1.1 Identifiers and names

The `id` attribute is available on most (but not all) objects in SBML, and all objects that have `id` attributes also have an optional `name` attribute. How should models treat identifiers and names?

The following is the recommended practice for handling `name`. If a software tool has the capability to display the content of `name` attributes, it should display this content to the user as a component's label instead of the component's `id`. If the user interface does not have this capability (e.g., because it cannot display or use special characters in symbol names), or if the `name` attribute is missing on a given component, then the user interface should display the value of the `id` attribute instead.

As a consequence of the above, authors of software systems that automatically generate values for `id` attributes should be aware some other systems may display the `id`'s to the user. (Script language interpreters are especially likely to display `id` instead of `name`.) Authors therefore may wish to take some care to have their software create `id` values that are: (a) reasonably easy for humans to type and read, and (b) likely to be meaningful (e.g., by making the `id` attribute is an abbreviated form of the `name` attribute value).

#### 8.1.2 Initial Values

SBML allows for the creation of **Compartment**, **Species**, **Parameter**, **LocalParameter** and **SpeciesReference** objects without declaring their initial values directly on the object instances. That is, a **Compartment** object can be created without defining a value for its `size` attribute; a **Species** object can be created without defining a value for either its `initialConcentration` or `initialAmount` attribute; **Parameter** and **LocalParameter** objects can be created without giving a value to their `value` attributes; and a **SpeciesReference** object can be created without assigning a value to its `stoichiometry` attribute. A missing value in the case of **Compartment**, **Species**, **Parameter**, and **SpeciesReference** objects implies that the value is either set via an **InitialAssignment** object elsewhere in the model, or is meant to be obtained from an external source (e.g., by querying the user of a software system), or is unknown. In the case of **LocalParameter** objects, a missing value implies that the value is either unknown or meant to be obtained from an external source.

Where initial values are available and are decimal numbers that *can* be set using the appropriate attribute on an object, the best practice recommendation is to do that in preference to using an **InitialAssignment** construct if there is no particular reason to use **InitialAssignment**. Setting the relevant attribute directly on the **Compartment**, **Species**, and **Parameter** and **SpeciesReference** object is simpler and may be more interoperable with different software systems. This is especially true of `stoichiometry` on **SpeciesReference**, which in the vast majority of models, is never more than a constant floating-point value anyway.

An additional point is worth noting in passing. Although the value attributes of various SBML components are of type `double` (e.g., **Parameter**'s attribute `value`), this does not mean that component values are limited only to decimal numbers. As noted above, other constructs such as **InitialAssignment** can be used to set the value of an object, and since those constructs offer the power of MathML, the results may be rational numbers such as fractions. Software developers should be aware of this possibility when planning the type of storage variables used to hold SBML objects' values.

##### 8.1.3 *The* `constant` *flag*

There is a mandatory `boolean` attribute called `constant` on the **Compartment**, **Species**, **SpeciesReference** and **Parameter** components. A value of "`true`" means that the SBML object in question will not be changed by other constructs in SBML except possibly an **InitialAssignment**. A value of "`false`" indicates an intention to change the element's value by an **AssignmentRule**, **RateRule**, **AlgebraicRule**, **Reaction** or **Event** in the model.

A `constant` attribute value of "`false`" does not *require* that the object in question is changed; strictly speaking, an SBML model is valid even if it sets all `constant` attributes to "`false`" but never actually modify any of the values. However, the best practice recommendation is to communicate intentions by setting `constant` to "`true`" unless an entity in a model really is intended to be changed. The exception to this is **Species**, which are usually part of the reaction system and thus usually need to have `constant`="`false`".

##### 8.1.4 Annotations

*Use of Identifiers.org*

All of the examples of annotations in this specification use URIs from the service hosted at Identifiers.org (Juty et al., 2012). The use of Identifiers.org URIs is not required by SBML, but it is considered a current best practice for SBML annotations. Regardless of whether Identifiers.org URIs are used, a general best practice for URIs used in annotations is that they should be chosen to be as persistent as possible, so future consumers of SBML documents can continue to dereference and understand the annotations.

*Appropriate uses of annotations*

In the description of the **Annotation** object available on every component derived from ***SBase*** (Section 3.2.6 on p. 16), we already made the point that it is critical not to put data essential to understanding a model into annotations. This raises a question: what kind of data may be appropriately put into annotations? Here are some examples:

- Identification information for cross-referencing components in a model with items in a data resource such as a database. This is the purpose of the annotation scheme described in Section 6 on p. 100.
- Application-specific processing instructions that do not change the essential meaning of a model, but help a particular application with tasks such as managing the model, maintaining state data across editing sessions, etc.
- Evidence codes for annotating a model with controlled vocabulary terms that indicate (e.g.) the quality of biological evidence supporting the inclusion of each component in the model. The annotation scheme of Section 6 on p. 100 can be used in this capacity.
- Information about the model that cannot be readily encoded in existing SBML elements, but that does not alter the mathematical meaning of the model.

*Specificity of annotations*

The annotation data (Section 3.2.6 on p. 16) attached to a specific SBML object in a model is assumed by other applications to be directly associated with that particular object. Therefore, it is important to decompose and locate annotation data appropriately in an SBML document. Applications are advised to avoid encoding all their annotations in a single top-level attribute on (e.g.) the **Model** object. The data associated with, for example, an individual **Species** object in a model should be encoded in the `<annotation>` element enclosed within the SBML `<species>` element representing that species in the SBML file.

*Syntax of annotations*

The annotation scheme described in Section 6 on p. 100 is useful for many, but not all, situations. It is tempting to develop new annotation syntaxes for situations that fall outside the scope of the SBML MIRIAM annotation scheme. However, a proliferation of proprietary annotation schemes will hinder software interoperability in the long run.

We recommend the following approach when faced with a need to use alternate annotation syntaxes:

1. The modular nature of SBML Level 3 Version 2 Core means that data that in SBML Level 2 could only be stored in annotations may now be supported using a full SBML Level 3 package. Therefore, software developers and modelers should first check if there already exists a package that may serve their needs. A list of SBML Level 3 packages is always maintained at the SBML website, `http://sbml.org`.

2. If no package exists, developers and modelers may wish to check if someone else has already developed a similar annotation syntax for use with another software system. A list of known SBML annotation schemes is maintained online at `http://sbml.org/Community/Wiki/Known_SBML_annotations`.

3. If none of the above alternatives provide a satisfactory result, developers and modelers should query the SBML discussion list (`sbml-discuss@caltech.edu`) to see if anyone else has been faced with similar problems. Other SBML users may have insights or even partial solutions already available.

### 8.2 Recommended practices concerning specific SBML components

In this section, we describe expectations and recommendations concerning specific SBML components. We do not reiterate the recommendations presented in Section 8.1 on p. 146, but they apply to many of the SBML components discussed here and should be kept in mind. The order of the components discussed here follows the order of their presentation in Section 4 on p. 34, but we only include here those components for which we have specific recommendations.

#### *8.2.1 Unit definitions*

We advise modelers and software tools to declare the units of all quantities in a model, insofar as this is possible, using the various mechanisms provided for this in SBML. Fully declared units can allow software tools to perform dimensional analysis on the units of mathematical expressions, and such analysis can be valuable in helping modelers produce correct models. In addition, it can allow model-wide operations such as conversion or rescaling of units.

##### *Recommendations for choices of units*

Table 9 on the next page lists the units recommended for different SBML components. While a single unit will typically be used in each situation, combinations of the recommended types may also be used, such as `mmol/g` for `substance` elements that are calculated in terms of milimoles per gram dry weight.

##### *Handling units requiring the use of offsets*

As already mentioned, unit definitions and conversions that require offsets cannot be done directly using the simple **UnitDefinition** and **Unit** system in SBML. In fact, SBML does not predefine a unit for Celsius precisely because it would require the use of an offset, and so its inclusion would result in an inconsistent system. Definitions involving Celsius, Fahrenheit or other units with offsets require a different approach.

We discuss approaches to handling units with offsets, starting with the case of degrees Celsius:

- *Handling Celsius.* A model in which certain quantities are temperatures measured in degrees Celsius can be converted straightforwardly to a model in which those temperatures are in kelvin. A software tool could do this by performing a substitution using the following relationship:

$$T_{\text{kelvin}} = T_{\text{Celsius}} + 273.15$$

In every mathematical formula of the model where a quantity (call it $x$) in degrees Celsius appears, replace $x$ with $x_{\text{k}} + 273.15$ where $x_{\text{k}}$ is now in kelvin. An alternative approach would be to use a **FunctionDefinition** to define a function encapsulating this relationship above and then using that in the rest of the model as needed. Since Celsius is a commonly-used unit, software tools could help users by providing users with the ability to express temperatures in Celsius in the tools' interfaces, and making substitutions automatically when writing out SBML.

***Table 9:*** *Units recommended for use on different SBML model components. Note that* `avogadro` *is considered to be derived from* `dimensionless` *as a consequence of its definition; see Section 4.4.2 on p. 43.*

<table>
<tr><th>Component attribute</th><th>Unit recommendations</th></tr>
<tr><td>Model substanceUnits</td><td>mole, item, dimensionless, kilogram, gram, or units derived from these</td></tr>
<tr><td>Model timeUnits</td><td>second, dimensionless, or units derived from these</td></tr>
<tr><td>Model volumeUnits</td><td>litre, metre$^3$, dimensionless, or units derived from these</td></tr>
<tr><td>Model areaUnits</td><td>metre$^2$, dimensionless, or units derived from these</td></tr>
<tr><td>Model lengthUnits</td><td>metre, dimensionless, or units derived from these</td></tr>
<tr><td>Model extentUnits</td><td>mole, item, dimensionless, kilogram, gram, or units derived from these</td></tr>
<tr><td>Compartment units</td><td>
<table>
<tr><th>Value of attribute spatialDimensions</th><th>Recommended units</th></tr>
<tr><td>“3”</td><td>litre, metre$^3$, dimensionless, or units derived from these</td></tr>
<tr><td>“2”</td><td>metre$^2$, dimensionless, or units derived from these</td></tr>
<tr><td>“1”</td><td>metre, dimensionless, or units derived from these</td></tr>
<tr><td>other</td><td>no specific recommendations</td></tr>
</table>
</td></tr>
<tr><td>Species substanceUnits</td><td>mole, item, dimensionless, kilogram, gram, or units derived from these</td></tr>
<tr><td>Parameter units</td><td>no specific recommendations</td></tr>
</table>

- *Handling other units requiring offsets.* The only other units requiring offsets in SBML's domain of common applications are other temperature units such as Fahrenheit. Few modern scientists employ Fahrenheit degrees; therefore, this is an unusual situation. The complication inherent in converting between degrees Fahrenheit and kelvin is that both a multiplier and an offset are required:

$$T_{\text{kelvin}} = \frac{T_{\text{F}} + 459.67}{1.8}$$

One approach to handling this is to use a **FunctionDefinition** to define a function encapsulating the relationship above, then to substitute a call to this function wherever the original temperature in Fahrenheit appears in the model's mathematical formulas. We provide a candidate definition in Figure 30 on the following page.

An alternative approach not requiring the use of function definitions is to use an **AssignmentRule** for each variable in Fahrenheit units. The **AssignmentRule** could compute the conversion from Fahrenheit to (say) kelvin, assign its value to a variable (with units declared to be "`kelvin`"), and then that variable could be used elsewhere in the model. Still another approach is to rewrite the mathematical formulas of a model to directly incorporate the conversion above wherever the quantity appears.

All of these approaches provide general solutions to the problem of supporting any units requiring offsets in the unit system of SBML Level 3. It can be used for other temperature units requiring an offset (e.g., degrees Rankine or degrees Réaumur), although the likelihood of a real-life model requiring such other temperature units seems exceedingly small.

In summary, the fact that SBML units do not support specifying an offset does not impede the creation of models using alternative units. If conversions are needed, then converting between temperature in degrees Celsius and thermodynamic temperature can be handled rather easily by the simple substitution described above. For the rare case of Fahrenheit and other units requiring combinations of multipliers and offsets, users are encouraged to employ the power of **FunctionDefinition**, **AssignmentRule**, or other constructs in SBML.

```
<listOfUnitDefinitions>
    <unitDefinition id="degree_Fahrenheit">
        <notes><p xmlns="http://www.w3.org/1999/xhtml">
            This captures the notion that the size of a degree in Fahrenheit is 5/9 the size
            of a degree on the kelvin scale.
        </notes>
        <listOfUnits>
            <unit kind="kelvin"        multiplier="5" scale="0" exponent="1"/>
            <unit kind="dimensionless" multiplier="9" scale="0" exponent="-1"/>
        </listOfUnits>
    </unitDefinition>
</listOfUnitDefinitions>
...
<listOfFunctionDefinitions>
    <functionDefinition id="Fahrenheit_to_kelvin">
        <notes><p xmlns="http://www.w3.org/1999/xhtml">
            This function takes a number assumed to be in Fahrenheit degrees and returns a number
            in kelvin degrees.  Callers could use the definition of unit "degree_Fahrenheit" to
            attach units to the argument passed to the call to this function.
        </notes>
        <math xmlns="http://www.w3.org/1998/Math/MathML"
              xmlns:sbml="http://www.sbml.org/sbml/level3/version2/core">
            <lambda>
                <bvar><ci> arg_temp_in_Fahrenheit </ci></bvar>
                <apply>
                    <divide/>
                    <apply>
                        <plus/>
                        <ci> arg_temp_in_fahrenheit </ci>
                        <cn sbml:units="degree_Fahrenheit"> 459.67</cn>
                    </apply>
                    <apply>
                        <divide/>
                        <cn sbml:units="degree_Fahrenheit"> 1.8 </cn>
                        <cn sbml:units="kelvin"> 1 </cn>
                    </apply>
                </apply>
            </lambda>
        </math>
    </functionDefinition>
</listOfFunctionDefinitions>
```

***Figure 30:*** *SBML fragment showing a candidate definition of a function to convert Fahrenheit temperature to kelvin, along with necessary unit definitions to make the definition complete.*

#### *8.2.2 Compartments*

*Setting the* `size` *attribute on a* `compartment`

As mentioned in Section 4.5.3 on p. 47, we highly recommend that every **Compartment** object in a model has its `size` set. There are three major technical reasons for this. First, if the model contains any species whose initial amounts are given in terms of concentrations, and there is at least one reaction in the model referencing such a species, then the model will be numerically incomplete if it lacks a value for the size of the compartment in which the species is located. The reason is that SBML reactions are expected to be in terms of intensive properties such as *amount*/*time* (or more generally, *extent units*/*time units*; see Section 4.11.7 on p. 78), and converting from concentration to amount requires knowing the compartment size. Second, models ideally should be capable of being instantiated in a variety of simulation frameworks. A commonly-used one is the discrete stochastic framework (Gillespie, 1977; Wilkinson, 2006) in which species are represented as item counts (e.g., molecule counts). If species' initial quantities are given in terms of concentrations or densities, it is impossible to convert the values to item counts without knowing compartment sizes. Third, if a model contains multiple compartments whose sizes are not all identical to each other, it is impossible to quantify the reaction rate expressions without knowing the compartment volumes. The reason for the latter is again that reaction rates in SBML are defined in terms *extent*/*time*, and when species quantities are given in terms of concentrations or densities, the compartment sizes usually become factors in the reaction rate expressions.

*Indicating a default compartment*

Some types of models do not use compartments, for example because they factor out volumes completely. Since SBML requires at least one compartment to be defined if any species exists in a model, the representation of models where no compartments are needed sometimes leaves model creators wishing they could indicate that a compartment is only a "default" in some sense. The recommended approach to handling this situation is to annotate the **Compartment** object by setting its `sboTerm` attribute to an appropriate SBO term, specifically "`SBO:0000410`".

#### 8.2.3 Rules

Section 4.9.5 on p. 64 establishes the fact that when **AlgebraicRule** objects are used, it is possible to produce a model that is overdetermined. When a model includes both **Event** and **Reaction** objects, it is necessary to analyze the set of equations produced from the rules and reactions and the set of equations produces from rules and the event assignments of each event. Each set of equations must not be overdetermined. In addition, each set of equations must be fully determined if accurate simulation is to be performed.

The following example illustrates a case where the set of equations is fully determined. First, we present the SBML expression of the model:

```
<?xml version="1.0" encoding="UTF-8"?>
<sbml xmlns="http://www.sbml.org/sbml/level3/version2/core" level="3" version="2">
    <model id="example" substanceUnits="mole" volumeUnits="litre"
           timeUnits="second" extentUnits="mole">
        <listOfUnitDefinitions>
            <unitDefinition id="conc">
                <listOfUnits>
                    <unit kind="mole"  multiplier="1" scale="0" exponent="1"/>
                    <unit kind="litre" multiplier="1" scale="0" exponent="-1"/>
                </listOfUnits>
            </unitDefinition>
            <unitDefinition id="per_second">
                <listOfUnits>
                    <unit kind="second" exponent="-1" scale="0" multiplier="1"/>
                </listOfUnits>
            </unitDefinition>
        </listOfUnitDefinitions>
        <listOfCompartments>
            <compartment id="C" size="1" spatialDimensions="3" constant="true"/>
        </listOfCompartments>
        <listOfSpecies>
            <species id="S1" compartment="C" initialConcentration="1" constant="false"
                             boundaryCondition="false" hasOnlySubstanceUnits="false"/>
            <species id="S2" compartment="C" initialConcentration="0" constant="false"
                             boundaryCondition="false" hasOnlySubstanceUnits="false"/>
            <species id="S3" compartment="C" initialConcentration="0" constant="false"
                             boundaryCondition="false" hasOnlySubstanceUnits="false"/>
        </listOfSpecies>
        <listOfParameters>
            <parameter id="p1" value="1"   constant="true" units="conc"/>
            <parameter id="p2" value="1.5" constant="true" units="conc"/>
        </listOfParameters>
        <listOfRules>
            <algebraicRule>
                <math xmlns="http://www.w3.org/1998/Math/MathML">
                    <apply> <minus/> <ci> S1 </ci> <ci> S3 </ci> </apply>
                </math>
            </algebraicRule>
        </listOfRules>
        <listOfReactions>
            <reaction id="R" reversible="true">
                <listOfReactants>
                    <speciesReference species="S1" stoichiometry="1" constant="true"/>
                </listOfReactants>
                <listOfProducts>
```

```
                    <speciesReference species="S2" stoichiometry="1" constant="true"/>
                </listOfProducts>
                <kineticLaw>
                    <math xmlns="http://www.w3.org/1998/Math/MathML">
                        <apply> <times/> <ci> C </ci> <ci> k1 </ci> <ci> S1 </ci>
                        </apply>
                    </math>
                    <listOfLocalParameters>
                        <localParameter id="k1" value="0.1" units="per_second"/>
                    </listOfLocalParameters>
                </kineticLaw>
            </reaction>
        </listOfReactions>
        <listOfEvents>
            <event useValuesFromTriggerTime="true">
                <trigger persistent="false" initialValue="true">
                    <math xmlns="http://www.w3.org/1998/Math/MathML">
                        <apply> <gt/> <ci> S2 </ci> <ci> p1 </ci> </apply>
                    </math>
                </trigger>
                <listOfEventAssignments>
                  <eventAssignment variable="S1">
                    <math xmlns="http://www.w3.org/1998/Math/MathML">
                        <ci> p1 </ci>
                    </math>
                  </eventAssignment>
                  <eventAssignment variable="S2">
                    <math xmlns="http://www.w3.org/1998/Math/MathML">
                        <ci> p2 </ci>
                    </math>
                  </eventAssignment>
                </listOfEventAssignments>
            </event>
        </listOfEvents>
    </model>
</sbml>
```

There are three species in the model above whose values may vary. The first set of equations to consider is the set produced by the **Reaction** and the **AlgebraicRule** objects:

$$\frac{d[S_1]}{dt} = -C \cdot k_1 \cdot [S_1]$$

$$\frac{d[S_2]}{dt} = C \cdot k_1 \cdot [S_1]$$

$$[S_1] - [S_3] = 0$$

This set of equations is fully determined, i.e., each of the three variables $S_1$, $S_2$ and $S_3$ are derived from one equation. The second set of equations to consider is produced by the **Event** and the **AlgebraicRule** objects:

$$[S_1] = 1$$

$$[S_2] = 1.5$$

$$[S_1] - [S_3] = 0$$

Again the set of equations is fully determined, but had the event assignment for species $S_1$ been absent, the algebraic rule would produce an ambiguity regarding which variable should be adjusted.

In this example, as is often the case when an **AlgebraicRule** has been used, the **AlgebraicRule** could be replaced by an **AssignmentRule**:

```
<assignmentRule variable="S3">
    <math xmlns="http://www.w3.org/1998/Math/MathML">
       <ci> S1 </ci>
    </math>
</assignmentRule>
```

Replacing **AlgebraicRule** objects with **AssignmentRule** objects, particularly in models that use events, reduces the possibilities for creating either overdetermined or ambiguous models and produces models that can be exchanged with greater ease.

##### *8.2.4 Reactions*

Consider a very simple model consisting of a single enzymatic reaction $R$ that converts $S_1$ to $S_2$ for which a traditional kinetic law $v_R$ is given:

$$\mathrm{S}_1 \xrightarrow{v_R} \mathrm{S}_2$$

where

$$v_R = \frac{v_{\mathrm{max}} \cdot [S_1]}{K_{\mathrm{M}} + [S_1]}$$

with $v_R$ and $v_{\mathrm{max}}$ given in units of concentration per time.

As mentioned above, when a rate law is presented in the traditional way, it usually embodies (implicitly or explicitly) several assumptions: that all species are located in the same compartment, that the compartment size does not change, and that the reaction takes place uniformly throughout the volume of the compartment, i.e. the enzyme is not localized in any special way. Under these circumstances it is possible to construct rate equations for the *concentration* of the species:

$$\frac{d[S_2]}{dt} = -\frac{d[S_1]}{dt} = v_R$$

In SBML, however, the rate equations are constructed for the rate of change of the *amount* of the species:

$$\frac{dn_{S_2}}{dt} = -\frac{dn_{S_1}}{dt} = \hat{v}_R = V \cdot v_R$$

where $\hat{v}_R$ is the modified SBML kinetic law and $V$ is the volume of the compartment. Since the traditional kinetic law $v_R$ describes how fast the amount of the species changes *per volume*, the SBML kinetic law $\hat{v}_R$ simply equals the product of $v_R$ and the compartment volume $V$. This means that the actual rate of change of the amounts of the species is proportional to the compartment size, which will only be true if the reaction takes place uniformly throughout the compartment. (See Section 7.13 on p. 141 for an example of a reaction that is located at the boundary of a compartment.) The concentrations of the species (that are needed in the definition of $v_R$) can easily be recovered through the relation $[S_i] = n_{S_i}/V$.

An important property of the amount rate equation is that it is still valid if the volume $V$ changes during a simulation. This is not true for the concentration rate equations.

##### *8.2.5 Events*

The following recommendations concern **Event** objects and their subcomponents.

###### *Undefined ordering*

Section 4.12 on p. 79 describes how to interpret SBML events; however, the explanation explicitly leaves *undefined* how events should be ordered in the absense of priorites attached to the events. This curious omission in the specification reflects the state of agreement in biological modeling software today, but at the same time, it does not help software developers with the goal of implementing support for SBML events.

In practice, a variety of simple approaches can satisfy the “undefined ordering” requirement. For example, a software system could assign an arbitrary priority value to all events with undefined priorities. Another approach is for a simulator to execute the events in whatever order they happen to be stored in the implementation of the software. This part of SBML event behavior is left up to developers.

Regardless of the above, developers should keep in mind that the ordering requirements for events *with* **Priority** subobjects *are* defined, including for the case where such events in the model are mixed with events having undefined priorities. For example, if a model contains three simultaneously-firing events, one with priority 10, one with priority 4, and one with an undefined priority (call it $X$), there are three valid results for an implementation following the combined priority and "undefined ordering" requirements in SBML: 10-4-X, 10-X-4, and X-10-4. The implementation could always pick the same option among those three (as would happen if it assigned events with undefined priorities an artificial priority value, as mentioned above), or it could pick randomly between the three alternatives on different simulation runs, as it would if it were trying to be robustly stochastic. But the simulator should never execute the events in the order 4-10-X, nor should it quit unexpectedly. By defining the events in the model in this way, the creator of the model has clearly stated that the event with priority 10 should be executed before the event with priority 4, and that the event with $X$ must also be executed at *some* point. Beyond that, nothing more can be said or assumed about the modeler's intention.

#### *Simultaneous event execution*

Another concern with SBML events is how to implement true "simultaneous" execution of events. A model defines the conditions mathematically, but software realizations generally need to use numerical methods; the limited precision inherent with numerical methods can result in the system not executing precisely at the same time events that are meant to be simultaneous, or conversely, executing simultaneously some events that are *not* meant to be exactly simultaneous. Calculating the time of event execution depends on finding the trigger time, because an event's execution time is the sum (counting from the simulation start time) of the trigger time plus any delay in the event. If two events have the same delay but different triggers, they should trigger simultaneously if the moment that their trigger expressions transition to "`true`" is the same.

In part, the ultimate behavior may depend on how the modeler has written the model, and careful modelers will write models that are robust against slight numerical imprecision. For their part, software developers can take steps to increase the likelihood that the times at which trigger expressions transition in value are all detected equally, by doing such things as caching the calculated times at which embedded Boolean subexpressions in **Trigger** formulas switch their truth states. (For example, given two events, one with trigger expression $[(s1 > 5)\ and\ (s2 > 7)]$ and the other with trigger expression $[(s1 > 5)\ and\ (s3 > 10)]$, the time at which $s1$ transitions from less than 5 to greater than 5 can be calculated just once, cached, and reused thereafter, thereby helping to mitigate against small timing differences that might occur if the expression is reevaluated at different times.) If the trigger times are thus numerically identical, and if they have the same delay equation (which should also be cached if need be, for the same reason), they will then execute simultaneously, as they were intended to do in the model.

When creating models containing (e.g.) two events $A$ and $B$ that have different delays, model authors should not expect to achieve simultaneous execution simply by arranging for the sum of $A$'s trigger time plus $A$'s delay to be equal to the sum of $B$'s trigger time plus $B$'s delay. It is unlikely that different software implementations will resolve the execution times precisely in the same way, so it is unlikely the model will behave as the author expected in this scenario.

## A Validation and consistency rules for SBML

This section summarizes all the conditions that must (or in some cases, at least *should*) be true of a model encoded in SBML Level 3 Core format. We use the following conventions in the list of rules that follow:

- There are different degrees of rule strictness. Formally, the differences are expressed in the statement of a rule: either a rule states that a condition *must* be true, or a rule states that it *should* be true. Rules of the former kind are strict SBML validation rules—a model encoded in SBML must conform to all of them in order to be considered valid. Rules of the latter kind are consistency rules. To help highlight these differences, we use the following three symbols next to the rule numbers:
  - ☑ A checked box indicates a *requirement* for SBML conformance. If a model does not follow this rule, it does not conform to the SBML specification. (Mnemonic intention behind the choice of symbol: "This must be checked.")
  - ▲ A triangle indicates a *recommendation* for model consistency. If a model does not follow this rule, it is not considered strictly invalid as far as the SBML specification is concerned; however, it indicates that the model contains a physical or conceptual inconsistency. (Mnemonic intention behind the choice of symbol: "This is a cause for warning.")
  - ★ A star indicates a strong recommendation for good modeling practice. This rule is not strictly a matter of SBML encoding, but the recommendation comes from logical reasoning. As in the previous case, if a model does not follow this rule, it is not strictly considered an invalid SBML encoding. (Mnemonic intention behind the choice of symbol: "You're a star if you heed this.")
- Most rules have existed in previous Levels/Versions of SBML. Note that, because each SBML specification is independent, the precise details of a given rule in *this* specification may differ slightly from its formulation in other Levels/Versions, to reflect changes in SBML Level 3; however, the essential purpose of the rule will be the same.
- Rules that may have been introduced in lower Levels/Versions of SBML sometimes are removed in higher Levels/Versions. (This can happen, for example, if they become irrelevant due to changes in the language in a higher Level or Version of SBML.) Rule numbers, however, remain unique and are never reused for a different purpose. Consequently, there exist gaps in the sequence numbers of the rules.
- New rules introduced by this SBML Level 3 specification are indicated by an underlined rule number (e.g., 10104 instead of 10104).
- XML element attributes mentioned in the following validation and consistency rules have no XML namespace prefix, and must appear in SBML documents without any explicit XML namespace, even if the declared namespace is otherwise correct for SBML Level 3.

#### *General rules concerning basic XML requirements*

10101. ☑ An SBML XML file must use UTF-8 as the character encoding. More precisely, the `encoding` attribute of the XML declaration at the beginning of the XML data stream cannot have a value other than "`UTF-8`". An example valid declaration is `<?xml version="1.0" encoding="UTF-8"?>`. (References: SBML L3V1 Section 4.1; SBML L3V2 Section 4.1.)

10102. ☑ An SBML XML document must not contain undefined elements or attributes in the SBML Level 3 Core namespace or in a SBML Level 3 package namespace. Documents containing unknown elements or attributes placed in an SBML namespace do not conform to the SBML specification. (References: SBML L3V1 Section 4.1; SBML L3V2 Section 4.1.)

10104. ☑ An SBML document must conform to the rules of XML well-formedness defined in the XML 1.0 specification. These rules define the basic structural and syntactic constraints with which all XML documents must comply. (References: SBML L3V1 Section 4.1; SBML L3V2 Section 4.1.)

#### *General rules for MathML content in SBML*

10201. ☑ Wherever MathML content appears in an SBML document, the MathML content must be placed within a `math` element, and that `math` element must be either explicitly or implicitly declared to be in the XML namespace "`http://www.w3.org/1998/Math/MathML`". (References: SBML L3V1 Section 3.4; SBML L3V2 Section 3.4.)

10202. ☑ The following is a list of the only MathML 2.0 elements permitted in SBML Level 3: `abs`, `and`, `annotation`, `annotation-xml`, `apply`, `arccosh`, `arccos`, `arccoth`, `arccot`, `arccsch`, `arccsc`, `arcsech`, `arcsec`, `arcsinh`, `arcsin`, `arctanh`, `arctan`, `bvar`, `ceiling`, `ci`, `cn`, `cosh`, `cos`, `coth`, `cot`, `csch`, `csc`, `csymbol`, `degree`, `divide`, `eq`, `exponentiale`, `exp`, `factorial`, `false`, `floor`, `geq`, `gt`, `implies`, `infinity`, `lambda`, `leq`, `ln`, `logbase`, `log`, `lt`, `max`, `min`, `minus`, `neq`, `notanumber`, `not`, `or`, `otherwise`, `piecewise`, `piece`, `pi`, `plus`, `power`, `quotient`, `rem`, `root`, `sech`, `sec`, `semantics`, `sep`, `sinh`, `sin`, `tanh`, `tan`, `times`, `true`, and `xor`. An SBML package may allow new MathML elements to be added to this list, and if so, the package must define `required`="`true`" on the SBML container element `<sbml>`. (References: SBML L3V1 Section 3.4.1; SBML L3V2 Section 3.4.1.)

10203. ☑ In the SBML subset of MathML 2.0, the MathML attribute `encoding` is only permitted on `csymbol`, `annotation` and `annotation-xml`. No other MathML elements may have an `encoding` attribute. An SBML package may allow the `encoding` attribute on other elements, and if so, the package must define `required`="`true`" on the SBML container element `<sbml>`. (References: SBML L3V1 Section 3.4.1; SBML L3V2 Section 3.4.1.)

10204. ☑ In the SBML subset of MathML 2.0, the MathML attribute `definitionURL` is only permitted on `ci`, `csymbol` and `semantics`. No other MathML elements may have a `definitionURL` attribute. An SBML package may allow the `definitionURL` attribute on other elements, and if so, the package must define `required`="`true`" on the SBML container element `<sbml>`. (References: SBML L3V1 Section 3.4.1; SBML L3V2 Section 3.4.1.)

10205. ☑ In SBML Level 3, the only values permitted for the attribute `definitionURL` on a `csymbol` are "`http://www.sbml.org/sbml/symbols/time`", "`http://www.sbml.org/sbml/symbols/delay`", "`http://www.sbml.org/sbml/symbols/avogadro`", and "`http://www.sbml.org/sbml/symbols/rateOf`". An SBML package may allow new values for the `definitionURL` attribute of a `csymbol`, and if so, the package must define `required`="`true`" on the SBML container element `<sbml>`. (References: SBML L3V1 Section 3.4.6; SBML L3V2 Section 3.4.6.)

10206. ☑ In the SBML subset of MathML 2.0, the MathML attribute `type` is only permitted on the `cn` construct. No other MathML elements may have a `type` attribute. An SBML package may allow the `type` attribute on other elements, and if so, the package must define `required`="`true`" on the SBML container element `<sbml>`. (References: SBML L3V1 Section 3.4.1; SBML L3V2 Section 3.4.1.)

10207. ☑ The only permitted values for the attribute `type` on MathML `cn` elements are "`e-notation`", "`real`", "`integer`", and "`rational`". An SBML package may allow new values for the `type` attribute, and if so, the package must define `required`="`true`" on the SBML container element `<sbml>`. (References: SBML L3V1 Section 3.4.2; SBML L3V2 Section 3.4.2.)

10208. ☑ MathML `lambda` elements are only permitted as either the first element inside the `math` element of a **FunctionDefinition** object, or as the first element of a `semantics` element immediately inside the `math` element of a **FunctionDefinition** object. MathML `lambda` elements may not be used elsewhere in an SBML model. An SBML package may allow `lambda` elements on other elements, and if so, the package must define `required`="`true`" on the SBML container element `<sbml>`. (References: SBML L3V1 Section 3.4.1 and 4.3.2; SBML L3V2 Section 3.4.1 and Section 4.3.2.)

10209. ☑ (This validation rule does not apply in SBML Level 3 Version 2 Core.) [1]

[1]Original rule: "The arguments of the MathML logical operators `and`, `not`, `or`, and `xor` must evaluate to Boolean values."

10210. ☑ (This validation rule does not apply in SBML Level 3 Version 2 Core.) [2]

10211. ☑ (This validation rule does not apply in SBML Level 3 Version 2 Core.) [3]

10212. ▲ (This validation rule does not apply in SBML Level 3 Version 2 Core.) [4]

10213. ☑ (This validation rule does not apply in SBML Level 3 Version 2 Core.) [5]

10214. ☑ Outside of a **FunctionDefinition** object, if a MathML `ci` element is the first element within a MathML `apply` element, then the `ci` element's value can only be chosen from the set of identifiers of **FunctionDefinition** objects defined in the enclosing SBML **Model** object. (References: SBML L3V1 Section 4.3.2; SBML L3V2 Section 4.3.2.)

10215. ☑ Outside of a **FunctionDefinition** object, if a MathML `ci` element is not the first element within a MathML `apply`, then the `ci` element's value may only be chosen from the following set of identifiers: the identifiers of **Species**, **Compartment**, **Parameter**, **SpeciesReference** and **Reaction** objects defined in the enclosing **Model** object; the identifiers of **LocalParameter** objects that are children of the **Reaction** in which the **FunctionDefinition** appears (if it appears inside the **Math** object of a **KineticLaw**); and any identifiers (in the `SId` namespace of the model) belonging to an object class defined by an SBML Level 3 package as having mathematical meaning. (References: SBML L3V1 Section 3.4.3; SBML L3V2 Section 3.4.3.)

10216. ☑ The `id` attribute value of a **LocalParameter** object defined within a **KineticLaw** object may only be used, in core, in MathML `ci` elements within the `math` element of that same **KineticLaw**; in other words, the identifier of the **LocalParameter** object is not visible to other parts of the model outside of that **Reaction** instance. In package constructs, the `id` attribute value of a **LocalParameter** object may only be used in MathML `ci` elements or as the target of an `SIdRef` attribute if that package construct is a child of the parent **Reaction**. (References: SBML L3V1 Section 3.3.1, 3.4.3, and 4.11.5; SBML L3V2 Section 3.3.1, Section 3.4.3, and Section 4.11.5.)

10217. ☑ (This validation rule does not apply in SBML Level 3 Version 2 Core.) [6]

10218. ☑ A MathML operator must be supplied the number of arguments appropriate for that operator. (References: SBML L3V1 Section 3.4.1; SBML L3V2 Section 3.4.1.)

10219. ☑ The number of arguments used in a call to a function defined by a **FunctionDefinition** object must equal the number of arguments accepted by that function, if defined. In other words, it must equal the number of MathML `bvar` elements inside the `lambda` element of the function definition, if present. (References: SBML L3V1 Section 4.3.4; SBML L3V2 Section 4.3.4.)

10220. ☑ The SBML attribute `units` may only be added to MathML `cn` elements; no other MathML elements are permitted to have the `units` attribute. An SBML package may allow the `units` attribute on other elements, and if so, the package must define `required`="`true`" on the SBML container element `<sbml>`. (References: SBML L3V1 Section 3.4.2; SBML L3V2 Section 3.4.2.)

10221. ☑ The value of the SBML attribute `units` on a MathML `cn` element must be chosen from either the set of identifiers of **UnitDefinition** objects in the model, or the set of base units defined by SBML. (References: SBML L3V1 Section 3.4.2; SBML L3V2 Section 3.4.2.)

[2] Original rule: "The arguments to the following MathML constructs must evaluate to numeric values (more specifically, they must evaluate to MathML real, integer, rational, or "e-notation" numbers, or the *time*, *delay*, *avogadro*, `csymbol` elements): `abs`, `arccosh`, `arccos`, `arccoth`, `arccot`, `arccsch`, `arccsc`, `arcsech`, `arcsec`, `arcsinh`, `arcsin`, `arctanh`, `arctan`, `ceiling`, `cosh`, `cos`, `coth`, `cot`, `csch`, `csc`, `divide`, `exp`, `factorial`, `floor`, `ln`, `log`, `minus`, `plus`, `power`, `root`, `sech`, `sec`, `sinh`, `sin`, `tanh`, `tan`, and `times`."

[3] Original rule: "The values of all arguments to MathML `eq` and `neq` operators must evaluate to the same type, either all Boolean or all numeric."

[4] Original rule: "The types of the values within MathML `piecewise` operators should all be consistent; i.e., the set of expressions that make up the first arguments of the `piece` and `otherwise` operators within the same `piecewise` operator should all return values of the same type."

[5] Original rule: "The second argument of a MathML `piece` operator must evaluate to a Boolean value."

[6] Original rule: "The MathML formulas in the following elements must yield numeric values (that is, MathML real, integer or "e-notation" numbers, or the *time*, *delay*, *avogadro*, or *rateOf* `csymbol`): `math` in **KineticLaw**, `math` in **InitialAssignment**, `math` in **AssignmentRule**, `math` in **RateRule**, `math` in **AlgebraicRule**, `math` in **Event Delay**, and `math` in **EventAssignment**."

10223. ☑ The single argument for the *rateOf* `csymbol` function must be a `ci` element. (Reference: SBML L3V2 Section 3.4.6)

10224. ☑ The target of a *rateOf* `csymbol` function must not appear as the `variable` of an **AssignmentRule**, nor may its value be determined by an **AlgebraicRule**. (Reference: SBML L3V2 Section 3.4.6)

10225. ☑ If the target of a *rateOf* `csymbol` function is a **Species** with a `hasOnlySubstanceUnits` value of "`false`", the `compartment` of that **Species** must not appear as the `variable` of an **AssignmentRule**, nor may its `size` be determined by an **AlgebraicRule**. (Reference: SBML L3V2 Section 3.4.6)

#### *General rules for identifiers*

10301. ☑ The value of the attribute `id` on every instance of the following classes of objects must be unique across the set of all `id` attribute values of all such objects in a model: **AlgebraicRule**, **AssignmentRule**, **Compartment**, **Constraint**, **Delay**, **Event**, **EventAssignment**, **FunctionDefinition**, **InitialAssignment**, **KineticLaw**, **ListOfCompartments**, **ListOfConstraints**, **ListOfEventAssignments**, **ListOfEvents**, **ListOfFunctionDefinitions**, **ListOfInitialAssignments**, **ListOfLocalParameters**, **ListOfModifierSpeciesReferences**, **ListOfParameters**, **ListOfReactions**, **ListOfRules**, **ListOfSpecies**, **ListOfSpeciesReferences**, **ListOfUnitDefinitions**, **ListOfUnits**, **Model**, **ModifierSpeciesReference**, **Parameter**, **Priority**, **RateRule**, **Reaction**, **Species**, **SpeciesReference**, **Trigger**, and **Unit**, plus the `id` attribute values of any SBML Level 3 package element defined to be in the `SId` namespace of the model. (References: SBML L3V1 Section 3.3; SBML L3V2 Section 3.3.)

10302. ☑ The value of the attribute `id` of every **UnitDefinition** object must be unique across the set of all the **UnitDefinition** objects in the entire model. (References: SBML L3V1 Section 3.3 and 4.4; SBML L3V2 Section 3.3 and Section 4.4.)

10303. ☑ The value of the attribute `id` of every **LocalParameter** object defined within a **KineticLaw** object must be unique across the set of all such parameter definitions within that particular **KineticLaw** instance. (References: SBML L3V1 Section 3.3.1 and 4.11.5; SBML L3V2 Section 3.3.1 and Section 4.11.5.)

10304. ☑ The value of the attribute `variable` of every **AssignmentRule** and **RateRule** objects must be unique across the set of all **AssignmentRule** and **RateRule** objects in a model. In other words, a given model component cannot be the subject of both an assignment rule and a rate rule simultaneously. (References: SBML L3V1 Section 4.9.3; SBML L3V2 Section 4.9.3.)

10305. ☑ In every **Event** object, the value of the attribute `variable` within each **EventAssignment** subobject must be unique across the set of all such **EventAssignment** subobjects within that particular **Event** object. In other words, a single **Event** cannot make more than one assignment to the same model component. (References: SBML L3V1 Section 4.12.5; SBML L3V2 Section 4.12.5.)

10306. ☑ An identifier used as the value of the attribute `variable` of an **EventAssignment** object cannot also appear as the value of the `variable` attribute in an **AssignmentRule** object. In other words, a given model component cannot be the subject of both an assignment rule and an assignment by an event. (References: SBML L3V1 Section 4.12.5; SBML L3V2 Section 4.12.5.)

10307. ☑ Every `metaid` attribute value must be unique across the set of all `metaid` values in a model. (References: SBML L3V1 Section 3.2.1 and 3.1.6; SBML L3V2 Section 3.2.3 and Section 3.1.6.)

10308. ☑ The value of the attribute `sboTerm` must always conform to the syntax of the SBML data type `SBOTerm`, which is a string consisting of the characters 'S', 'B', 'O', ':', followed by exactly seven digits. (References: SBML L3V1 Section 3.1.11; SBML L3V2 Section 3.1.12.)

10309. ☑ The value of a `metaid` attribute must always conform to the syntax of the XML data type `ID`. (References: SBML L3V1 Section 3.2.1 and 3.1.6; SBML L3V2 Section 3.2.3 and Section 3.1.6.)

10310. ☑ The value of an `id` attribute must always conform to the syntax of the SBML data type `SId`. (References: SBML L3V1 Section 3.1.7; SBML L3V2 Section 3.1.7.)

10311. ☑ Unit identifiers (that is, the values of the `id` attribute on **UnitDefinition**, the `units` attribute on **Compartment**, the `units` attribute on **Parameter**, the `units` attribute on **LocalParameter**, the `substanceUnits` attribute on **Species**, the SBML `units` attribute on MathML `cn` elements, and the `substanceUnits`, `volumeUnits`, `areaUnits`, `lengthUnits`, `timeUnits` and `extentUnits` on **Model**) must always conform to the syntax of the SBML data type `UnitSId`. (References: SBML L3V1 Section 3.1.9; SBML L3V2 Section 3.1.9.)

10312. ☑ The value of a `name` attribute must always conform to the syntax of type `string`. (References: SBML L3V1 Section 3.1.1; SBML L3V2 Section 3.1.1.)

10313. ☑ Unit identifier references (that is, the `units` attribute on **Compartment**, the `units` attribute on **Parameter**, the `units` attribute on **LocalParameter**, the `substanceUnits` attribute on **Species**, the SBML `units` attribute on MathML `ci` elements, and the `substanceUnits`, `volumeUnits`, `areaUnits`, `lengthUnits`, `timeUnits` and `extentUnits` on **Model**) must be the identifier of a **UnitDefinition** in the **Model**, or the identifier of a predefined unit in SBML, that is, any of the following base units: "`ampere`", "`avogadro`", "`becquerel`", "`candela`", "`coulomb`", "`dimensionless`", "`farad`", "`gram`", "`gray`", "`henry`", "`hertz`", "`item`", "`joule`", "`katal`", "`kelvin`", "`kilogram`", "`litre`", "`lumen`", "`lux`", "`metre`", "`mole`", "`newton`", "`ohm`", "`pascal`", "`radian`", "`second`", "`siemens`", "`sievert`", "`steradian`", "`tesla`", "`volt`", "`watt`", or "`weber`". (References: SBML L3V1 Section 4.4.2; SBML L3V2 Section 4.4.2.)

#### *General rules for `annotation` elements*

10401. ☑ Every top-level XML element within an **Annotation** object must have an XML namespace declared. (References: SBML L3V1 Section 3.2.4; SBML L3V2 Section 3.2.6.)

10402. ☑ A given XML namespace cannot be the namespace of more than one top-level element within a given **Annotation** object. (References: SBML L3V1 Section 3.2.4; SBML L3V2 Section 3.2.6.)

10404. ☑ A given SBML element may contain at most one **Annotation** subobject. (References: SBML L3V1 Section 3.2; SBML L3V2 Section 3.2.)

#### *General rules for units*

10501. ▲ The units of the expressions used as arguments to a function call should match the units expected for the arguments of that function. (References: SBML L3V1 Section 3.4; SBML L3V2 Section 3.4.)

10503. ▲ The unit of measurement associated with the mathematical formula in the MathML `math` element of every **KineticLaw** object in a model should be identical to all **KineticLaw** objects in the model. (References: SBML L3V1 Section 3.4; SBML L3V2 Section 3.4.)

10511. ▲ When the value of the attribute `variable` in an **AssignmentRule** object refers to a **Compartment** object, the unit of measurement associated with the mathematical expression in the rule's MathML `math` element should be consistent with the unit of that compartment's size. (References: SBML L3V1 Section 4.9.3; SBML L3V2 Section 4.9.3.)

10512. ▲ When the value of the attribute `variable` in an **AssignmentRule** object refers to a **Species** object, the unit of measurement associated with the mathematical expression in the rule's MathML `math` element should be consistent with the unit of that species' quantity. (References: SBML L3V1 Section 4.9.3; SBML L3V2 Section 4.9.3.)

10513. ▲ When the value of the attribute `variable` in an **AssignmentRule** object refers to a **Parameter** object, the unit of measurement associated with the mathematical expression in the rule's MathML `math` element should be consistent with the unit declared for that parameter's value. (References: SBML L3V1 Section 4.9.3; SBML L3V2 Section 4.9.3.)

10514. ▲ When the value of the attribute `variable` in an **AssignmentRule** object refers to a **SpeciesReference** object, the unit of measurement associated with the rule's right-hand side should be consistent with the unit of stoichiometry, that is, `dimensionless`. (References: SBML L3V1 Section 4.9.3; SBML L3V2 Section 4.9.3.)

10521. ▲ When the value of the attribute `variable` in an **InitialAssignment** object refers to a **Compartment** object, the unit of measurement associated with the **InitialAssignment**'s `math` expression should be consistent with the unit of that compartment's size. (References: SBML L3V1 Section 4.8; SBML L3V2 Section 4.8.)

10522. ▲ When the value of the attribute `variable` in an **InitialAssignment** object refers to a **Species** object, the unit of measurement associated with the **InitialAssignment**'s `math` expression should be consistent with the unit of that species' quantity. (References: SBML L3V1 Section 4.8; SBML L3V2 Section 4.8.)

10523. ▲ When the value of the attribute `variable` in an **InitialAssignment** object refers to a **Parameter** object, the unit of measurement associated with the **InitialAssignment**'s `math` expression should be consistent with the unit declared for that parameter's value. (References: SBML L3V1 Section 4.8; SBML L3V2 Section 4.8.)

10524. ▲ When the value of the attribute `variable` in an **InitialAssignment** object refers to a **SpeciesReference** object, the unit of measurement associated with the **InitialAssignment**'s `math` expression should be consistent with the unit of stoichiometry, that is, `dimensionless`. (References: SBML L3V1 Section 4.8; SBML L3V2 Section 4.8.)

10531. ▲ When the value of the attribute `variable` in a **RateRule** object refers to a **Compartment** object, the unit of measurement associated with the **RateRule**'s `math` expression should be consistent with {unit of compartment size}/{unit of *time*}. (References: SBML L3V1 Section 4.5.4, 4.2.4, and 4.9.4; SBML L3V2 Section 4.5.4, Section 4.2.3, and Section 4.9.4.)

10532. ▲ When the value of the attribute `variable` in a **RateRule** object refers to a **Species** object, the unit of measurement associated with the **RateRule**'s `math` expression should be consistent with {unit of species quantity}/{unit of *time*}. (References: SBML L3V1 Section 4.6.5, 4.2.4, and 4.9.4; SBML L3V2 Section 4.6.5, Section 4.2.3, and Section 4.9.4.)

10533. ▲ When the value of the attribute `variable` in a **RateRule** object refers to a **Parameter** object, the unit of measurement associated with the **RateRule**'s `math` expression should be consistent with {parameter's `units`}/{unit of *time*}. (References: SBML L3V1 Section 4.7.3, 4.2.4, and 4.9.4; SBML L3V2 Section 4.7.3, Section 4.2.3, and Section 4.9.4.)

10534. ▲ When the value of the attribute `variable` in a **RateRule** object refers to a **SpeciesReference** object, the unit of measurement associated with the **RateRule**'s `math` expression should be consistent with {unit derived from `dimensionless`}/{unit of *time*}. (References: SBML L3V1 Section 4.11.3, 4.2.4, and 4.9.4; SBML L3V2 Section 4.11.3, Section 4.2.3, and Section 4.9.4.)

10541. ▲ In a **KineticLaw** object, the unit of measurement associated with the formula in the **KineticLaw**'s `math` expression should be equal to {unit of reaction *extent*}/{unit of *time*}. (References: SBML L3V1 Section 4.11.7, 4.2.4, and 4.9.4; SBML L3V2 Section 4.11.7, Section 4.2.3, and Section 4.9.4.)

10542. ▲ For every **Species** object produced or consumed in a reaction (that is, referenced by a **SpeciesReference** object), the unit of measurement of the species' substance should be consistent with the unit of *extent* for the model times the unit of the conversion factor for that species. More precisely, the product of the units indicated by the **Model** object's `extentUnits` and the `conversionFactor` attribute for that particular **Species** (whether the attribute is set directly on the **Species** object or inherited from the enclosing **Model** object) should be consistent with the unit specified by that **Species** object's `substanceUnits` attribute value. (References: SBML L3V1 Section 4.2.6; SBML L3V2 Section 4.2.5.)

10551. ▲ In an **Event** object, the unit of measurement associated with a **Delay** object's `math` expression object should be identical to the unit indicated by the **Model** object's `timeUnits` attribute. (References: SBML L3V1 Section 4.12.4; SBML L3V2 Section 4.12.4.)

10561. ▲ When the value of the attribute `variable` of an **EventAssignment** object is the identifier of a **Compartment** object, the unit of measurement associated with the **EventAssignment**'s `math` expression should be consistent with the unit of that compartment's size. (References: SBML L3V1 Section 4.12.5; SBML L3V2 Section 4.12.5.)

10562. ▲ When the value of the attribute `variable` of an **EventAssignment** object is the identifier of a **Species** object, the unit of measurement associated with the **EventAssignment**'s `math` expression should be consistent with the unit of that species' size. (References: SBML L3V1 Section 4.12.5; SBML L3V2 Section 4.12.5.)

10563. ▲ When the value of the attribute `variable` of an **EventAssignment** object is the identifier of a **Parameter** object, the unit of measurement associated with the **EventAssignment**'s `math` expression should be consistent with the unit declared for that parameter's value. (References: SBML L3V1 Section 4.12.5; SBML L3V2 Section 4.12.5.)

10564. ▲ When the value of the attribute `variable` of an **EventAssignment** object is the identifier of a **SpeciesReference** object, the unit of measurement associated with the **EventAssignment**'s `math` expression should be consistent with the unit of stoichiometry, i.e., `dimensionless`. (References: SBML L3V1 Section 4.12.5; SBML L3V2 Section 4.12.5.)

10565. ▲ In an **Event** object, the unit of measurement associated with a **Priority** object's `math` expression object should be `dimensionless`. (References: SBML L3V1 Section 4.12.3; SBML L3V2 Section 4.12.3.)

#### *General rules for model definitions*

10601. ☑ A system of equations created from an SBML model must not be overdetermined. (References: SBML L3V1 Section 4.9.5; SBML L3V2 Section 4.9.5.)

#### *General rules for SBO usage*

10701. ▲ The value of the attribute `sboTerm` on a **Model** object should be an SBO identifier referring to an interaction or modeling framework defined in SBO. That is, the value should be a term derived from `SBO:0000231`, "occurring entity representation", or `SBO:0000004`, "modeling framework". (References: SBML L3V1 Section 5; SBML L3V2 Section 5.)

10702. ▲ The value of the attribute `sboTerm` on a **FunctionDefinition** object should be an SBO identifier referring to a mathematical expression. That is, the value should be a term derived from `SBO:0000064`, "mathematical expression". (References: SBML L3V1 Section 5; SBML L3V2 Section 5.)

10703. ▲ The value of the attribute `sboTerm` on a **Parameter** object should be an SBO identifier referring to a quantitative parameter. That is, the value should be a term derived from `SBO:0000545`, "systems description parameter". (References: SBML L3V1 Section 5; SBML L3V2 Section 5.)

10704. ▲ The value of the attribute `sboTerm` on an **InitialAssignment** object should be an SBO identifier referring to a mathematical expression. That is, the value should be a term derived from `SBO:0000064`, "mathematical expression". (References: SBML L3V1 Section 5; SBML L3V2 Section 5.)

10705. ▲ The value of the attribute `sboTerm` on a **AlgebraicRule**, **RateRule** or **AssignmentRule** object should be an SBO identifier referring to a mathematical expression. That is, the value should be a term derived from `SBO:0000064`, "mathematical expression". (References: SBML SBML L3V1 Section 5; SBML L3V2 Section 5.)

10706. ▲ The value of the attribute `sboTerm` on a **Constraint** object should be an SBO identifier referring to a mathematical expression. That is, the value should be a term derived from `SBO:0000064`, "mathematical expression". (References: SBML L3V1 Section 5; SBML L3V2 Section 5.)

10707. ▲ The value of the attribute `sboTerm` on a **Reaction** object should be an SBO identifier referring to an interaction framework. That is, the value should be a term derived from `SBO:0000231`, "occurring entity representation". (References: SBML L3V1 Section 5; SBML L3V2 Section 5.)

10708. ▲ The value of the attribute sboTerm on a **SpeciesReference** or a **ModifierSpeciesReference** object should be an SBO identifier referring to a participant role. That is, the value should be a term derived from SBO:0000003, "participant role". The appropriate term depends on whether the entity is a reactant, product or modifier. (References: SBML L3V1 Section 5; SBML L3V2 Section 5.)

10709. ▲ The value of the attribute sboTerm on a **KineticLaw** object should be an SBO identifier referring to a rate law. That is, the value should be a term derived from SBO:0000001, "rate law". (References: SBML L3V1 Section 5; SBML L3V2 Section 5.)

10710. ▲ The value of the attribute sboTerm on an **Event** object should be an SBO identifier referring to a mathematical expression. That is, the value should be a term derived from SBO:0000231, "occurring entity representation". (References: SBML L3V1 Section 5; SBML L3V2 Section 5.)

10711. ▲ The value of the attribute sboTerm on an **EventAssignment** object should be an SBO identifier referring to a mathematical expression. That is, the value should be a term derived from SBO:0000064, "mathematical expression". (References: SBML L3V1 Section 5; SBML L3V2 Section 5.)

10712. ▲ The value of the attribute sboTerm on a **Compartment** object should be an SBO identifier referring to a material entity. That is, the value should be a term derived from SBO:0000236, "physical entity representation". (References: SBML L3V1 Section 5; SBML L3V2 Section 5.)

10713. ▲ The value of the attribute sboTerm on a **Species** object should be an SBO identifier referring to a material entity. That is, the value should be a term derived from SBO:0000236, "physical entity representation". (References: SBML L3V1 Section 5; SBML L3V2 Section 5.)

10716. ▲ The value of the attribute sboTerm on a **Trigger** object should be an SBO identifier referring to a mathematical expression. That is, the value should be a term derived from SBO:0000064, "mathematical expression". (References: SBML L3V1 Section 5; SBML L3V2 Section 5.)

10717. ▲ The value of the attribute sboTerm on a **Delay** object should be an SBO identifier referring to a mathematical expression. That is, the value should be a term derived from SBO:0000064, "mathematical expression". (References: SBML L3V1 Section 5; SBML L3V2 Section 5.)

10718. ▲ The value of the attribute sboTerm on a **LocalParameter** object should be an SBO identifier referring to a quantitative parameter. That is, the value should be a term derived from SBO:0000545, "systems description parameter". (References: SBML L3V1 Section 5; SBML L3V2 Section 5.)

10719. ▲ The value of the attribute sboTerm on the **SBML** object should be an SBO identifier referring to a modeling framework. That is, the value should be a term derived from SBO:0000004, "modeling framework". (References: SBML L3V2 Section 5.)

#### *General rules for* notes *elements*

10801. ☑ The contents of a **Notes** object must be explicitly placed in the XHTML XML namespace. (References: SBML L3V1 Section 3.2.3; SBML L3V2 Section 3.2.5.)

10802. ☑ The contents of a **Notes** object must not contain an XML declaration, i.e., a string of the form "<?xml version="1.0" encoding="UTF-8"?>" or similar. (References: SBML L3V1 Section 3.2.3; SBML L3V2 Section 3.2.5.)

10803. ☑ The content of a **Notes** object must not contain an XML DOCTYPE declaration, i.e., a string beginning with the characters "<!DOCTYPE". (References: SBML L3V1 Section 3.2.3; SBML L3V2 Section 3.2.5.)

10805. ☑ A given SBML object may contain at most one **Notes** subobject. (References: SBML L3V1 Section 3.2; SBML L3V2 Section 3.2.)

#### *Rules for the* `<sbml>` *container element*

20101. ☑ The `sbml` container element must declare the XML Namespace for SBML, and this declaration must be consistent with the values of the `level` and `version` attributes on the `sbml` element. (References: SBML L3V1 Section 4.1; SBML L3V2 Section 4.1.)

20102. ☑ The `sbml` container element must declare the SBML Level using the attribute `level`, and this declaration must be consistent with the XML Namespace declared for the `sbml` element. (References: SBML L3V1 Section 4.1; SBML L3V2 Section 4.1.)

20103. ☑ The `sbml` container element must declare the SBML Version using the attribute `version`, and this declaration must be consistent with the XML Namespace declared for the `sbml` element. (References: SBML L3V1 Section 4.1; SBML L3V2 Section 4.1.)

20104. ☑ The `sbml` container element must declare the XML Namespace for any SBML Level 3 packages used within the SBML document. This declaration must be consistent with the values of the `level` and `version` attributes on the `sbml` element. (References: SBML L3V1 Section 4.1.2; SBML L3V2 Section 4.1.3.)

20105. ☑ The attribute `level` on the `sbml` container element must have a value of type `positiveInteger`. (References: SBML L3V1 Section 3.1.4; SBML L3V2 Section 3.1.4.)

20106. ☑ The attribute `version` on the `sbml` container element must have a value of type `positiveInteger`. (References: SBML L3V1 Section 3.1.4; SBML L3V2 Section 3.1.4.)

20107. ☑ The attribute `xmlns` on the `sbml` container element must have a value of type `string`. (References: SBML L3V1 Section 3.1.1; SBML L3V2 Section 3.1.1.)

20108. ☑ The `sbml` object may have the optional attributes `id`, `name`, `metaid` and `sboTerm`. (References: SBML L3V1 Section 4.2.8; SBML L3V2 Section 4.2.7.)

#### *Rules for Model components*

20201. ☑ An SBML document may contain at most one **Model** object. (References: SBML L3V1 Section 4.1; SBML L3V2 Section 4.1).

20203. ☑ (This validation rule does not apply in SBML Level 3 Version 2 Core.) [7]

20204. ☑ If a model defines any **Species** object, then the model must also define at least one **Compartment** object. This is an implication of the fact that the `compartment` attribute on **Species** is not optional. (References: SBML L3V1 Section 4.6.3; SBML L3V2 Section 4.6.3.)

20205. ☑ There may be at most one instance of each of the following kind of object in a **Model** object: **ListOfFunctionDefinitions**, **ListOfUnitDefinitions**, **ListOfCompartments**, **ListOfSpecies**, **ListOfParameters**, **ListOfInitialAssignments**, **ListOfRules**, **ListOfConstraints**, **ListOfReactions** and **ListOfEvents**. (References: SBML L3V1 Section 4.2; SBML L3V2 Section 4.2.)

20206. ☑ Apart from the general notes and annotation subobjects permitted on all SBML components, a **ListOfFunctionDefinitions** container object may only contain **FunctionDefinition** objects. (References: SBML L3V1 Section 4.2.8; SBML L3V2 Section 4.2.7.)

20207. ☑ Apart from the general notes and annotation subobjects permitted on all SBML components, a **ListOfUnitDefinitions** container object may only contain **UnitDefinition** objects. (References: SBML L3V1 Section 4.2.8; SBML L3V2 Section 4.2.7.)

[7] Original rule: "The various `listOf_` container objects in a **Model** instance are optional, but if present, such container elements must not be empty. Specifically, if any of the following is present in a **Model**, it must not be empty: **ListOfFunctionDefinitions**, **ListOfUnitDefinitions**, **ListOfCompartments**, **ListOfSpecies**, **ListOfParameters**, **ListOfInitialAssignments**, **ListOfRules**, **ListOfConstraints**, **ListOfReactions** and **ListOfEvents**."

20208. ☑ Apart from the general notes and annotation subobjects permitted on all SBML components, a **ListOfCompartments** container object may only contain **Compartment** objects. (References: SBML L3V1 Section 4.2.8; SBML L3V2 Section 4.2.7.)

20209. ☑ Apart from the general notes and annotation subobjects permitted on all SBML components, a **ListOfSpecies** container object may only contain **Species** objects. (References: SBML L3V1 Section 4.2.8; SBML L3V2 Section 4.2.7.)

20210. ☑ Apart from the general notes and annotation subobjects permitted on all SBML components, a **ListOfParameters** container object may only contain **Parameter** objects. (References: SBML L3V1 Section 4.2.8; SBML L3V2 Section 4.2.7.)

20211. ☑ Apart from the general notes and annotation subobjects permitted on all SBML components, a **ListOfInitialAssignments** container object may only contain **InitialAssignment** objects. (References: SBML L3V1 Section 4.2.8; SBML L3V2 Section 4.2.7.)

20212. ☑ Apart from the general notes and annotation subobjects permitted on all SBML components, a **ListOfRules** container object may only contain **AssignmentRule**, **AlgebraicRule** and/or **RateRule** objects. (References: SBML L3V1 Section 4.2.8; SBML L3V2 Section 4.2.7.)

20213. ☑ Apart from the general notes and annotation subobjects permitted on all SBML components, a **ListOfConstraints** container object may only contain **Constraint** objects. (References: SBML L3V1 Section 4.2.8; SBML L3V2 Section 4.2.7.)

20214. ☑ Apart from the general notes and annotation subobjects permitted on all SBML components, a **ListOfReactions** container object may only contain **Reaction** objects. (References: SBML L3V1 Section 4.2.8; SBML L3V2 Section 4.2.7.)

20215. ☑ Apart from the general notes and annotation subobjects permitted on all SBML components, a **ListOfEvents** container object may only contain **Event** objects. (References: SBML L3V1 Section 4.2.8; SBML L3V2 Section 4.2.7.)

20216. ☑ The value of the attribute `conversionFactor` on a **Model** object must be the identifier of an existing **Parameter** object defined in the **Model** object's **ListOfParameters**. (References: SBML L3V1 Section 4.2; SBML L3V2 Section 4.2.)

20217. ★ The value of the attribute `timeUnits` on a **Model** object should be either the units "`second`", "`dimensionless`", or the identifier of a **UnitDefinition** object based on these units, or a combination of these units. (References: SBML L3V1 Section 4.2.4; SBML L3V2 Section 4.2.3.)

20218. ★ The value of the attribute `volumeUnits` on a **Model** object should be either the units "`litre`", "`dimensionless`", or the identifier of a **UnitDefinition** object based on these units or a unit derived from "`metre`", or a combination of these units. (References: SBML L3V1 Section 4.2.5; SBML L3V2 Section 4.2.4.)

20219. ★ The value of the attribute `areaUnits` on a **Model** object should be either "`dimensionless`" or the identifier of a **UnitDefinition** object based on "`dimensionless`" or a unit derived from "`metre`", or a combination of these units. (References: SBML L3V1 Section 4.2.5; SBML L3V2 Section 4.2.4.)

20220. ★ The value of the attribute `lengthUnits` on a **Model** object should be either the units "`metre`", "`dimensionless`", or the identifier of a **UnitDefinition** object based on these units, or a combination of these units. (References: SBML L3V1 Section 4.2.5; SBML L3V2 Section 4.2.4.)

20221. ★ The value of the attribute `extentUnits` on a **Model** object should be either the units "`mole`", "`item`", "`avogadro`", "`dimensionless`", "`kilogram`", "`gram`", or the identifier of a **UnitDefinition** object based on these units, or a combination of these units. (References: SBML L3V1 Section 4.2.6; SBML L3V2 Section 4.2.5.)

20222. ☑ A **Model** object may only have the following attributes, all of which are optional: `metaid`, `sboTerm`, `id`, `name`, `substanceUnits`, `timeUnits`, `volumeUnits`, `areaUnits`, `lengthUnits`, `extentUnits` and `conversionFactor`. No other attributes from the SBML Level 3 Core namespace are permitted on a **Model** object. (References: SBML L3V1 Section 4.2; SBML L3V2 Section 4.2.)

20223. ☑ A **ListOfFunctionDefinitions** object may have the optional attributes `id`, `name`, `metaid` and `sboTerm`. No other attributes from the SBML Level 3 Core namespace are permitted on a **ListOfFunctionDefinitions** object. (References: SBML L3V1 Section 4.2.8; SBML L3V2 Section 4.2.7.)

20224. ☑ A **ListOfUnitDefinitions** object may have the optional attributes `id`, `name`, `metaid` and `sboTerm`. No other attributes from the SBML Level 3 Core namespace are permitted on a **ListOfUnitDefinitions** object. (References: SBML L3V1 Section 4.2.8; SBML L3V2 Section 4.2.7.)

20225. ☑ A **ListOfCompartments** object may have the optional attributes `id`, `name`, `metaid` and `sboTerm`. No other attributes from the SBML Level 3 Core namespace are permitted on a **ListOfCompartments** object. (References: SBML L3V1 Section 4.2.8; SBML L3V2 Section 4.2.7.)

20226. ☑ A **ListOfSpecies** object may have the optional attributes `id`, `name`, `metaid` and `sboTerm`. No other attributes from the SBML Level 3 Core namespace are permitted on a **ListOfSpecies** object. (References: SBML L3V1 Section 4.2.8; SBML L3V2 Section 4.2.7.)

20227. ☑ A **ListOfParameters** object may have the optional attributes `id`, `name`, `metaid` and `sboTerm`. No other attributes from the SBML Level 3 Core namespace are permitted on a **ListOfParameters** object. (References: SBML L3V1 Section 4.2.8; SBML L3V2 Section 4.2.7.)

20228. ☑ A **ListOfInitialAssignments** object may have the optional attributes `id`, `name`, `metaid` and `sboTerm`. No other attributes from the SBML Level 3 Core namespace are permitted on a **ListOfInitialAssignments** object. (References: SBML L3V1 Section 4.2.8; SBML L3V2 Section 4.2.7.)

20229. ☑ A **ListOfRules** object may have the optional attributes `id`, `name`, `metaid` and `sboTerm`. No other attributes from the SBML Level 3 Core namespace are permitted on a **ListOfRules** object. (References: SBML L3V1 Section 4.2.8; SBML L3V2 Section 4.2.7.)

20230. ☑ A **ListOfConstraints** object may have the optional attributes `id`, `name`, `metaid` and `sboTerm`. No other attributes from the SBML Level 3 Core namespace are permitted on a **ListOfConstraints** object. (References: SBML L3V1 Section 4.2.8; SBML L3V2 Section 4.2.7.)

20231. ☑ A **ListOfReactions** object may have the optional attributes `id`, `name`, `metaid` and `sboTerm`. No other attributes from the SBML Level 3 Core namespace are permitted on a **ListOfReactions** object. (References: SBML L3V1 Section 4.2.8; SBML L3V2 Section 4.2.7.)

20232. ☑ A **ListOfEvents** object may have the optional attributes `id`, `name`, `metaid` and `sboTerm`. No other attributes from the SBML Level 3 Core namespace are permitted on a **ListOfEvents** object. (References: SBML L3V1 Section 4.2.8; SBML L3V2 Section 4.2.7.)

20233. ★ The value of the attribute `substanceUnits` on a **Model** object should be either the units "`mole`", "`item`", "`avogadro`", "`dimensionless`", "`kilogram`", "`gram`", or the identifier of a **UnitDefinition** object based on these units, or a combination of these units. (References: SBML L3V1 Section 4.2.3; SBML L3V2 Section 4.2.2.)

#### *Rules for FunctionDefinition components*

20301. ☑ The top-level element within the MathML `math` element in a **FunctionDefinition** object, if present, must be either exactly one MathML `lambda` element, or exactly one MathML `semantics` element containing exactly one `lambda` element. (References: SBML L3V1 Section 4.3.2; SBML L3V2 Section 4.3.2.)

20303. ☑ Inside the `lambda` MathML element within a **FunctionDefinition** object, the identifier of that object (i.e., value of the **FunctionDefinition**'s `id` attribute) cannot appear as the value of a `ci` element.

Such usage would entail a recursive function call, but SBML functions are not permitted to be recursive or mutually recursive. (References: SBML L3V1 Section 3.4.3 and 4.3.2; SBML L3V2 Section 3.4.3 and Section 4.3.2.)

20304. ☑ Inside the `lambda` MathML element within a **FunctionDefinition** object, if a `ci` element is not the first element within a MathML `apply`, then the `ci` element's value may only be an identifier provided as the value of a `bvar` element declared in that `lambda`. This restriction also applies to the `csymbol` objects for *time*, *avogadro*, *delay*, and *rateOf*. In other words, all model quantities and variables referenced inside a function definition must be passed as arguments to that function. (References: SBML L3V1 Section 3.4.3 and 4.3.2; SBML L3V2 Section 3.4.3 and Section 4.3.2.)

20305. ☑ The type of value returned by a **FunctionDefinition** object's `math` MathML expression must be either Boolean or numeric. (References: SBML L3V1 Section 3.4.9; SBML L3V2 Section 3.4.11.)

20306. ☑ A **FunctionDefinition** object may contain at most one MathML `math` element. (References: SBML L3V1 Section 4.3; SBML L3V1 Section 4.3; SBML L3V2 .)

20307. ☑ A **FunctionDefinition** object must have the required attribute `id`, and may have the optional attributes `metaid`, `sboTerm` and `name`. No other attributes from the SBML Level 3 Core namespace are permitted on a **FunctionDefinition** object. (References: SBML L3V1 Section 4.3; SBML L3V2 Section 4.3.)

20308. ☑ Inside the `lambda` MathML element within a **FunctionDefinition**, `bvar` elements must contain exactly one instance of a `ci` element. No other elements are permitted within a `bvar` element. (References: SBML L3V1 Section 4.3.2; SBML L3V2 Section 4.3.2.)

#### *Rules for Unit and UnitDefinition components*

20401. ☑ The value of the attribute `id` in a **UnitDefinition** object must not be identical to any unit predefined in SBML. That is, the identifier must not be the same as any of the following base units: "`ampere`", "`avogadro`", "`becquerel`", "`candela`", "`coulomb`", "`dimensionless`", "`farad`", "`gram`", "`gray`", "`henry`", "`hertz`", "`item`", "`joule`", "`katal`", "`kelvin`", "`kilogram`", "`litre`", "`lumen`", "`lux`", "`metre`", "`mole`", "`newton`", "`ohm`", "`pascal`", "`radian`", "`second`", "`siemens`", "`sievert`", "`steradian`", "`tesla`", "`volt`", "`watt`", or "`weber`". (References: SBML L3V1 Section 4.4.2; SBML L3V2 Section 4.4.2.)

20410. ☑ The value of the attribute `kind` of a **Unit** object must conform to the syntax of the SBML data type `UnitSId` and may only take on the value of a base unit defined in SBML; that is, the value must be one of the following units: "`ampere`", "`avogadro`", "`becquerel`", "`candela`", "`coulomb`", "`dimensionless`", "`farad`", "`gram`", "`gray`", "`henry`", "`hertz`", "`item`", "`joule`", "`katal`", "`kelvin`", "`kilogram`", "`litre`", "`lumen`", "`lux`", "`metre`", "`mole`", "`newton`", "`ohm`", "`pascal`", "`radian`", "`second`", "`siemens`", "`sievert`", "`steradian`", "`tesla`", "`volt`", "`watt`", or "`weber`". The SBML unit system is not hierarchical, and user-defined units cannot be defined using other user-defined units. (References: SBML L3V1 Section 4.4.2; SBML L3V2 Section 4.4.2.)

20413. ☑ (This validation rule does not apply in SBML Level 3 Version 2 Core.) [8]

20414. ☑ There may be at most one **ListOfUnits** container objects in a **UnitDefinition** object. (References: SBML L3V1 Section 4.4; SBML L3V2 Section 4.4.)

20415. ☑ Apart from the general notes and annotation subobjects permitted on all SBML components, a **ListOfUnits** container object may only contain **Unit** objects. (References: SBML L3V1 Section 4.2.8; SBML L3V2 Section 4.2.7.)

20416. ☑ The attribute `exponent` on a **Unit** object must have a value of type `double`. (References: SBML L3V1 Section 3.1.5; SBML L3V2 Section 3.1.5.)

[8] Original rule: "The **ListOfUnits** container object in a **UnitDefinition** object is optional, but if present, it must not be empty."

20417. ☑ The attribute `scale` on a **Unit** object must have a value of type `int`. (References: SBML L3V1 Section 3.1.3; SBML L3V2 Section 3.1.3.)

20418. ☑ The attribute `multiplier` on a **Unit** object must have a value of type `double`. (References: SBML L3V1 Section 3.1.5; SBML L3V2 Section 3.1.5.)

20419. ☑ A **UnitDefinition** object must have the required attribute `id` and may have the optional attributes `metaid`, `sboTerm` and `name`. No other attributes from the SBML Level 3 Core namespace are permitted on a **UnitDefinition** object. (References: SBML L3V1 Section 4.4; SBML L3V2 Section 4.4.)

20420. ☑ A **ListOfUnits** object may have the optional attributes `id`, `name`, `metaid` and `sboTerm`. No other attributes from the SBML Level 3 Core namespace are permitted on a **ListOfUnits** object. (References: SBML L3V1 Section 4.2.8; SBML L3V2 Section 4.2.7.)

20421. ☑ A **Unit** object must have the required attributes `kind`, `exponent`, `scale` and `multiplier`, and may have the optional attributes `id`, `name`, `metaid` and `sboTerm`. No other attributes from the SBML Level 3 Core namespace are permitted on a **Unit** object. (References: SBML L3V1 Section 4.4; SBML L3V2 Section 4.4.)

#### *Rules for Compartment components*

20507. ★ The value of the attribute `units` on a **Compartment** object having `spatialDimensions` of "`1`" should be either "`metre`", "`dimensionless`", or the identifier of a **UnitDefinition** object based on either `metre` or `dimensionless`, or a combination of these units. (References: SBML L3V1 Section 4.5.4; SBML L3V2 Section 4.5.4.)

20508. ★ The value of the attribute `units` on a **Compartment** object having `spatialDimensions` of "`2`" should be either "`dimensionless`", or the identifier of a **UnitDefinition** object based on either `metre` or `dimensionless`, or a combination of these units. (References: SBML L3V1 Section 4.5.4; SBML L3V2 Section 4.5.4.)

20509. ★ The value of the attribute `units` on a **Compartment** object having `spatialDimensions` of "`3`" should be either "`litre`", or the identifier of a **UnitDefinition** object based on either `litre`, `metre` or `dimensionless`, or a combination of these units. (References: SBML L3V1 Section 4.5.4; SBML L3V2 Section 4.5.4.)

20511. ★ If the attribute `units` on a **Compartment** object having a `spatialDimensions` attribute value of "`1`" has not been set, then the unit of measurement associated with the compartment's size is determined by the value of the enclosing **Model** object's `lengthUnits` attribute. If neither the **Compartment** object's `units` nor the enclosing **Model** object's `lengthUnits` attributes are set, the unit of compartment size is undefined. (References: SBML L3V1 Section 4.5.4; SBML L3V2 Section 4.5.4.)

20512. ★ If the attribute `units` on a **Compartment** object having a `spatialDimensions` attribute value of "`2`" has not been set, then the unit of measurement associated with the compartment's size is determined by the value of the enclosing **Model** object's `areaUnits` attribute. If neither the **Compartment** object's `units` nor the enclosing **Model** object's `areaUnits` attributes are set, the unit of compartment size is undefined. (References: SBML L3V1 Section 4.5.4; SBML L3V2 Section 4.5.4.)

20513. ★ If the attribute `units` on a **Compartment** object having a `spatialDimensions` attribute value of "`3`" has not been set, then the unit of measurement associated with the compartment's size is determined by the value of the enclosing **Model** object's `volumeUnits` attribute. If neither the **Compartment** object's `units` nor the enclosing **Model** object's `volumeUnits` attributes are set, the unit of compartment size is undefined. (References: SBML L3V1 Section 4.5.4; SBML L3V2 Section 4.5.4.)

20514. ☑ The attribute `spatialDimensions` on a **Compartment** object must have a value of type `double`. (References: SBML L3V1 Section 3.1.5; SBML L3V2 Section 3.1.5.)

20515. ☑ The attribute `size` on a **Compartment** object must have a value of type `double`. (References: SBML L3V1 Section 3.1.5; SBML L3V2 Section 3.1.5.)

20516. ☑ The attribute `constant` on a **Compartment** object must have a value of type `boolean`. (References: SBML L3V1 Section 3.1.2; SBML L3V2 Section 3.1.2.)

20517. ☑ A **Compartment** object must have the required attributes `id` and `constant`, and may have the optional attributes `metaid`, `sboTerm`, `name`, `spatialDimensions`, `size` and `units`. No other attributes from the SBML Level 3 Core namespace are permitted on a **Compartment** object. (References: SBML L3V1 Section 4.5; SBML L3V2 Section 4.5.)

20518. ★ If neither the attribute `units` nor the attribute `spatialDimensions` on a **Compartment** object is set, the unit associated with that compartment's size is undefined. (References: SBML L3V1 Section 4.5.4; SBML L3V2 Section 4.5.4)

#### *Rules for Species components*

20601. ☑ The value of the attribute `compartment` in a **Species** object must be the identifier of an existing **Compartment** object defined in the enclosing **Model** object. (References: SBML L3V1 Section 4.6.3; SBML L3V2 Section 4.6.3.)

20608. ★ The value of a **Species** object's `substanceUnits` attribute should only be one of the following: "`mole`", "`item`", "`gram`", "`kilogram`", "`dimensionless`", "`avogadro`" or the identifier of a **UnitDefinition** object derived from "`mole`", "`item`", "`gram`", "`kilogram`", "`avogadro`", or "`dimensionless`", or a combination of these units. (References: SBML L3V1 Section 4.6.5; SBML L3V2 Section 4.6.4.)

20609. ☑ A **Species** object cannot have values for both its `initialConcentration` and `initialAmount` attributes because these attributes are mutually exclusive. (References: SBML L3V1 Section 4.6.4; SBML L3V2 Section 4.6.4.)

20610. ☑ The quantity of a **Species** object in a model cannot be determined simultaneously by both reactions and rules. More formally, if the identifier of a **Species** object having attribute values `boundaryCondition`="`false`" and `constant`="`false`" is referenced by a **SpeciesReference** object anywhere in a model, then this identifier cannot also appear as the value of a `variable` in an **AssignmentRule** or a **RateRule** object. (References: SBML L3V1 Section 4.6.6; SBML L3V2 Section 4.6.6.)

20611. ☑ A **Species** object having a value of "`false`" for its attribute `boundaryCondition` cannot appear as a reactant or product in any reaction if that **Species** also has a value of "`true`" for its attribute `constant`. (References: SBML L3V1 Section 4.6.6; SBML L3V2 Section 4.6.6.)

20614. ☑ The attribute `compartment` in **Species** is mandatory. A **Species** object in a model must include a value for this attribute. (References: SBML L3V1 Section 4.6.3; SBML L3V2 Section 4.6.3.)

20616. ★ If the attribute `substanceUnits` in a **Species** object has not been set, then the unit of measurement associated with the species' quantity is determined by the value of the enclosing **Model** object's `substanceUnits` attribute. If neither the **Species** object's `substanceUnits` attribute nor the enclosing **Model** object's `substanceUnits` attribute are set, then the unit of that species' quantity is undefined. (References: SBML L3V1 Section 4.6.5; SBML L3V2 Section 4.6.5.)

20617. ☑ The value of the attribute `conversionFactor` on a **Species** object must be the identifier of an existing **Parameter** object defined in the enclosing **Model** object. (References: SBML L3V1 Section 4.6.7; SBML L3V2 Section 4.6.7.)

20618. ☑ The attribute `initialAmount` on a **Species** object must have a value of type `double`. (References: SBML L3V1 Section 3.1.5; SBML L3V2 Section 3.1.5.)

20619. ☑ The attribute `initialConcentration` on a **Species** object must have a value of type `double`. (References: SBML L3V1 Section 3.1.5; SBML L3V2 Section 3.1.5.)

20620. ☑ The attribute `hasOnlySubstanceUnits` on a **Species** object must have a value of type `boolean`. (References: SBML L3V1 Section 3.1.2; SBML L3V2 Section 3.1.2.)

20621. ☑ The attribute `boundaryCondition` on a **Species** object must have a value of type `boolean`. (References: SBML L3V1 Section 3.1.2; SBML L3V2 Section 3.1.2.)

20622. ☑ The attribute `constant` on a **Species** object must have a value of type `boolean`. (References: SBML L3V1 Section 3.1.2; SBML L3V2 Section 3.1.2.)

20623. ☑ A **Species** object must have the required attributes `id`, `compartment`, `hasOnlySubstanceUnits`, `boundaryCondition` and `constant`, and may have the optional attributes `metaid`, `sboTerm`, `name`, `initialAmount`, `initialConcentration`, `substanceUnits` and `conversionFactor`. No other attributes from the SBML Level 3 Core namespace are permitted on a **Species** object. (References: SBML L3V1 Section 4.6; SBML L3V2 Section 4.6.)

#### *Rules for Parameter components*

20701. ☑ *[Superceded by validation rule 10313.]*

20702. ★ If the attribute `units` on a given **Parameter** object has not been set, then the unit of measurement associated with that parameter's value is undefined. (References: SBML L3V1 Section 4.7.3; SBML L3V2 Section 4.7.3.)

20703. ☑ The attribute `value` on a **Parameter** object must have a value of type `double`. (References: SBML L3V1 Section 3.1.5; SBML L3V2 Section 3.1.5.)

20704. ☑ The attribute `constant` on a **Parameter** object must have a value of type `boolean`. (References: SBML L3V1 Section 3.1.2; SBML L3V2 Section 3.1.2.)

20705. ☑ A **Parameter** object referenced by the attribute `conversionFactor` on a **Species** or **Model** object must have a value of "`true`" for its attribute `constant`. (References: SBML L3V1 Section 4.6.7; SBML L3V2 Section 4.6.7.)

20706. ☑ A **Parameter** object must have the required attributes `id` and `constant`, and may have the optional attributes `metaid`, `sboTerm`, `name`, `value` and `units`. No other attributes from the SBML Level 3 Core namespace are permitted on a **Parameter** object. (References: SBML L3V1 Section 4.7; SBML L3V2 Section 4.7.)

#### *Rules for InitialAssignment components*

20801. ☑ The value of the attribute `symbol` in an **InitialAssignment** object must be the identifier of an existing **Compartment**, **Species**, **Parameter** or **SpeciesReference** object defined in the model, or any identifier in the SId namespace of the model belonging to an element defined by an SBML Level 3 package as having with mathematical meaning. (References: SBML L3V1 Section 4.8; SBML L3V2 Section 4.8.)

20802. ☑ A given identifier cannot appear as the value of more than one **InitialAssignment** object's `symbol` attribute across the set of all **InitialAssignment** objects in a model. (References: SBML L3V1 Section 4.8; SBML L3V2 Section 4.8.)

20803. ☑ The identifier given as the value of the attribute `symbol` in any **InitialAssignment** object cannot also appear as the value of the `variable` attribute in an **AssignmentRule** object. In other words, a model cannot simultaneously define both an initial assignment and an assignment rule for the same species, compartment or parameter in a model. (References: SBML L3V1 Section 4.8; SBML L3V2 Section 4.8.)

20804. ☑ An **InitialAssignment** object may contain at most one MathML `math` element. (References: SBML L3V1 Section 4.8; SBML L3V2 Section 4.8.)

20805. ☑ An **InitialAssignment** object must have the required attribute `symbol` and may have the optional attributes `id`, `name`, `metaid` and `sboTerm`. No other attributes from the SBML Level 3 Core namespace are permitted on an **InitialAssignment** object. (References: SBML L3V1 Section 4.8; SBML L3V2 Section 4.8.)

#### *Rules for AssignmentRule, RateRule and AlgebraicRule components*

20901. ☑ The value of an **AssignmentRule** object's `variable` attribute must be the identifier of an existing **Compartment**, **Species**, **Parameter** or **SpeciesReference** object defined in the model, or any identifier in the SId namespace of the model belonging to an element defined by an SBML Level 3 package as having with mathematical meaning, and that is allowed to change over the course of a simulation. (References: SBML L3V1 Section 4.9.3; SBML L3V2 Section 4.9.3.)

20902. ☑ The value of a **RateRule** object's `variable` attribute must be the identifier of an existing **Species**, **Compartment**, **Parameter** or **SpeciesReference** object defined in the model, or any identifier in the SId namespace of the model belonging to an element defined by an SBML Level 3 package as having with mathematical meaning, and that is allowed to change over the course of a simulation. (References: SBML L3V1 Section 4.9.4; SBML L3V2 Section 4.9.4.)

20903. ☑ Any **Compartment**, **Species**, **Parameter** or **SpeciesReference** object whose identifier is the value of the attribute `variable` in an **AssignmentRule** object, must have a value of "`false`" for its `constant` attribute. (References: SBML L3V1 Section 4.9.3; SBML L3V2 Section 4.9.3.)

20904. ☑ Any **Compartment**, **Species**, **Parameter** or **SpeciesReference** object whose identifier is the value of the attribute `variable` in a **RateRule** object, must have a value of "`false`" for its `constant` attribute. (References: SBML L3V1 Section 4.9.4; SBML L3V2 Section 4.9.4.)

20906. ☑ There must not be circular dependencies in the combined set of **InitialAssignment**, **AssignmentRule** and **KineticLaw** objects in a model. Each of these constructs has the effect of assigning a value to an identifier (i.e., the identifier given in the attribute `symbol` in **InitialAssignment**, the attribute `variable` in **AssignmentRule**, and the attribute `id` on the **KineticLaw**'s enclosing **Reaction**). Each of these constructs computes the value using a mathematical formula. The formula for a given identifier cannot make reference to a second identifier whose own definition depends directly or indirectly on the first identifier. (References: SBML L3V1 Section 4.9.5; SBML L3V2 Section 4.9.5.)

20907. ☑ Every **AssignmentRule**, **RateRule** and **AlgebraicRule** object may contain at most one MathML `math` element. (References: SBML L3V1 Section 4.9; SBML L3V2 Section 4.9.)

20908. ☑ An **AssignmentRule** object must have the required attribute `variable` and may have the optional attributes `id`, `name`, `metaid` and `sboTerm`. No other attributes from the SBML Level 3 Core namespace are permitted on an **AssignmentRule** object. (References: SBML L3V1 Section 4.9; SBML L3V2 Section 4.9.)

20909. ☑ A **RateRule** object must have the required attribute `variable` and may have the optional attributes `id`, `name`, `metaid` and `sboTerm`. No other attributes from the SBML Level 3 Core namespace are permitted on a **RateRule** object. (References: SBML L3V1 Section 4.9; SBML L3V2 Section 4.9.)

20910. ☑ An **AlgebraicRule** object may have the optional attributes `id`, `name`, `metaid` and `sboTerm`. No other attributes from the SBML Level 3 Core namespace are permitted on an **AlgebraicRule** object. (References: SBML L3V1 Section 4.9; SBML L3V2 Section 4.9.)

20912. ☑ There must not be circular dependencies in calculating the rate of change of any model variable. The *rateOf* `csymbol` may not be used directly nor indirectly in the calculation of the rate of change of the variable it references. In particular, the **RateRule** and **KineticLaw** constructs are used to calculate rates of change: the formulas they use may not contain *rateOf* `csymbols` referencing the model variables whose rates of change they control, nor may they reference variables who in turn are calculated (directly nor indirectly) by using a *rateOf* `csymbol` referencing those same changing variables. (Reference: SBML L3V2 Section 4.9.5)

#### *Rules for Constraint components*

21001. ☑ (This validation rule does not apply in SBML Level 3 Version 2 Core.) [9]

21004. ☑ The contents of the **Message** subobject in a **Constraint** object must not contain an XML declaration (i.e., a string of the form "`<?xml version="1.0" encoding="UTF-8"?>`" or similar). (References: SBML L3V1 Section 4.10.2; SBML L3V2 Section 4.10.2.)

21005. ☑ The contents of the **Message** subobject in a **Constraint** object must not contain an XML DOCTYPE declaration (i.e., a string beginning with the characters "`<!DOCTYPE`". (References: SBML L3V1 Section 4.10.2; SBML L3V2 Section 4.10.2.)

21007. ☑ A **Constraint** object may contain at most one MathML `math` element. (References: SBML L3V1 Section 4.10; SBML L3V2 Section 4.10.)

21008. ☑ A **Constraint** object may contain at most one **Message** subobject. (References: SBML L3V1 Section 4.10; SBML L3V2 Section 4.10.)

21009. ☑ A **Constraint** object may have the optional attributes `id`, `name`, `metaid` and `sboTerm`. No other attributes from the SBML Level 3 Core namespace are permitted on a **Constraint** object. (References: SBML L3V1 Section 4.10; SBML L3V2 Section 4.10.)

#### *Rules for Reaction components*

21101. ☑ (This validation rule does not apply in SBML Level 3 Version 2 Core.) [10]

21103. ☑ (This validation rule does not apply in SBML Level 3 Version 2 Core.) [11]

21104. ☑ Apart from the general notes and annotation subobjects permitted on all SBML components, the **ListOfSpeciesReferences** container objects (i.e., the **Reaction** elements `listOfReactants` and `listOfProducts`) may only contain **SpeciesReference** objects. (References: SBML L3V1 Section 4.11; SBML L3V2 Section 4.11.)

21105. ☑ Apart from the general notes and annotation subobjects permitted on all SBML components, **ListOfModifierSpeciesReferences** container objects (i.e., the **Reaction** element `listOfModifiers`) may only contain **ModifierSpeciesReference** objects. (References: SBML L3V1 Section 4.11; SBML L3V2 Section 4.11.)

21106. ☑ A **Reaction** object may contain at most one of each of the following elements: `listOfReactants`, `listOfProducts`, `listOfModifiers`, and `kineticLaw`. (References: SBML L3V1 Section 4.11; SBML L3V2 Section 4.11.)

21107. ☑ The value of the attribute `compartment` in a **Reaction** object is optional, but if present, must be the identifier of an existing **Compartment** object defined in the model. (References: SBML L3V1 Section 4.11.1; SBML L3V2 Section 4.11.1.)

21108. ☑ The attribute `reversible` on a **Reaction** object must have a value of type `boolean`. (References: SBML L3V1 Section 3.1.2; SBML L3V2 Section 3.1.2.)

21109. ☑ (This validation rule does not apply in SBML Level 3 Version 2 Core.) [12]

[9] Original rule: "The MathML `math` element in a **Constraint** object must evaluate to a value of type `boolean`."

[10] Original rule: "A **Reaction** object must contain at least one **SpeciesReference** object, either in its `listOfReactants` or its `listOfProducts` element. A reaction without any reactant or product species is not permitted, regardless of whether the reaction has any modifier species."

[11] Original rule: "The following are all optional in a **Reaction** object, but if any is present, it must not be empty: **KineticLaw**, the elements `listOfReactants` and `listOfProducts` (both **ListOfSpeciesReferences** objects) and the element `listOfModifiers` (a **ListOfModifierSpeciesReferences** object)."

[12] Original rule: "The attribute `fast` on a **Reaction** object must have a value of type `boolean`."

21110. ☑ A **Reaction** object must have the required attributes `id` and `reversible`, and may have the optional attributes `metaid`, `sboTerm`, `name` and `compartment`. No other attributes from the SBML Level 3 Core namespace are permitted on a **Reaction** object. (References: SBML L3V1 Section 4.11; SBML L3V2 Section 4.11.)

21150. ☑ A **ListOfSpeciesReferences** object may have the optional attributes `id`, `name`, `metaid` and `sboTerm`. No other attributes from the SBML Level 3 Core namespace are permitted on a **ListOfSpeciesReferences** object. (References: SBML L3V1 Section 4.11; SBML L3V2 Section 4.11.)

21151. ☑ A **ListOfModifierSpeciesReferences** object may have the optional attributes `id`, `name`, `metaid` and `sboTerm`. No other attributes from the SBML Level 3 Core namespace are permitted on an object of class **ListOfModifierSpeciesReferences**. (References: SBML L3V1 Section 4.11; SBML L3V2 Section 4.11.)

#### *Rules for SpeciesReference and ModifierSpeciesReference components*

21111. ☑ The value of a **SpeciesReference** object's `species` attribute must be the identifier of an existing **Species** object in the model. (References: SBML L3V1 Section 4.11.3; SBML L3V2 Section 4.11.3.)

21114. ☑ The attribute `stoichiometry` on a **SpeciesReference** object must have a value of type `double`. (References: SBML L3V1 Section 3.1.5; SBML L3V2 Section 3.1.5.)

21115. ☑ The attribute `constant` on a **SpeciesReference** object must have a value of type `boolean`. (References: SBML L3V1 Section 3.1.2; SBML L3V2 Section 3.1.2.)

21116. ☑ A **SpeciesReference** object must have the required attributes `species` and `constant`, and may have the optional attributes `metaid`, `sboTerm`, `id`, `name` and `stoichiometry`. No other attributes from the SBML Level 3 Core namespace are permitted on a **SpeciesReference** object. (References: SBML L3V1 Section 4.11; SBML L3V2 Section 4.11.)

21117. ☑ A **ModifierSpeciesReference** object must have the required attribute `species` and may have the optional attributes `metaid`, `sboTerm`, `id` and `name`. No other attributes from the SBML Level 3 Core namespace are permitted on a **ModifierSpeciesReference** object. (References: SBML L3V1 Section 4.11; SBML L3V2 Section 4.11.)

#### *Rules for KineticLaw components*

21121. ☑ All **Species** objects referenced in the MathML `math` element of a **KineticLaw** object within a given **Reaction** object must first be declared using **SpeciesReference** or **ModifierSpeciesReference** objects. In other words, if a **Species** object identifier appears in a MathML `ci` element within the **Reaction**'s **KineticLaw** `math` content, that same species' identifier must also appear in at least one object of type **SpeciesReference** or **ModifierSpeciesReference** within the `listOfReactants`, `listOfProducts` and/or `listOfModifiers` elements of the **Reaction** object. (References: SBML L3V1 Section 4.11.5; SBML L3V2 Section 4.11.5.)

21123. ☑ (This validation rule does not apply in SBML Level 3 Version 2 Core.) [13]

21127. ☑ A **KineticLaw** object may contain at most one **ListOfLocalParameters** container object. (References: SBML L3V1 Section 4.11; SBML L3V2 Section 4.11.)

21128. ☑ Apart from the general notes and annotation subobjects permitted on all SBML components, a **ListOfLocalParameters** container object may only contain **LocalParameter** objects. (References: SBML L3V1 Section 4.2.8; SBML L3V2 Section 4.2.7.)

21129. ☑ A **ListOfLocalParameters** object may have the optional attributes `id`, `name`, `metaid` and `sboTerm`. No other attributes from the SBML Level 3 Core namespace are permitted on a **ListOfLocalParameters** object. (References: SBML L3V1 Section 4.11; SBML L3V2 Section 4.11.)

[13] Original rule: "The **ListOfLocalParameters** container object in a **KineticLaw** object is optional, but if present, it must not be empty."

21130. ☑ A **KineticLaw** object may contain at most one MathML `math` element. (References: SBML L3V1 Section 4.11; SBML L3V2 Section 4.11.)

21132. ☑ A **KineticLaw** object may have the optional attributes `id`, `name`, `metaid` and `sboTerm`. No other attributes from the SBML Level 3 Core namespace are permitted on a **KineticLaw**. (References: SBML L3V1 Section 4.11; SBML L3V2 Section 4.11.)

#### *Rules for LocalParameter components*

21171. ☑ The attribute `value` on a **LocalParameter** object must have a value of type `double`. (References: SBML L3V1 Section 3.1.5; SBML L3V2 Section 3.1.5.)

21172. ☑ A **LocalParameter** object must have the required attribute `id` and may have the optional attributes `id`, `name`, `metaid`, `sboTerm`, `name`, `value` and `units`. No other attributes from the SBML Level 3 Core namespace are permitted on a **LocalParameter** object. (References: SBML L3V1 Section 4.11; SBML L3V2 Section 4.11.)

21173. ☑ The `id` of a **LocalParameter** object must not be the same as the `species` attribute of any **SpeciesReference** in the same **Reaction**. (References: SBML L3V2 Section 4.11.6.)

21174. ★ If the attribute `units` on a given **LocalParameter** object has not been set, then the unit of measurement associated with that parameter's value is undefined. (References: SBML L3V1 Section 4.11.6; SBML L3V2 Section 4.11.6.)

#### *Rules for Event components*

21201. ☑ An **Event** object may contain at most one **Trigger** object. (References: SBML L3V1 Section 4.12.2; SBML L3V2 Section 4.12.2.)

21202. ☑ (This validation rule does not apply in SBML Level 3 Version 2 Core.) [14]

21203. ☑ (This validation rule does not apply in SBML Level 3 Version 2 Core.) [15]

21208. ☑ The attribute `useValuesFromTriggerTime` on an **Event** object must have a value of type `boolean`. (References: SBML L3V1 Section 3.1.2; SBML L3V2 Section 3.1.2.)

21209. ☑ A **Trigger** object may contain at most one MathML `math` element. (References: SBML L3V1 Section 4.12; SBML L3V2 Section 4.12.)

21210. ☑ A **Delay** object may contain at most one MathML `math` element. (References: SBML L3V1 Section 4.12; SBML L3V2 Section 4.12.)

21221. ☑ An **Event** object may contain at most one **Delay** object. (References: SBML L3V1 Section 4.12; SBML L3V2 Section 4.12.)

21222. ☑ An **Event** object may contain at most one **ListOfEventAssignments** object. (References: SBML L3V1 Section 4.12; SBML L3V2 Section 4.12.)

21223. ☑ Apart from the general notes and annotation subobjects permitted on all SBML components, a **ListOfEventAssignments** container object may only contain **EventAssignment** objects. (References: SBML L3V1 Section 4.12.5; SBML L3V2 Section 4.12.5.)

21224. ☑ A **ListOfEventAssignments** object may have the optional attributes `id`, `name`, `metaid` and `sboTerm`. No other attributes from the SBML Level 3 Core namespace are permitted on a **ListOfEventAssignments** object. (References: SBML L3V1 Section 4.12.5; SBML L3V2 Section 4.12.5.)

[14] Original rule: "The MathML `math` element of a **Trigger** object must evaluate to a value of type `boolean`."
[15] Original rule: "The **ListOfEventAssignments** container object in an **Event** object is optional, but if present, it must not be empty."

21225. ☑ An **Event** object must have the required attribute `useValuesFromTriggerTime`, and in addition, may have the optional attributes `metaid`, `sboTerm`, `id`, and `name`. No other attributes from the SBML Level 3 Core namespace are permitted on an **Event** object. (References: SBML L3V1 Section 4.12; SBML L3V2 Section 4.12.)

21226. ☑ A **Trigger** object must have the required attributes `persistent` and `initialValue`, and in addition, may have the optional attributes `id`, `name`, `metaid` and `sboTerm`. No other attributes from the SBML Level 3 Core namespace are permitted on a **Trigger** object. (References: SBML L3V1 Section 4.12; SBML L3V2 Section 4.12.)

21227. ☑ A **Delay** object may have the optional attributes `id`, `name`, `metaid` and `sboTerm`. No other attributes from the SBML Level 3 Core namespace are permitted on a **Delay** object. (References: SBML L3V1 Section 4.12; SBML L3V2 Section 4.12.)

21228. ☑ The attribute `persistent` on an **Trigger** object must have a value of type `boolean`. (References: SBML L3V1 Section 3.1.2; SBML L3V2 Section 3.1.2.)

21229. ☑ The attribute `initialValue` on a **Trigger** object must have a value of type `boolean`. (References: SBML L3V1 Section 3.1.2; SBML L3V2 Section 3.1.2.)

21230. ☑ An **Event** object may contain at most one **Priority** object. (References: SBML L3V1 Section 4.12; SBML L3V2 Section 4.12.)

21231. ☑ A **Priority** object may contain at most one MathML `math` element. (References: SBML L3V1 Section 4.12; SBML L3V2 Section 4.12.)

21232. ☑ A **Priority** object may have the optional attributes `id`, `name`, `metaid` and `sboTerm`. No other attributes from the SBML Level 3 Core namespace are permitted on a **Priority** object. (References: SBML L3V1 Section 4.12; SBML L3V2 Section 4.12.)

#### *Rules for EventAssignment components*

21211. ☑ The value of the attribute `variable` in an **EventAssignment** object may only be the identifier of an existing **Compartment**, **Species**, **Parameter** or **SpeciesReference** object in the model, or any identifier in the SId namespace of the model belonging to an element defined by an SBML Level 3 package as having with mathematical meaning, and that is allowed to change over the course of a simulation. (References: SBML L3V1 Section 4.12.5; SBML L3V2 Section 4.12.5.)

21212. ☑ Any **Compartment**, **Species**, **Parameter** or **SpeciesReference** object whose identifier is used as the value of the attribute `variable` of an **EventAssignment** object, must have a value of "`false`" for its `constant` attribute. (References: SBML L3V1 Section 4.12.5; SBML L3V2 Section 4.12.5.)

21213. ☑ An **EventAssignment** object may contain at most one MathML `math` element. (References: SBML L3V1 Section 4.12; SBML L3V2 Section 4.12.)

21214. ☑ An **EventAssignment** object must have the required attribute `variable` and may have the optional attributes `id`, `name`, `metaid` and `sboTerm`. No other attributes from the SBML Level 3 Core namespace are permitted on an **EventAssignment** object. (References: SBML L3V1 Section 4.12; SBML L3V2 Section 4.12.)

## B A method for assessing whether an SBML model is overdetermined

As explained in Section 4.9.5 on p. 64, an SBML model must not be overdetermined. It is possible to use purely static analysis to assess this condition for the system of equations implied by a model, by constructing a bipartite graph of the model's variables and equations and then searching for a maximal matching (Chartrand, 1977). An efficient algorithm for finding a maximal matching is described by Hopcroft and Karp (1973). In this appendix, we provide a concrete application to SBML of the general approach described in Section 4.9.5 on p. 64. The approach is defined in terms of the ordinary differential equations (ODEs) implied by an SBML model; despite our use of a differential equation framework for this explanation, it should be understood that this use of ODEs has no implication about the framework actually used to simulate the model.

### Definition of the method

First, we assume that an ODE is constructed for each species determined by one or more **Reaction**'s **KineticLaw** `math` expressions. We also assume that the model has already been determined to be valid in all other respects (e.g., there are no undefined variables in the equations), and what remains is to evaluate whether it is overdetermined.

We construct the bipartite graph for a given SBML model as follows:

1. For each of the following in the model, create one vertex representing an equation:
   (a) Every **Species** object having `boundaryCondition`="`false`", `constant`="`false`", and which is referenced as a reactant or product in one or more **Reaction** objects containing **KineticLaw** objects
   (b) Every **AssignmentRule** object
   (c) Every **RateRule** object
   (d) Every **AlgebraicRule** object
   (e) Every **KineticLaw** object
2. For each of the following in the model, create one vertex representing a variable:
   (a) Every **Species** object having `constant`="`false`"
   (b) Every **Compartment** object having `constant`="`false`"
   (c) Every global **Parameter** having `constant`="`false`"
   (d) Every **SpeciesReference** object having `constant`="`false`"
   (e) Every **Reaction** object
3. For each of the following, create one edge:
   (a) Every vertex created in step 2(a) to that species' equation vertex created in step 1(a)
   (b) Every vertex created in step 1(b) to the particular vertex created in steps 2(a)–2(e) that represents the variable referenced by the `variable` attribute of the rule
   (c) Every vertex created in step 1(c) to the particular vertex created in steps 2(a)–2(e) that represents the variable referenced by the `variable` attribute of the rule
   (d) Every vertex created in step 1(e) to the particular vertex created in step 2(e) that is the **Reaction** object containing that particular **KineticLaw** object
   (e) Every vertex created in steps 2(a)–2(e) representing an identifier appearing as the content of a MathML `ci` element within an expression of an **AlgebraicRule**, to the vertex for that particular **AlgebraicRule** created in step 1(d)

### Example application of the method

What follows is an example of applying the method above to the SBML model shown below:

```
<?xml version="1.0" encoding="UTF-8"?>
<sbml xmlns="http://www.sbml.org/sbml/level3/version2/core" level="3" version="2">
    <model id="example" substanceUnits="mole" volumeUnits="litre"
           timeUnits="second" extentUnits="mole">
        <listOfUnitDefinitions>
            <unitDefinition id="per_time">
                <listOfUnits>
                    <unit kind="second" exponent="-1" scale="0" multiplier="1"/>
                </listOfUnits>
            </unitDefinition>
        </listOfUnitDefinitions>
        <listOfCompartments>
            <compartment id="C" size="1" spatialDimensions="3" constant="true"/>
        </listOfCompartments>
        <listOfSpecies>
            <species id="S1" compartment="C" initialConcentration="1" constant="false"
                             boundaryCondition="false" hasOnlySubstanceUnits="false"/>
            <species id="S2" compartment="C" initialConcentration="0" constant="false"
                             boundaryCondition="false" hasOnlySubstanceUnits="false"/>
        </listOfSpecies>
        <listOfRules>
            <algebraicRule>
                <math xmlns="http://www.w3.org/1998/Math/MathML">
                    <apply>
                        <minus/>
                        <apply> <plus/> <ci> S1 </ci> <ci> S2 </ci> </apply>
                        <cn> 5 </cn>
                    </apply>
                </math>
            </algebraicRule>
        </listOfRules>
        <listOfReactions>
            <reaction id="R" reversible="false">
                <listOfReactants>
                    <speciesReference species="S1" stoichiometry="1" constant="true"/>
                </listOfReactants>
                <listOfProducts>
                    <speciesReference species="S2" stoichiometry="1" constant="true"/>
                </listOfProducts>
                <kineticLaw>
                    <math xmlns="http://www.w3.org/1998/Math/MathML">
                        <apply> <times/> <ci> C </ci> <ci> k1 </ci> <ci> S1 </ci> </apply>
                    </math>
                    <listOfLocalParameters>
                        <localParameter id="k1" value="0.1" units="per_time"/>
                    </listOfLocalParameters>
                </kineticLaw>
            </reaction>
        </listOfReactions>
    </model>
</sbml>
```

For the model above, we create *equation* vertices as follows:

1. [Corresponding to step 1(a) in Section B on the preceding page.] *For every* ***Species*** *object which has* `boundaryCondition`*="*`false`*",* `constant`*="*`false`*", and which is referenced as a reactant or product in one or more* ***Reaction*** *objects containing* ***KineticLaw*** *objects.* This generates two vertices, for "`S1`" and "`S2`".

2. [Corresponding to step 1(b) in Section B on the previous page.] *Every* ***AlgebraicRule*** *object.* This generates one vertex, for the model's lone algebraic rule (call it "`rule`").

3. [Corresponding to step 1(e) in Section B on the preceding page.] *Every* ***KineticLaw*** *object.* This generates one vertex, for the lone kinetic law in the model (call it "`law`").

We create *variable* vertices for the following:

1. [Corresponding to step 2(a) in Section B on p. 175.] *Every* ***Species*** *object having* `constant`=*"*`false`*"*. This generates two vertices, for "`S1`" and "`S2`".

2. [Corresponding to step 2(e) in Section B on p. 175.] *Every* ***Reaction*** *object.* This generates one vertex, for "`R`".

Note that it is not necessary to include parameters declared within **KineticLaw** objects because they are local to a particular reaction and cannot be affected by rules. With the steps above, we have the following set of graph nodes:

*Vertices for equations*

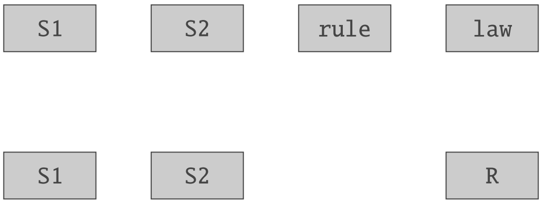

*Vertices for variables*

Next, we create edges following the procedure described above. Doing so results in the following graph:

*Vertices for equations*

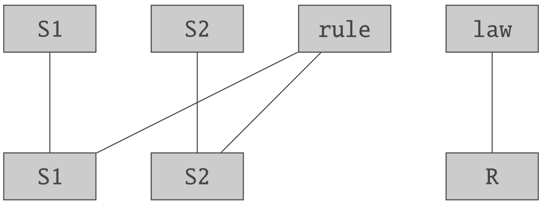

*Vertices for variables*

The algorithm of Hopcroft and Karp (1973) can now be applied to search for a maximal matching of the bipartite graph. A maximal matching is a graph in which each vertex is connected to at most one other vertex and the maximum possible number of connections have been made. Doing so here results in the following:

*Vertices for equations*

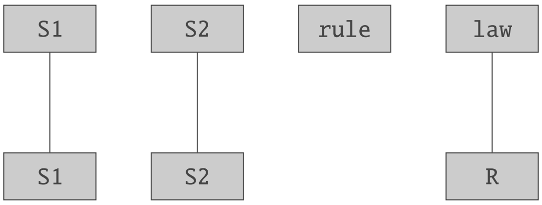

*Vertices for variables*

If the maximal matching of the bipartite graph leaves any equation vertex unconnected, then the model is considered overdetermined. That is the case for the example shown here, because the equation vertex for "`rule`" is unconnected in the maximal matching.

## C A mathematical technique for maintaining unit consistency in a kinetic law with variable stoichiometry

*(Appendix contributed by Chris Myers, University of Utah.)*

Section 4.11.3 on p. 71 describes how the stoichiometry of a **SpeciesReference** can be changed as part of a simulation. This can be useful in some cases, such as reactions where the stoichiometry depends upon pH.

However, it can be difficult to get the **KineticLaw** of such reactions to maintain the correct units as the stoichiometry of the reaction changes. For example, let us assume that we are modeling the following set of chemical reactions:

$$A + A \underset{k_\mathrm{r}}{\overset{k_\mathrm{f}}{\rightleftharpoons}} A_2$$

$$A_2 + A \underset{k_\mathrm{r}}{\overset{k_\mathrm{f}}{\rightleftharpoons}} A_3$$

$$\ldots$$

$$A_{n-1} + A \underset{k_\mathrm{r}}{\overset{k_\mathrm{f}}{\rightleftharpoons}} A_n$$

where we would like to allow $n$ to be a variable. In order to allow for this, we can approximate the above set of equations with a single equation as shown below using a *quasi-steady state approximation* (namely that the species $A_2, \ldots A_{n-1}$ have a short lifetime).

$$nA \underset{k_\mathrm{r}}{\overset{\mathrm{k_r} \cdot \mathrm{K_{eq}}^{\mathrm{n-1}}}{\rightleftharpoons}} A_n$$

where $K_\mathrm{eq}$ is equal to $k_\mathrm{f}/k_\mathrm{r}$. The rate law for the above reaction is:

$$k_\mathrm{r} \cdot {K_\mathrm{eq}}^{n-1} \cdot A^n - k_\mathrm{r} \cdot A_n$$

Let us assume that all species $A$, $A_2$, ..., $A_n$ have units of mole, $k_\mathrm{f}$ is in units of $(\mathrm{mole} \cdot \mathrm{second})^{-1}$, and $k_\mathrm{r}$ is in units of $\mathrm{second}^{-1}$. Therefore, $K_\mathrm{eq}$ is in units of $\mathrm{mole}^{-1}$ which makes the rate law above have units of mole/second as desired, regardless of the value of $n$, the stoichiometry of $A$.

### Acknowledgments

The development of SBML was originally funded by the Japan Science and Technology Agency (JST) under the ERATO Kitano Symbiotic Systems Project during the years 2000–2003. From 2003 to the present, funding for development of SBML and associated software such as libSBML and the SBML Test Suite has been provided chiefly by the National Institute of General Medical Sciences (USA) via grant numbers GM070923 and GM077671. Additional grant funding has in the past been provided by National Human Genome Research Institute (USA); the International Joint Research Program of NEDO (Japan); the JST ERATO-SORST Program (Japan); the Japanese Ministry of Agriculture; the Japanese Ministry of Education, Culture, Sports, Science and Technology; the BBSRC e-Science Initiative (UK); the DARPA IPTO Bio-Computation Program (USA); the Army Research Office's Institute for Collaborative Biotechnologies (USA); and the Air Force Office of Scientific Research (USA).

Additional support has been or continues to be provided by the following institutions, either directly for activities related to SBML or indirectly by supporting the work of present and past SBML Editors: the Beckman Institute at the California Institute of Technology (USA), EML Research gGmbH (Germany), the University of Heidelberg (Germany), the European Molecular Biology Laboratory's European Bioinformatics Institute (UK), the Molecular Sciences Institute (USA), the University of Hertfordshire (UK), the University of Newcastle (UK), the Systems Biology Institute (Japan), and the Virginia Bioinformatics Institute (USA). Fengkai Zhang was supported by the intramural program of NIAID, NIH.

The following individuals served as past SBML Editors and authors of SBML specifications. Their efforts helped shape what SBML is today:

- Hamid Bolouri
- Andrew M. Finney
- Herbert M. Sauro

SBML was first conceived at the JST/ERATO-sponsored *First Workshop on Software Platforms for Systems Biology*, held in April, 2000, at the California Institute of Technology in Pasadena, California, USA. The participants collectively decided to begin developing a common XML-based declarative language for representing models. The development and evolution of the Systems Biology Markup Language has continued ever since. Many discussions are archived online in the mailing list/forums area of `http://sbml.org`; many more discussions took place during meetings and workshops (a list of which is also available at `http://sbml.org`).

SBML Level 3 has benefitted from so many contributions, large and small, by so many people who constitute the international *SBML Forum*, that we regret it has become infeasible to list individuals by name. We thank everyone who has participated in SBML's development throughout the years, and we hope that this latest specification before you is a good step forward in SBML's continued evolution.
